# IL-17A Increases Doxorubicin Efficacy in Triple Negative Breast Cancer

**DOI:** 10.3389/fonc.2022.928474

**Published:** 2022-07-18

**Authors:** Nicholas R. Hum, Aimy Sebastian, Kelly A. Martin, Naiomy D. Rios-Arce, Sean F. Gilmore, David M. Gravano, Elizabeth K. Wheeler, Matthew A. Coleman, Gabriela G. Loots

**Affiliations:** ^1^ Physical and Life Sciences Directorate, Lawrence Livermore National Laboratory, Livermore, CA, United States; ^2^ Stem Cell Instrumentation Foundry, University of California Merced, Merced, CA, United States; ^3^ Engineering Directorate, Lawrence Livermore National Laboratory, Livermore, CA, United States; ^4^ Department of Radiation Oncology, University of California Davis, Sacramento, CA, United States

**Keywords:** triple negative breast cancer, doxorubicin, IL-17A, γδ T cells, chemoresistance, 4T1, single cell RNA seq

## Abstract

Due to lack of targetable receptors and intertumoral heterogeneity, triple negative breast cancer (TNBC) remains particularly difficult to treat. Doxorubicin (DOX) is typically used as nonselective neoadjuvant chemotherapy, but the diversity of treatment efficacy remains unclear. Comparable to variability in clinical response, an experimental model of TNBC using a 4T1 syngeneic mouse model was found to elicit a differential response to a seven-day treatment regimen of DOX. Single-cell RNA sequencing identified an increase in T cells in tumors that responded to DOX treatment compared to tumors that continued to grow uninhibited. Additionally, compared to resistant tumors, DOX sensitive tumors contained significantly more CD4 T helper cells (339%), γδ T cells (727%), Naïve T cells (278%), and activated CD8 T cells (130%). Furthermore, transcriptional profiles of tumor infiltrated T cells in DOX responsive tumors revealed decreased exhaustion, increased chemokine/cytokine expression, and increased activation and cytotoxic activity. γδ T cell derived IL-17A was identified to be highly abundant in the sensitive tumor microenvironment. IL-17A was also found to directly increase sensitivity of TNBC cells in combination with DOX treatment. In TNBC tumors sensitive to DOX, increased IL-17A levels lead to a direct effect on cancer cell responsiveness and chronic stimulation of tumor infiltrated T cells leading to improved chemotherapeutic efficacy. IL-17A’s role as a chemosensitive cytokine in TNBC may offer new opportunities for treating chemoresistant breast tumors and other cancer types.

## Introduction

The aggressiveness and lack of targetable receptors in triple negative breast cancer (TNBC) pose significant clinical challenges in treating this disease. Additionally, the frequent late-stage of diagnosis further complicates current therapeutic success. Standard of care for TNBC is a combination of surgery, radiation, and systemic chemotherapy, depending on genetic drivers and the severity of disease progression. Systemic, non-specific cytotoxic chemotherapy remains a common treatment approach, yet no clinically significant differences in efficacy have been established between neoadjuvant (prior to surgical intervention) or adjuvant (post-surgical) administration (1). Standard chemotherapeutic therapy is comprised of an anthracycline (doxorubicin, daunorubicin, epirubicin) and a taxane (paclitaxel, docetaxel) given in sequence to avoid excessive toxicity (2). Anthracyclines exhibit their efficacy *via* numerous established mechanisms of action; these include inhibition of topoisomerase II, DNA intercalation, and generation of reactive oxygen species (3). However, regardless of treatment, resistance to chemotherapy in TNBC is prevalent. Mechanisms of resistance include altered metabolism and upregulation of ATP-binding cassette transporters (4–6) and pose a significant challenge to patients as late stage TNBC has poor prognosis (up to 20% response rate) and very low median progression-free survival (~4.2 months) (7, 8).

The inability to effectively treat TNBC may in part be attributed to disease heterogeneity. Thus, innovative therapies focus on finding subtype-specific treatment regimens and determining subtype vulnerabilities that can be targeted and customized to the patient (9). Prior studies have shown that doxorubicin (DOX) therapy modulates both the cancer and the stromal cells found in the tumor microenvironment (TME), independently. For example, neutrophil exposure to anthracycline drugs resulted in the suppression of extracellular traps of DNA-protein complexes which may affect the cytotoxic and inflammatory response to DOX (10). Furthermore, regulation of neutrophil phenotypes (specifically down regulation of CD133 and CD309 *via* administration of berberine) has been shown to increase cancer cell sensitivity to DOX (11). Other myeloid lineages have also been implicated in altering chemotherapeutic efficacy, specifically DOX has been shown to stimulate proliferation of myeloid derived suppressor cells and subdue antitumor activity (12). In the TME, macrophages have been shown to decrease the localized response of DOX by filtering away the drug from cancer cells yet they can also potentiate the anti-cancer effects through activation and release of active DOX into the cancer cells (13). This stromal response can be further exploited as a potential stromal-modulating treatment in combination with alternative anticancer therapies. In fact, the immunomodulatory effect of DOX treatment increases the efficacy of adoptive T-cell transfer therapy in some breast cancer subtypes, when administered before immunotherapy (14). CD86 expression has also been shown to be upregulated in B cells that then enhance CD4+ T cell anti-cancer activity (15).

IL-17 is a proinflammatory cytokine that has previously been linked to several tumor behaviors. It is primarily secreted by T helper 17 (Th17) cells and innate lymphocytes [γδ T cells, natural killer (NK) cells, and innate lymphoid cells] (16) yet the receptor it binds to is broadly expressed across a variety of immune and non-immune cell types (17, 18). In multiple types of cancer, IL-17 expression has been correlated with tumor progression and is associated with poor prognosis in breast cancer (19) however, previous studies have also identified both pro- and anti- tumor function in the TME. Chronic IL-17 expression leads to a pro-tumor microenvironment through modulation of stromal cell types that increase angiogenesis and antitumor immunity (19). Yet upon exposure to chemotherapeutics, the presence of IL-17 appears to encourage anti-tumor effects (20). Furthermore, decreased efficacy of anthracyclines and oxaliplatin was observed in mouse fibrosarcoma allografts in IL-17A knockout mice, a phenotype that was rescued upon adoptive transfer of γδ T cells with normal IL-17 production (21). In breast adenocarcinomas a similar correlation was observed in which optimal DOX therapeutic efficacy was found to require IL-17, in mice (22). Correlation with increased efficacy was also observed in gastric cancer patients (20). IL-17’s proinflammatory role in combination with chemotherapy has been implied to aide in recruitment of antitumor cytotoxic T cells, however, specific mechanisms of action remain to be elucidated (21).

To advance our understanding of emergent drug resistance and develop novel approaches for overcoming it, this study examined the altered stromal composition and behavior of cells residing in the TNBC TME to determine molecular and cellular drivers of DOX treatment response. To recapitulate clinical relevance in an immune competent animal model, 4T1 murine cancer cells were injected into the mammary fat pad of BALB/c mice to generate syngeneic tumors. These tumor bearing mice exhibit a range of responses to a 7-day dosing regimen with DOX ranging from suppressed to unaltered tumor growth. Further examination of the TME identified a significant increase in the absolute number of T cells in chemo-responsive, DOX-sensitive tumors. T cell subtypes with increased cytokine secretion and decreased exhaustion were found to be more prevalent in DOX -sensitive relative to -resistant tumors. Specifically, we found a significant increase in IL-17A+ CD8 T cells in DOX-sensitive tumors and these lymphocytes may exert effects on several TME cell types. *In vitro* analysis of IL-17’s activity directly on cancer cells showed anti-tumor effects by increasing tumor responsiveness to DOX upon co-administration with recombinant IL-17A. In response to IL-17A and DOX, 4T1 cells increased cytokine signaling and cell cycle dysfunction while decreasing DOX-induced stimulation of immune response genes which may contribute to T cell exhaustion. This study demonstrates the effects of the TME on doxorubicin response in TNBC and identifies IL17+ T cells as a potential prognostic marker or therapeutic target for improved chemotherapeutic efficacy.

## Materials and Methods

### Cell Culturing and Allograft Generation

4T1-Thy1.1 cell line, referred to as 4T1 throughout the manuscript, (a gift from Dr. Julian Lum) (23) was used in all allograft and *in vitro* experiments. 4T1 cells were cultured in RPMI Medium 1640 containing 10% FBS with 100,000 U/L of penicillin and 100 mg/L of streptomycin at 37°C with 5% CO_2_. Female mice (8-10 weeks old) NOD.*Cg-Prkdc^scid^Il2rg^tm1Wjl^*/SzJ (NSG) or BALB/c mice (Jackson Laboratories, Bar Harbor, ME, USA) were injected with 1×10^5^ 4T1 cells into the mammary fat pad, as previously described (24). Tumors were established to 70–140mm^3^ size range prior to Doxorubicin (Sigma Aldrich, St. Louis, MO, USA) (DOX) administration. DOX was introduced intravenously (IV) by tail vein injection at 5 mg/kg for 3 treatments, 3-days apart. All mice were weighed, and tumor sizes were determined using manual palpation and caliper measurements prior and during chemotherapeutic treatment, up to terminal endpoints. Tumor volume calculations were determined using the formula: volume=½ x (length x width^2^). Moribund behavior was evaluated regularly throughout the tumor bearing period. All animal experimental procedures were completed under an approved Institutional Animal Care and Use Committee (IACUC) protocol at Lawrence Livermore National Laboratory (LLNL) and conforming to the National Institute of Health (NIH) guide for the care and use of laboratory animals.

### Tumor Single Cell Isolation and Enrichment

Single-cell suspensions of tumor cells were prepared as previously described using a combination of physical dissociation and enzymatic digest (24). Red blood cell lysis was performed using ACK Lysing Buffer (Gibco, Waltham, MA, USA) per manufacturer’s recommendation. Digests were filtered through a 100µm cell strainer prior to debris removal (Miltenyi Biotec, Bergisch Gladbach, Germany; Cat # 130-109-398). Cells were resuspended in BD FACS Pre-Sort Buffer (BD, Franklin Lakes, NJ, USA; Cat # 563503) for further fluorescently activated cell sorting (FACS) analyses, or in washed 2X in sterile PBS+0.04% non-acetylated BSA for single cell sequencing.

### Single-Cell Sequencing and Data Analysis

Immune and cancer cell depletions were performed for T cell specific reactions using Pan T Cell Isolation Kit II, mouse (Miltenyi Biotec, Bergisch Gladbach, Germany; Cat# 130-095-130) in combination with CD90.1 MicroBeads (Miltenyi Biotec, Bergisch Gladbach, Germany; Cat# 130-121-273) per manufacturers protocols prior to were cell depletion using LS columns (Miltenyi Biotec, Bergisch Gladbach, Germany; Cat# 130-042-401). Sequenced T cell populations were derived from 3 independent syngeneic 4T1 tumors in BALB/c mice as previously described; independent experiments (N=3) were pooled into a single sequencing population. Cell pellets were resuspended in PBS with 0.04% non-acetylated BSA prior to single-cell sequencing preparation using Chromium Single-cell 3′ GEM, Library & Gel Bead Kit v3 (10× Genomics, Pleasanton CA, USA Cat # 1000075) on a 10× Genomics Chromium Controller following manufacturers protocol.

Sequencing data was demultiplexed, quality controlled, and analyzed using Cell Ranger (10× Genomics, Pleasanton CA, USA) and Seurat (25). The Cell Ranger Single-Cell Software Suite was used to perform sample demultiplexing, barcode processing, and single-cell 3′ gene counting. Samples were first demultiplexed and then aligned to the mouse genome (mm10) using “cellranger mkfastq” with default parameters. Unique molecular identifier counts were generated using “cellranger count”. Further analysis was performed using Seurat V4 (26).

### Flow Cytometry

Cell preparations for tumor cells were derived from cell suspensions as previously described in the single cell isolation section then resuspended in FACS buffer (PBS with 2% FBS). Bone marrow preparations were performed from isolated femurs. Femoral epiphyses were removed from the bone then the marrow cavity was flushed with a 28-gauge needles with 2 mL of PBS. The bone marrow derived cell suspensions were centrifuged at 500g for 10 minutes followed by red blood cell lysis and resuspension in FACS buffer prior to cytometric analysis. Splenocytes were prepared from isolated spleens that were forced through a 40-µm cell strainer. Cells were washed with PBS and pelleted by centrifugation at 500g for 10 min. Red blood cell lysis was performed using ACK lysis buffer and resuspended in FACS buffer prior to cytometric analysis.

Cell suspensions were stained with the following antibodies for 30 minutes on ice prior: BioLegend (San Diego, CA, USA): CD45 (1:100; Cat# 103116, 157613), CD3ϵ (1:50; Cat# 100312), CD4 (1:100; Cat# 100414, 100406), CD8b (1:100, Cat# 126622, 126609), CD279 (PD-1) (1:100, Cat# 135213), IL-17A (1:100, Cat# 506922), TCR γ/δ (1:100, Cat# 118107; Miltenyi Biotec (Miltenyi Biotec, Bergisch Gladbach, Germany): CD90.1 (1:10, Cat# 130-102-637). Viability dyes Zombie Violet™ Fixable Viability Kit (BioLegend, San Diego, CA, USA), Zombie Aqua™ Fixable Viability Kit (BioLegend, San Diego, CA, USA), or eBioscience Fixable Viability Dye eFluor506 (Invitrogen, Waltham, MA, USA) were utilized to discriminate live/dead cells. Following staining, cell populations were washed 2 times with FACS buffer prior to 20-minute fixation using Cytofix Buffer (BD Biosciences, San Jose, CA; USA) then resuspension in FACS buffer for analysis.

For cytokine detection, tumor derived cells were cultured in RPMI supplemented with 10% FBS, 50ng/ml PMA Sigma, St. Louis, MO, USA, Cat# P-8139, 1µg/ml Ionomycin (Sigma, St. Louis, MO, USA, Cat# I-0634), and GolgiPlug (BD Biosciences, San Jose, CA; USA) at 37°C with 5% CO_2_ for 4 hours followed by extracellular staining then fixation as described previously. Intracellular staining was accomplished using Intracellular Staining Permeabilization Wash Buffer (BioLegend, San Diego, CA, USA) for permeabilization, staining buffer, and subsequent washes followed by resuspension in FACS buffer for downstream analysis. Flow cytometric analysis was performed using FACSMelody (BD Biosciences, San Jose, CA; USA), BD LSR II (BD Biosciences, San Jose, CA; USA), FACSAria Fusion (BD Biosciences, San Jose, CA; USA) instrument.

### Immunofluorescent Staining

Tumor samples were collected at terminal time points, snap frozen in liquid nitrogen and stored at -80°C until processing. Frozen tumors were embedded in optimal cutting temperature (OCT) compound (Fisher Healthcare, Waltham, MA, USA) and sectioned at 10µm slices. Tumor sections were placed onto Superfrost Plus microscope slides (Fisher Scientific, Waltham, MA, USA) and stored at -80°C until utilized. To stain sections, slides were warmed to room temperature and then were immersed in PBS with 4% formaldehyde for 15 minutes. Slides were then immersed in PBS with 0.1% Tween 20 and 10% goat serum for one hour at room temperature. Primary antibody IL-17-A [Abcam, Cambridge, MA, USA, ab79056, (1:250)] was incubated overnight at 4°C. Sample slides were then incubated at room temperature for 1 hour with the secondary antibody goat anti-rabbit [Thermo Fisher Scientific, Waltham, MA, USA; A-11037 (1:1000)]. Negative control slides were incubated with secondary antibody only. Stained slides were mounted with Prolong Gold with DAPI (Molecular Probes, Eugene, OR, USA). Slides were imaged using a Leica DM5000 microscope. ImagePro Plus V7.0 Software and a QIClick CCD camera (QImaging, Surrey, BC, Canada) were used for imaging and photo editing.

### Western Blot

Tumor samples lysed in RadioImmunoPrecipitation Assay (RIPA) buffer followed by centrifuging at 14,000g for 5 min. The supernatants were collected and analyzed using the Jess automated Western blotting system (ProteinSimple, San Jose, CA, USA). Jess reagents (biotinylated molecular weight marker, streptavidin-HRP fluorescent standards, sample buffer, DTT, stacking matrix, separation matrix, running buffer, wash buffer, matrix removal buffer, fluorescent labeled secondary antibodies, antibody diluent, and capillaries) were purchased from the manufacturer and used according to the manufacturer’s standard protocol. Antibodies were diluted with ProteinSimple antibody diluent at the following dilutions: anti IL-17-A (1:50, Abcam, Cambridge, MA, USA, ab79056, and GAPDH (1:100, Licor, Cat# 926-42216). Target protein concentration is quantitated using Compass for SW 4.0 software (https://www.proteinsimple.com/compass/downloads/). The expression of each target protein is normalized to the expression of GAPDH.

### *Ex Vivo* Culturing and *In Vitro* Doxorubicin and IL-17A Administration

Single cell suspensions from syngeneic tumors were performed as described in the Single-cell sequencing section. CD90.1 MicroBeads, mouse and rat (Miltenyi Biotec, Bergisch Gladbach, Germany; Cat# 130-121-273) were used for cell isolation using LS columns. Subsequent elution of cells of the cell isolation columns were then cultured overnight. Cancer cell populations from 3 syngeneic tumors derived from unique mice were utilized for *ex vivo* 4T1 DOX response experiments for each tumor phenotype.

Doxorubicin (200ng/ml, Sigma Aldrich, St. Louis, MO, USA) and/or Recombinant Mouse IL-17A Protein (25ng/mL, R&D Systems, Minneapolis, MN, USA) were administered for 48 hours prior to cell quantitation using CellTiterGlo 2.0 (Promega, Madison, WI) according to manufacturer’s protocols then read for luminescent signal on a Modulus II Microplate Multimode Reader. Raw reads were first background (media without cells) subtracted then normalized to untreated cells for cell quantitation. 3 independent experiments were performed for *in vitro* DOX viability assays.

### Bulk RNA Sequencing and Analysis

4T1 cells were cultured to 25% confluency in a 12 well culture plate. Total RNA was isolated using RNeasy mini spin columns (Qiagen). Sequencing library preparation was performed using QuantSeq 3’ mRNA-Seq Library Prep Kit FWD for Illumina (Vienna, Austria; Cat# 015.96) according to manufacturer’s protocols. and single end 75 base pair sequencing was performed using an Illumina NextSeq 500. Sequencing data quality was checked using FastQC software (https://www.bioinformatics.babraham.ac.uk/projects/fastqc/). Reads were mapped to the mouse genome (mm10) using STAR (version 2.6) (27) and read counts per gene were determined using “featureCounts” from Rsubread package (version 1.30.5; https://bioconductor.org/packages/release/bioc/html/Rsubread.html). Subsequently, data was normalized using TMM normalization (28) and differentially expressed genes were identified using voom and limma (29). A gene was significantly differentially expressed when its false discovery rate adjusted *p*-value was <0.05 and fold change was >2. Gene set enrichment analysis was performed using GenePattern with Reactome pathway ontologies (30, 31).

### Statistical Analyses

Statistical analyses were performed using GraphPad Prism. Data is presented from at least three biological replicates. One-way ANOVA and *post-hoc* Tukey’s Test or Student’s t-test were used to assess statistically significant differences of mean expression values. Results were considered statistically significant for *p* values < 0.05. IC50 curves were generated using a nonlinear regression curve fit analysis.

## Results

### Stromal Complexity Correlates With TNBC Responsiveness to Doxorubicin Treatment, in Mouse Allografts

**Figure 1:**
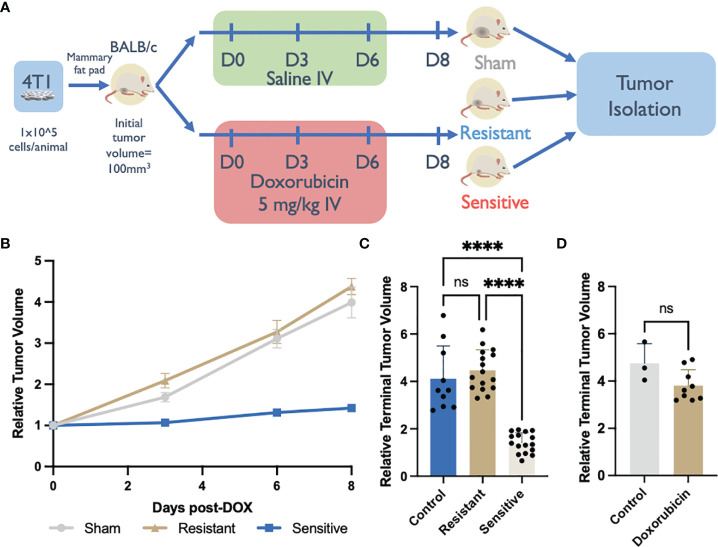
Syngeneic 4T1 tumors differentially respond to doxorubicin (DOX) treatment. **(A)** DOX treated BALB/c 4T1 mammary fat pad tumor experimental design. **(B)** Syngeneic tumor growth rates of tumors in response to DOX. Relative tumor volume was normalized to initial volume and calculated from caliper measurements throughout the experiment. **(C)** Relative terminal tumor volume at Day 8 post DOX initiation from saline-treated controls or DOX treated tumors in immunocompetent BALB/c mice binned into resistant or sensitive populations (n=10-16). **(D)** Relative terminal tumor volume at Day 8 post DOX initiation from saline-treated controls or DOX treated tumors in immunodeficient NSG mice (n=3-9). ns: non-significant *p* > 0.05, ****p < 0.0001.

To recapitulate the TNBC microenvironment, syngeneic allografts by injection of 4T1 cells were generated by delivery into the mammary fat pad of immune competent BALB/c mice. Upon reaching a volume of ~100 mm^3^, tumor bearing mice were administered 3 doses of DOX treatment over 7 days. Two days following the final dose, tumors were measured and harvested for downstream analysis (**Figure 1A**).

In control, saline-treated mice (n=10), tumors reached a volume ~400% of the initial recorded size, on day 8 post-treatment initiation (**Figures 1B, C**). However, in the DOX-treated group, tumor growth was either inhibited (sensitive tumors; n=16) or was unaffected (resistant tumors; n=16) (**Figures 1B, C**). Sensitive tumors showed a consistent inhibition of growth throughout the chemotherapeutic treatment yielding tumors that were significantly smaller (142% terminal tumor volume) than control tumors. Conversely the growth rate of resistant tumors was comparable, and not significantly different than the growth rate of saline-treated tumors (438% terminal tumor volume) (**Figures 1B, C**). This polarized response to DOX treatment was specific to the immune competent BALB/c allograft host strain and was not recapitulated in the immunocompromised (NSG) host strain that lacks mature B, natural killer (NK), and T cells in addition to having functionally defective macrophages and dendritic cells (32, 33). All NSG-DOX dosed tumors exhibited a drug response that resembled drug resistant DOX response in BALB/c mice; no statistically significant differences in growth rates were observed between DOX- and saline-treated control tumors (**Figure 1D**). The absence of sensitivity to DOX in mice lacking functional immune cells suggests that DOX treatment efficacy is dependent on immune subtypes present in the tumor microenvironment.

### Increased Abundance of Tumor Infiltrating T-Cells in DOX Sensitive Tumors

**Figure 2:**
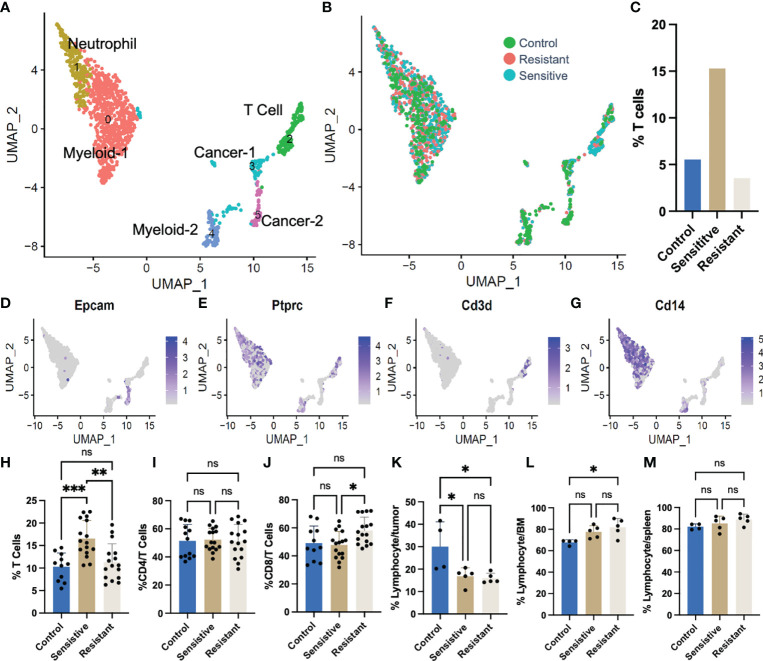
Single cell RNA sequencing (scRNA-Seq) of tumors following DOX administration. **(A)** UMAP plot representing all tumor cells following DOX treatment. **(B)** Colors depict cells derived from tumors of different treatment/response. **(C)** T cell [Ptprc (CD45)+ CD3d+] relative abundance derived from single cell RNAseq data. **(D–G)** Feature plots of gene markers used in identifying cell clusters. **(H)** Flow cytometric quantification of T cell (CD3ϵ+) abundance in syngeneic 4T1 tumors. **(I)** Flow cytometric quantification of cytotoxic T cell (CD8+CD3ϵ+) abundance in syngeneic 4T1 tumors. **(J)** Flow cytometric quantification of immune [Ptprc (CD45)+] abundance in syngeneic 4T1 tumors. **(K)** Flow cytometric quantification of immune [Ptprc (CD45)+] abundance in bone marrow of tumor bearing mice. **(L)** Flow cytometric quantification of immune [Ptprc (CD45)+] abundance in spleens of tumor bearing mice. **(M)** Flow cytometric quantification of immune [Ptprc (CD45)+] abundance in spleens of tumor bearing mice. ns: non-significant *p* > 0.05; *p ≤ 0.05; **p ≤ 0.01; ***p ≤ 0.001.

Single cell RNA sequencing (scRNA-Seq) was performed on 1,325 tumor derived cells from representative tumors from each DOX responsive category (untreated controls, drug sensitive and drug resistant) to further investigate alterations in the abundance of stromal cell subtypes. Unsupervised hierarchical clustering of cell types based on transcriptional profiles identified cancer, T cell, neutrophil, and myeloid populations as represented in a UMAP projection (**Figures 2A, D–G**). The percentage of T cells was found to be increased in sensitive tumors (15.3%) relative to saline-treated controls (5.54%) and drug-resistant tumors (3.6%) (**Figures 2B, C**). ScRNA-Seq data was further validated using flow cytometric analysis (**Figures 2H–M**). Sensitive tumors were comprised of 16.6% T cells in the primary tumors which were significantly increased relative to saline-treated control tumors (10.3%) or tumors unresponsive to treatment (11.3%) (**Figure 2H**). There was no significant change in CD4 T cell abundance observed across conditions (**Figure 2I**), yet an increase in the number of CD8+ T cells within the tumor T cell population of drug resistant primary tumors, compared to the drug-sensitive tumors (58.1%, 47.8% respectively) was observed (**Figure 2J**).

Responsiveness to DOX did not increase tumor infiltration of other immune subtypes. In fact, DOX treatment decreased the absolute abundance of tumor infiltrating lymphocytes, in drug-treated tumors, regardless of tumor response, and significant increases in total number of CD45+ immune cells were only observed in saline-treated tumors (30.0% of tumor cells) compared to both sensitive and resistant tumors (16.85%, 15.92%, respectively) (**Figure 2K**). Furthermore, the increase in lymphocyte abundance was specific to the TME as both the bone marrow (**Figure 2L**) and the spleen (**Figure 2M**) were found to be largely unchanged with a slight increase in abundance only observed in the bone marrow of mice bearing resistant tumors compared to control tumor bearing mice (**Figure 2L**).

### Altered T Cell Composition in Chemoresistant Tumors

**Figure 3:**
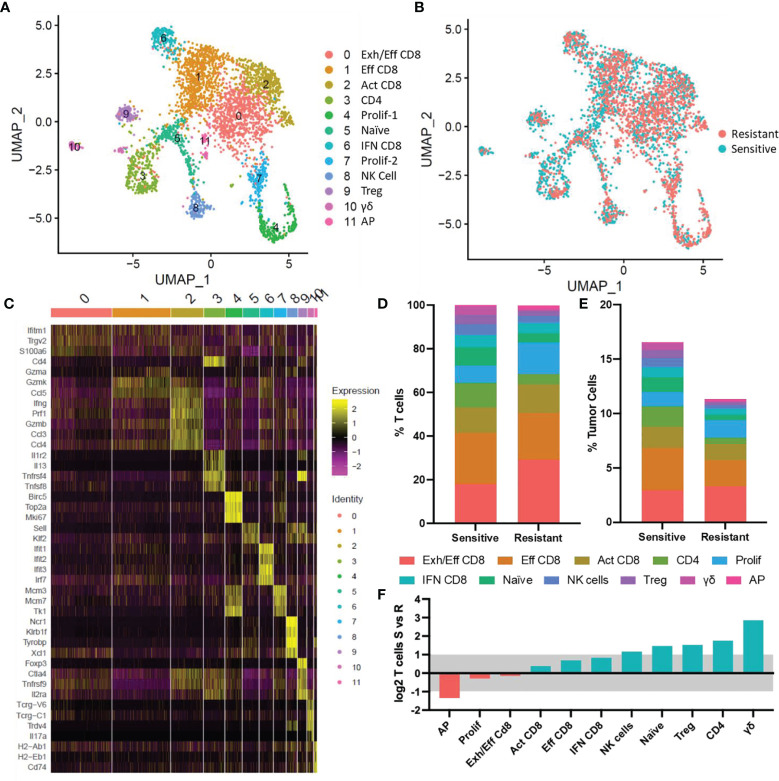
ScRNA-Seq of tumor infiltrating T cells. **(A)** UMAP plot of tumor infiltrated T cells. 12 populations identified *via* transcriptional profiles are denoted by color. **(B)** UMAP plot with colors denoting tumor response to DOX. **(C)** Heat map showing relative gene expression for genes of interest per cell in each cluster. **(D)** Distribution of T cell subtypes in sensitive and resistant tumors. **(E)** Normalized T cell subtype abundance in sensitive or resistant tumors relative to all tumor cells. **(F)** Shifts in abundance of tumor infiltrating T cells in sensitive and resistant tumors. Blue bars denote populations with increased abundance in sensitive tumors, red bars denote populations increased in resistant tumors. Bars outside the grey shaded region represent significant (>2-fold) shifts in cell type abundance. Exh/Eff CD8, exhausted effector CD8 T cells; Eff CD8, effector CD8 T cells; Act CD8, activated CD8 T cells; CD4, CD4 T cells; Prolif-1, proliferating T cells-1; Naïve, naïve T cells; IFN CD8, interferon stimulated CD8 T cells; Prolif-2, proliferating T cells-2; NK Cell, natural killer cells; Treg, T regulatory cells; γδ, *γδ* T cells; AP, antigen presenting T cells.

The function and behavior of these infiltrated lymphocytes was further examined using targeted scRNA-Seq focused on tumor residing lymphocyte populations. 3,495 *Ptprc* (CD45) and *CD3e* expressing cells were identified from pooled resistant and sensitive tumors (**SFigure 1**). *In silico* dimensional reduction of the transcriptional profiles of each cell produced 12 clusters of cells denoting different T-cell subtypes, as visualized in a UMAP plot (**Figure 3A** and **SFigure 1**). CD8^+^ T cells were more abundant in resistant (84.8% of T cells) than in sensitive tumors (67.6%) and conversely CD4^+^ T cells were more abundant in sensitive (15.5%) than in resistant tumors (7.0%). No single population was uniquely present in either sensitive or resistant tumors however, biases toward specific subtypes were identified correlating with chemosensitivity status (**Figure 3B**). Cluster identification was performed using published gene markers of T cell subtypes (**Figure 3C**) (34, 35) and distribution of T cell abundance was quantitated based on cell quantity per cluster (**Figure 3D**). In order to identify modulation of T cell subtypes in the TME, normalization of relative T cell abundance was performed based on overall T cell abundance observed in previously described flow cytometric analysis (**Figures 2H**, **3E**), and shifting populations were compared to identify modulating populations of interest (**Figure 2F**).

**Figure 4:**
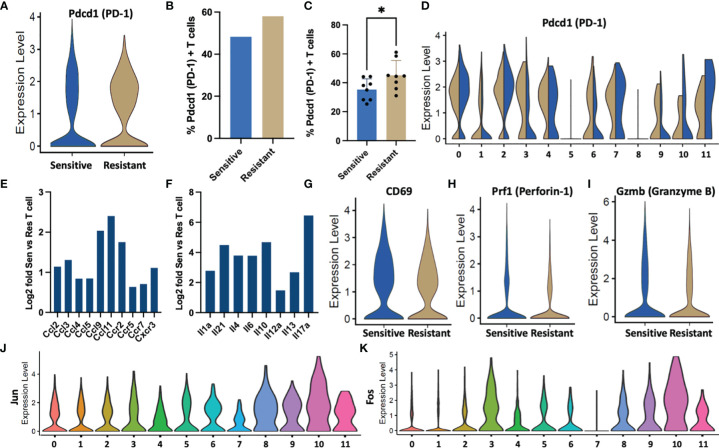
T cell behavior in response to DOX sensitivity. **(A)** Violin plots denoting expression of *Pdcd1* per T cell in sensitive or resistant tumors. **(B)** Percentage of tumor residing T cells expressing *Pdcd1* as quantified in scRNA-Seq data. **(C)** PD-1+ T cells quantified from tumor infiltrated T cells following DOX treatment using flow cytometric analysis. **(D)** ScRNA-Seq expression levels of *Pdcd1* by cluster ID depicted as violin plots. **(E)** Fold upregulation of chemokine and **(F)** cytokine genes in sensitive vs resistant T cell populations inferred from scRNA-Seq data. **(G–I)** Activation gene markers (CD69) and cytotoxic genes (*Prf1*, *GzmB)* associated with T cell activity expression in sensitive or resistant tumors depicted as violin plots. **(J, K)** Dimeric transcription factors comprising the AP-1 transcription factor indicative of T cell activation expression segregated by cell types identified in single cell transcriptomic data. *p ≤ 0.05.

Antigen-presenting (AP) T cells (expressing *H2-Ab1, H2-Eb1, CD74)* were the only lymphocyte subtype found to be significantly increased in resistant tumors however, this population represented the least abundant cell type observed (Resistant: 0.2%; Sensitive 0.1% of lymphocytes). Minor shifts in T cell subtype abundance were observed in 6 clusters (**Figure 3F**). Three effector CD8^+^ populations expressing classical cytotoxic T cell markers (*CD8b*, *Ifng*, *Gzm* genes, *Prf1*), were the dominant subtype of cells in the TME comprising 63.7% and 53.3% of infiltrating T cells in resistant and sensitive tumors, respectively. Minor increases in effector (Eff CD8^+^) or activated effector (Act CD8^+^) T cells expressing increased cytotoxic genes were observed in sensitive tumors, while exhausted effector CD8 (Exh/Eff CD8^+^) T cells characterized by decreased cytotoxic genes and increased *Pdcd1* (PD-1) expression (**Figure 4A**) relative to effector CD8^+^ T cells were also found to be relatively unchanged in relation to all cells in the TME. Furthermore, interferon stimulated CD8 T cells (IFN CD8^+^) characterized by high levels of Jak/Stat signaling pathway activation genes (i.e., *Stat1*, *Stat2*) and interferon response genes (*Ifit1*, *Ifit2*, *Ifit3*) were 77.8% more abundant in sensitive compared to resistant tumors. Proliferating T cell populations (*Prolif-1, Prolif-2*) were observed in two clusters and characterized by expression of Mki67 (*Ki-67*), mini-chromosome maintenance genes (*Mcm3, Mcm7*), and cell cycle progression genes (*Top2a*, *Ccna2*). This population was found to be only slightly elevated (1.2%, 1.3% respectively) in abundance in resistant tumors.

Upon comparison of infiltrated T cells from sensitive tumors and resistant tumors, four T cell subtypes in addition to Natural Killer cells were observed to be significantly increased in sensitive tumors (**Figure 3F**). Naïve T cells expressing genes associated with immature T cells and quiescence (*Klf2*, *Klf3*, *Sell* (CD62L), *Lef1)* (34, 36) were found to be increased to 1.3% of cells in the sensitive TME relative to 0.5% in the resistant TME. Sensitive tumors were also found to possess more CD4^+^ T helper cells, regulatory T cells (Tregs), and γδ T cells. CD4^+^ T cells expressing *CD4* and several cytokines (*IL4*, *IL5*, *IL13*) increased to 1.8% of all cells in sensitive tumors compared to 0.5% in resistant tumors. Tregs, denoted by increased expression of *Foxp3*, *Ctla4*, and *Tnfrsf4* (CD134), represented 4.5% of sensitive tumor T cells compared to half as many (2.3%) in the resistant populations. γδ T cells expressing gamma and delta T cell receptors (*Tcrg-C1* and *Trdv4*) in addition to *TNFa* and *IL-17A* were a minor population of T cells yet accounted for a significant, 6-fold increase in abundance in sensitive tumors (Resistant: 0.1%; Sensitive 0.6% of total tumor residing cells).

### T Cells Populations From DOX Sensitive Tumors Are Less Exhausted, Release Higher Levels of Cytokines and Show Increased Activation

Because the increased number of T cell subsets in chemo-sensitive tumors may not necessarily translate into an increase in activation, further analysis of the single cell transcriptomic data was performed to examine critical T-cell functions in the TME. Chronic antigen stimulation is a well-documented phenomenon in tumor activation resulting in an exhausted phenotype characteristic of decreased effector function, proliferation, and cytokine production and can be identified through over expression of *Pdcd1* (37, 38). T cells from DOX resistant tumors expressed higher levels of *Pdcd1* per cell with increased median normalized expression (**Figure 4A**). Additionally, a 10% increase in the frequency of *Pdcd1^+^* exhausted T cells was observed in resistant tumors (Resistant: 58%, Sensitive: 48%) (**Figure 4B**). Cytometric analysis of tumor infiltrated T cells identified fewer PD-1^+^ T cells compared to the single-cell transcriptional data however, a significant increase in abundance was confirmed in resistant tumors (**Figure 4C**). Upon segregation of lymphocytes by subtype, elevated exhaustion was observed in exhausted effector CD8 (Cluster 0), activated effector CD8 (Cluster 2), proliferating cell populations (Cluster 4,7), and antigen presentation (Cluster 11) (**Figure 4D**). Furthermore, DOX resistant populations with increased exhaustion relative to sensitive tumors were observed in CD4 (Cluster 3), interferon response CD8 (Cluster 6), and Treg (Cluster 9) populations.

These differentially exhausted subpopulations in addition to *γδ T cells* (Cluster 10) were found to be responsible for increased cytokine and chemokine genes and these T cell subtypes were found to be increased in abundance in sensitive tumors (**Figures 4E, F**). Furthermore, T cells from sensitive tumors exhibited an increased mean and median expression of *CD69*, an activation marker (39) (**Figure 4G**). Effector proteins associated with cytotoxic activity, *Gzmb* and *Prf1*, were also found to be increased in the sensitive T cell population (**Figures 4H, I**). Additionally, expression levels of *Jun* and *Fos*, transcription factors critical to T cell activation, were elevated in the cytokine producing CD4 and *γδ* populations potentially indicating a critical role in tumor response (**Figures 4J, K**) (40).

### Higher Numbers of CD8 γδ IL-17+ T Cells in the Tumor Microenvironment of DOX Sensitive Tumors

**Figure 5:**
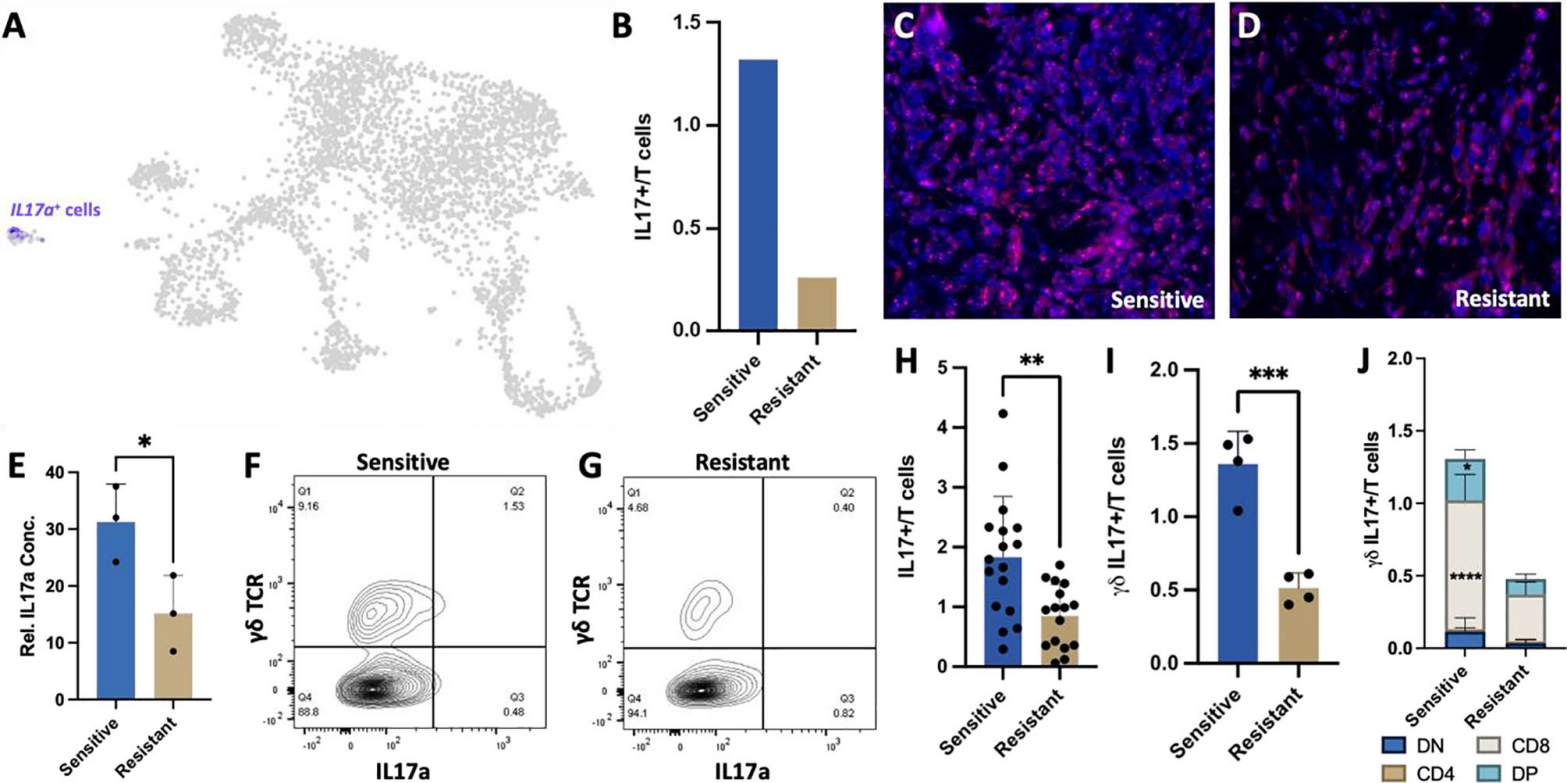
Increased γδ IL-17+ T cells in the DOX sensitive tumor microenvironment. **(A)** UMAP projection of 4T1 syngeneic tumor cells identifying that *IL17a* expression is restricted to γδ T cells. **(B)** Ratio of IL17a+ cells in sensitive and resistant tumors extrapolated from scRNA-Seq data. **(C, D)** Representative immunohistochemistry images of *IL17A* expression in 4T1 tumor sections from sensitive and resistant tumors following DOX therapy. **(E)** Protein abundance quantification in tumors normalized to GAPDH expression (n=3). **(F, G)** Representative flow cytometry plots identifying γδ IL-17+ T cells in T cell populations. **(H)** Quantitation of IL17A+ T cells from DOX sensitive and resistant tumors. **(I)** Quantitation of γδ IL17A+ T cells from DOX sensitive and resistant tumors (n=4). **(J)** Distribution of γδ IL17A+ T cells identifying expanded CD8 and double positive (CD4+CD8+) cells in sensitive tumors (n=4). *p ≤ 0.05, **p ≤ 0.01, ***p ≤ 0.001, ****p ≤ 0.0001.

Due to the increased cytokine production and activation of cytokine secreting T cell populations in tumors responsive to chemotherapeutic treatment, we next examined the impact of cytokines on cancer cells. Specifically, *IL-17A* expressing T cells were found to be more abundant in DOX- sensitive than in resistant tumors. IL-17A is a proinflammatory cytokine with known pro and anti-tumor effects (41–44). Further transcriptional characterization of *IL-17A* expressing T cells revealed that they are not derived from αβ CD4 T cells but from γδ IL-17+ T cells based on expression of delta and gamma T cell receptor genes (*Trdv4, Trdc, Tcrg-V6, Tcrg-C1)* (**Figures 3C**, **5A**). This T cell subpopulation comprised 1.32% of all T cells in sensitive tumors and 0.26% of all T cells in resistant tumors (**Figure 5B**). While this population only represents a minor portion of the tumor infiltrating lymphocytes, scRNA-Seq on syngeneic 4T1 tumors revealed that *IL-17A* is uniquely expressed and secreted from these specialized T cells into the TME yet numerous cell types in the TME can bind this cytokine by expressing its receptor (*IL-17ra*) underlying the potential impact of IL-17A in the TME (**SFigure 2**). Furthermore, despite the low number of these T cells, histological analysis of representative sensitive and resistant tumors confirmed detectable levels of IL-17A protein throughout the tumor with increased relative abundance in DOX sensitive tumors (**Figures 5C, D**). Protein quantification by western blot further confirmed 257% higher IL-17A levels in homogenized tumor samples of sensitive tumors compared to resistant tumors (**Figure 5E**).

Flow cytometry analysis of tumor infiltrating T cells was consistent with the scRNA-Seq data, confirming a significantly higher number of *IL-17A* expressing T cells in sensitive tumors than resistant tumors (1.83%, 0.85% respectively, *p-value*=0.0016) (**Figures 5F–H**) with γδ T cells constituting the majority of the *IL-17A* expressing cells (1.36%, 0.51% respectively) regardless of tumor chemotherapeutic response (**Figure 5I**). Interestingly, CD8^+^ IL17A^+^ T cells were identified as the most abundant and significantly increased population in sensitive tumors (*p*<0.0001). CD4^+^CD8^+^IL17A^+^ double positive T cells population were also found to be significantly increased in chemo-sensitive tumors relative to resistant (*p*=0.0218) while double negative and CD4 T cells were not found to be significantly altered in abundance (**Figure 5J**).

### IL-17A Increases DOX Sensitivity of 4T1 Cells

**Figure 6:**
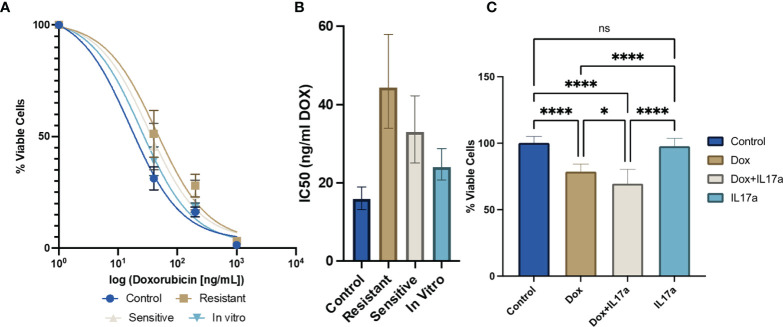
IL-17A co-administration with DOX directly affects chemotherapeutic efficacy in cancer cells. **(A)** *Ex vivo* DOX sensitivity from 4T1-Thy1.1 cells isolated from primary tumors or from *in vitro* culture (n=12 from 3 independent tumors). **(B)** IC50 values extrapolated from dose response curves with error bars represent 95% CI. **(C)** Relative viability of 4T1-Thy1.1 cells cultured in the presence of DOX and/or IL-17A for 48 hours (n=9-11). ns: non-significant *p* > 0.05; *p ≤ 0.05; ****p ≤ 0.0001.

Cancer cells were found to express the *IL-17* receptor (**SFigure 2**), and therefore to be potential targets of IL-17A signaling. While cancer resistance evolution driven by genomic mutations has been thoroughly reported as a long-term mechanism for waning efficacy of chemotherapy, the hypothesis that transient molecular changes in the stroma drive differential responses to treatment has been minimally explored. Here we examined the *ex vivo* cytotoxicity of 4T1 cells isolated from DOX-sensitive and -resistant tumors to examine intrinsic alterations in chemotherapeutic response conferred from the *in vivo* manipulations. IC50 curves were generated for 4T1 cells derived from the parental cell line, saline-treated control, DOX sensitive, or DOX resistant tumors by administering a range of drug dosages over 48 hours in culture. Both *in vivo* DOX treated 4T1 exhibited a higher tolerance to the drug treatment (4T1 from Resistant Tumors: 44.32 ng/mL; 4T1 from Sensitive Tumors: 32.67 ng/mL) than 4T1 from saline-treated tumors (15.89 ng/mL) or mouse-naïve 4T1 cells (24.50 ng/mL) (**Figures 6A, B**). *In vivo* DOX treatment, regardless of drug response, increased the tolerance of 4T1 cancer cells relative to the parental control cell line. Next, we determined whether IL-17A directly contributes to DOX chemosensitivity. The potency of DOX treatment was significantly enhanced when IL-17A was co-administered to the culture media (**Figure 6C**). This data suggests that the tumor behavior is only partially driven by cell autonomous response of cancer cells to tolerate the administered drug and in part also by IL-17A levels in the TME.

### IL-17A Mitigates PD-L1, Induces Cytokine Signaling, Cell Cycle Dysregulation, and Mitigates Interferon Activation in TNBC Cells

**Figure 7:**
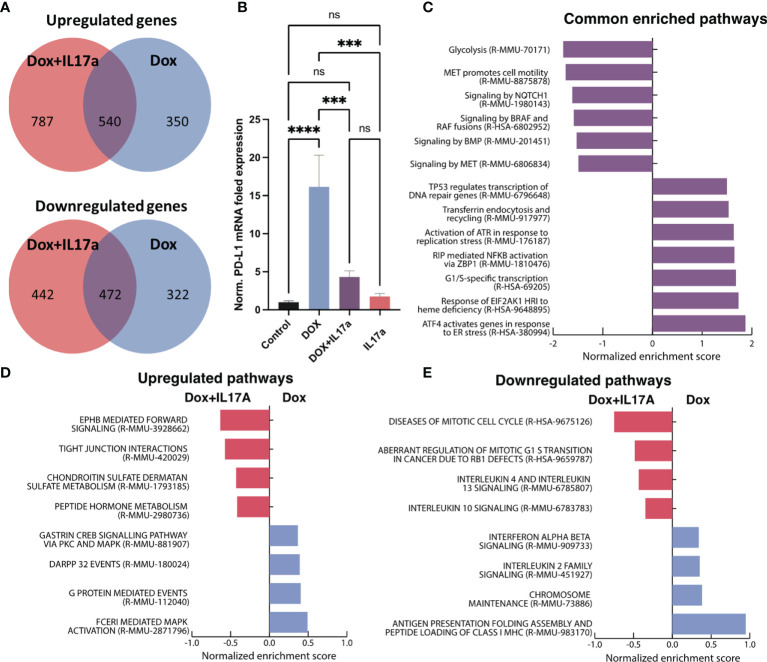
Transcriptomic analysis of 4T1 cells in response to DOX and IL-17A. **(A)** Venn diagrams depicting overlapping differentially expressed genes from 4T1 cells exposed to DOX +/- IL-17A for 48 hours. **(B)** Normalized gene expression of CD274 (PD-L1) across *in vitro* conditions. **(C)** GSEA analysis of enriched Reactome pathways in common to DOX treated cells regardless of IL-17A. **(D)** Upregulated pathways with differential enrichment. **(E)** Downregulated pathways with differential enrichment (n=3-4). ns non-significant *p* > 0.05; ***p ≤ 0.001; ****p ≤ 0.0001.

Bulk RNA sequencing was performed on 4T1 cells exposed to DOX or DOX co-administered with IL-17A. DOX alone up-regulated 890 genes and down-regulated 794 genes while the DOX-IL-17A co-treatment yielded 1327 up-regulated and 914 down-regulated genes relative to untreated controls. Five hundred and forty up-regulated and 472 down-regulated genes were consistent across DOX treatments regardless of IL-17 inclusion (**Figure 7A**). PD-1 ligand (*CD274*, PD-L1) was up-regulated upon DOX treatment consistent with prior studies (45). The addition of recombinant IL-17A to the culture media during DOX exposure abrogated the *CD274* upregulation. Expression levels were found to be significantly different relative to DOX only treatment but not control cells. IL-17A was not found to significantly alter *CD274* gene expression levels when administered alone when compared to control or combinatorial conditions (**Figure 7B**).

Consistent with prior reports, gene set enrichment analysis of the transcriptional profiles determined that DOX elicits enrichment in genes associated with cellular stress response pathways (ATF4, EIF2AK1, NFKB, ATR, P53) (46–48) while down-regulating pathways associated with cellular proliferation (BMP, MET) (49, 50) regardless of IL-17A co-administration (**STable 1**). Cell to cell signaling (*Braf* and *Notch)* and glycolysis (51) were also found to be consistently down-regulated in response to DOX independent of IL-17A treatment (**Figure 7C**). Furthermore, enrichment scores for specific biological processes were discovered to be affected by the co-administration of IL-17A with DOX. Specifically, genes correlating with cell cycle dysfunction in progression from G1 to S phase were also found to be highly up-regulated when IL-17A was co-administered, as well as genes associated with increased immune recruiting (*IL4, IL10,* and *IL13*). IL-17A mitigated pathways of immune activation that were upregulated because of DOX treatment including inhibition of MHC1 antigen presentation, IL2 signaling, and interferon α/β signaling (**Figure 7D**). Consistent with previous studies (52–54), mitogen-activated protein kinase (MAPK) activation was found to be increased in 4T1 cells treated with IL-17 and DOX relative to DOX alone (**Figure 7E**).

## Discussion

**Figure 8:**
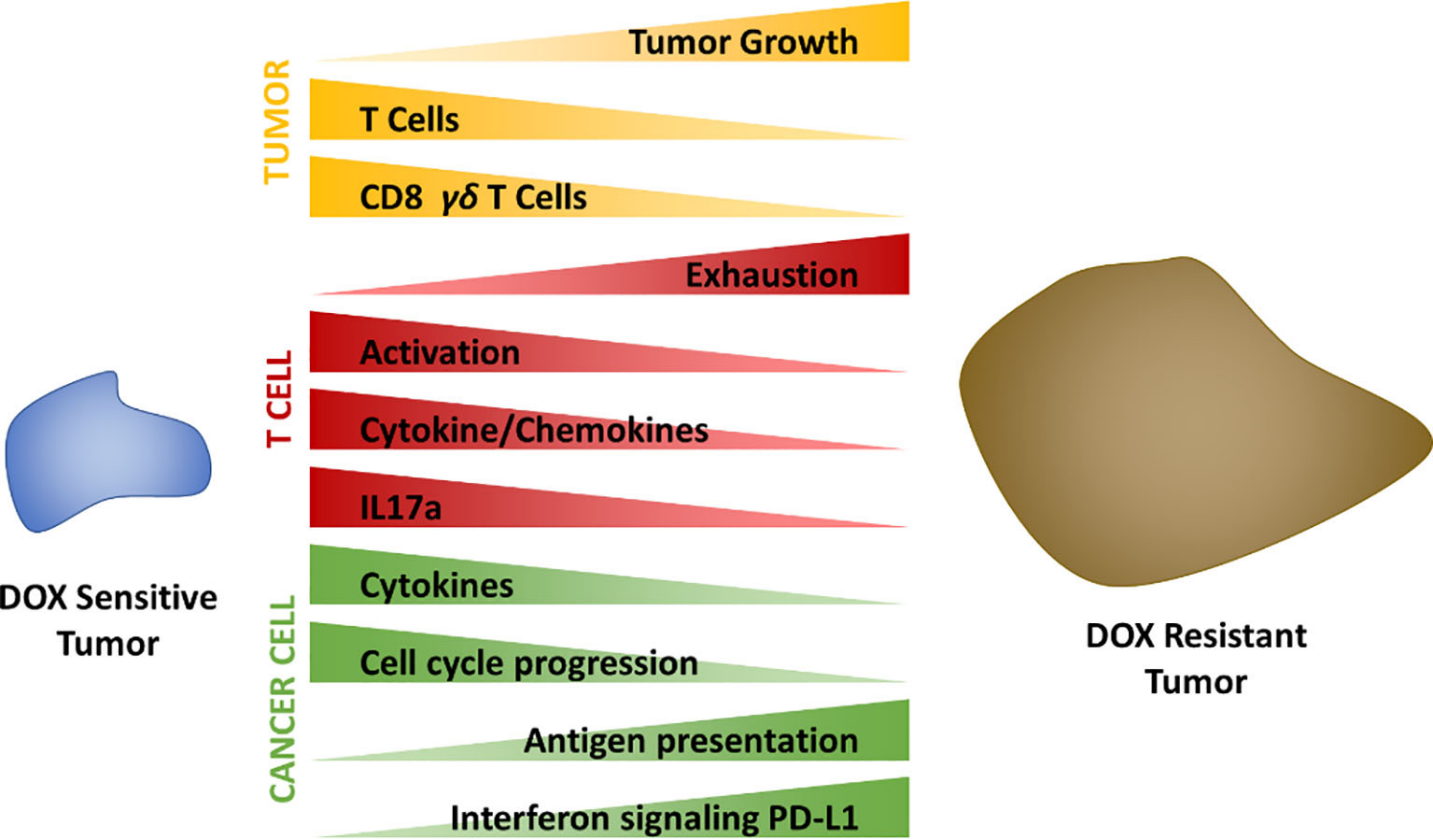
Summary schematic.

This study demonstrates the importance of stromal cellular composition to chemotherapeutic efficacy (**Figure 8**). Single cell transcriptomics and flow cytometric analysis identified increased T cell abundance to correlate with tumors sensitivity to DOX treatment relative to resistant tumors. Upon deeper transcriptomic characterization of tumor infiltrated T cell populations, alterations in the activity, behavior, and subtypes of those T cells were found to also be strongly correlated to chemotherapeutic response. Furthermore, IL-17A secreted by T cells was found to play a direct role in cancer cell sensitivity to DOX however transcriptional alterations as a result of IL-17A in cancer cells also likely contributed to signaling cascades inducing T cell exhaustion, recruitment and activation.

Despite the overall increase in T cells in sensitive tumors, the TME of resistant tumors were found to possess an increase in effector CD8 cells, however, these T cells exhibited increased exhaustion as assessed by the transcriptional expression of *Pdcd1, Cd27*, and *Lag3.* Increased levels of these genes has been previously linked to decreased effector function and failure to eliminate cancer cells (55). This cellular state manifests distinctly from the chronic stimulation of antigen in tumors. Interestingly, cells exposed to DOX alone induced several processes contributing to this phenotype such as antigen presentation, interferon signaling, and increased *CD274* (PD-L1) expression. The upregulation of these processes was significantly mitigated upon co-administration of IL-17A protein, suggesting that the anti-exhaustion inducing effects are derived from the cancer cells. The increased cytotoxic gene expression (upregulated *Prf1* and *GzmB)* in T cells found in chemo-sensitive tumors further substantiates the increased anti-cancer activity of IL-17A+ DOX sensitive tumors.

In addition to increased activity of cytotoxic effector T cells in DOX sensitive tumors, elevated chemokine and cytokine secretion was inferred from the increased abundance of CD4 T helper cell and γδ T cell populations. Chemokines identified, such as *Ccl3, Ccl4, Ccl5*, and *Cxcl9*, have demonstrated roles in increasing T cell infiltration into the TME (56, 57). Furthermore, CC chemokines have also been correlated to improved prognosis in breast cancer patients (57). These potent chemotactic molecules may contribute to the increased infiltration of T cells into the DOX sensitive TME increasing anti-cancer effects through an increased quantity of active T cells. ScRNA-Seq further identified increases in *Il-4*, *IL-5*, *IL-10, Il-13, IL-17A*, and *Il-21* transcripts. These cytokines support a wide range of biological functions within the TME (58). Specifically, IL-4 and IL-13 have demonstrated induction of cancer apoptosis as well as anti-inflammatory and innate immune activation functions (59, 60). IL-10 also shares immunosuppressive function in addition to proven roles in antiangiogenic function in tumors and a correlation with improved prognosis in breast cancer (61–63). These functions suggest that the sensitive TME may be stimulated for increased anti-cancer function while modulating the infiltration from pro-tumor stromal cell populations. Despite the anti-tumor functions described previously, cytokine function in the context of DOX treatment will require further studies to fully elucidate the effects these cytokines have in the TME.

Interestingly, IL-21 can play a role in stimulating IL-17 production and has also been found to be upregulated in sensitive tumors (55). Surprisingly, the IL-17A production was largely identified to be secreted from γδ but not Th17 T cells in 4T1 tumors. Specifically, sensitive tumors expanded the γδ CD8+IL-17A+ T cell population resulting in a net increase in *IL-17A* protein levels. This potent cytokine has potentially wide-spanning effects on numerous cell types found in the tumor and has a direct effect on increasing DOX efficacy in cancer cells. The presence of IL-17A has been examined in multiple cancer types and has emerged as an attractive cancer biomarker (64, 65). Studies on the impact of IL-17A in the TME have yielded both pro- and anti-tumor functions (20, 41). This phenomenon may be driven by the unique TME compositions found in different cancer types. IL-17 in developing tumors was found to have a negative correlation with survival, enhanced tumor development, or poor prognosis in numerous tumor types including breast cancer, head and neck, ovarian, prostate, and colorectal cancer (64–67) yet anti-tumor benefits have also been identified in and esophageal squamous cell carcinoma (68, 69). Contrasting anti and protumor effects have also been reported within the same tumor type as seen in melanoma (70, 71) and lung cancer (72, 73) suggesting that TME heterogeneity independent of tissue of origin may also play a contributing role in IL-17 response. This study determined no observable alterations in proliferation when 4T1 TNBC cells were exposed to IL-17 alone. IL-17A increased anti-cancer phenotypes observed *in vitro* only upon coadministration with DOX suggesting an alternate function in the presence of chemotherapeutics. The increased tumor responsiveness to chemotherapeutics in the presence of IL-17 has been previously reported in a range of cancer types (20–22, 74). The data provided here supports the benefit of IL-17 in the TME upon anthracycline administration in syngeneic TNBC tumors and functionally characterizes this correlation to direct and indirect effects on cancer elimination.

The findings presented here suggest possible therapeutic benefits of IL-17A coadministration or stimulation in conjunction with anthracycline treatment regimens. Several outstanding concerns still need to be addressed prior to clinical implementation to better understand the pleiotropic pro and anti-tumor effects noted previously. Furthermore, the long-term effects of the presence of these IL-17A producing cells in TNBC following DOX treatment will require further analysis to establish therapeutic efficacy as studies on the effects of IL-17 in tumor development and progression have been controversial (20, 21, 73) and outcomes may be dependent on tumor subtypes and stage of the disease.

The source of γδ IL-17A+ T cell populations was not identified in this study. Future therapeutic avenues utilizing γδ IL-17A+ T cells will require investigating cellular steps involved in differentiation pathways and/or means of recruitment into the TME. Recruitment appears to be a viable therapeutic option as intratumoral adoptive cell transfer of γδ T cells during DOX administration has been demonstrated to rescue the efficacy of chemotherapeutics in IL-17A knockout mice. However, subpopulations of γδ T cells may need to be selected for as T cells lacking IL-17A were unable to recover the sensitive phenotype (21). Additionally, the molecular cues triggering the presence of these beneficial T cells within the course of doxorubicin treatment will allow for the identification of novel therapeutic targets. The merits of recombinant IL-17A coadministration DOX may also provide therapeutic benefits yet bioavailability, targeting, and stability of this molecule will need to be optimized to evaluate utility. Overall, the data presented herein strongly supports the contribution of IL-17A produced from γδ T cells for modulating a tumor microenvironment with increased T cell infiltration and cytotoxic activity upon exposure to DOX in TNBC.

## Data Availability Statement

The datasets presented in this study can be found in the NCBI Gene Expression Omnibus under GEO series number GSE205552.

## Ethics Statement

The animal study was reviewed and approved by LLNL IACUC.

## Author Contributions

Conceptualization, NH and GL; data acquisition, NH, KM, NR-A, SG, and DG formal analysis, NH and AS; writing NH and GL; project administration, EW, MC, and GL. All authors have read and agreed to the published version of the manuscript.

## Funding

The research was supported by LLNL LDRD-19-SI-003, LDRD-17-ER-121. LDRD 21-LW-028, GL was also supported in part by a UC Davis Comprehensive Cancer Center Support Grant (CCCSG) awarded by the National Cancer Institute (NCI P30CA093373).

## Conflict of Interest

The authors declare that the research was conducted in the absence of any commercial or financial relationships that could be construed as a potential conflict of interest.

## Publisher’s Note

All claims expressed in this article are solely those of the authors and do not necessarily represent those of their affiliated organizations, or those of the publisher, the editors and the reviewers. Any product that may be evaluated in this article, or claim that may be made by its manufacturer, is not guaranteed or endorsed by the publisher.
