# Supplementary material for: IL-17A Increases Doxorubicin Efficacy in Triple Negative Breast Cancer
Source: Front Oncol. 2022 Jul 18;12:928474. doi: 10.3389/fonc.2022.928474 (PMC9340269; doi:10.3389/fonc.2022.928474)
Supplement: Supplementary file 1 [file DataSheet_1.pdf]

Supplementary Table 1. Differentially expressed genes in 4T1 cells exposed to doxorubicin and/or IL-17A

	Il17 vs Ctrl			*Dox+Il17 vs Il17*			*Dox vs Ctrl*		
**Gene**	**log2(Fold Change)**	**p value**	**adj p value**	**log2(Fold Change)**	**p value**	**adj p value**	**log2(Fold Change)**	**p value**	**adj p value**
Lce1g	2.5079	0.1308	0.9995	4.2637	0.0007	0.0151	6.7127	0.0001	0.0026
Sycp1	0.0948	0.9453	0.9995	6.4519	0.0000	0.0017	6.5330	0.0000	0.0006
Speer3	1.5754	0.3650	0.9995	4.1870	0.0035	0.0366	6.5152	0.0001	0.0035
Il6	0.0948	0.9533	0.9995	7.5871	0.0000	0.0014	6.4338	0.0000	0.0018
Rnf223	1.6103	0.2420	0.9995	3.0309	0.0054	0.0481	6.0574	0.0000	0.0014
Oas2	0.5403	0.7434	0.9995	2.9093	0.0342	0.1437	5.9381	0.0001	0.0036
Ifi209	0.8267	0.6297	0.9995	4.2228	0.0037	0.0383	5.9155	0.0003	0.0052
Serpina3h	0.5965	0.7648	0.9995	5.9566	0.0009	0.0174	5.8765	0.0010	0.0118
Gm6650	0.5965	0.7398	0.9995	4.9514	0.0020	0.0263	5.8338	0.0005	0.0072
Mx1	0.9923	0.6226	0.9995	2.3580	0.1631	0.3691	5.7744	0.0014	0.0146
Tg	0.0948	0.9399	0.9995	4.8163	0.0001	0.0068	5.7707	0.0000	0.0009
U90926	0.6001	0.6797	0.9995	8.3097	0.0000	0.0002	5.7524	0.0001	0.0022
Dok2	1.8499	0.2530	0.9995	2.0784	0.0963	0.2679	5.7119	0.0003	0.0061
Serpinb2	-1.3228	0.3466	0.9995	6.7421	0.0000	0.0016	5.6334	0.0000	0.0012
Spib	0.1768	0.9243	0.9995	-0.3993	0.8367	0.9210	5.6241	0.0006	0.0085
Cpq	0.0948	0.9427	0.9995	3.9316	0.0020	0.0260	5.5685	0.0000	0.0015
Cyp3a13	1.3556	0.4868	0.9995	3.5262	0.0339	0.1426	5.5078	0.0018	0.0173
Ccl17	3.3884	0.0148	0.9995	2.2475	0.0083	0.0610	5.4589	0.0001	0.0037
Ccl2	1.2995	0.3414	0.9995	4.2353	0.0004	0.0115	5.4398	0.0001	0.0027
Stxbp5l	0.0948	0.9593	0.9995	4.7003	0.0061	0.0510	5.4344	0.0011	0.0129
Bank1	0.0948	0.9440	0.9995	5.1691	0.0001	0.0068	5.4167	0.0001	0.0020
Plekhs1	0.2400	0.7294	0.9995	4.3418	0.0000	0.0002	5.4056	0.0000	0.0000
Rapgef4	3.3707	0.0575	0.9995	1.2811	0.3061	0.5330	5.4021	0.0020	0.0181
Trim30c	2.2205	0.2355	0.9995	1.2009	0.3754	0.5993	5.3794	0.0026	0.0216
Serping1	2.3936	0.1090	0.9995	3.0427	0.0083	0.0613	5.3450	0.0003	0.0061
Gbp11	0.5965	0.7335	0.9995	2.9460	0.0544	0.1904	5.3304	0.0009	0.0112
Pglyrp2	-0.5570	0.7543	0.9995	3.3681	0.0445	0.1683	5.2765	0.0008	0.0102
Sema3a	2.5478	0.0633	0.9995	1.7393	0.0683	0.2155	5.2709	0.0002	0.0043
Syt1	-0.3638	0.7903	0.9995	4.9114	0.0003	0.0104	5.2565	0.0001	0.0024
Map7d2	0.0948	0.9437	0.9995	4.2262	0.0012	0.0203	5.2253	0.0001	0.0026
Slc5a1	0.5965	0.6729	0.9995	3.1852	0.0131	0.0810	5.2122	0.0002	0.0038
Tnf	0.8021	0.5754	0.9995	4.7891	0.0003	0.0109	5.2121	0.0002	0.0042
Aloxe3	3.7379	0.0096	0.9995	1.9324	0.0241	0.1167	5.1539	0.0004	0.0066
Xirp2	0.0948	0.9418	0.9995	3.5474	0.0045	0.0432	5.1158	0.0001	0.0025
Maats1	0.0948	0.9369	0.9995	5.5372	0.0000	0.0020	5.0855	0.0000	0.0016
Anxa13	-0.4566	0.7432	0.9995	4.7202	0.0006	0.0135	5.0509	0.0001	0.0036
Dnah5	0.8202	0.6293	0.9995	3.5799	0.0156	0.0894	5.0489	0.0012	0.0132
Serpinb9c	0.9878	0.4953	0.9995	3.4502	0.0055	0.0486	5.0221	0.0003	0.0059
Plcb1	1.2409	0.3616	0.9995	3.5428	0.0034	0.0362	4.9790	0.0002	0.0044
Cd93	1.5792	0.3886	0.9995	3.7316	0.0069	0.0551	4.9719	0.0040	0.0283
Dnah14	0.0948	0.9413	0.9995	3.4785	0.0048	0.0450	4.9644	0.0001	0.0030
Dennd1c	0.9156	0.5344	0.9995	2.7984	0.0356	0.1470	4.9617	0.0004	0.0070
4932431P20Rik	0.0948	0.9547	0.9995	2.8014	0.0698	0.2189	4.9401	0.0012	0.0136
Cngb3	0.0948	0.9444	0.9995	2.6183	0.0437	0.1666	4.9266	0.0002	0.0044
Gsdmc2	-0.3638	0.8118	0.9995	4.6812	0.0013	0.0217	4.9246	0.0005	0.0072
4930555B11Rik	0.0948	0.9454	0.9995	1.0841	0.4218	0.6401	4.9110	0.0002	0.0050
Cxcl9	0.0673	0.9635	0.9995	2.2920	0.0760	0.2314	4.8990	0.0003	0.0057

Serpina3g	0.4724	0.7793	0.9995	4.4514	0.0030	0.0336	4.8966	0.0012	0.0131
Igf1	0.0948	0.9469	0.9995	3.7626	0.0056	0.0491	4.8954	0.0003	0.0060
D7Ertd443e	0.1804	0.9110	0.9995	4.0761	0.0058	0.0496	4.8693	0.0009	0.0111
Gm7609	0.8713	0.6075	0.9995	1.2452	0.4149	0.6339	4.8681	0.0017	0.0169
Tex15	0.0948	0.9422	0.9995	5.5636	0.0000	0.0031	4.8617	0.0001	0.0038
Myh11	-0.0129	0.9932	0.9995	1.1030	0.4522	0.6650	4.8357	0.0005	0.0075
C130073F10Rik	0.6697	0.6502	0.9995	3.3758	0.0083	0.0610	4.8212	0.0004	0.0071
a	0.0948	0.9401	0.9995	5.1389	0.0001	0.0046	4.8149	0.0001	0.0033
Ifit1bl1	0.0948	0.9485	0.9995	1.2268	0.3846	0.6071	4.7555	0.0006	0.0084
Prl2c1	3.3368	0.0599	0.9995	0.1848	0.8786	0.9421	4.7387	0.0065	0.0393
Nwd2	-0.3638	0.8178	0.9995	1.7477	0.2551	0.4794	4.7329	0.0009	0.0113
Icam1	1.8468	0.0730	0.9995	2.5588	0.0005	0.0124	4.7290	0.0000	0.0016
Krt42	0.0948	0.9464	0.9995	2.4931	0.0626	0.2057	4.7142	0.0004	0.0072
Krt17	1.0415	0.5051	0.9995	1.4968	0.2503	0.4745	4.7095	0.0016	0.0162
Pcdh15	0.0948	0.9472	0.9995	1.8205	0.1763	0.3857	4.6960	0.0005	0.0080
Nexmif	0.0948	0.9474	0.9995	0.7534	0.5934	0.7698	4.6870	0.0006	0.0084
Tmem130	-1.3751	0.2036	0.9995	5.3425	0.0000	0.0018	4.6755	0.0000	0.0004
Casp4	2.2561	0.0581	0.9995	2.3288	0.0066	0.0536	4.6563	0.0001	0.0035
Ifi44	-0.0284	0.9561	0.9995	2.4012	0.0000	0.0038	4.6433	0.0000	0.0000
Ifit1bl2	0.6078	0.6840	0.9995	2.3850	0.0565	0.1943	4.6061	0.0010	0.0116
Trim30d	0.1221	0.9457	0.9995	2.3279	0.1449	0.3429	4.5993	0.0019	0.0178
Gbp5	-0.0538	0.9630	0.9995	2.9002	0.0057	0.0494	4.5939	0.0001	0.0020
Plce1	-0.1930	0.9000	0.9995	4.3034	0.0029	0.0327	4.5927	0.0009	0.0110
Csf2	2.3042	0.0886	0.9995	3.7650	0.0003	0.0111	4.5808	0.0007	0.0098
Ccl4	0.1804	0.8940	0.9995	3.7164	0.0034	0.0360	4.5802	0.0004	0.0063
Gcnt1	0.5965	0.6285	0.9995	4.4123	0.0003	0.0096	4.5786	0.0002	0.0042
Tnfsf15	0.1183	0.8933	0.9995	4.3306	0.0000	0.0017	4.5357	0.0000	0.0003
Isg15	0.2709	0.5910	0.9995	1.8364	0.0006	0.0137	4.5259	0.0000	0.0000
Dock4	2.4586	0.0653	0.9995	1.9183	0.0334	0.1414	4.5058	0.0008	0.0100
Gm16793	0.6389	0.6640	0.9995	4.1922	0.0021	0.0271	4.5053	0.0011	0.0122
Il22ra1	1.5532	0.2944	0.9995	4.1637	0.0011	0.0198	4.5035	0.0015	0.0155
Cxcl10	0.3467	0.6519	0.9995	2.3886	0.0009	0.0177	4.4799	0.0000	0.0002
Arhgdib	2.6166	0.0501	0.9995	1.9962	0.0214	0.1079	4.4752	0.0009	0.0110
Calcb	0.0948	0.9511	0.9995	1.0841	0.4712	0.6804	4.4736	0.0017	0.0166
Zfp169	2.3341	0.1779	0.9995	0.3845	0.7893	0.8914	4.4722	0.0069	0.0408
Nrxn3	0.8021	0.6582	0.9995	4.3909	0.0052	0.0465	4.4596	0.0059	0.0369
Ace2	-0.5570	0.7633	0.9995	2.1245	0.2140	0.4334	4.4558	0.0050	0.0334
Siglecg	0.8960	0.4508	0.9995	1.1812	0.2474	0.4717	4.4539	0.0001	0.0035
Gm10634	-1.6729	0.2273	0.9995	5.2185	0.0002	0.0085	4.4539	0.0001	0.0030
4921511M17Rik	0.0948	0.9508	0.9995	0.5684	0.7059	0.8410	4.4486	0.0017	0.0166
Gm10057	0.0948	0.9508	0.9995	0.5684	0.7059	0.8410	4.4486	0.0017	0.0166
Gm15140	0.0948	0.9508	0.9995	0.5684	0.7059	0.8410	4.4486	0.0017	0.0166
Gm15143	0.0948	0.9508	0.9995	0.5684	0.7059	0.8410	4.4486	0.0017	0.0166
Gm5646	0.0948	0.9508	0.9995	0.5684	0.7059	0.8410	4.4486	0.0017	0.0166
Samt1	0.0948	0.9508	0.9995	0.5684	0.7059	0.8410	4.4486	0.0017	0.0166
BC023105	1.2025	0.4091	0.9995	-0.5393	0.7022	0.8397	4.4470	0.0013	0.0139
Mx2	-0.0594	0.9566	0.9995	2.3633	0.0167	0.0932	4.4164	0.0000	0.0014
Cntnap2	0.0948	0.9414	0.9995	4.1035	0.0012	0.0202	4.3917	0.0004	0.0070
Pla2g4c	1.1625	0.4108	0.9995	2.2989	0.0534	0.1881	4.3860	0.0013	0.0141
Rbms3	0.0948	0.9516	0.9995	4.1662	0.0041	0.0406	4.3784	0.0021	0.0191
Kcnip4	0.6357	0.6804	0.9995	2.4639	0.0750	0.2295	4.3646	0.0021	0.0191

Il13ra2	0.5507	0.7538	0.9995	6.2541	0.0001	0.0064	4.3607	0.0052	0.0342
Atp6v1c2	-0.2008	0.8994	0.9995	3.9962	0.0053	0.0472	4.3574	0.0017	0.0168
Nmu	0.6107	0.6900	0.9995	0.5545	0.7055	0.8410	4.3262	0.0022	0.0195
Neb	-0.4761	0.7338	0.9995	3.6638	0.0042	0.0412	4.3203	0.0005	0.0077
Prlr	0.0948	0.9501	0.9995	5.0576	0.0005	0.0127	4.3150	0.0020	0.0184
Dnah10	0.5965	0.6862	0.9995	1.6996	0.2100	0.4282	4.3029	0.0017	0.0168
Zbp1	0.3242	0.5053	0.9995	2.2237	0.0000	0.0024	4.2936	0.0000	0.0000
Trp53cor1	0.0948	0.9557	0.9995	2.8748	0.0681	0.2154	4.2701	0.0052	0.0340
Gm10419	0.4137	0.7712	0.9995	3.0066	0.0140	0.0839	4.2664	0.0010	0.0117
Ifit3b	-0.3442	0.7029	0.9995	2.3663	0.0076	0.0579	4.2626	0.0000	0.0003
1700023F02Rik	0.0948	0.9518	0.9995	2.0854	0.1646	0.3709	4.2408	0.0030	0.0236
Gap43	1.0999	0.3856	0.9995	3.5926	0.0019	0.0260	4.2339	0.0006	0.0084
Rpe65	0.0948	0.9483	0.9995	3.4671	0.0124	0.0789	4.2184	0.0019	0.0176
Gsdmc3	-0.3638	0.8118	0.9995	4.2322	0.0032	0.0350	4.2154	0.0022	0.0193
Fst	0.5046	0.7885	0.9995	2.8249	0.0583	0.1973	4.2143	0.0091	0.0491
Cfb	1.9763	0.0133	0.9995	2.7672	0.0000	0.0017	4.2086	0.0000	0.0005
Saa3	1.8280	0.3930	0.9995	6.4314	0.0002	0.0073	4.2081	0.0300	0.1058
Ahrr	0.5965	0.7000	0.9995	-0.0240	0.9875	0.9951	4.2066	0.0030	0.0234
Lama2	-0.6718	0.6518	0.9995	4.9079	0.0008	0.0166	4.2023	0.0015	0.0154
Slc2a6	0.0705	0.9672	0.9995	-0.0622	0.9701	0.9862	4.1892	0.0050	0.0332
Dapl1	0.0948	0.9446	0.9995	3.4064	0.0081	0.0605	4.1754	0.0012	0.0130
Gm3448	2.3101	0.0706	0.9995	1.6795	0.0764	0.2322	4.1617	0.0011	0.0126
Rbfox1	0.5965	0.6782	0.9995	4.2652	0.0014	0.0219	4.1587	0.0018	0.0175
Plb1	1.3161	0.3353	0.9995	2.9787	0.0079	0.0595	4.1433	0.0016	0.0161
Ankrd33b	2.7411	0.0300	0.9995	-0.1975	0.8307	0.9171	4.1355	0.0010	0.0120
Evi2a	2.2396	0.0868	0.9995	1.2635	0.1758	0.3851	4.1313	0.0013	0.0139
Slco1a5	-1.4911	0.3401	0.9995	4.6342	0.0019	0.0257	4.1232	0.0017	0.0166
Slc1a2	2.0478	0.1518	0.9995	4.6318	0.0002	0.0070	4.1229	0.0026	0.0216
Colec10	0.0948	0.9404	0.9995	3.9661	0.0014	0.0219	4.1150	0.0007	0.0097
Elavl4	0.0948	0.9399	0.9995	3.9933	0.0012	0.0204	4.1023	0.0007	0.0094
Cpeb1	0.0948	0.9496	0.9995	2.3312	0.0991	0.2727	4.0667	0.0030	0.0235
Nckap5	0.0948	0.9517	0.9995	3.5108	0.0171	0.0947	4.0632	0.0041	0.0293
Spag16	0.5965	0.7085	0.9995	3.0192	0.0374	0.1514	4.0627	0.0050	0.0331
Phf11a	-0.2968	0.8292	0.9995	3.3296	0.0057	0.0494	4.0604	0.0009	0.0112
Efcab3	0.0948	0.9482	0.9995	2.4334	0.0785	0.2364	4.0486	0.0026	0.0215
Prrx1	0.8119	0.6042	0.9995	1.3637	0.3225	0.5486	4.0449	0.0043	0.0300
Ddx25	0.0948	0.9474	0.9995	4.4776	0.0010	0.0191	4.0444	0.0024	0.0204
Rnf43	3.3284	0.0082	0.9995	-2.1495	0.0580	0.1969	4.0422	0.0011	0.0125
Rec114	0.0948	0.9486	0.9995	4.6749	0.0008	0.0170	4.0359	0.0028	0.0225
Nalcn	0.0948	0.9478	0.9995	0.8736	0.5345	0.7283	4.0266	0.0026	0.0216
Rab30	1.6627	0.1137	0.9995	1.1206	0.1479	0.3470	4.0234	0.0002	0.0046
Myo1f	-0.3638	0.8135	0.9995	0.7494	0.6201	0.7878	4.0013	0.0037	0.0268
Als2cr12	-1.1171	0.3880	0.9995	4.4235	0.0007	0.0156	4.0003	0.0003	0.0055
5031410I06Rik	0.4295	0.7511	0.9995	1.7195	0.1691	0.3761	4.0001	0.0015	0.0159
Col14a1	0.0948	0.9571	0.9995	4.5724	0.0045	0.0430	3.9995	0.0100	0.0526
A530040E14Rik	-0.3252	0.8379	0.9995	-0.3502	0.8292	0.9162	3.9901	0.0032	0.0245
Stra6	1.3545	0.3509	0.9995	1.3634	0.2550	0.4793	3.9656	0.0032	0.0247
Alox5ap	-0.1850	0.9044	0.9995	4.3511	0.0023	0.0291	3.9647	0.0030	0.0233
Epgn	-0.2308	0.8677	0.9995	2.9874	0.0187	0.0994	3.9631	0.0014	0.0146
Adgb	0.0948	0.9519	0.9995	0.5684	0.7123	0.8452	3.9513	0.0053	0.0344
P4ha3	2.0930	0.0483	0.9995	0.2508	0.7234	0.8523	3.9397	0.0003	0.0061

Cxcl11	-2.4826	0.0910	0.9995	3.4812	0.0176	0.0961	3.9367	0.0000	0.0018
Irgm2	-0.5891	0.5744	0.9995	2.5707	0.0069	0.0549	3.9302	0.0001	0.0033
Prr5l	0.5965	0.7038	0.9995	1.7460	0.2271	0.4493	3.9192	0.0060	0.0375
Tex11	0.0948	0.9445	0.9995	4.0206	0.0022	0.0278	3.9137	0.0020	0.0185
Pde6b	0.0948	0.9518	0.9995	3.3987	0.0172	0.0949	3.9079	0.0058	0.0368
Cd274	0.7605	0.2489	0.9995	1.2258	0.0260	0.1220	3.8872	0.0000	0.0002
Slc15a3	-0.4292	0.7669	0.9995	3.1436	0.0186	0.0991	3.8774	0.0020	0.0182
Cldn18	2.4195	0.0812	0.9995	-0.6650	0.5790	0.7612	3.8758	0.0038	0.0273
Vgll3	0.7773	0.5047	0.9995	3.5623	0.0005	0.0123	3.8742	0.0006	0.0084
Prx	0.2877	0.8451	0.9995	4.0017	0.0025	0.0303	3.8717	0.0037	0.0269
Fbxo43	-0.3638	0.8246	0.9995	2.2840	0.1474	0.3461	3.8676	0.0071	0.0417
Gm4841	1.0492	0.4965	0.9995	-2.1573	0.1923	0.4054	3.8643	0.0062	0.0383
Gch1	1.0929	0.3708	0.9995	2.9538	0.0011	0.0193	3.8442	0.0017	0.0169
A630001G21Rik	0.5368	0.7251	0.9995	1.1979	0.3952	0.6163	3.8413	0.0051	0.0338
Slc9a4	0.0948	0.9533	0.9995	4.0967	0.0063	0.0517	3.8324	0.0081	0.0456
A530046M15Rik	0.0948	0.9496	0.9995	2.2802	0.1076	0.2859	3.8304	0.0051	0.0335
Rsad2	0.3337	0.4362	0.9995	0.8440	0.0394	0.1565	3.8267	0.0000	0.0000
Dgkk	0.0948	0.9491	0.9995	1.9472	0.1677	0.3745	3.8253	0.0047	0.0319
Prnd	3.6557	0.0002	0.3624	1.0901	0.0153	0.0885	3.8209	0.0001	0.0027
Gm11351	0.0948	0.9489	0.9995	0.7494	0.6010	0.7746	3.8205	0.0047	0.0319
Runx1t1	1.4216	0.3642	0.9995	0.0337	0.9815	0.9917	3.8006	0.0084	0.0467
Tm4sf4	0.5965	0.7111	0.9995	1.1217	0.4529	0.6655	3.8004	0.0086	0.0475
Gbp6	0.6337	0.1827	0.9995	1.7900	0.0002	0.0076	3.8001	0.0000	0.0000
Tmem116	3.0373	0.0237	0.9995	-1.0555	0.3214	0.5475	3.7816	0.0040	0.0283
Gbp3	0.0812	0.8765	0.9995	1.5230	0.0040	0.0398	3.7667	0.0000	0.0000
Arsj	0.0948	0.9474	0.9995	3.3741	0.0136	0.0828	3.7631	0.0044	0.0302
Iigp1	-0.9218	0.3827	0.9995	1.6243	0.1117	0.2927	3.7523	0.0000	0.0017
Zfp534	-0.5971	0.7006	0.9995	1.9186	0.1943	0.4085	3.7457	0.0046	0.0313
Lmx1a	0.9808	0.5003	0.9995	-0.1326	0.9251	0.9661	3.7437	0.0053	0.0346
Rgs7bp	2.0581	0.1597	0.9995	-1.2396	0.3679	0.5926	3.7260	0.0074	0.0429
Zfp981	-0.5570	0.7489	0.9995	4.8500	0.0028	0.0322	3.7189	0.0125	0.0610
Edn1	1.0627	0.0471	0.9995	3.5344	0.0000	0.0001	3.7091	0.0000	0.0001
Mgam	-0.4593	0.7738	0.9995	3.3385	0.0219	0.1097	3.6991	0.0059	0.0371
Clgn	0.8021	0.5891	0.9995	3.8837	0.0038	0.0390	3.6956	0.0067	0.0401
Fndc7	2.3223	0.0314	0.9995	2.6939	0.0005	0.0124	3.6867	0.0007	0.0095
Mov10l1	0.0948	0.9513	0.9995	4.6693	0.0016	0.0236	3.6757	0.0087	0.0478
Gm3417	1.6243	0.2113	0.9995	2.4191	0.0159	0.0902	3.6742	0.0035	0.0263
Tcte3	1.6243	0.2113	0.9995	2.4191	0.0159	0.0902	3.6742	0.0035	0.0263
C330024C12Rik	-0.3638	0.7832	0.9995	-0.0729	0.9565	0.9823	3.6734	0.0024	0.0207
Mroh8	-0.5570	0.7194	0.9995	3.4173	0.0209	0.1064	3.6630	0.0069	0.0408
A530032D15Rik	-1.1559	0.4234	0.9995	1.1623	0.4063	0.6267	3.6472	0.0020	0.0185
Lrrc6	-0.8090	0.6472	0.9995	5.1618	0.0019	0.0257	3.6374	0.0136	0.0645
Scn7a	0.0948	0.9506	0.9995	3.5552	0.0123	0.0786	3.6359	0.0083	0.0464
Rrad	0.7038	0.6256	0.9995	3.4848	0.0063	0.0517	3.6307	0.0056	0.0360
Pdlim1	1.4186	0.1074	0.9995	1.4782	0.0223	0.1107	3.6205	0.0001	0.0028
Pet2	-0.3362	0.8095	0.9995	2.4151	0.0555	0.1928	3.6154	0.0019	0.0178
Gm5468	2.3486	0.0632	0.9995	2.6096	0.0068	0.0544	3.6143	0.0034	0.0253
Spink2	0.2638	0.8541	0.9995	3.5660	0.0068	0.0543	3.6102	0.0051	0.0338
Gm12250	-0.2746	0.8504	0.9995	-0.8777	0.5801	0.7614	3.5961	0.0030	0.0233
Hecw2	-1.1450	0.4074	0.9995	3.8809	0.0032	0.0351	3.5957	0.0010	0.0120
Ankrd45	0.6207	0.6382	0.9995	3.3265	0.0057	0.0494	3.5866	0.0035	0.0258

Phf11b	-0.1813	0.8694	0.9995	0.8020	0.4457	0.6594	3.5634	0.0004	0.0067
Tent5a	-0.6977	0.5871	0.9995	1.6832	0.1819	0.3921	3.5477	0.0013	0.0139
Gbp10	0.6578	0.2212	0.9995	1.5190	0.0021	0.0274	3.5457	0.0000	0.0001
Tgtp1	0.6659	0.3413	0.9995	1.0679	0.0636	0.2071	3.5330	0.0000	0.0010
Tgtp2	0.6471	0.3569	0.9995	1.0196	0.0781	0.2356	3.5200	0.0000	0.0010
Slc39a8	0.7295	0.5531	0.9995	2.3835	0.0181	0.0975	3.5098	0.0026	0.0216
Eln	0.0948	0.9429	0.9995	-0.0729	0.9561	0.9821	3.5093	0.0042	0.0296
1500015L24Rik	-0.1514	0.9037	0.9995	3.4512	0.0029	0.0328	3.5073	0.0017	0.0167
Cyb5rl	1.8764	0.1734	0.9995	1.3146	0.2157	0.4356	3.4881	0.0066	0.0398
Ifit3	-0.7064	0.1880	0.9995	2.0220	0.0005	0.0128	3.4606	0.0000	0.0000
Ifit1	-0.1363	0.8038	0.9995	1.0524	0.0482	0.1764	3.4601	0.0000	0.0001
Irf7	0.4067	0.4325	0.9995	1.1561	0.0194	0.1015	3.4472	0.0000	0.0001
Slc1a3	0.0948	0.9533	0.9995	4.5509	0.0025	0.0302	3.4294	0.0178	0.0759
Ctse	0.5956	0.6280	0.9995	2.4980	0.0159	0.0902	3.4239	0.0027	0.0223
Slco4a1	0.4343	0.6779	0.9995	2.7552	0.0021	0.0270	3.4217	0.0007	0.0091
Parm1	-0.1104	0.8915	0.9995	3.7667	0.0000	0.0023	3.4129	0.0000	0.0012
Atf3	0.4791	0.4393	0.9995	1.9238	0.0010	0.0191	3.3998	0.0000	0.0004
Tnfrsf9	2.3544	0.0192	0.9995	0.5908	0.3032	0.5302	3.3900	0.0010	0.0118
2610528J11Rik	-0.8054	0.5843	0.9995	-0.0729	0.9615	0.9844	3.3716	0.0080	0.0455
9230114K14Rik	-0.1033	0.8925	0.9995	3.5352	0.0000	0.0019	3.3651	0.0000	0.0012
Socs1	0.9916	0.4215	0.9995	1.8935	0.0416	0.1619	3.3624	0.0043	0.0298
Gm17767	1.7787	0.1791	0.9995	1.5978	0.1269	0.3159	3.3506	0.0082	0.0461
Irf5	1.1118	0.3548	0.9995	1.3027	0.1503	0.3505	3.3419	0.0038	0.0274
Gfpt2	2.0559	0.3081	0.9995	4.6873	0.0033	0.0355	3.3417	0.0666	0.1794
Gna14	1.0440	0.4540	0.9995	0.9549	0.4399	0.6551	3.3319	0.0093	0.0499
Helz2	-0.2746	0.8071	0.9995	1.8307	0.0580	0.1969	3.3128	0.0017	0.0167
Efnb3	-2.3999	0.0486	0.9995	4.5430	0.0004	0.0114	3.2956	0.0004	0.0072
5430421F17Rik	0.0847	0.9521	0.9995	2.9646	0.0213	0.1078	3.2882	0.0086	0.0473
Gbp4	-0.0765	0.8223	0.9995	1.6210	0.0001	0.0041	3.2857	0.0000	0.0000
Rbm38	0.6075	0.5123	0.9995	2.6038	0.0007	0.0160	3.2846	0.0005	0.0080
Ube2l6	0.2365	0.8360	0.9995	1.4988	0.1532	0.3544	3.2792	0.0015	0.0153
Zfpm2	1.6363	0.1176	0.9995	1.5178	0.0372	0.1509	3.2705	0.0018	0.0171
Bcl2l15	1.1063	0.0153	0.9995	2.9870	0.0000	0.0001	3.2676	0.0000	0.0000
Casq2	-0.9581	0.5323	0.9995	2.8231	0.0551	0.1920	3.2649	0.0082	0.0459
Pamr1	-1.3257	0.3198	0.9995	3.7344	0.0046	0.0435	3.2621	0.0036	0.0264
Cep295nl	0.1473	0.8858	0.9995	1.9363	0.0344	0.1440	3.2541	0.0007	0.0090
Dnah11	0.7135	0.5783	0.9995	2.7775	0.0173	0.0951	3.2522	0.0061	0.0379
Coq10b	1.5825	0.0399	0.9995	1.7265	0.0026	0.0315	3.2439	0.0001	0.0027
Cmpk2	-0.1118	0.8684	0.9995	1.7216	0.0076	0.0579	3.2424	0.0000	0.0006
Abca13	0.0948	0.9562	0.9995	4.7469	0.0027	0.0319	3.2374	0.0339	0.1145
Upk2	-1.3228	0.3423	0.9995	4.0357	0.0028	0.0321	3.2236	0.0061	0.0377
Rex2	-0.5432	0.6583	0.9995	3.3402	0.0043	0.0417	3.2231	0.0024	0.0207
Oas1g	-0.2350	0.6355	0.9995	1.9187	0.0005	0.0130	3.2128	0.0000	0.0001
Phex	1.6152	0.1659	0.9995	2.2555	0.0088	0.0634	3.1805	0.0048	0.0320
Gdnf	-0.9839	0.3685	0.9995	3.2266	0.0026	0.0311	3.1743	0.0005	0.0082
Abi3bp	1.0081	0.4483	0.9995	-0.7616	0.5707	0.7557	3.1642	0.0085	0.0471
Oas1a	-0.2844	0.5675	0.9995	1.9509	0.0005	0.0123	3.1606	0.0000	0.0001
Phf11d	-0.0242	0.9618	0.9995	1.7259	0.0009	0.0175	3.1462	0.0000	0.0001
Ptchd1	-1.1683	0.3915	0.9995	4.4957	0.0008	0.0168	3.1198	0.0056	0.0358
Cxcl2	1.6534	0.0915	0.9995	3.2831	0.0001	0.0038	3.1183	0.0016	0.0163
Mef2c	-0.4761	0.7893	0.9995	4.5432	0.0052	0.0467	3.1120	0.0355	0.1178

Hinfp	0.2105	0.8724	0.9995	1.6962	0.1294	0.3195	3.1093	0.0067	0.0401
Angptl4	0.8578	0.3907	0.9995	1.8967	0.0223	0.1107	3.0920	0.0013	0.0141
Trim30a	-0.1814	0.6922	0.9995	1.5511	0.0017	0.0247	3.0897	0.0000	0.0001
Akap6	-1.1048	0.3854	0.9995	3.6071	0.0033	0.0352	3.0499	0.0037	0.0269
Gm6623	1.7255	0.1323	0.9995	0.9440	0.1829	0.3934	3.0433	0.0082	0.0459
Efcab9	0.7533	0.5437	0.9995	2.7003	0.0132	0.0813	3.0392	0.0083	0.0464
Ttn	-0.5540	0.5966	0.9995	2.5819	0.0082	0.0607	3.0357	0.0007	0.0098
Usp18	-0.2981	0.5746	0.9995	1.6012	0.0036	0.0378	3.0255	0.0000	0.0002
Gm8801	1.0432	0.3647	0.9995	1.1058	0.1655	0.3718	3.0039	0.0081	0.0457
Slc16a6	-0.2878	0.6576	0.9995	3.7615	0.0000	0.0007	2.9968	0.0000	0.0007
Sh3rf2	-0.8234	0.5061	0.9995	1.7031	0.1520	0.3529	2.9935	0.0031	0.0238
Adgrf5	1.2576	0.0955	0.9995	1.8019	0.0023	0.0288	2.9925	0.0001	0.0037
Gsdmc	-0.3638	0.8075	0.9995	4.5726	0.0016	0.0233	2.9905	0.0221	0.0876
Cebpd	2.3363	0.0270	0.9995	2.4975	0.0005	0.0130	2.9826	0.0047	0.0316
Tnfrsf26	0.2768	0.7775	0.9995	2.5947	0.0031	0.0347	2.9794	0.0010	0.0118
Fyb	-0.0561	0.9661	0.9995	0.9547	0.4402	0.6552	2.9713	0.0081	0.0455
Ankdd1b	2.6790	0.0123	0.9995	0.1235	0.8636	0.9348	2.9593	0.0047	0.0319
Stat1	0.1482	0.7795	0.9995	1.0294	0.0466	0.1732	2.9574	0.0000	0.0003
Fam171b	-0.0969	0.8856	0.9995	2.3058	0.0007	0.0157	2.9395	0.0000	0.0010
Thbs3	0.3419	0.6921	0.9995	2.9252	0.0003	0.0104	2.9266	0.0004	0.0071
Sv2c	0.0948	0.9401	0.9995	4.4759	0.0004	0.0112	2.9238	0.0118	0.0587
Hsd11b1	-0.0899	0.9450	0.9995	2.8827	0.0151	0.0878	2.9185	0.0062	0.0384
Mars2	0.8426	0.3901	0.9995	1.3360	0.0944	0.2653	2.9165	0.0017	0.0167
Kbtbd8	-0.0547	0.9633	0.9995	3.1509	0.0034	0.0362	2.9136	0.0053	0.0345
Il18bp	1.2224	0.2735	0.9995	0.0026	0.9977	0.9987	2.9039	0.0066	0.0398
Rnd1	0.5724	0.2853	0.9995	2.2606	0.0000	0.0031	2.8979	0.0000	0.0004
Adh6b	-0.7166	0.3443	0.9995	1.5718	0.0321	0.1380	2.8936	0.0000	0.0017
Shpk	-0.9814	0.4307	0.9995	2.4774	0.0357	0.1472	2.8883	0.0049	0.0326
Selp	0.5771	0.4154	0.9995	3.0042	0.0000	0.0030	2.8676	0.0001	0.0032
Foxa1	0.5149	0.6462	0.9995	2.8091	0.0023	0.0286	2.8613	0.0062	0.0381
Casz1	0.5589	0.5115	0.9995	1.3923	0.0452	0.1698	2.8576	0.0007	0.0095
Ifi205	0.0979	0.8727	0.9995	1.8180	0.0027	0.0321	2.8482	0.0000	0.0010
Havcr2	1.6909	0.0715	0.9995	1.6588	0.0157	0.0899	2.8407	0.0019	0.0180
Oacyl	-1.6432	0.2809	0.9995	4.3869	0.0028	0.0322	2.8205	0.0148	0.0680
Sesn2	-0.6404	0.5351	0.9995	3.6767	0.0004	0.0119	2.8192	0.0014	0.0146
Xcr1	-1.1678	0.4762	0.9995	5.0042	0.0017	0.0244	2.8052	0.0355	0.1178
Lactb	0.9100	0.0627	0.9995	1.5159	0.0007	0.0160	2.7931	0.0000	0.0003
Oas1b	0.8392	0.2980	0.9995	0.3827	0.5677	0.7534	2.7753	0.0006	0.0089
Col17a1	0.8731	0.4379	0.9995	1.9594	0.0289	0.1293	2.7699	0.0089	0.0484
Dlk2	1.8384	0.0888	0.9995	0.6462	0.3412	0.5669	2.7696	0.0093	0.0500
Tenm1	0.0948	0.9485	0.9995	5.1468	0.0003	0.0104	2.7691	0.0366	0.1202
Ifi207	0.7586	0.4093	0.9995	0.1703	0.8253	0.9135	2.7482	0.0017	0.0168
Krt16	0.3175	0.6855	0.9995	1.7590	0.0148	0.0872	2.7335	0.0003	0.0055
Eaf1	0.2300	0.8279	0.9995	2.0999	0.0155	0.0890	2.7018	0.0066	0.0397
Rgs16	1.1408	0.1891	0.9995	2.7998	0.0001	0.0061	2.6968	0.0024	0.0207
Gbp7	0.1775	0.6323	0.9995	0.7618	0.0403	0.1588	2.6909	0.0000	0.0001
Igtp	0.1756	0.7013	0.9995	1.0141	0.0253	0.1198	2.6732	0.0000	0.0003
Icam4	2.5201	0.0086	0.9995	0.6702	0.2477	0.4719	2.6674	0.0042	0.0297
Ccdc115	0.8089	0.2872	0.9995	0.8781	0.1653	0.3717	2.6673	0.0009	0.0110
Ctu2	1.1101	0.1865	0.9995	1.3969	0.0292	0.1298	2.6621	0.0017	0.0168
Tnfrsf21	0.8698	0.1532	0.9995	1.1648	0.0170	0.0944	2.6587	0.0001	0.0024

Epsti1	1.0015	0.1662	0.9995	1.0016	0.0827	0.2450	2.6526	0.0003	0.0060
Stc2	-0.3991	0.7180	0.9995	2.4275	0.0178	0.0965	2.6512	0.0058	0.0369
Ebf1	0.4259	0.6633	0.9995	1.8773	0.0234	0.1146	2.6435	0.0037	0.0272
Gbp8	-0.7956	0.2847	0.9995	2.0084	0.0072	0.0561	2.6372	0.0001	0.0021
Cxcl1	2.3389	0.0172	0.9995	2.0036	0.0173	0.0952	2.6285	0.0061	0.0379
Bst2	-0.6815	0.3307	0.9995	2.0650	0.0046	0.0438	2.6085	0.0001	0.0020
Gm14057	-1.0891	0.4053	0.9995	3.7067	0.0027	0.0319	2.6071	0.0122	0.0598
Chac1	-0.1824	0.7309	0.9995	2.6468	0.0000	0.0020	2.5870	0.0000	0.0007
Xaf1	0.1791	0.6296	0.9995	1.2516	0.0014	0.0219	2.5824	0.0000	0.0001
AA467197	1.1772	0.1406	0.9995	0.4054	0.5058	0.7050	2.5779	0.0012	0.0136
Lrrc2	-2.7386	0.0566	0.9995	4.6455	0.0014	0.0222	2.5766	0.0110	0.0556
Mrc1	0.1622	0.8711	0.9995	2.7001	0.0031	0.0341	2.5678	0.0040	0.0284
Mfap3l	-1.5236	0.3156	0.9995	4.9101	0.0008	0.0166	2.5363	0.0248	0.0943
Dph2	1.6439	0.0188	0.9995	1.1377	0.0183	0.0980	2.5358	0.0004	0.0068
Arf2	0.1706	0.7754	0.9995	2.0670	0.0010	0.0184	2.5319	0.0000	0.0017
Igsf11	0.7846	0.2445	0.9995	1.8998	0.0014	0.0223	2.5305	0.0002	0.0047
Rapsn	0.6861	0.2316	0.9995	1.0964	0.0239	0.1160	2.5205	0.0000	0.0018
Afp	0.6855	0.3781	0.9995	1.7842	0.0063	0.0521	2.5160	0.0010	0.0121
Klrg2	0.1566	0.8388	0.9995	2.0916	0.0027	0.0320	2.5089	0.0007	0.0094
Hecw1	0.0948	0.9540	0.9995	4.5472	0.0027	0.0321	2.4977	0.0868	0.2144
Snhg15	0.8262	0.1049	0.9995	1.3812	0.0020	0.0266	2.4954	0.0000	0.0011
Car7	0.3429	0.7177	0.9995	0.2342	0.7897	0.8916	2.4870	0.0043	0.0298
Herc6	0.1571	0.6735	0.9995	1.0578	0.0053	0.0474	2.4730	0.0000	0.0001
H2-K1	0.8038	0.2685	0.9995	0.3204	0.6291	0.7935	2.4664	0.0014	0.0147
Unc80	-1.6195	0.3229	0.9995	5.2899	0.0008	0.0166	2.4590	0.0583	0.1641
Snord14e	1.7762	0.0346	0.9995	1.2397	0.0265	0.1229	2.4529	0.0033	0.0251
Aen	0.2689	0.6561	0.9995	2.0647	0.0010	0.0184	2.4327	0.0001	0.0031
Blnk	1.2409	0.0496	0.9995	1.5023	0.0032	0.0347	2.4278	0.0002	0.0042
Nubp2	0.3284	0.3567	0.9995	2.0694	0.0000	0.0008	2.4198	0.0000	0.0001
Gm17399	-0.1349	0.8940	0.9995	3.0559	0.0016	0.0238	2.4196	0.0060	0.0374
Prkcb	-0.1138	0.9006	0.9995	2.4659	0.0049	0.0452	2.4096	0.0030	0.0233
Gm13363	0.9657	0.1331	0.9995	0.6632	0.2463	0.4710	2.4089	0.0005	0.0081
Coprs	0.6619	0.0679	0.9995	1.2597	0.0007	0.0155	2.4083	0.0000	0.0001
Dclre1b	0.2191	0.7341	0.9995	2.1791	0.0004	0.0121	2.4024	0.0002	0.0044
Gadd45g	0.2345	0.6389	0.9995	1.0268	0.0316	0.1365	2.3941	0.0000	0.0011
Cdr2	0.8317	0.0693	0.9995	1.6669	0.0001	0.0055	2.3925	0.0000	0.0007
Gm5113	1.0018	0.2836	0.9995	0.6430	0.4167	0.6356	2.3873	0.0065	0.0393
Mafb	-0.2539	0.6990	0.9995	2.4816	0.0003	0.0103	2.3797	0.0001	0.0032
Dhx58	0.0830	0.8700	0.9995	0.3107	0.5160	0.7136	2.3635	0.0000	0.0012
Adra1b	0.1121	0.9090	0.9995	1.3075	0.1282	0.3177	2.3592	0.0069	0.0408
Slc5a6	0.5525	0.3208	0.9995	0.8653	0.0640	0.2074	2.3560	0.0001	0.0026
Slc31a2	0.4381	0.3638	0.9995	1.6599	0.0004	0.0117	2.3541	0.0000	0.0011
Samd9l	0.0940	0.7947	0.9995	1.4274	0.0004	0.0117	2.3498	0.0000	0.0001
A930006K02Rik	-0.5989	0.6027	0.9995	3.4886	0.0019	0.0255	2.3409	0.0132	0.0630
Gm19519	1.1956	0.4662	0.9995	4.0423	0.0041	0.0405	2.3385	0.1187	0.2628
Apol9a	-0.2244	0.6699	0.9995	0.7993	0.1226	0.3105	2.3373	0.0000	0.0011
Tmem158	0.9012	0.1507	0.9995	1.3041	0.0099	0.0685	2.3319	0.0004	0.0064
Ccdc114	-0.5732	0.5579	0.9995	3.0985	0.0014	0.0221	2.3204	0.0041	0.0288
Gm12059	0.0865	0.9316	0.9995	2.2373	0.0116	0.0754	2.3189	0.0085	0.0469
Eif2b1	0.7336	0.1199	0.9995	1.2893	0.0034	0.0364	2.3160	0.0000	0.0012
Ccdc180	1.3134	0.1548	0.9995	0.3351	0.6561	0.8099	2.3106	0.0083	0.0463

Ppp1r15a	0.3216	0.5250	0.9995	1.6050	0.0016	0.0233	2.2936	0.0000	0.0016
Plk2	0.2260	0.6380	0.9995	2.2943	0.0000	0.0030	2.2535	0.0000	0.0014
Ccdc137	-0.0171	0.9567	0.9995	2.3735	0.0000	0.0002	2.2533	0.0000	0.0001
Zfp850	0.1994	0.8204	0.9995	1.1389	0.1344	0.3277	2.2473	0.0050	0.0331
Oasl2	-0.0267	0.9373	0.9995	1.0292	0.0045	0.0431	2.2296	0.0000	0.0001
Pik3r3	0.2252	0.7647	0.9995	1.6345	0.0208	0.1061	2.2291	0.0012	0.0132
Pradc1	0.3522	0.6610	0.9995	1.5389	0.0272	0.1252	2.2198	0.0030	0.0236
Foxred1	0.7041	0.2425	0.9995	1.1388	0.0315	0.1363	2.2184	0.0005	0.0081
Teddm1b	0.7741	0.3652	0.9995	0.9402	0.1808	0.3909	2.2172	0.0065	0.0395
Ccdc25	0.1116	0.6871	0.9995	1.8356	0.0000	0.0007	2.2157	0.0000	0.0000
Plk3	-0.1493	0.8248	0.9995	2.4627	0.0004	0.0114	2.2153	0.0004	0.0068
Tigar	0.0557	0.8906	0.9995	2.3935	0.0000	0.0009	2.2118	0.0000	0.0004
Helq	0.6258	0.2030	0.9995	1.5040	0.0012	0.0206	2.2080	0.0000	0.0017
Milr1	-0.7577	0.5295	0.9995	4.8482	0.0001	0.0055	2.2077	0.0314	0.1091
Ifi27	0.1469	0.6288	0.9995	1.5479	0.0001	0.0042	2.2000	0.0000	0.0001
Miga2	1.0191	0.0800	0.9995	1.0161	0.0294	0.1304	2.1945	0.0002	0.0051
Trmt6	0.4210	0.2378	0.9995	1.4425	0.0003	0.0108	2.1922	0.0000	0.0003
Gm9268	-0.6838	0.5041	0.9995	2.4044	0.0123	0.0784	2.1904	0.0085	0.0468
Nanos1	0.5115	0.3261	0.9995	2.0543	0.0001	0.0049	2.1879	0.0001	0.0027
Usp44	1.8354	0.0113	0.9995	0.2175	0.6608	0.8133	2.1820	0.0022	0.0192
Gemin6	0.7334	0.3330	0.9995	0.9041	0.1318	0.3233	2.1717	0.0042	0.0294
Iscu	0.3296	0.6941	0.9995	1.3008	0.0750	0.2295	2.1625	0.0071	0.0417
Fstl1	-0.0838	0.9198	0.9995	2.1060	0.0085	0.0621	2.1576	0.0029	0.0229
Zeb2os	0.1781	0.7989	0.9995	1.8884	0.0042	0.0409	2.1492	0.0011	0.0126
Mcam	-0.1984	0.8071	0.9995	0.8078	0.2933	0.5209	2.1453	0.0026	0.0216
Tlr2	0.9853	0.1555	0.9995	-0.1543	0.7883	0.8911	2.1452	0.0018	0.0175
Cptp	0.9465	0.0726	0.9995	1.6882	0.0004	0.0111	2.1445	0.0002	0.0039
Fos	0.8493	0.3273	0.9995	2.7757	0.0004	0.0114	2.1437	0.0079	0.0452
Iqgap2	-1.8710	0.0549	0.9995	2.7051	0.0063	0.0517	2.1380	0.0016	0.0165
Akr1b3	0.5901	0.1558	0.9995	1.5491	0.0006	0.0147	2.1377	0.0000	0.0011
Exosc2	0.4471	0.2551	0.9995	1.6783	0.0001	0.0051	2.1350	0.0000	0.0006
AW112010	-0.9317	0.3149	0.9995	-0.3541	0.7195	0.8494	2.1341	0.0034	0.0257
Cep76	0.3444	0.6756	0.9995	1.4228	0.0394	0.1566	2.1333	0.0054	0.0348
Cox7a1	-0.4456	0.7716	0.9995	4.1791	0.0035	0.0370	2.1327	0.1055	0.2437
Relt	-0.9189	0.1909	0.9995	2.7759	0.0002	0.0079	2.1249	0.0004	0.0064
Mreg	-1.4893	0.1882	0.9995	3.8726	0.0009	0.0177	2.1233	0.0080	0.0455
Pla1a	0.8152	0.0877	0.9995	1.8709	0.0001	0.0040	2.1232	0.0001	0.0020
Bfsp1	-0.6947	0.6682	0.9995	5.5649	0.0004	0.0113	2.1154	0.1277	0.2743
Tbc1d30	-1.4820	0.1583	0.9995	2.2417	0.0399	0.1578	2.1054	0.0037	0.0269
Mtmr7	-0.1776	0.8391	0.9995	2.5601	0.0023	0.0289	2.0704	0.0069	0.0408
Hsd17b13	0.0948	0.9446	0.9995	4.4160	0.0009	0.0176	2.0699	0.0947	0.2271
Stx11	0.3928	0.5552	0.9995	1.1882	0.0371	0.1505	2.0666	0.0018	0.0173
Sp110	-2.0627	0.0162	0.9995	1.1302	0.1950	0.4093	2.0657	0.0002	0.0044
Ctsw	-0.5259	0.2116	0.9995	2.4426	0.0000	0.0012	2.0657	0.0000	0.0005
Abcg2	-0.1209	0.7801	0.9995	2.5064	0.0000	0.0011	2.0622	0.0000	0.0009
Max	0.7193	0.1598	0.9995	1.0997	0.0244	0.1176	2.0563	0.0003	0.0054
Zfand2a	0.0912	0.8444	0.9995	1.5258	0.0019	0.0258	2.0559	0.0001	0.0024
Psd	-0.0650	0.8639	0.9995	1.9005	0.0000	0.0031	2.0469	0.0000	0.0005
Trp53	-0.3803	0.6641	0.9995	2.0804	0.0124	0.0789	2.0467	0.0048	0.0321
Tcte2	-0.8598	0.3451	0.9995	2.3632	0.0092	0.0653	2.0461	0.0056	0.0357
F5	-0.9128	0.4310	0.9995	3.5351	0.0021	0.0271	2.0415	0.0249	0.0946

Cfap54	0.0948	0.9489	0.9995	4.3378	0.0021	0.0269	2.0400	0.1284	0.2752
Rtp4	-0.1077	0.7249	0.9995	1.0572	0.0020	0.0266	2.0379	0.0000	0.0001
Orc6	0.4719	0.2897	0.9995	1.4277	0.0018	0.0253	2.0355	0.0001	0.0022
Cdc6	0.5882	0.0443	0.9995	1.4424	0.0000	0.0021	2.0343	0.0000	0.0001
Tspan13	-2.3640	0.0537	0.9995	3.9730	0.0012	0.0207	2.0271	0.0120	0.0592
Gbp2b	0.7526	0.0413	0.9995	-0.2854	0.3962	0.6173	2.0245	0.0000	0.0005
Csrnp1	0.3938	0.5161	0.9995	1.0023	0.0559	0.1936	2.0211	0.0009	0.0109
Ddit3	-0.1291	0.7312	0.9995	1.9182	0.0000	0.0029	2.0204	0.0000	0.0005
Aoc2	0.1103	0.8662	0.9995	1.7112	0.0052	0.0466	2.0178	0.0012	0.0131
Ptp4a1	0.7053	0.1124	0.9995	0.7609	0.0731	0.2252	2.0137	0.0001	0.0026
Nsl1	0.5429	0.3508	0.9995	1.2276	0.0158	0.0901	2.0026	0.0007	0.0097
H2-T22	0.4143	0.4680	0.9995	0.7167	0.1637	0.3699	2.0008	0.0008	0.0104
Rcc1	0.7238	0.3216	0.9995	0.6285	0.3359	0.5615	1.9982	0.0066	0.0399
Zfp367	0.1704	0.7954	0.9995	1.5395	0.0155	0.0892	1.9952	0.0026	0.0215
Eif1ad	0.0507	0.9117	0.9995	1.7213	0.0006	0.0139	1.9951	0.0001	0.0027
Ddias	-0.0325	0.9619	0.9995	1.6452	0.0096	0.0669	1.9945	0.0016	0.0164
Oasl1	-0.0017	0.9969	0.9995	0.6167	0.1438	0.3412	1.9941	0.0000	0.0014
Slc25a25	-0.9046	0.0814	0.9995	3.1546	0.0000	0.0008	1.9921	0.0000	0.0012
Tapbpl	0.3796	0.3497	0.9995	0.5726	0.1420	0.3386	1.9863	0.0000	0.0014
Ndufaf4	0.7424	0.0683	0.9995	1.4619	0.0003	0.0103	1.9737	0.0000	0.0012
Ciart	0.0305	0.9646	0.9995	1.6191	0.0122	0.0779	1.9719	0.0019	0.0176
H2-T24	0.9056	0.2363	0.9995	-1.2790	0.1084	0.2866	1.9691	0.0068	0.0407
Siah1b	0.2528	0.4838	0.9995	1.8970	0.0000	0.0020	1.9582	0.0000	0.0007
Tor3a	0.1973	0.5073	0.9995	1.0357	0.0015	0.0225	1.9502	0.0000	0.0002
Fen1	0.2186	0.6522	0.9995	1.0776	0.0267	0.1237	1.9499	0.0002	0.0051
Eif2ak2	-0.1086	0.7315	0.9995	1.1307	0.0016	0.0234	1.9475	0.0000	0.0003
Slfn9	-0.0887	0.7917	0.9995	1.3101	0.0007	0.0156	1.9448	0.0000	0.0005
Rraga	0.9762	0.0893	0.9995	0.4680	0.3776	0.6013	1.9338	0.0014	0.0146
Dcn	-1.3226	0.2756	0.9995	4.2195	0.0007	0.0151	1.9315	0.0414	0.1309
Gm10375	-1.2229	0.3865	0.9995	4.0148	0.0042	0.0409	1.9313	0.0734	0.1917
Gm10377	-1.2229	0.3865	0.9995	4.0148	0.0042	0.0409	1.9313	0.0734	0.1917
Gm16062	1.4199	0.0414	0.9995	1.0466	0.0429	0.1649	1.9286	0.0043	0.0301
Tie1	-1.7160	0.0846	0.9995	3.3722	0.0011	0.0192	1.9277	0.0087	0.0476
Kti12	0.1985	0.7901	0.9995	1.7718	0.0070	0.0554	1.9273	0.0074	0.0429
Stat2	-0.1682	0.5849	0.9995	0.6925	0.0309	0.1350	1.9271	0.0000	0.0002
Irgm1	0.1318	0.7677	0.9995	0.4652	0.2696	0.4954	1.9113	0.0001	0.0022
Cited4	-1.4496	0.1829	0.9995	3.5045	0.0013	0.0216	1.9092	0.0152	0.0690
Timm23	0.6683	0.2514	0.9995	1.2521	0.0271	0.1249	1.9060	0.0019	0.0176
Mitd1	0.2980	0.3528	0.9995	0.5937	0.0666	0.2123	1.9050	0.0000	0.0005
Cdc42ep3	-0.0222	0.9705	0.9995	1.8562	0.0022	0.0284	1.8930	0.0013	0.0142
Aste1	0.6653	0.3623	0.9995	1.0514	0.0881	0.2541	1.8895	0.0061	0.0378
Cpox	0.4544	0.4468	0.9995	1.0676	0.0546	0.1909	1.8885	0.0022	0.0193
Dusp8	-0.3383	0.4429	0.9995	2.0236	0.0001	0.0041	1.8872	0.0000	0.0015
Zfp979	0.4059	0.4980	0.9995	1.6440	0.0026	0.0308	1.8840	0.0013	0.0143
Gm6644	0.3626	0.2236	0.9995	1.5710	0.0000	0.0029	1.8820	0.0000	0.0003
Scyl3	1.0566	0.1495	0.9995	0.2826	0.6272	0.7923	1.8802	0.0090	0.0487
Cd14	1.4870	0.0493	0.9995	1.6669	0.0022	0.0278	1.8798	0.0114	0.0572
Myd88	0.0789	0.8476	0.9995	1.2437	0.0031	0.0341	1.8763	0.0000	0.0018
Ifi35	-0.1620	0.6855	0.9995	1.3165	0.0024	0.0299	1.8711	0.0000	0.0012
Fosb	1.7351	0.0143	0.9995	0.3097	0.5127	0.7108	1.8692	0.0066	0.0397
Gon7	-0.0167	0.9632	0.9995	1.7550	0.0000	0.0038	1.8690	0.0000	0.0010

Apol9b	-0.0660	0.8455	0.9995	0.9261	0.0094	0.0659	1.8676	0.0000	0.0004
Clcn7	0.1511	0.6731	0.9995	1.3281	0.0006	0.0135	1.8632	0.0000	0.0007
B230208H11Rik	-0.0139	0.9801	0.9995	1.3061	0.0136	0.0827	1.8626	0.0005	0.0081
Nfkbib	0.2194	0.3216	0.9995	1.8082	0.0000	0.0001	1.8609	0.0000	0.0000
Slc25a44	0.1506	0.6823	0.9995	1.2881	0.0011	0.0196	1.8599	0.0000	0.0010
Qtrt2	0.4177	0.4265	0.9995	1.6527	0.0012	0.0201	1.8575	0.0005	0.0081
Slc11a2	1.2073	0.0398	0.9995	1.6926	0.0008	0.0162	1.8550	0.0017	0.0169
Cds1	0.5928	0.3500	0.9995	1.2527	0.0206	0.1055	1.8509	0.0028	0.0225
Apol7a	0.5031	0.1017	0.9995	0.0638	0.8200	0.9100	1.8505	0.0000	0.0002
Creb3	0.3910	0.2507	0.9995	0.7696	0.0175	0.0959	1.8410	0.0000	0.0006
Serpinb6b	0.4838	0.3818	0.9995	1.3558	0.0137	0.0831	1.8407	0.0016	0.0162
Trmt61a	0.2310	0.6305	0.9995	1.7244	0.0006	0.0141	1.8309	0.0003	0.0056
Oas3	0.1740	0.7057	0.9995	-0.2275	0.6185	0.7868	1.8276	0.0002	0.0045
Xrcc2	0.6763	0.2724	0.9995	1.4132	0.0070	0.0551	1.8263	0.0029	0.0232
Ube2g2	0.3666	0.5888	0.9995	1.0571	0.0881	0.2541	1.8261	0.0066	0.0397
Zfp142	0.6331	0.1711	0.9995	0.5214	0.2064	0.4235	1.8251	0.0002	0.0039
Gm18853	-0.8380	0.3006	0.9995	2.4835	0.0021	0.0272	1.8245	0.0048	0.0320
Trp53rkb	0.0790	0.8902	0.9995	1.1969	0.0252	0.1195	1.8229	0.0009	0.0109
Dnaja4	0.5589	0.2517	0.9995	1.5303	0.0011	0.0192	1.8140	0.0003	0.0057
Cenpu	0.5725	0.3029	0.9995	1.2306	0.0122	0.0778	1.8140	0.0012	0.0130
Prr3	0.1359	0.8099	0.9995	1.5557	0.0041	0.0404	1.8136	0.0010	0.0118
Ercc8	0.8401	0.1861	0.9995	0.8223	0.1287	0.3183	1.8118	0.0031	0.0241
Adar	-0.0241	0.9428	0.9995	0.8349	0.0162	0.0914	1.8112	0.0000	0.0006
Wdr77	0.6501	0.1031	0.9995	1.0525	0.0070	0.0551	1.8045	0.0001	0.0023
Cd74	-0.1019	0.8733	0.9995	1.1739	0.0622	0.2052	1.8016	0.0018	0.0173
Ifi47	0.1819	0.5689	0.9995	-0.0505	0.8724	0.9391	1.8001	0.0000	0.0005
Gm18852	-0.8981	0.2723	0.9995	2.5436	0.0019	0.0258	1.7997	0.0053	0.0346
Mindy3	-0.0643	0.8736	0.9995	2.0869	0.0000	0.0022	1.7990	0.0000	0.0019
Tuba4a	0.7534	0.1001	0.9995	0.8003	0.0679	0.2149	1.7966	0.0003	0.0062
Trim21	0.2222	0.6518	0.9995	0.4724	0.3039	0.5307	1.7919	0.0004	0.0065
Haspin	0.2126	0.5534	0.9995	1.2444	0.0009	0.0179	1.7918	0.0000	0.0011
Gins3	0.3084	0.4749	0.9995	1.2488	0.0047	0.0442	1.7882	0.0001	0.0036
H2-Q7	-0.6598	0.2994	0.9995	0.8423	0.1763	0.3857	1.7869	0.0014	0.0149
Enpp2	0.2529	0.6012	0.9995	0.6526	0.1454	0.3435	1.7722	0.0004	0.0065
Phf11c	-1.3570	0.0707	0.9995	1.9703	0.0095	0.0665	1.7706	0.0012	0.0136
Lig4	0.0750	0.8653	0.9995	0.8471	0.0491	0.1784	1.7661	0.0002	0.0039
Cops7b	1.0258	0.0183	0.9995	0.2597	0.4419	0.6563	1.7566	0.0001	0.0036
Rrp9	0.5308	0.1664	0.9995	1.8271	0.0000	0.0025	1.7562	0.0000	0.0017
Nfatc2ip	-0.3330	0.5451	0.9995	1.7781	0.0019	0.0256	1.7525	0.0005	0.0080
Timm9	0.4028	0.4654	0.9995	1.1940	0.0229	0.1131	1.7515	0.0020	0.0183
Fam53a	0.5083	0.2688	0.9995	0.6258	0.1371	0.3312	1.7507	0.0004	0.0068
Nbl1	0.2605	0.6763	0.9995	1.7536	0.0028	0.0321	1.7498	0.0028	0.0225
Slc10a3	0.5057	0.4447	0.9995	0.7426	0.1797	0.3897	1.7479	0.0073	0.0426
Snip1	0.5908	0.2585	0.9995	0.5708	0.2089	0.4265	1.7434	0.0012	0.0137
Lgals9	-0.4600	0.1423	0.9995	0.9835	0.0037	0.0383	1.7431	0.0000	0.0004
Pno1	0.3758	0.1798	0.9995	1.5551	0.0000	0.0016	1.7427	0.0000	0.0002
1700086P04Rik	0.4310	0.5142	0.9995	0.3228	0.5907	0.7679	1.7413	0.0050	0.0332
Areg	-0.1242	0.7927	0.9995	2.3046	0.0000	0.0029	1.7400	0.0003	0.0053
9930104L06Rik	0.9744	0.1025	0.9995	1.4533	0.0034	0.0360	1.7356	0.0032	0.0244
Klf16	0.3377	0.5196	0.9995	1.3333	0.0068	0.0545	1.7354	0.0012	0.0132
2810408I11Rik	-0.0401	0.9153	0.9995	2.0007	0.0000	0.0018	1.7351	0.0000	0.0015

Fastkd3	0.4508	0.2568	0.9995	1.2200	0.0021	0.0271	1.7297	0.0001	0.0025
Akirin1	0.6261	0.0726	0.9995	0.9835	0.0035	0.0367	1.7255	0.0000	0.0011
Slc35b1	0.0192	0.9663	0.9995	1.6251	0.0010	0.0191	1.7220	0.0003	0.0058
Dbr1	-0.1749	0.5927	0.9995	1.6807	0.0000	0.0034	1.7219	0.0000	0.0008
Traf4	0.2573	0.4272	0.9995	1.3465	0.0002	0.0076	1.7199	0.0000	0.0007
Timm10	0.4787	0.1866	0.9995	1.2352	0.0011	0.0192	1.7198	0.0000	0.0015
Wars	0.3417	0.2244	0.9995	1.4684	0.0000	0.0021	1.7168	0.0000	0.0003
Nppb	-2.5438	0.0861	0.9995	4.9230	0.0013	0.0209	1.7166	0.0672	0.1807
Jag1	-1.2769	0.0025	0.9995	2.9403	0.0000	0.0002	1.7147	0.0000	0.0010
Sephs2	0.2120	0.6414	0.9995	1.7356	0.0004	0.0116	1.7136	0.0004	0.0068
Utp4	0.6966	0.0994	0.9995	1.2618	0.0033	0.0355	1.7126	0.0002	0.0050
Arhgef3	0.2223	0.6101	0.9995	1.1748	0.0056	0.0487	1.7104	0.0002	0.0042
Asb6	0.1957	0.7258	0.9995	1.1744	0.0211	0.1072	1.7099	0.0022	0.0194
Klf4	-0.3912	0.2480	0.9995	0.8134	0.0202	0.1040	1.7070	0.0000	0.0007
Zfp707	0.5631	0.2096	0.9995	0.7013	0.0762	0.2318	1.7068	0.0004	0.0063
B2m	0.2552	0.4230	0.9995	0.8406	0.0148	0.0873	1.7023	0.0000	0.0012
Btbd10	0.3836	0.4531	0.9995	0.8032	0.0870	0.2524	1.6947	0.0013	0.0140
Trim12c	0.0092	0.9783	0.9995	0.5153	0.1264	0.3153	1.6942	0.0000	0.0011
Calr	0.5919	0.0737	0.9995	0.7505	0.0203	0.1045	1.6893	0.0000	0.0011
Fbxo33	0.4507	0.2136	0.9995	0.6861	0.0430	0.1651	1.6850	0.0000	0.0017
Mtfr2	0.3025	0.3756	0.9995	1.3474	0.0003	0.0096	1.6827	0.0000	0.0011
Bpgm	0.2698	0.6872	0.9995	1.2014	0.0389	0.1553	1.6795	0.0075	0.0434
Adh7	0.0795	0.8329	0.9995	1.1941	0.0027	0.0318	1.6721	0.0001	0.0026
5033406O09Rik	-0.1834	0.7448	0.9995	1.6302	0.0044	0.0421	1.6662	0.0011	0.0130
Mrps11	0.6544	0.2193	0.9995	1.0793	0.0164	0.0920	1.6596	0.0020	0.0182
2810013P06Rik	0.0777	0.8346	0.9995	1.5070	0.0002	0.0093	1.6585	0.0001	0.0020
Dll1	0.1859	0.7603	0.9995	1.2415	0.0253	0.1199	1.6552	0.0042	0.0296
Cxcl16	0.2785	0.3620	0.9995	1.8695	0.0000	0.0013	1.6516	0.0000	0.0010
Ptprc	-0.8704	0.4884	0.9995	4.0834	0.0012	0.0205	1.6484	0.0980	0.2324
Btc	-0.3279	0.5146	0.9995	1.4680	0.0053	0.0474	1.6469	0.0004	0.0065
Mphosph10	0.4242	0.1835	0.9995	1.1620	0.0009	0.0182	1.6468	0.0000	0.0011
Tspan2	-0.6360	0.2167	0.9995	1.9958	0.0003	0.0111	1.6463	0.0004	0.0063
Slc25a33	0.5503	0.3932	0.9995	1.5107	0.0083	0.0613	1.6459	0.0084	0.0467
Nop2	0.2342	0.3965	0.9995	1.6479	0.0000	0.0013	1.6433	0.0000	0.0004
Parp12	-0.1260	0.7232	0.9995	0.6704	0.0629	0.2059	1.6389	0.0000	0.0018
Rap1gap2	0.4764	0.4436	0.9995	0.8592	0.1026	0.2785	1.6374	0.0079	0.0452
Pola2	0.7217	0.2445	0.9995	0.9416	0.0913	0.2600	1.6310	0.0085	0.0470
Slc20a1	0.6731	0.0251	0.9995	0.9610	0.0023	0.0286	1.6288	0.0000	0.0007
Gm2436	-0.5690	0.5708	0.9995	2.8899	0.0028	0.0321	1.6283	0.0521	0.1531
Gm2446	-0.5690	0.5708	0.9995	2.8899	0.0028	0.0321	1.6283	0.0521	0.1531
Ier5	0.6907	0.0322	0.9995	1.3467	0.0001	0.0059	1.6269	0.0000	0.0010
Recql4	-0.6751	0.5697	0.9995	3.4819	0.0025	0.0306	1.6248	0.0870	0.2146
Chchd4	0.5816	0.1956	0.9995	1.1548	0.0060	0.0505	1.6208	0.0006	0.0086
Mob3c	0.7175	0.2566	0.9995	0.9772	0.0577	0.1965	1.6203	0.0079	0.0453
1810055G02Rik	0.4640	0.2106	0.9995	0.7700	0.0248	0.1187	1.6136	0.0001	0.0027
Zc3h8	0.4511	0.2964	0.9995	1.5477	0.0005	0.0130	1.6116	0.0003	0.0062
Tap1	-0.1606	0.6301	0.9995	-0.3335	0.3429	0.5688	1.6104	0.0000	0.0011
Traip	0.1699	0.7063	0.9995	1.3334	0.0042	0.0412	1.6093	0.0005	0.0078
Nip7	0.2961	0.5064	0.9995	1.2776	0.0049	0.0454	1.6092	0.0007	0.0096
4930453N24Rik	0.3729	0.3323	0.9995	1.0445	0.0062	0.0517	1.6072	0.0001	0.0037
Dclre1a	0.2141	0.6513	0.9995	1.1917	0.0104	0.0705	1.6064	0.0011	0.0124

Ppm1h	0.1275	0.8482	0.9995	1.1760	0.0526	0.1864	1.6016	0.0082	0.0461
Cxcl5	3.1002	0.0000	0.1219	0.7896	0.1532	0.3544	1.6002	0.0060	0.0375
Ublcp1	0.0990	0.8246	0.9995	1.3670	0.0030	0.0336	1.5992	0.0006	0.0084
Orai1	0.5859	0.1904	0.9995	0.8364	0.0412	0.1610	1.5987	0.0006	0.0083
Daxx	-0.1584	0.6063	0.9995	0.8602	0.0095	0.0663	1.5980	0.0000	0.0010
Ccne1	0.1627	0.7345	0.9995	2.1065	0.0001	0.0048	1.5965	0.0008	0.0100
Fam118b	-0.1258	0.8154	0.9995	1.6359	0.0025	0.0303	1.5963	0.0021	0.0191
Slfn2	0.2033	0.5471	0.9995	1.1934	0.0013	0.0217	1.5960	0.0000	0.0019
Ehf	2.5938	0.0000	0.0727	1.0076	0.0012	0.0207	1.5950	0.0009	0.0111
Palb2	0.6591	0.2900	0.9995	1.4369	0.0083	0.0610	1.5923	0.0076	0.0437
Mylk	-0.1146	0.8427	0.9995	1.7050	0.0038	0.0387	1.5905	0.0030	0.0235
Spn	0.6086	0.1638	0.9995	1.2644	0.0029	0.0331	1.5890	0.0005	0.0077
Abcc5	0.4632	0.2877	0.9995	0.7592	0.0561	0.1938	1.5876	0.0006	0.0084
Cd40	0.5553	0.3826	0.9995	1.6463	0.0038	0.0387	1.5857	0.0084	0.0467
E130309D02Rik	0.1198	0.7518	0.9995	0.8802	0.0184	0.0984	1.5842	0.0001	0.0028
Slfn8	-0.1305	0.6061	0.9995	0.7196	0.0081	0.0605	1.5838	0.0000	0.0002
Spink10	-0.1191	0.8605	0.9995	1.2626	0.0428	0.1648	1.5833	0.0084	0.0468
Macrod2	-0.2875	0.6216	0.9995	1.5178	0.0081	0.0605	1.5832	0.0023	0.0200
Dennd5b	-0.0087	0.9902	0.9995	2.0638	0.0024	0.0299	1.5814	0.0168	0.0732
Exo1	0.3760	0.4129	0.9995	0.8427	0.0468	0.1737	1.5781	0.0010	0.0116
Map4k1	-0.0799	0.9012	0.9995	1.1265	0.0674	0.2137	1.5777	0.0049	0.0329
Gbp9	-0.0642	0.8763	0.9995	0.4988	0.2282	0.4504	1.5772	0.0003	0.0056
Bysl	0.1536	0.6983	0.9995	2.0236	0.0000	0.0026	1.5771	0.0002	0.0046
D6Wsu163e	-0.0571	0.9240	0.9995	1.1597	0.0391	0.1559	1.5750	0.0048	0.0320
Fas	1.0073	0.0459	0.9995	0.8251	0.0436	0.1665	1.5735	0.0019	0.0176
Eef1e1	0.6443	0.1812	0.9995	0.8871	0.0403	0.1588	1.5724	0.0013	0.0140
Aars	0.0298	0.9215	0.9995	1.4118	0.0002	0.0070	1.5715	0.0000	0.0012
Orc1	0.3059	0.4226	0.9995	1.2539	0.0011	0.0195	1.5697	0.0001	0.0037
Ifih1	-0.2999	0.3577	0.9995	0.6293	0.0599	0.2007	1.5664	0.0000	0.0012
Tsen15	0.4715	0.3459	0.9995	0.8179	0.0686	0.2159	1.5626	0.0022	0.0197
D030056L22Rik	0.6211	0.1071	0.9995	1.0748	0.0054	0.0478	1.5603	0.0002	0.0050
Mak16	0.3511	0.1421	0.9995	1.1934	0.0000	0.0037	1.5596	0.0000	0.0002
Tedc2	0.3271	0.4308	0.9995	0.9223	0.0184	0.0986	1.5585	0.0003	0.0058
Plcxd2	-0.0542	0.8948	0.9995	1.8023	0.0001	0.0061	1.5571	0.0002	0.0044
Slfn3	0.3361	0.3836	0.9995	1.2532	0.0017	0.0245	1.5553	0.0002	0.0041
Slc35a2	0.3002	0.5875	0.9995	0.9009	0.0647	0.2088	1.5494	0.0046	0.0315
Prl7a2	-1.3228	0.3315	0.9995	3.9759	0.0028	0.0326	1.5487	0.1696	0.3278
Trak2	0.6880	0.1188	0.9995	0.8168	0.0385	0.1541	1.5460	0.0008	0.0104
Ifrd1	0.5438	0.0367	0.9995	0.8516	0.0021	0.0270	1.5435	0.0000	0.0004
Fam220a	0.7634	0.0761	0.9995	0.6729	0.0672	0.2132	1.5361	0.0006	0.0089
Gtf2h1	0.3611	0.1116	0.9995	1.1440	0.0000	0.0032	1.5357	0.0000	0.0001
Atg4a	-0.1758	0.7115	0.9995	1.6424	0.0010	0.0186	1.5352	0.0007	0.0098
Parp10	0.6820	0.0734	0.9995	-0.1394	0.6836	0.8273	1.5351	0.0002	0.0040
Inip	0.0829	0.8282	0.9995	1.6731	0.0001	0.0067	1.5346	0.0002	0.0041
Swsap1	0.7684	0.1802	0.9995	0.6087	0.2025	0.4182	1.5337	0.0057	0.0364
Nop9	0.6530	0.2328	0.9995	1.1999	0.0146	0.0864	1.5328	0.0052	0.0340
Cpeb2	-0.2568	0.4691	0.9995	2.0376	0.0000	0.0013	1.5327	0.0000	0.0019
Orc2	0.2494	0.4239	0.9995	1.2908	0.0003	0.0109	1.5323	0.0000	0.0015
Pgam5	0.2948	0.4026	0.9995	1.0072	0.0059	0.0504	1.5322	0.0001	0.0034
Trafd1	0.0098	0.9790	0.9995	0.8167	0.0322	0.1386	1.5305	0.0002	0.0044
Dynap	0.2756	0.5277	0.9995	1.1563	0.0124	0.0787	1.5295	0.0011	0.0128

Rassf1	-0.1933	0.5775	0.9995	1.4206	0.0005	0.0127	1.5294	0.0001	0.0028
Mrm3	0.0508	0.9072	0.9995	1.5068	0.0010	0.0191	1.5263	0.0005	0.0079
A630089N07Rik	-0.3930	0.3079	0.9995	1.4077	0.0010	0.0190	1.5254	0.0001	0.0037
Ddx60	-0.1499	0.5800	0.9995	0.0069	0.9799	0.9912	1.5210	0.0000	0.0005
Mcm10	0.0425	0.8772	0.9995	1.1392	0.0004	0.0121	1.5165	0.0000	0.0007
Myl7	0.0948	0.9487	0.9995	4.6282	0.0010	0.0184	1.5145	0.2588	0.4319
Rad51	0.3383	0.1993	0.9995	1.3795	0.0000	0.0023	1.5144	0.0000	0.0005
Polr2g	0.3576	0.2158	0.9995	0.9891	0.0018	0.0252	1.5138	0.0000	0.0011
Bop1	0.3089	0.5075	0.9995	1.4870	0.0018	0.0251	1.5114	0.0013	0.0142
Ulbp1	0.6386	0.2861	0.9995	0.5245	0.3246	0.5504	1.5081	0.0076	0.0439
Ippk	-0.2309	0.7042	0.9995	1.3417	0.0208	0.1061	1.5043	0.0065	0.0395
C1ra	0.9185	0.0723	0.9995	0.7352	0.0867	0.2518	1.5031	0.0033	0.0250
Ccdc86	0.3859	0.2477	0.9995	1.2420	0.0007	0.0153	1.5012	0.0001	0.0025
Pbp2	-1.6144	0.2239	0.9995	3.8564	0.0036	0.0375	1.4965	0.1231	0.2685
Heatr3	0.4560	0.3139	0.9995	0.6901	0.0986	0.2720	1.4937	0.0014	0.0151
Wdhd1	0.3904	0.2957	0.9995	1.0679	0.0039	0.0394	1.4928	0.0002	0.0046
Lrrc59	0.0038	0.9942	0.9995	1.8334	0.0011	0.0198	1.4927	0.0031	0.0239
Riok2	0.0801	0.8811	0.9995	1.2934	0.0133	0.0819	1.4918	0.0046	0.0315
Parp14	-0.3955	0.1432	0.9995	0.7493	0.0094	0.0660	1.4910	0.0000	0.0006
Nxt1	0.5644	0.2386	0.9995	1.0013	0.0206	0.1054	1.4894	0.0023	0.0200
Hey1	-2.7441	0.0775	0.9995	4.5383	0.0032	0.0351	1.4889	0.1728	0.3316
Wisp1	-0.3627	0.4865	0.9995	1.5871	0.0042	0.0410	1.4889	0.0033	0.0253
Dusp4	0.1182	0.8356	0.9995	1.2853	0.0252	0.1196	1.4885	0.0071	0.0417
Arhgap1	0.3928	0.2123	0.9995	0.8287	0.0088	0.0634	1.4870	0.0000	0.0018
Parp11	0.3128	0.5102	0.9995	-0.1301	0.7741	0.8826	1.4865	0.0018	0.0173
Pim2	2.2356	0.0036	0.9995	1.2417	0.0052	0.0466	1.4811	0.0390	0.1256
L3mbtl2	0.0730	0.8612	0.9995	1.2069	0.0049	0.0452	1.4798	0.0005	0.0080
Trim11	0.6272	0.2283	0.9995	0.9601	0.0436	0.1665	1.4792	0.0047	0.0318
Alg13	-0.2286	0.6855	0.9995	1.6348	0.0037	0.0383	1.4746	0.0032	0.0244
Abhd10	0.2406	0.6217	0.9995	0.9946	0.0288	0.1290	1.4715	0.0019	0.0177
Psmb8	0.3145	0.2338	0.9995	-0.2494	0.3386	0.5645	1.4711	0.0000	0.0007
Asf1b	0.2050	0.4766	0.9995	1.3495	0.0001	0.0055	1.4704	0.0000	0.0012
Clu	-0.7738	0.0092	0.9995	2.4089	0.0000	0.0001	1.4700	0.0000	0.0009
Tmed5	0.1882	0.4181	0.9995	1.4457	0.0000	0.0012	1.4683	0.0000	0.0003
Slbp	0.3805	0.3944	0.9995	0.9829	0.0292	0.1297	1.4679	0.0019	0.0176
Spata2	0.3075	0.2800	0.9995	1.1235	0.0004	0.0116	1.4674	0.0000	0.0011
Ogfod1	0.0732	0.8442	0.9995	1.4053	0.0005	0.0134	1.4638	0.0002	0.0049
Fem1a	0.1764	0.6496	0.9995	1.1084	0.0061	0.0510	1.4635	0.0004	0.0068
Psmg3	0.5638	0.1681	0.9995	0.8607	0.0286	0.1285	1.4603	0.0008	0.0102
Urb2	-0.0047	0.9910	0.9995	1.3947	0.0014	0.0221	1.4589	0.0006	0.0084
Uqcrq	0.1725	0.4284	0.9995	1.4726	0.0000	0.0008	1.4562	0.0000	0.0002
Cdc42bpg	0.2278	0.6199	0.9995	0.8800	0.0522	0.1857	1.4536	0.0023	0.0202
Sod2	0.6481	0.1939	0.9995	1.1761	0.0137	0.0831	1.4511	0.0042	0.0294
2700038G22Rik	-0.1652	0.7426	0.9995	2.1790	0.0001	0.0049	1.4510	0.0021	0.0186
Cgas	0.2617	0.4030	0.9995	0.7860	0.0131	0.0810	1.4485	0.0001	0.0020
Dck	0.3372	0.4696	0.9995	1.1110	0.0137	0.0832	1.4485	0.0023	0.0200
Rdh13	-0.0610	0.9048	0.9995	1.4275	0.0057	0.0495	1.4476	0.0022	0.0194
Lins1	0.5162	0.2893	0.9995	0.5836	0.1602	0.3654	1.4465	0.0027	0.0224
Prrg4	0.0836	0.7950	0.9995	0.7236	0.0263	0.1226	1.4449	0.0001	0.0022
Samd4b	0.2049	0.5791	0.9995	0.7557	0.0459	0.1717	1.4448	0.0005	0.0072
9130008F23Rik	-0.5798	0.2153	0.9995	2.3398	0.0000	0.0027	1.4448	0.0006	0.0086

Mocs2	0.6270	0.0511	0.9995	1.0238	0.0011	0.0196	1.4445	0.0001	0.0020
Herpud1	0.3100	0.4179	0.9995	1.3160	0.0011	0.0195	1.4444	0.0004	0.0072
Tmem18	0.2848	0.5056	0.9995	1.4375	0.0008	0.0171	1.4427	0.0011	0.0122
Nup85	0.6190	0.1795	0.9995	0.5047	0.2456	0.4702	1.4415	0.0026	0.0217
Amotl2	-0.1353	0.6838	0.9995	1.4929	0.0001	0.0069	1.4414	0.0001	0.0030
Ampd2	0.1974	0.6491	0.9995	1.5811	0.0006	0.0137	1.4408	0.0013	0.0139
Plpbp	0.2000	0.5792	0.9995	1.0297	0.0051	0.0461	1.4403	0.0002	0.0051
Pde12	0.6831	0.1123	0.9995	0.7913	0.0357	0.1472	1.4382	0.0012	0.0131
Tmem69	0.1973	0.7046	0.9995	1.1125	0.0198	0.1028	1.4359	0.0040	0.0285
Pura	0.5629	0.2520	0.9995	-0.0110	0.9813	0.9916	1.4359	0.0046	0.0314
Umps	0.3345	0.2365	0.9995	1.0025	0.0011	0.0192	1.4350	0.0000	0.0011
Enoph1	0.2661	0.5614	0.9995	1.2778	0.0040	0.0403	1.4340	0.0020	0.0184
Pdia5	0.8559	0.0714	0.9995	0.5361	0.1709	0.3783	1.4322	0.0025	0.0213
Timm8a1	0.5053	0.0934	0.9995	1.0160	0.0014	0.0219	1.4320	0.0000	0.0017
Rbm34	0.4072	0.2833	0.9995	0.8129	0.0319	0.1374	1.4315	0.0005	0.0081
Ccne2	0.2403	0.6162	0.9995	1.3422	0.0063	0.0518	1.4302	0.0027	0.0224
Zfp296	0.1832	0.7469	0.9995	1.8675	0.0007	0.0152	1.4259	0.0082	0.0459
Gm8995	0.1566	0.5777	0.9995	1.0681	0.0008	0.0162	1.4244	0.0000	0.0012
Mndal	0.5212	0.1374	0.9995	-0.0197	0.9534	0.9811	1.4236	0.0003	0.0057
Gen1	-0.2871	0.4800	0.9995	1.4589	0.0010	0.0191	1.4231	0.0004	0.0069
Rexo2	0.0660	0.7893	0.9995	1.6337	0.0000	0.0008	1.4223	0.0000	0.0005
Nup35	0.0088	0.9826	0.9995	1.4077	0.0013	0.0210	1.4212	0.0007	0.0098
Sh3d21	0.3183	0.4568	0.9995	0.4996	0.2161	0.4360	1.4192	0.0011	0.0129
Clptm1l	0.2280	0.6191	0.9995	1.2437	0.0072	0.0563	1.4167	0.0025	0.0213
Ptgs1	-0.0030	0.9935	0.9995	1.2598	0.0014	0.0219	1.4161	0.0003	0.0055
Dgke	-0.2894	0.6301	0.9995	1.8283	0.0028	0.0324	1.4159	0.0065	0.0393
Rbm4	0.3520	0.3094	0.9995	0.7924	0.0200	0.1036	1.4132	0.0002	0.0045
Hyou1	0.2708	0.3160	0.9995	1.3909	0.0000	0.0025	1.4130	0.0000	0.0010
Rad51ap1	0.1479	0.6455	0.9995	1.1446	0.0014	0.0218	1.4092	0.0001	0.0030
Arpc5l	0.3005	0.3818	0.9995	0.8682	0.0159	0.0902	1.4086	0.0003	0.0056
Gbp2	0.5447	0.0778	0.9995	-0.4947	0.1043	0.2807	1.4075	0.0001	0.0021
Nkapd1	0.1831	0.5964	0.9995	0.5165	0.1299	0.3201	1.4041	0.0003	0.0052
Sgk1	-0.3867	0.2956	0.9995	1.7877	0.0001	0.0041	1.4037	0.0002	0.0044
Exosc1	0.3547	0.1686	0.9995	1.2474	0.0001	0.0040	1.4031	0.0000	0.0007
Frg2f1	0.0989	0.9261	0.9995	2.6645	0.0055	0.0485	1.4013	0.1375	0.2870
Zfp948	-0.4080	0.2398	0.9995	1.4690	0.0002	0.0086	1.3998	0.0001	0.0027
Dhfr	0.1526	0.6232	0.9995	1.3379	0.0002	0.0086	1.3988	0.0001	0.0026
Mgme1	-0.4536	0.2328	0.9995	1.4980	0.0004	0.0121	1.3987	0.0002	0.0050
Odc1	0.6932	0.0481	0.9995	0.7287	0.0371	0.1504	1.3968	0.0003	0.0055
Surf6	0.1593	0.6273	0.9995	1.4857	0.0001	0.0055	1.3968	0.0001	0.0033
Ifit2	0.1742	0.6550	0.9995	0.5424	0.1551	0.3575	1.3964	0.0005	0.0081
Cfap44	0.0948	0.9501	0.9995	4.6418	0.0011	0.0200	1.3954	0.3106	0.4864
Relb	0.1873	0.6399	0.9995	0.7067	0.0668	0.2127	1.3915	0.0008	0.0104
Wdr74	0.3540	0.2533	0.9995	1.0663	0.0013	0.0209	1.3913	0.0001	0.0025
Slc3a2	0.3731	0.4164	0.9995	1.0631	0.0241	0.1165	1.3911	0.0037	0.0269
C1qtnf12	-0.6432	0.5730	0.9995	3.3722	0.0020	0.0263	1.3890	0.1329	0.2811
Serpinb9	-0.1547	0.6097	0.9995	1.3850	0.0001	0.0055	1.3860	0.0000	0.0016
Atp1b1	0.0062	0.9836	0.9995	1.2061	0.0005	0.0123	1.3842	0.0001	0.0021
Usp27x	0.6204	0.1253	0.9995	0.7077	0.0515	0.1839	1.3835	0.0009	0.0108
Nts	-0.7103	0.4705	0.9995	3.2053	0.0011	0.0198	1.3834	0.0822	0.2071
Slc30a1	0.7284	0.1644	0.9995	0.4440	0.3203	0.5465	1.3815	0.0072	0.0423

Lurap1l	0.6526	0.0706	0.9995	0.0156	0.9614	0.9844	1.3812	0.0003	0.0055
Pop5	-0.0940	0.7940	0.9995	1.2942	0.0012	0.0206	1.3774	0.0003	0.0056
Atg3	0.2722	0.4069	0.9995	0.8386	0.0106	0.0712	1.3715	0.0002	0.0039
Il24	1.3009	0.0006	0.7314	0.9672	0.0017	0.0242	1.3711	0.0002	0.0046
Ptcd3	0.5343	0.2229	0.9995	0.6707	0.1065	0.2847	1.3693	0.0025	0.0214
Gm8989	0.1201	0.7410	0.9995	0.3807	0.2794	0.5061	1.3686	0.0004	0.0065
Sf3b4	0.2943	0.5276	0.9995	1.0466	0.0247	0.1184	1.3681	0.0041	0.0292
Tbc1d14	0.7179	0.1389	0.9995	0.3856	0.3453	0.5711	1.3681	0.0049	0.0326
Kitl	-1.2120	0.0049	0.9995	2.2042	0.0000	0.0018	1.3671	0.0001	0.0032
Flywch2	0.0828	0.7697	0.9995	1.1229	0.0004	0.0118	1.3669	0.0000	0.0015
Ythdf1	0.3073	0.3960	0.9995	0.9490	0.0076	0.0580	1.3665	0.0004	0.0066
Mosmo	0.0346	0.9186	0.9995	1.4962	0.0001	0.0066	1.3649	0.0002	0.0041
Ccdc141	-0.5226	0.3485	0.9995	1.7123	0.0029	0.0332	1.3642	0.0039	0.0279
1700123O20Rik	0.2464	0.5147	0.9995	1.0274	0.0063	0.0521	1.3641	0.0006	0.0084
Mfsd11	0.0801	0.8470	0.9995	1.0767	0.0103	0.0703	1.3623	0.0013	0.0139
Psmd8	0.4397	0.1226	0.9995	0.7872	0.0083	0.0611	1.3618	0.0000	0.0019
Ppa1	0.4899	0.0369	0.9995	0.9449	0.0003	0.0107	1.3600	0.0000	0.0004
Ddx21	0.1643	0.5950	0.9995	1.1802	0.0012	0.0201	1.3564	0.0002	0.0042
Gm20324	-2.0188	0.1104	0.9995	3.8869	0.0023	0.0291	1.3561	0.0899	0.2199
Gm8979	0.1340	0.6957	0.9995	0.3885	0.2439	0.4684	1.3539	0.0002	0.0050
Nolc1	0.1492	0.6740	0.9995	1.2695	0.0019	0.0258	1.3524	0.0007	0.0091
Msrb3	-0.2632	0.6321	0.9995	1.1790	0.0263	0.1226	1.3518	0.0074	0.0429
Nadk	-0.0342	0.9221	0.9995	1.5384	0.0002	0.0088	1.3505	0.0004	0.0072
Tlr3	-0.2613	0.2583	0.9995	0.6186	0.0118	0.0764	1.3496	0.0000	0.0004
Cherp	0.5868	0.0395	0.9995	0.6120	0.0209	0.1064	1.3491	0.0000	0.0015
Zfp598	0.4061	0.3229	0.9995	0.7903	0.0484	0.1770	1.3462	0.0018	0.0171
D11Wsu47e	0.3128	0.4967	0.9995	0.7347	0.0931	0.2631	1.3451	0.0025	0.0212
Pnp	0.2601	0.4203	0.9995	0.8508	0.0100	0.0685	1.3451	0.0002	0.0042
Ifi211	-0.0830	0.8144	0.9995	0.7433	0.0417	0.1619	1.3443	0.0004	0.0066
Rsl1d1	0.6086	0.0747	0.9995	0.6097	0.0713	0.2219	1.3431	0.0003	0.0062
Neurl3	1.2106	0.0005	0.6730	0.7444	0.0070	0.0554	1.3419	0.0001	0.0026
Grwd1	0.3261	0.3682	0.9995	1.2677	0.0011	0.0192	1.3393	0.0005	0.0079
Med22	-0.1358	0.6636	0.9995	1.7926	0.0000	0.0016	1.3382	0.0001	0.0027
Ppp1r2	0.1191	0.7157	0.9995	1.2069	0.0012	0.0201	1.3369	0.0003	0.0056
Serpine1	-0.0532	0.9174	0.9995	1.9667	0.0005	0.0135	1.3363	0.0080	0.0454
Rrs1	0.4728	0.1970	0.9995	0.9207	0.0128	0.0803	1.3347	0.0007	0.0093
Ifi30	0.3249	0.4910	0.9995	0.4937	0.2790	0.5057	1.3337	0.0058	0.0365
Rrm2	0.1386	0.8014	0.9995	1.4813	0.0044	0.0421	1.3329	0.0090	0.0487
Fbxo45	0.3760	0.3308	0.9995	1.0592	0.0061	0.0512	1.3321	0.0010	0.0121
Trib3	-0.6195	0.0919	0.9995	1.9253	0.0000	0.0024	1.3304	0.0002	0.0046
Yy2	-0.0302	0.9490	0.9995	1.5478	0.0015	0.0231	1.3277	0.0028	0.0228
Plscr2	0.5858	0.0929	0.9995	0.7596	0.0250	0.1190	1.3267	0.0004	0.0070
Itgb1bp1	0.5978	0.0648	0.9995	0.9404	0.0027	0.0317	1.3239	0.0002	0.0039
Otud6b	0.4123	0.2178	0.9995	0.9383	0.0067	0.0541	1.3236	0.0003	0.0058
Abcf2	-0.2059	0.5577	0.9995	1.6675	0.0001	0.0062	1.3211	0.0005	0.0080
Gltp	0.1038	0.8172	0.9995	0.9483	0.0344	0.1440	1.3194	0.0037	0.0270
Ifitm3	-0.3108	0.2484	0.9995	0.9864	0.0015	0.0227	1.3166	0.0000	0.0019
Rnf213	-0.1038	0.6536	0.9995	0.7046	0.0057	0.0494	1.3153	0.0000	0.0006
Smim10l1	0.0784	0.8363	0.9995	0.7763	0.0433	0.1660	1.3147	0.0011	0.0124
Fbn1	-1.3879	0.1202	0.9995	2.6527	0.0038	0.0389	1.3100	0.0350	0.1166
Med10	0.5322	0.1695	0.9995	0.7288	0.0474	0.1747	1.3097	0.0012	0.0136

Hspa14	0.1460	0.6797	0.9995	0.8832	0.0178	0.0965	1.3096	0.0007	0.0098
Pcsk6	0.6797	0.1806	0.9995	0.3015	0.4897	0.6937	1.3084	0.0081	0.0457
Vps37b	0.4944	0.0592	0.9995	0.9723	0.0005	0.0124	1.3028	0.0000	0.0011
Enpp4	0.2593	0.4685	0.9995	0.5823	0.0938	0.2642	1.3024	0.0006	0.0089
Wdr55	0.7359	0.1169	0.9995	0.3113	0.4253	0.6428	1.3017	0.0053	0.0344
Vac14	0.2355	0.5987	0.9995	0.5703	0.1757	0.3850	1.3016	0.0033	0.0251
Tor1aip1	0.4225	0.2368	0.9995	-0.1534	0.6578	0.8112	1.3005	0.0008	0.0104
Casp8ap2	0.0465	0.8689	0.9995	0.8562	0.0058	0.0497	1.3005	0.0001	0.0026
Enc1	0.1309	0.6943	0.9995	0.8518	0.0156	0.0895	1.3004	0.0004	0.0072
Tcf19	-0.2568	0.5104	0.9995	1.9592	0.0000	0.0030	1.2978	0.0009	0.0109
Mrps14	0.4445	0.3026	0.9995	0.8853	0.0338	0.1424	1.2966	0.0034	0.0254
Mfsd1	0.4495	0.1464	0.9995	0.3031	0.3111	0.5374	1.2957	0.0002	0.0046
Tor1aip2	0.4892	0.1282	0.9995	0.3294	0.2851	0.5121	1.2949	0.0003	0.0057
Ndc1	0.1066	0.8177	0.9995	1.0402	0.0189	0.0999	1.2944	0.0045	0.0308
Gtf2e1	0.1476	0.6762	0.9995	0.9110	0.0103	0.0699	1.2933	0.0004	0.0071
Cebpg	0.0403	0.9021	0.9995	1.0217	0.0040	0.0401	1.2909	0.0003	0.0062
Cyp1b1	-0.2129	0.6422	0.9995	1.5436	0.0015	0.0226	1.2908	0.0030	0.0233
D1Ertd622e	0.2185	0.5213	0.9995	1.0622	0.0026	0.0308	1.2886	0.0004	0.0065
Atf5	-0.4928	0.4075	0.9995	1.8743	0.0032	0.0351	1.2879	0.0141	0.0658
Smcr8	-0.2701	0.5379	0.9995	1.2028	0.0085	0.0623	1.2866	0.0019	0.0176
Rmdn3	0.2149	0.5260	0.9995	1.0471	0.0039	0.0393	1.2850	0.0004	0.0072
Tusc2	0.0869	0.7641	0.9995	1.1203	0.0006	0.0140	1.2849	0.0001	0.0025
Ifi203	0.5179	0.2245	0.9995	-0.1479	0.7218	0.8507	1.2846	0.0039	0.0279
Atpaf2	0.2148	0.5758	0.9995	0.7868	0.0343	0.1437	1.2802	0.0011	0.0127
Ppm1f	0.0246	0.9495	0.9995	1.7395	0.0001	0.0054	1.2767	0.0010	0.0120
Adgre5	-0.5418	0.6709	0.9995	3.4141	0.0038	0.0385	1.2755	0.2260	0.3947
Mad2l1bp	0.2150	0.5389	0.9995	0.9698	0.0060	0.0506	1.2747	0.0006	0.0084
Limk1	0.4536	0.2500	0.9995	0.7476	0.0433	0.1660	1.2745	0.0013	0.0143
Dolk	0.1852	0.7025	0.9995	1.0874	0.0176	0.0961	1.2738	0.0064	0.0391
C2cd2l	-0.1126	0.8336	0.9995	1.5176	0.0049	0.0454	1.2738	0.0078	0.0446
Kifc3	0.0605	0.9001	0.9995	0.7753	0.1010	0.2762	1.2710	0.0077	0.0444
Prkx	0.1123	0.8086	0.9995	0.9377	0.0413	0.1610	1.2700	0.0062	0.0384
Ccdc117	0.5448	0.1204	0.9995	0.9126	0.0064	0.0526	1.2687	0.0006	0.0084
Sgf29	-0.0833	0.8680	0.9995	1.4798	0.0044	0.0426	1.2676	0.0051	0.0337
Ak2	0.3363	0.1207	0.9995	1.1750	0.0000	0.0024	1.2666	0.0000	0.0005
Nol8	0.1157	0.6819	0.9995	1.0424	0.0013	0.0212	1.2654	0.0001	0.0032
Grpel2	0.0321	0.9377	0.9995	1.6531	0.0003	0.0094	1.2651	0.0019	0.0176
Sfxn2	0.1588	0.6395	0.9995	0.7634	0.0258	0.1212	1.2639	0.0004	0.0071
Uchl3	0.4831	0.2926	0.9995	0.8640	0.0538	0.1891	1.2620	0.0067	0.0402
Mettl13	0.5263	0.1429	0.9995	0.9235	0.0073	0.0565	1.2611	0.0007	0.0095
Asb1	0.4346	0.3704	0.9995	0.5325	0.2142	0.4337	1.2598	0.0068	0.0404
Ifi203-ps	0.5110	0.2682	0.9995	-0.0459	0.9165	0.9618	1.2596	0.0070	0.0413
Polr3h	0.1797	0.6899	0.9995	1.0538	0.0200	0.1037	1.2595	0.0042	0.0296
Asna1	-0.0623	0.8794	0.9995	1.4150	0.0014	0.0223	1.2591	0.0019	0.0177
Emc7	0.1701	0.6534	0.9995	0.8940	0.0207	0.1056	1.2590	0.0015	0.0159
Pnpla7	-0.1453	0.7572	0.9995	0.0510	0.9145	0.9609	1.2587	0.0038	0.0277
Cdc45	0.2043	0.5499	0.9995	1.2360	0.0009	0.0179	1.2586	0.0006	0.0085
Yae1d1	0.4323	0.3192	0.9995	0.6411	0.1043	0.2807	1.2573	0.0039	0.0280
2410002F23Rik	-0.1748	0.6793	0.9995	1.4633	0.0015	0.0231	1.2549	0.0020	0.0181
Pop4	0.8113	0.0653	0.9995	0.9142	0.0150	0.0878	1.2542	0.0037	0.0271
Dclre1c	0.0715	0.8031	0.9995	0.9847	0.0017	0.0241	1.2537	0.0001	0.0030

Tsr1	0.3993	0.1748	0.9995	0.8547	0.0052	0.0468	1.2514	0.0001	0.0036
Cdc42se2	0.5459	0.1020	0.9995	0.6459	0.0397	0.1573	1.2509	0.0005	0.0072
Phlda1	0.6876	0.0166	0.9995	0.6982	0.0123	0.0785	1.2479	0.0001	0.0026
Ska3	-0.0886	0.8400	0.9995	0.7873	0.0671	0.2131	1.2468	0.0037	0.0269
Rpa2	-0.0868	0.8153	0.9995	1.1033	0.0069	0.0549	1.2465	0.0016	0.0162
4833438C02Rik	-0.0177	0.9686	0.9995	1.2355	0.0070	0.0551	1.2450	0.0035	0.0260
Hells	0.2656	0.2253	0.9995	0.7754	0.0019	0.0255	1.2448	0.0000	0.0007
Znfx1	0.1379	0.6199	0.9995	0.4247	0.1278	0.3171	1.2441	0.0001	0.0028
C3	0.9863	0.0022	0.9995	1.3069	0.0001	0.0064	1.2439	0.0002	0.0039
Caml	0.0918	0.8157	0.9995	1.1589	0.0040	0.0401	1.2433	0.0018	0.0173
Zfp933	0.0825	0.8627	0.9995	0.9912	0.0311	0.1353	1.2431	0.0055	0.0355
Dcp2	-0.1992	0.4779	0.9995	1.1504	0.0004	0.0115	1.2422	0.0001	0.0022
Vma21	0.2866	0.3775	0.9995	0.9542	0.0053	0.0472	1.2410	0.0005	0.0075
2310007B03Rik	-0.3710	0.3683	0.9995	1.3491	0.0018	0.0255	1.2403	0.0013	0.0142
Plekha3	0.0905	0.8006	0.9995	1.0902	0.0046	0.0433	1.2401	0.0011	0.0122
Uros	-3.2565	0.0399	0.9995	4.9335	0.0019	0.0257	1.2390	0.2317	0.4017
Nfkbia	1.0617	0.0019	0.9995	0.3981	0.1779	0.3882	1.2390	0.0003	0.0054
Procr	0.7174	0.0157	0.9995	0.5026	0.0644	0.2084	1.2387	0.0001	0.0033
Rmi2	0.0736	0.8542	0.9995	1.3929	0.0011	0.0192	1.2360	0.0020	0.0184
Tor4a	0.7334	0.1017	0.9995	0.0950	0.8112	0.9040	1.2340	0.0059	0.0369
Cdipt	0.2362	0.4952	0.9995	0.9860	0.0062	0.0517	1.2326	0.0008	0.0100
Cenph	0.3046	0.1746	0.9995	0.7458	0.0023	0.0291	1.2324	0.0000	0.0008
Mthfd2	-0.5233	0.2633	0.9995	2.0112	0.0003	0.0104	1.2305	0.0074	0.0431
Srprb	0.1259	0.6546	0.9995	0.9405	0.0018	0.0251	1.2298	0.0001	0.0028
Arl14ep	0.1264	0.6832	0.9995	0.7811	0.0151	0.0880	1.2292	0.0003	0.0059
Mbd1	0.2112	0.5801	0.9995	0.7418	0.0461	0.1723	1.2287	0.0016	0.0161
Tcof1	0.3330	0.2487	0.9995	0.4972	0.0875	0.2532	1.2276	0.0002	0.0045
AW209491	0.2442	0.5501	0.9995	1.2043	0.0032	0.0348	1.2263	0.0030	0.0233
Coq5	0.4835	0.1971	0.9995	0.5823	0.1113	0.2920	1.2250	0.0019	0.0177
Pms2	0.4011	0.1745	0.9995	1.0345	0.0009	0.0174	1.2247	0.0002	0.0039
Rom1	-0.3479	0.4537	0.9995	1.2501	0.0087	0.0628	1.2242	0.0032	0.0247
Cisd1	0.3965	0.1134	0.9995	0.9747	0.0005	0.0123	1.2240	0.0000	0.0015
Naa20	0.2019	0.6373	0.9995	0.4518	0.2780	0.5047	1.2224	0.0047	0.0320
Zgrf1	0.3857	0.3034	0.9995	0.6000	0.0895	0.2567	1.2217	0.0016	0.0161
Nup93	0.5399	0.1865	0.9995	0.5391	0.1538	0.3556	1.2198	0.0034	0.0254
Dnajb11	0.4243	0.1804	0.9995	0.8831	0.0056	0.0490	1.2191	0.0004	0.0065
Rbm43	0.1684	0.6024	0.9995	0.7165	0.0256	0.1208	1.2162	0.0004	0.0070
Nop56	-0.0395	0.8995	0.9995	1.0480	0.0034	0.0360	1.2156	0.0006	0.0084
Pptc7	0.8225	0.0352	0.9995	0.1362	0.7015	0.8394	1.2092	0.0022	0.0197
Ddx51	0.2770	0.5498	0.9995	1.1218	0.0122	0.0778	1.2043	0.0081	0.0455
Cryab	0.3221	0.3857	0.9995	0.3746	0.2794	0.5061	1.2030	0.0017	0.0168
Cenpw	0.6140	0.1257	0.9995	0.4514	0.2400	0.4640	1.2026	0.0036	0.0264
Lamtor3	0.3644	0.4067	0.9995	0.7334	0.0776	0.2346	1.2023	0.0062	0.0384
Hccs	-0.1419	0.7243	0.9995	1.4515	0.0009	0.0177	1.2018	0.0025	0.0209
Gm4610	0.2743	0.3670	0.9995	0.9374	0.0039	0.0396	1.2011	0.0003	0.0062
Nkrf	0.2093	0.6033	0.9995	1.1161	0.0051	0.0465	1.2011	0.0027	0.0222
Rnd3	0.4563	0.1669	0.9995	0.8082	0.0131	0.0810	1.2011	0.0006	0.0087
Sssca1	0.7334	0.0258	0.9995	0.9883	0.0020	0.0260	1.2009	0.0005	0.0073
Vps11	-0.0043	0.9911	0.9995	0.5321	0.1565	0.3598	1.2001	0.0017	0.0168
Aven	0.4758	0.2355	0.9995	0.6084	0.0984	0.2716	1.1987	0.0028	0.0225
Gnl3	0.3080	0.2180	0.9995	0.6974	0.0094	0.0661	1.1973	0.0001	0.0021

St3gal5	-0.0128	0.9730	0.9995	0.5355	0.1505	0.3506	1.1966	0.0017	0.0169
Pigm	0.0101	0.9819	0.9995	0.8939	0.0386	0.1545	1.1963	0.0060	0.0374
Stk17b	0.3094	0.4672	0.9995	0.5483	0.1752	0.3844	1.1960	0.0052	0.0342
Cgn	0.1265	0.7482	0.9995	-0.0197	0.9599	0.9840	1.1956	0.0037	0.0271
Cltb	-0.1476	0.6134	0.9995	1.7506	0.0000	0.0013	1.1927	0.0002	0.0040
Cdt1	0.3561	0.0940	0.9995	0.6934	0.0025	0.0301	1.1925	0.0000	0.0006
Oxnad1	0.2717	0.4689	0.9995	0.8435	0.0210	0.1067	1.1921	0.0019	0.0179
Phactr2	-3.1307	0.0091	0.9995	5.0079	0.0001	0.0050	1.1920	0.1045	0.2419
Noct	0.0124	0.9758	0.9995	1.5775	0.0004	0.0114	1.1910	0.0027	0.0224
Shmt2	0.2555	0.4231	0.9995	1.3175	0.0002	0.0088	1.1885	0.0005	0.0079
Atg16l1	0.1291	0.7677	0.9995	0.9971	0.0186	0.0993	1.1853	0.0056	0.0358
BC002163	0.3758	0.2037	0.9995	0.5906	0.0423	0.1635	1.1831	0.0003	0.0056
Rbmx2	0.3217	0.3045	0.9995	0.6755	0.0284	0.1281	1.1823	0.0005	0.0074
Slc35e1	-0.3611	0.2049	0.9995	1.7443	0.0000	0.0011	1.1813	0.0001	0.0026
Prpf4	-0.0489	0.8971	0.9995	1.3368	0.0015	0.0231	1.1809	0.0022	0.0193
Mrps10	0.6349	0.0679	0.9995	0.8642	0.0097	0.0671	1.1807	0.0010	0.0121
Mcm8	0.2304	0.4636	0.9995	0.6161	0.0475	0.1749	1.1788	0.0004	0.0070
Dnajc9	0.3631	0.2788	0.9995	0.6945	0.0364	0.1490	1.1772	0.0010	0.0117
Cycs	-0.1589	0.6171	0.9995	1.6506	0.0001	0.0040	1.1739	0.0008	0.0103
Gle1	0.4660	0.1347	0.9995	0.8119	0.0082	0.0607	1.1716	0.0004	0.0072
Dnajc25	0.2896	0.4003	0.9995	0.5251	0.1166	0.3001	1.1709	0.0011	0.0126
Srp9	0.3430	0.4321	0.9995	0.3734	0.3683	0.5927	1.1633	0.0080	0.0455
Alkbh4	0.5481	0.1627	0.9995	0.6442	0.0650	0.2091	1.1630	0.0031	0.0238
Alkbh1	-0.0383	0.9334	0.9995	0.9355	0.0336	0.1421	1.1623	0.0067	0.0403
Anapc16	0.2009	0.6029	0.9995	0.4380	0.2393	0.4633	1.1619	0.0033	0.0251
Shmt1	0.4304	0.3203	0.9995	0.9967	0.0183	0.0980	1.1613	0.0065	0.0395
Tent5b	0.3015	0.4972	0.9995	1.0440	0.0157	0.0896	1.1608	0.0070	0.0413
Tomm22	0.3176	0.3911	0.9995	0.8602	0.0256	0.1208	1.1596	0.0030	0.0234
Usp16	-0.0249	0.9301	0.9995	1.1072	0.0008	0.0171	1.1578	0.0003	0.0057
Ghitm	0.0382	0.8837	0.9995	1.0519	0.0007	0.0155	1.1560	0.0002	0.0039
Zfp428	0.3966	0.2861	0.9995	1.1424	0.0024	0.0301	1.1559	0.0020	0.0183
Ndufs5	0.3661	0.2172	0.9995	0.6185	0.0351	0.1456	1.1558	0.0004	0.0065
Ppp5c	0.1885	0.4926	0.9995	0.9871	0.0011	0.0192	1.1553	0.0002	0.0039
Ncoa4	0.1182	0.7883	0.9995	1.0284	0.0194	0.1015	1.1550	0.0080	0.0454
Gm14137	-0.3165	0.3171	0.9995	1.8788	0.0000	0.0011	1.1546	0.0003	0.0055
Napg	0.3119	0.3282	0.9995	0.7066	0.0239	0.1160	1.1534	0.0006	0.0087
Gm8096	-0.4463	0.6152	0.9995	2.4568	0.0036	0.0375	1.1525	0.1245	0.2704
Dtl	0.1615	0.5180	0.9995	1.1459	0.0001	0.0064	1.1512	0.0001	0.0024
Gadd45b	0.1682	0.6283	0.9995	0.8314	0.0220	0.1099	1.1506	0.0018	0.0173
Slc25a10	-0.0708	0.8375	0.9995	1.5631	0.0001	0.0054	1.1495	0.0010	0.0120
Rrn3	-0.0358	0.9165	0.9995	1.0699	0.0044	0.0421	1.1471	0.0014	0.0145
8430429K09Rik	0.2046	0.6086	0.9995	0.6996	0.0694	0.2180	1.1468	0.0033	0.0251
Slc35c1	0.2406	0.5587	0.9995	0.6039	0.1166	0.3001	1.1468	0.0050	0.0330
Tpst2	-0.0875	0.7654	0.9995	1.2008	0.0004	0.0115	1.1464	0.0003	0.0057
Nr2c2ap	0.1235	0.7138	0.9995	0.7729	0.0229	0.1130	1.1450	0.0009	0.0115
Adamts4	0.5149	0.1377	0.9995	1.5499	0.0000	0.0031	1.1406	0.0014	0.0145
Donson	0.0742	0.8330	0.9995	1.0881	0.0036	0.0373	1.1395	0.0014	0.0151
Hars	0.1509	0.6517	0.9995	1.0746	0.0030	0.0334	1.1394	0.0013	0.0141
Csf1	0.1131	0.7689	0.9995	0.5338	0.1673	0.3741	1.1376	0.0044	0.0302
Got1	0.2466	0.5122	0.9995	0.7645	0.0449	0.1692	1.1362	0.0036	0.0266
Wdr75	0.1640	0.6654	0.9995	0.8183	0.0344	0.1440	1.1352	0.0036	0.0266

Osgin2	0.5430	0.2027	0.9995	0.5352	0.1621	0.3677	1.1336	0.0068	0.0405
Triap1	0.4374	0.2142	0.9995	0.7320	0.0353	0.1461	1.1322	0.0020	0.0184
Zfp566	-0.9722	0.1854	0.9995	2.2090	0.0029	0.0333	1.1311	0.0414	0.1309
Cyb5r1	0.0172	0.9576	0.9995	1.2463	0.0007	0.0155	1.1308	0.0009	0.0112
Sp140	0.4241	0.2790	0.9995	-0.2445	0.5213	0.7177	1.1286	0.0039	0.0280
Sco1	0.5916	0.1350	0.9995	0.5219	0.1452	0.3433	1.1285	0.0039	0.0277
Prkab1	0.2882	0.2341	0.9995	0.8052	0.0017	0.0248	1.1276	0.0001	0.0019
9130208D14Rik	0.1501	0.7185	0.9995	0.7377	0.0703	0.2199	1.1260	0.0065	0.0394
Mrps18c	0.1842	0.5639	0.9995	0.5205	0.0983	0.2715	1.1252	0.0009	0.0113
Btg3	0.4844	0.1600	0.9995	0.7239	0.0305	0.1340	1.1221	0.0017	0.0168
Mapt	0.0936	0.7929	0.9995	-0.3097	0.3908	0.6123	1.1204	0.0017	0.0169
Osgin1	-0.1431	0.7446	0.9995	1.3860	0.0029	0.0333	1.1198	0.0072	0.0422
Ly6e	-0.4774	0.2371	0.9995	0.3506	0.3842	0.6069	1.1194	0.0027	0.0224
BC003965	0.2575	0.4211	0.9995	0.9294	0.0047	0.0440	1.1188	0.0009	0.0111
Pwp2	0.0471	0.8802	0.9995	1.0567	0.0018	0.0255	1.1187	0.0006	0.0085
Pdhx	0.2846	0.4809	0.9995	1.1115	0.0060	0.0510	1.1176	0.0055	0.0354
Mcts2	0.1457	0.7343	0.9995	1.2930	0.0029	0.0332	1.1167	0.0077	0.0441
Brms1	0.3267	0.4364	0.9995	0.6005	0.1261	0.3149	1.1150	0.0063	0.0385
Ppil4	0.1132	0.6807	0.9995	0.5921	0.0405	0.1594	1.1150	0.0004	0.0064
Ykt6	0.0791	0.7593	0.9995	0.9767	0.0009	0.0179	1.1149	0.0002	0.0038
Tgoln1	-0.1115	0.6992	0.9995	0.9747	0.0029	0.0328	1.1123	0.0005	0.0079
Hps5	0.2658	0.5170	0.9995	0.3010	0.4386	0.6540	1.1123	0.0055	0.0351
Exosc6	0.6454	0.0372	0.9995	0.3550	0.2041	0.4203	1.1107	0.0006	0.0085
Eif5a	0.8072	0.0452	0.9995	0.4327	0.2574	0.4819	1.1085	0.0056	0.0358
Dtx3l	0.0142	0.9591	0.9995	0.2468	0.3751	0.5992	1.1084	0.0004	0.0063
Polq	-0.3681	0.3827	0.9995	1.5073	0.0008	0.0171	1.1079	0.0037	0.0269
Bud23	0.2349	0.4355	0.9995	0.8017	0.0100	0.0687	1.1070	0.0007	0.0090
Tank	0.0760	0.8198	0.9995	0.7195	0.0330	0.1405	1.1069	0.0014	0.0151
Anks3	-0.4379	0.2416	0.9995	1.1144	0.0047	0.0443	1.1067	0.0016	0.0159
Ndel1	0.1351	0.6974	0.9995	0.7469	0.0360	0.1478	1.1064	0.0024	0.0206
Slc1a5	0.1497	0.6010	0.9995	1.2539	0.0002	0.0078	1.1042	0.0004	0.0070
Tmem14c	0.5914	0.0631	0.9995	0.3465	0.2470	0.4715	1.1026	0.0010	0.0118
Steap1	0.9000	0.0333	0.9995	0.4358	0.2449	0.4693	1.1013	0.0076	0.0436
Nifk	0.0596	0.8411	0.9995	1.3769	0.0002	0.0075	1.1004	0.0008	0.0102
Tprn	0.1659	0.6990	0.9995	1.1064	0.0094	0.0659	1.0977	0.0088	0.0482
Synj1	0.2581	0.3447	0.9995	0.8145	0.0049	0.0454	1.0975	0.0003	0.0057
Pprc1	0.3141	0.3275	0.9995	0.6777	0.0313	0.1358	1.0968	0.0011	0.0123
Tatdn2	0.1951	0.5799	0.9995	0.8040	0.0265	0.1232	1.0963	0.0026	0.0216
Ltv1	0.0836	0.7801	0.9995	0.9405	0.0037	0.0384	1.0958	0.0006	0.0088
Alg8	0.2843	0.4374	0.9995	0.9492	0.0095	0.0663	1.0921	0.0030	0.0233
Hmgn5	0.3267	0.2547	0.9995	0.4449	0.1204	0.3066	1.0911	0.0006	0.0084
Tm4sf1	0.0153	0.9689	0.9995	1.4551	0.0013	0.0209	1.0904	0.0069	0.0408
Terf2ip	-0.1335	0.6682	0.9995	1.2566	0.0004	0.0118	1.0904	0.0008	0.0101
Gid8	0.2403	0.4834	0.9995	0.6041	0.0647	0.2088	1.0901	0.0019	0.0176
Gnb4	0.4709	0.2436	0.9995	0.9814	0.0110	0.0728	1.0896	0.0058	0.0367
Zfp472	0.1989	0.5316	0.9995	0.9497	0.0041	0.0406	1.0893	0.0009	0.0112
Ccdc186	-0.0641	0.7861	0.9995	0.9525	0.0007	0.0151	1.0867	0.0001	0.0027
Myo10	0.0384	0.9032	0.9995	1.1452	0.0012	0.0207	1.0858	0.0011	0.0122
Ddx55	0.0129	0.9628	0.9995	0.7997	0.0076	0.0579	1.0849	0.0004	0.0066
Riox2	-0.0776	0.9006	0.9995	1.9258	0.0023	0.0285	1.0846	0.0451	0.1388
Hmgcll1	0.5965	0.6834	0.9995	3.9524	0.0032	0.0351	1.0845	0.4242	0.5928

Atad5	0.2097	0.4479	0.9995	0.3990	0.1454	0.3436	1.0836	0.0004	0.0065
Chchd7	-0.0072	0.9850	0.9995	0.6283	0.0976	0.2700	1.0833	0.0044	0.0303
Zfp280b	-0.0666	0.8762	0.9995	1.4320	0.0012	0.0206	1.0831	0.0068	0.0408
Mad2l1	0.5242	0.1725	0.9995	0.5699	0.1135	0.2954	1.0808	0.0053	0.0346
Tomm40	0.0358	0.9284	0.9995	0.5629	0.1529	0.3541	1.0803	0.0068	0.0404
Tipin	0.3481	0.1381	0.9995	0.9099	0.0005	0.0135	1.0782	0.0001	0.0024
Psmb10	0.2421	0.3848	0.9995	-0.0781	0.7765	0.8832	1.0780	0.0005	0.0075
Kpna2	0.3609	0.3287	0.9995	0.5171	0.1597	0.3645	1.0768	0.0050	0.0331
Atp6v0a2	-0.4260	0.1840	0.9995	1.9137	0.0000	0.0011	1.0762	0.0005	0.0081
Llph-ps1	0.4732	0.1023	0.9995	0.3026	0.2533	0.4778	1.0761	0.0004	0.0071
Rtel1	0.1430	0.6830	0.9995	1.3645	0.0004	0.0121	1.0759	0.0023	0.0198
Vcam1	0.3204	0.1152	0.9995	0.5961	0.0059	0.0503	1.0749	0.0000	0.0010
Tmx2	-0.0884	0.7987	0.9995	1.2840	0.0010	0.0191	1.0738	0.0022	0.0196
Slc35d1	-0.0621	0.8819	0.9995	0.9576	0.0245	0.1179	1.0729	0.0065	0.0395
Rpp14	0.3445	0.2607	0.9995	0.5452	0.0675	0.2138	1.0727	0.0010	0.0116
Cenpq	0.1520	0.5993	0.9995	0.7137	0.0172	0.0950	1.0719	0.0006	0.0085
Timeless	0.2588	0.3145	0.9995	0.7181	0.0081	0.0605	1.0694	0.0002	0.0046
Gorab	0.1620	0.5783	0.9995	0.7146	0.0167	0.0935	1.0692	0.0006	0.0089
Cdc37l1	0.3584	0.1032	0.9995	0.4893	0.0266	0.1236	1.0676	0.0000	0.0016
Cox18	-0.0110	0.9671	0.9995	0.8667	0.0029	0.0333	1.0671	0.0003	0.0054
Tnfrsf10b	0.0154	0.9637	0.9995	0.8890	0.0127	0.0797	1.0666	0.0025	0.0209
Nhp2	-0.0933	0.8405	0.9995	1.5023	0.0032	0.0351	1.0663	0.0176	0.0753
Espl1	0.3115	0.3540	0.9995	0.6972	0.0317	0.1368	1.0645	0.0020	0.0185
Rfc3	0.2517	0.3733	0.9995	0.5853	0.0417	0.1619	1.0631	0.0006	0.0084
Clspn	-0.1860	0.5019	0.9995	1.1645	0.0004	0.0115	1.0628	0.0004	0.0067
Mafk	-0.0936	0.7980	0.9995	1.3200	0.0011	0.0195	1.0622	0.0029	0.0232
Pfkfb3	0.3773	0.2589	0.9995	0.4768	0.1350	0.3284	1.0621	0.0022	0.0193
Hgh1	0.0363	0.9286	0.9995	0.4981	0.2046	0.4209	1.0619	0.0063	0.0385
Cox5a	0.6848	0.0842	0.9995	0.5420	0.1556	0.3583	1.0617	0.0074	0.0429
Ogfr	-0.2356	0.5202	0.9995	0.9231	0.0172	0.0949	1.0615	0.0034	0.0256
Shisa5	-0.3582	0.3213	0.9995	0.8332	0.0290	0.1295	1.0611	0.0043	0.0298
Lats2	0.3027	0.4033	0.9995	0.8298	0.0198	0.1028	1.0606	0.0032	0.0244
Mrpl10	0.3491	0.3930	0.9995	0.6967	0.0765	0.2323	1.0604	0.0092	0.0496
Nom1	0.2116	0.4244	0.9995	0.9053	0.0021	0.0271	1.0602	0.0003	0.0061
Trim26	0.1991	0.5566	0.9995	0.1551	0.6372	0.7978	1.0591	0.0022	0.0196
Rnf39	-1.4629	0.3283	0.9995	4.1270	0.0046	0.0439	1.0587	0.3927	0.5640
Ctdp1	0.2492	0.5196	0.9995	1.0180	0.0074	0.0569	1.0577	0.0052	0.0342
Cyba	0.4561	0.1930	0.9995	0.3485	0.2933	0.5209	1.0567	0.0033	0.0250
Upf3b	0.3417	0.1817	0.9995	0.3719	0.1389	0.3344	1.0565	0.0002	0.0050
Bola3	0.0091	0.9665	0.9995	1.2701	0.0000	0.0018	1.0559	0.0000	0.0018
Noc3l	-0.0244	0.9399	0.9995	1.1491	0.0016	0.0237	1.0556	0.0017	0.0168
Cops8	-0.0049	0.9868	0.9995	1.1121	0.0010	0.0190	1.0551	0.0009	0.0109
Zfp955a	0.0483	0.8890	0.9995	0.6465	0.0627	0.2058	1.0542	0.0028	0.0228
Pml	0.0403	0.8835	0.9995	0.5544	0.0509	0.1826	1.0541	0.0005	0.0073
Snhg12	0.6166	0.0708	0.9995	0.0531	0.8641	0.9351	1.0537	0.0024	0.0207
Tomm70a	0.5595	0.0652	0.9995	0.4846	0.0982	0.2712	1.0533	0.0010	0.0118
Srm	-0.0070	0.9827	0.9995	1.4447	0.0002	0.0081	1.0531	0.0018	0.0173
Psmc3ip	0.4421	0.2432	0.9995	0.4763	0.1685	0.3758	1.0527	0.0046	0.0315
Pdzrn4	-1.4941	0.3094	0.9995	4.6446	0.0013	0.0212	1.0522	0.3813	0.5540
Usp36	-0.2306	0.5507	0.9995	1.2469	0.0034	0.0362	1.0516	0.0055	0.0353
Ngdn	0.2225	0.4081	0.9995	1.1174	0.0003	0.0111	1.0514	0.0004	0.0068

H2-D1	0.0202	0.9243	0.9995	0.3668	0.0957	0.2668	1.0509	0.0000	0.0019
Elk1	0.3184	0.3450	0.9995	1.1064	0.0014	0.0223	1.0486	0.0024	0.0204
Xpnpep3	0.1451	0.7519	0.9995	1.2696	0.0042	0.0412	1.0471	0.0170	0.0738
Haus3	0.1495	0.5431	0.9995	0.5878	0.0217	0.1090	1.0465	0.0002	0.0040
Rnf19b	0.0421	0.8432	0.9995	1.4511	0.0000	0.0008	1.0440	0.0000	0.0018
Tfrc	0.4691	0.0937	0.9995	1.2526	0.0001	0.0069	1.0432	0.0006	0.0084
Cox5b	0.4045	0.1428	0.9995	0.5884	0.0370	0.1502	1.0427	0.0006	0.0084
Nmi	0.0275	0.9121	0.9995	-0.1624	0.5220	0.7182	1.0389	0.0002	0.0044
Golt1b	-0.0847	0.7741	0.9995	1.2052	0.0004	0.0114	1.0385	0.0007	0.0094
Cdc25a	0.1278	0.6082	0.9995	1.0555	0.0003	0.0106	1.0348	0.0002	0.0047
Abhd17a	0.5043	0.1115	0.9995	0.6949	0.0232	0.1142	1.0347	0.0016	0.0162
Arl16	-0.2106	0.4533	0.9995	0.7166	0.0155	0.0890	1.0341	0.0004	0.0068
Commd6	0.3258	0.3891	0.9995	0.6511	0.0633	0.2063	1.0328	0.0060	0.0376
Zbed4	0.5089	0.0910	0.9995	0.8069	0.0066	0.0533	1.0307	0.0010	0.0116
Zfp131	0.2260	0.4236	0.9995	0.6779	0.0205	0.1051	1.0304	0.0008	0.0102
Zfp568	-0.0929	0.8135	0.9995	0.8208	0.0395	0.1569	1.0299	0.0056	0.0358
Taf1d	0.4325	0.1042	0.9995	0.2806	0.2759	0.5019	1.0272	0.0004	0.0072
Zbtb6	0.1986	0.5195	0.9995	0.7670	0.0139	0.0838	1.0270	0.0013	0.0142
Gvin1	-0.0863	0.7581	0.9995	0.6833	0.0231	0.1139	1.0268	0.0009	0.0110
Nme1	-0.1348	0.7118	0.9995	1.3592	0.0010	0.0191	1.0263	0.0046	0.0313
Spsb1	0.3348	0.4614	0.9995	1.5180	0.0012	0.0201	1.0262	0.0171	0.0741
Prelid3b	-0.4383	0.2222	0.9995	1.4946	0.0003	0.0110	1.0238	0.0031	0.0239
Fam208b	0.0767	0.8216	0.9995	0.9348	0.0086	0.0627	1.0212	0.0028	0.0225
Ftsj3	-0.0154	0.9675	0.9995	1.2526	0.0034	0.0364	1.0209	0.0088	0.0479
Trmt10c	-0.5128	0.1944	0.9995	1.4285	0.0010	0.0189	1.0208	0.0052	0.0342
Smim13	-0.1056	0.7668	0.9995	1.0707	0.0042	0.0412	1.0202	0.0030	0.0236
Brix1	0.0659	0.8398	0.9995	0.6863	0.0420	0.1628	1.0182	0.0029	0.0229
Med28	0.3814	0.2680	0.9995	0.4347	0.1979	0.4129	1.0181	0.0043	0.0300
Fbxo48	-3.3979	0.0068	0.9995	4.5855	0.0004	0.0118	1.0153	0.1186	0.2627
Ddx58	-0.8129	0.0035	0.9995	0.9399	0.0011	0.0199	1.0148	0.0002	0.0039
Gm7334	0.3484	0.2146	0.9995	0.8008	0.0059	0.0504	1.0148	0.0007	0.0097
Sdcbp2	-0.0209	0.9517	0.9995	1.3237	0.0008	0.0170	1.0144	0.0040	0.0283
Suv39h2	-0.1102	0.7431	0.9995	1.5101	0.0001	0.0061	1.0144	0.0023	0.0198
Dcaf13	0.4670	0.1727	0.9995	0.4380	0.1916	0.4044	1.0119	0.0042	0.0296
Tgds	-0.3053	0.4791	0.9995	0.6392	0.1306	0.3213	1.0114	0.0085	0.0470
Lin54	-0.1361	0.6183	0.9995	1.1611	0.0003	0.0104	1.0103	0.0005	0.0079
Pbdc1	0.1616	0.5441	0.9995	0.7784	0.0065	0.0527	1.0097	0.0005	0.0081
Prkrip1	0.0379	0.8796	0.9995	0.9006	0.0015	0.0226	1.0090	0.0003	0.0056
Bloc1s4	-0.1175	0.7685	0.9995	0.7394	0.0631	0.2061	1.0089	0.0091	0.0493
Tmco1	0.1158	0.6482	0.9995	0.9812	0.0010	0.0184	1.0082	0.0004	0.0071
U2af1	0.5933	0.0738	0.9995	0.2991	0.3398	0.5657	1.0075	0.0030	0.0235
Atp6ap2	0.3160	0.3354	0.9995	0.4460	0.1740	0.3830	1.0059	0.0035	0.0262
Nudc	0.2425	0.2971	0.9995	0.9243	0.0005	0.0123	1.0055	0.0001	0.0036
Tars	0.0530	0.8855	0.9995	0.7961	0.0411	0.1608	1.0052	0.0085	0.0468
Timm29	-0.4863	0.2603	0.9995	1.5900	0.0007	0.0159	1.0039	0.0093	0.0500
Pip5k1a	0.0960	0.6249	0.9995	0.8733	0.0003	0.0103	1.0036	0.0000	0.0016
Krt80	-0.0890	0.9204	0.9995	2.6396	0.0021	0.0271	1.0010	0.1973	0.3613
Trnt1	0.0215	0.9398	0.9995	0.9707	0.0022	0.0278	1.0004	0.0009	0.0114
Haus6	-0.1315	0.7128	0.9995	1.0245	0.0070	0.0552	1.0001	0.0046	0.0313
Snhg17	0.1031	0.8076	0.9995	1.2248	0.0044	0.0423	0.9999	0.0131	0.0627
Psme3	0.0179	0.9426	0.9995	1.3062	0.0000	0.0030	0.9997	0.0003	0.0054

Dgat2	0.9211	0.2537	0.9995	3.0360	0.0000	0.0030	0.9995	0.1844	0.3459
Fam98a	0.2704	0.4391	0.9995	0.8975	0.0115	0.0754	0.9993	0.0042	0.0296
Rmi1	0.1908	0.6130	0.9995	0.9285	0.0132	0.0814	0.9988	0.0069	0.0408
Wdr37	0.4591	0.1238	0.9995	0.6812	0.0178	0.0964	0.9979	0.0012	0.0131
Esco2	-0.2020	0.5669	0.9995	1.0243	0.0061	0.0510	0.9976	0.0038	0.0276
Gm4070	-0.1255	0.6502	0.9995	0.6923	0.0201	0.1039	0.9970	0.0010	0.0118
Arfip2	0.1975	0.5525	0.9995	0.8486	0.0124	0.0789	0.9961	0.0029	0.0232
Aftph	0.2403	0.3168	0.9995	0.0487	0.8356	0.9203	0.9958	0.0003	0.0054
Taf9	0.4819	0.0987	0.9995	0.4653	0.1030	0.2793	0.9955	0.0013	0.0138
Hsph1	0.0045	0.9852	0.9995	1.1549	0.0001	0.0065	0.9955	0.0003	0.0057
Rnf166	0.1784	0.6225	0.9995	0.5730	0.1032	0.2795	0.9938	0.0060	0.0376
Rita1	-0.1485	0.6792	0.9995	1.1970	0.0018	0.0255	0.9934	0.0042	0.0296
Prl2a1	0.8202	0.5933	0.9995	6.0334	0.0000	0.0038	0.9907	0.4876	0.6458
Cuta	0.1485	0.6554	0.9995	0.9369	0.0074	0.0569	0.9906	0.0034	0.0257
Sde2	0.2261	0.4834	0.9995	0.5024	0.1202	0.3063	0.9903	0.0034	0.0254
Ddx24	-0.1649	0.5717	0.9995	0.9317	0.0047	0.0439	0.9902	0.0018	0.0171
Rwdd4a	0.1359	0.5977	0.9995	1.0011	0.0008	0.0168	0.9900	0.0005	0.0081
Hspb8	0.3935	0.2005	0.9995	-0.1565	0.6012	0.7747	0.9900	0.0022	0.0197
Atp5g1	0.0096	0.9715	0.9995	1.2495	0.0001	0.0066	0.9900	0.0007	0.0091
Ifi204	0.0143	0.9616	0.9995	0.0933	0.7518	0.8708	0.9895	0.0016	0.0161
Parp3	-0.1698	0.5379	0.9995	0.9224	0.0027	0.0317	0.9893	0.0006	0.0090
Atp6v0c	0.1162	0.6247	0.9995	1.0864	0.0002	0.0088	0.9891	0.0003	0.0058
Acrv1	0.0948	0.9496	0.9995	4.3817	0.0019	0.0255	0.9881	0.4766	0.6376
Utp15	0.1933	0.5389	0.9995	1.0061	0.0030	0.0337	0.9875	0.0023	0.0200
Gpt2	-0.4098	0.3855	0.9995	1.5733	0.0016	0.0236	0.9874	0.0198	0.0814
Cdk11b	0.0880	0.6898	0.9995	0.6962	0.0048	0.0452	0.9851	0.0001	0.0037
Selenos	0.2451	0.4518	0.9995	1.0891	0.0018	0.0249	0.9838	0.0029	0.0230
Strap	0.2061	0.3663	0.9995	0.9510	0.0004	0.0121	0.9835	0.0002	0.0042
Prss23	-1.2245	0.0051	0.9995	2.3605	0.0000	0.0013	0.9830	0.0025	0.0209
Pfkfb2	-0.4414	0.1889	0.9995	1.0848	0.0027	0.0318	0.9827	0.0017	0.0166
Nars	0.1245	0.6133	0.9995	0.5855	0.0267	0.1239	0.9827	0.0005	0.0073
Acot2	-0.6187	0.1574	0.9995	0.9629	0.0297	0.1314	0.9823	0.0091	0.0492
Dpm1	0.1870	0.5658	0.9995	0.6695	0.0376	0.1517	0.9820	0.0032	0.0245
Gtf2f1	0.1232	0.6796	0.9995	0.6938	0.0274	0.1255	0.9813	0.0020	0.0184
2310057M21Rik	-0.4076	0.3459	0.9995	1.6279	0.0006	0.0139	0.9812	0.0105	0.0542
Tmem186	-0.0346	0.9261	0.9995	1.0293	0.0074	0.0571	0.9804	0.0061	0.0379
Stk16	0.3165	0.2521	0.9995	0.6779	0.0145	0.0862	0.9801	0.0008	0.0105
Rrm2b	0.2414	0.5408	0.9995	1.1394	0.0046	0.0438	0.9800	0.0106	0.0546
Zcchc10	-0.0058	0.9830	0.9995	0.9664	0.0017	0.0244	0.9778	0.0008	0.0104
Dnttip2	-0.0020	0.9911	0.9995	0.8950	0.0001	0.0059	0.9777	0.0000	0.0011
Atp6v0c-ps2	0.0562	0.8197	0.9995	1.1283	0.0002	0.0089	0.9773	0.0005	0.0077
Mrpl20	-0.2182	0.4017	0.9995	1.1813	0.0001	0.0069	0.9769	0.0004	0.0065
Dnaja2	0.3743	0.2025	0.9995	0.4364	0.1370	0.3310	0.9766	0.0018	0.0173
Tmem199	0.0781	0.7675	0.9995	1.1949	0.0001	0.0066	0.9743	0.0006	0.0084
Tm2d2	0.4653	0.2089	0.9995	0.3372	0.3202	0.5465	0.9742	0.0082	0.0459
Chka	0.2395	0.4012	0.9995	0.6203	0.0345	0.1441	0.9727	0.0015	0.0152
Dnaaf2	-0.1330	0.6602	0.9995	1.0111	0.0024	0.0296	0.9704	0.0015	0.0157
Slc7a6	0.0740	0.8036	0.9995	1.0137	0.0017	0.0248	0.9703	0.0015	0.0154
Mrpl49	0.2158	0.4607	0.9995	0.5089	0.0824	0.2443	0.9693	0.0016	0.0166
Mis12	0.1396	0.6854	0.9995	0.7665	0.0294	0.1304	0.9629	0.0051	0.0334
Twistnb	0.0806	0.7164	0.9995	0.8272	0.0013	0.0213	0.9629	0.0002	0.0042

Rbsn	0.1335	0.6802	0.9995	0.3741	0.2362	0.4603	0.9619	0.0036	0.0264
Tmem126a	0.4198	0.2151	0.9995	0.6008	0.0599	0.2008	0.9617	0.0048	0.0322
Mat2a	0.1933	0.5823	0.9995	0.9999	0.0096	0.0665	0.9600	0.0084	0.0467
Phospho2	-0.2389	0.4705	0.9995	1.0178	0.0042	0.0413	0.9599	0.0028	0.0228
Sdhaf2	0.0569	0.8661	0.9995	0.5185	0.1221	0.3094	0.9589	0.0050	0.0332
Gusb	0.4130	0.1329	0.9995	0.4175	0.1167	0.3002	0.9588	0.0010	0.0120
Birc5	0.4926	0.0494	0.9995	0.1929	0.4145	0.6334	0.9578	0.0004	0.0069
Med7	-0.1571	0.6659	0.9995	1.4213	0.0004	0.0122	0.9574	0.0063	0.0386
Acap3	0.0911	0.8111	0.9995	1.1777	0.0030	0.0334	0.9568	0.0085	0.0468
Pelo	0.3389	0.3648	0.9995	0.2895	0.4189	0.6377	0.9556	0.0092	0.0495
Zbtb21	-0.1080	0.7319	0.9995	0.5743	0.0702	0.2198	0.9554	0.0024	0.0204
Fastkd5	-0.5253	0.2233	0.9995	1.2252	0.0064	0.0525	0.9546	0.0085	0.0470
Saraf	0.3794	0.1748	0.9995	0.5366	0.0534	0.1880	0.9533	0.0013	0.0140
Paf1	0.0494	0.8478	0.9995	0.9390	0.0011	0.0192	0.9526	0.0005	0.0079
Sec23b	0.1486	0.5841	0.9995	0.5791	0.0339	0.1427	0.9526	0.0008	0.0108
Gmip	-0.2656	0.3831	0.9995	1.2212	0.0004	0.0119	0.9524	0.0014	0.0146
Glcci1	0.3517	0.2867	0.9995	0.5201	0.0978	0.2707	0.9523	0.0043	0.0301
Prpf19	0.2905	0.1967	0.9995	0.1891	0.3866	0.6084	0.9512	0.0002	0.0044
Txn2	0.3875	0.0951	0.9995	0.7456	0.0025	0.0301	0.9505	0.0002	0.0046
Ndufaf2	0.0930	0.7102	0.9995	0.8973	0.0014	0.0219	0.9501	0.0005	0.0076
Rangap1	0.0693	0.8001	0.9995	0.8514	0.0055	0.0484	0.9500	0.0015	0.0155
Psmb3	0.2987	0.2217	0.9995	1.1462	0.0001	0.0055	0.9498	0.0004	0.0070
Fam84b	-0.3928	0.2864	0.9995	1.3702	0.0009	0.0182	0.9497	0.0065	0.0393
Rcl1	0.2854	0.2720	0.9995	0.7519	0.0076	0.0579	0.9486	0.0009	0.0108
Wsb1	-0.0100	0.9630	0.9995	0.7944	0.0014	0.0217	0.9485	0.0001	0.0036
Timmdc1	0.0563	0.8529	0.9995	0.6113	0.0469	0.1739	0.9480	0.0024	0.0206
Sec22b	0.1519	0.6779	0.9995	0.7069	0.0512	0.1834	0.9475	0.0093	0.0497
Nol9	0.3817	0.0905	0.9995	0.6382	0.0058	0.0498	0.9467	0.0002	0.0038
Nop14	-0.0377	0.8784	0.9995	0.6991	0.0104	0.0705	0.9462	0.0007	0.0090
Ctsz	0.4141	0.2527	0.9995	0.4002	0.2479	0.4721	0.9455	0.0093	0.0499
Klhl21	0.3275	0.3178	0.9995	0.4737	0.1295	0.3196	0.9427	0.0044	0.0305
Polr2f	0.4138	0.0801	0.9995	0.9366	0.0005	0.0124	0.9422	0.0003	0.0057
Sap30bp	0.0301	0.9347	0.9995	1.0098	0.0079	0.0598	0.9413	0.0070	0.0414
Polr3d	0.0945	0.6976	0.9995	1.0265	0.0003	0.0110	0.9407	0.0004	0.0071
Smpd4	0.1609	0.6027	0.9995	0.8206	0.0096	0.0669	0.9403	0.0027	0.0219
Zbtb11	-0.2465	0.4006	0.9995	1.0003	0.0023	0.0289	0.9385	0.0017	0.0169
Sox9	-0.1424	0.5053	0.9995	0.5602	0.0159	0.0902	0.9380	0.0002	0.0040
Nop58	0.2499	0.3317	0.9995	0.3593	0.1675	0.3742	0.9380	0.0009	0.0114
Llph-ps2	0.0897	0.7497	0.9995	0.4867	0.0926	0.2623	0.9370	0.0019	0.0177
Letm1	0.0043	0.9891	0.9995	1.2412	0.0008	0.0164	0.9368	0.0043	0.0298
Styk1	0.1999	0.7216	0.9995	1.4935	0.0053	0.0472	0.9359	0.0688	0.1837
Cnbp	0.4431	0.0994	0.9995	0.1967	0.4418	0.6563	0.9345	0.0011	0.0124
Abhd13	0.3102	0.2501	0.9995	0.6400	0.0176	0.0959	0.9338	0.0010	0.0120
Denr	0.8723	0.0008	0.7431	0.1771	0.4072	0.6274	0.9336	0.0002	0.0049
Snrnp48	0.2530	0.3431	0.9995	0.1545	0.5557	0.7452	0.9322	0.0013	0.0139
Eif3j1	0.5145	0.0739	0.9995	0.0783	0.7730	0.8821	0.9315	0.0019	0.0176
Cep162	-0.0176	0.9587	0.9995	0.4566	0.1802	0.3904	0.9313	0.0064	0.0392
Cenpn	0.1261	0.6303	0.9995	0.7689	0.0060	0.0510	0.9313	0.0009	0.0109
Pithd1	0.0955	0.7316	0.9995	0.8121	0.0056	0.0491	0.9271	0.0014	0.0144
Mtrr	0.1047	0.7721	0.9995	0.7376	0.0412	0.1610	0.9262	0.0084	0.0467
Atg12	0.0717	0.7596	0.9995	0.8059	0.0022	0.0279	0.9259	0.0004	0.0067

Thap12	0.2038	0.4041	0.9995	0.7964	0.0035	0.0366	0.9218	0.0007	0.0091
Dnaja1	0.0867	0.7655	0.9995	0.5668	0.0628	0.2059	0.9205	0.0031	0.0239
Gcc1	0.0427	0.8957	0.9995	0.4525	0.1671	0.3739	0.9188	0.0048	0.0324
Gna11	0.5088	0.0829	0.9995	0.3845	0.1641	0.3706	0.9152	0.0024	0.0204
Endod1	-0.0834	0.6837	0.9995	1.0847	0.0000	0.0038	0.9151	0.0001	0.0037
Ufd1	-0.1630	0.4988	0.9995	0.8288	0.0023	0.0291	0.9151	0.0005	0.0076
Tgfb2	-0.6964	0.0493	0.9995	1.6727	0.0001	0.0040	0.9144	0.0029	0.0233
Foxn2	-0.3526	0.4589	0.9995	1.7516	0.0006	0.0145	0.9142	0.0313	0.1087
Eif2s1	0.2236	0.3727	0.9995	0.6585	0.0143	0.0852	0.9142	0.0009	0.0112
Zwilch	0.0129	0.9657	0.9995	0.8926	0.0056	0.0491	0.9131	0.0028	0.0228
Ttc9c	0.2380	0.3511	0.9995	0.4684	0.0714	0.2221	0.9115	0.0010	0.0120
Cebpz	0.2881	0.2666	0.9995	0.3333	0.1982	0.4132	0.9112	0.0012	0.0134
Snx16	0.0489	0.8266	0.9995	0.7909	0.0016	0.0233	0.9107	0.0002	0.0051
Phgdh	0.0877	0.6613	0.9995	0.9879	0.0001	0.0049	0.9103	0.0001	0.0029
Impdh2-ps	0.5079	0.0800	0.9995	0.3175	0.2480	0.4721	0.9103	0.0023	0.0202
Slc29a1	0.1470	0.6340	0.9995	1.1451	0.0007	0.0155	0.9097	0.0036	0.0266
Cks1b	0.6853	0.0513	0.9995	0.2786	0.3963	0.6173	0.9093	0.0086	0.0472
Atf6b	0.3428	0.2409	0.9995	0.4012	0.1505	0.3506	0.9090	0.0026	0.0215
Gm38426	0.2081	0.5345	0.9995	0.6043	0.0661	0.2113	0.9089	0.0060	0.0376
Eif1a	-0.0220	0.9153	0.9995	0.8896	0.0004	0.0114	0.9081	0.0002	0.0038
Clcn5	-0.3065	0.5440	0.9995	1.8785	0.0006	0.0144	0.9080	0.0332	0.1132
Ppm1g	0.1032	0.7005	0.9995	0.8826	0.0037	0.0383	0.9060	0.0019	0.0176
Krt14	-0.3537	0.3532	0.9995	1.2217	0.0033	0.0357	0.9036	0.0122	0.0597
Gm15834	-0.6353	0.2872	0.9995	2.0618	0.0013	0.0209	0.9031	0.0534	0.1555
Lsg1	0.1160	0.6867	0.9995	0.8859	0.0047	0.0443	0.9029	0.0026	0.0216
Slc25a4	0.4083	0.1860	0.9995	0.2796	0.3561	0.5827	0.9016	0.0048	0.0320
Utp6	0.4684	0.1069	0.9995	0.3523	0.1983	0.4132	0.9011	0.0025	0.0212
Zfp655	0.0505	0.8551	0.9995	0.7695	0.0088	0.0632	0.9007	0.0017	0.0168
Irf9	-0.2964	0.3064	0.9995	0.4621	0.1174	0.3013	0.9006	0.0021	0.0190
Gabarapl2	0.4477	0.1848	0.9995	0.2300	0.4779	0.6860	0.9001	0.0084	0.0467
Cpeb3	-0.9050	0.2403	0.9995	2.8687	0.0006	0.0138	0.8966	0.0996	0.2350
Gpatch4	0.1022	0.7293	0.9995	0.9168	0.0049	0.0455	0.8957	0.0037	0.0271
Lonp1	0.0155	0.9438	0.9995	0.6796	0.0054	0.0481	0.8935	0.0003	0.0062
Rbm45	-0.1431	0.6302	0.9995	0.9796	0.0026	0.0311	0.8929	0.0027	0.0222
Srp19	0.3001	0.2829	0.9995	0.6046	0.0334	0.1415	0.8922	0.0023	0.0202
Trim25	-0.1114	0.6789	0.9995	0.5184	0.0650	0.2091	0.8916	0.0019	0.0179
Mrpl16	-0.0702	0.8409	0.9995	1.1115	0.0037	0.0385	0.8914	0.0094	0.0504
Fam111a	0.3050	0.2114	0.9995	0.5646	0.0259	0.1218	0.8906	0.0008	0.0105
Sirt7	-0.1597	0.6359	0.9995	1.2003	0.0014	0.0219	0.8904	0.0062	0.0381
Atp13a1	0.2182	0.3732	0.9995	0.6671	0.0093	0.0658	0.8900	0.0007	0.0098
Pdrg1	0.0062	0.9847	0.9995	0.7766	0.0237	0.1154	0.8895	0.0070	0.0413
Fam126a	0.2468	0.2848	0.9995	0.6627	0.0073	0.0568	0.8883	0.0005	0.0075
Hook1	0.1719	0.5628	0.9995	0.1205	0.6775	0.8231	0.8876	0.0032	0.0242
Aurka	0.1003	0.7026	0.9995	0.8150	0.0046	0.0434	0.8838	0.0016	0.0162
Samhd1	0.1778	0.4294	0.9995	0.3013	0.1804	0.3906	0.8830	0.0004	0.0071
Tomm5	0.5697	0.0645	0.9995	0.6171	0.0310	0.1353	0.8799	0.0042	0.0294
Asns	-0.2448	0.4850	0.9995	1.3546	0.0007	0.0159	0.8797	0.0101	0.0529
Lyar	0.1401	0.5733	0.9995	0.8754	0.0022	0.0283	0.8767	0.0013	0.0142
Commd7	0.0671	0.8336	0.9995	0.5473	0.0844	0.2480	0.8766	0.0061	0.0379
Slc25a19	-0.0332	0.9230	0.9995	1.2224	0.0011	0.0192	0.8762	0.0075	0.0433
Trp53rka	-0.0044	0.9872	0.9995	0.9684	0.0014	0.0219	0.8728	0.0018	0.0174

Mrps2	0.6835	0.0342	0.9995	0.0160	0.9551	0.9821	0.8701	0.0059	0.0369
Rnaseh2a	0.6366	0.0442	0.9995	0.0565	0.8415	0.9229	0.8700	0.0053	0.0343
Setd6	0.3078	0.2578	0.9995	0.5992	0.0274	0.1256	0.8692	0.0020	0.0186
Ddx27	0.0310	0.9197	0.9995	0.7326	0.0263	0.1226	0.8691	0.0067	0.0403
Larp1b	0.3835	0.2144	0.9995	0.4200	0.1529	0.3541	0.8686	0.0054	0.0351
Mrpl22	0.1064	0.6927	0.9995	1.0300	0.0007	0.0155	0.8684	0.0020	0.0182
Riox1	0.2899	0.2780	0.9995	0.3095	0.2313	0.4545	0.8676	0.0018	0.0175
Rars	0.0589	0.8144	0.9995	0.8847	0.0022	0.0279	0.8670	0.0015	0.0159
Usp12	0.0830	0.7796	0.9995	0.5597	0.0635	0.2069	0.8669	0.0044	0.0303
Gtpbp10	-0.4272	0.3105	0.9995	1.2836	0.0039	0.0395	0.8668	0.0199	0.0817
Lrpprc	0.4807	0.1382	0.9995	0.2617	0.3995	0.6203	0.8663	0.0080	0.0454
Med17	-0.1348	0.6429	0.9995	0.7881	0.0109	0.0727	0.8619	0.0034	0.0253
Ebna1bp2	0.1111	0.6754	0.9995	0.6701	0.0177	0.0961	0.8610	0.0023	0.0198
Cib1	-0.1868	0.5897	0.9995	1.0137	0.0062	0.0516	0.8597	0.0089	0.0484
Mrfap1	0.5308	0.0894	0.9995	0.1518	0.6101	0.7817	0.8585	0.0064	0.0391
Krr1	0.1103	0.6507	0.9995	0.7538	0.0049	0.0454	0.8585	0.0011	0.0126
Slc25a29	-0.6207	0.3996	0.9995	2.1507	0.0039	0.0396	0.8565	0.1452	0.2978
AI506816	-0.6189	0.1314	0.9995	1.5162	0.0008	0.0168	0.8561	0.0149	0.0682
Fbxo34	0.5839	0.0578	0.9995	0.1801	0.5082	0.7068	0.8561	0.0047	0.0319
Jpt2	-0.2331	0.3390	0.9995	1.5358	0.0000	0.0010	0.8522	0.0007	0.0097
Timm17a	0.1278	0.6365	0.9995	0.7369	0.0101	0.0692	0.8517	0.0026	0.0215
Exog	-0.3792	0.4259	0.9995	1.3851	0.0052	0.0465	0.8514	0.0334	0.1135
Zfp597	-0.4458	0.2940	0.9995	1.2338	0.0054	0.0477	0.8511	0.0182	0.0770
Cpsf4	-0.0114	0.9635	0.9995	1.0181	0.0004	0.0121	0.8501	0.0012	0.0136
Med19	-0.0221	0.9234	0.9995	0.6914	0.0069	0.0549	0.8488	0.0009	0.0108
Dpm2	-0.1385	0.6207	0.9995	0.9699	0.0019	0.0260	0.8485	0.0027	0.0221
Id1	-0.2175	0.4977	0.9995	1.9689	0.0000	0.0012	0.8469	0.0071	0.0417
Nup54	0.0289	0.9326	0.9995	1.0361	0.0039	0.0395	0.8468	0.0109	0.0554
Taf11	0.3840	0.2139	0.9995	0.4879	0.0966	0.2684	0.8465	0.0063	0.0386
Sap18	0.1793	0.4031	0.9995	0.7693	0.0017	0.0246	0.8461	0.0005	0.0072
Dnajc2	0.2267	0.2525	0.9995	0.6326	0.0042	0.0409	0.8444	0.0002	0.0046
Rabgef1	0.0851	0.7733	0.9995	0.7516	0.0146	0.0864	0.8425	0.0047	0.0318
Snf8	-0.2804	0.3569	0.9995	1.2160	0.0006	0.0135	0.8422	0.0048	0.0321
Bola2	0.1523	0.6350	0.9995	0.5542	0.0812	0.2418	0.8413	0.0088	0.0482
Scoc	0.5686	0.0378	0.9995	0.5735	0.0326	0.1396	0.8388	0.0025	0.0209
Cbwd1	-0.1107	0.7085	0.9995	0.8456	0.0072	0.0561	0.8379	0.0043	0.0298
Tcirg1	0.1837	0.4508	0.9995	0.1999	0.4027	0.6235	0.8370	0.0013	0.0139
1500011B03Rik	0.1367	0.7201	0.9995	1.2126	0.0022	0.0278	0.8361	0.0195	0.0806
BC031181	0.0042	0.9903	0.9995	1.0215	0.0057	0.0494	0.8358	0.0126	0.0610
Tmbim4	0.2982	0.2696	0.9995	0.4944	0.0665	0.2122	0.8357	0.0029	0.0232
Thap2	-0.2733	0.4735	0.9995	1.1546	0.0042	0.0411	0.8357	0.0172	0.0743
Mplkip	-0.1474	0.7105	0.9995	1.1500	0.0058	0.0496	0.8348	0.0261	0.0975
Spdl1	-0.1458	0.6338	0.9995	0.8113	0.0130	0.0808	0.8345	0.0058	0.0367
Ecd	0.2732	0.2670	0.9995	0.6088	0.0160	0.0909	0.8274	0.0015	0.0153
Pinx1	0.9705	0.0028	0.9995	-0.3047	0.2732	0.4988	0.8271	0.0056	0.0358
Cwf19l1	0.0129	0.9662	0.9995	0.4092	0.1772	0.3869	0.8269	0.0060	0.0374
Akap12	0.3817	0.2117	0.9995	1.2221	0.0004	0.0121	0.8248	0.0075	0.0435
Uck1	0.3636	0.2366	0.9995	0.4827	0.0995	0.2734	0.8245	0.0068	0.0407
C330007P06Rik	-0.0161	0.9442	0.9995	0.8422	0.0014	0.0219	0.8218	0.0009	0.0113
Minos1	-0.0028	0.9910	0.9995	1.0322	0.0004	0.0121	0.8213	0.0018	0.0173
Tbrg1	0.0404	0.8199	0.9995	0.7708	0.0004	0.0117	0.8180	0.0001	0.0032

Rap2a	-0.1199	0.6770	0.9995	1.2815	0.0002	0.0071	0.8169	0.0043	0.0301
Sgo1	0.0396	0.8629	0.9995	0.5316	0.0286	0.1286	0.8166	0.0011	0.0125
Uqcc2	0.2750	0.3098	0.9995	0.4649	0.0857	0.2505	0.8132	0.0038	0.0272
Zcchc17	0.0313	0.8896	0.9995	0.6802	0.0061	0.0510	0.8128	0.0009	0.0111
Atic	0.1882	0.3919	0.9995	0.9023	0.0005	0.0130	0.8124	0.0008	0.0103
Naa50	0.1189	0.5976	0.9995	0.6929	0.0057	0.0494	0.8106	0.0011	0.0124
Dlst	0.5369	0.0764	0.9995	0.4180	0.1419	0.3385	0.8105	0.0070	0.0414
Nat10	0.0409	0.8903	0.9995	0.9870	0.0023	0.0292	0.8095	0.0061	0.0377
Aasdhppt	0.3398	0.2166	0.9995	0.5026	0.0599	0.2007	0.8086	0.0038	0.0276
1700047I17Rik2	0.1716	0.4516	0.9995	0.4529	0.0523	0.1857	0.8084	0.0010	0.0119
Mras	0.0997	0.7195	0.9995	0.3858	0.1701	0.3773	0.8080	0.0052	0.0340
Fam177a	0.1706	0.4546	0.9995	0.4529	0.0525	0.1862	0.8079	0.0010	0.0120
Pi4k2a	0.1026	0.6873	0.9995	0.5916	0.0262	0.1225	0.8078	0.0023	0.0202
Bub3	0.4123	0.1282	0.9995	0.3124	0.2335	0.4565	0.8040	0.0039	0.0279
Rhob	0.1648	0.5516	0.9995	0.6097	0.0315	0.1362	0.8035	0.0046	0.0315
Hdac1	0.1178	0.6115	0.9995	0.8552	0.0014	0.0221	0.8033	0.0014	0.0151
Rbm28	-0.0315	0.8868	0.9995	0.9104	0.0006	0.0141	0.8012	0.0010	0.0120
Taf12	-0.0246	0.9279	0.9995	0.6943	0.0146	0.0865	0.8000	0.0035	0.0260
Ckap2l	0.6795	0.0114	0.9995	-0.1532	0.5262	0.7216	0.7993	0.0024	0.0206
Srp14	0.5726	0.0469	0.9995	0.1899	0.4768	0.6851	0.7988	0.0053	0.0344
Sass6	-0.0207	0.9403	0.9995	0.6695	0.0205	0.1052	0.7986	0.0041	0.0288
Opa3	0.3810	0.1298	0.9995	0.2485	0.2967	0.5236	0.7980	0.0021	0.0190
Sap18b	0.1835	0.3498	0.9995	0.7887	0.0006	0.0145	0.7973	0.0003	0.0061
Ccdc92	0.1371	0.6530	0.9995	0.8539	0.0067	0.0541	0.7934	0.0087	0.0478
Ndufb6	0.1759	0.5443	0.9995	0.5939	0.0440	0.1670	0.7931	0.0072	0.0418
Nop16	0.0757	0.7775	0.9995	1.0133	0.0008	0.0171	0.7922	0.0038	0.0272
Rhbdd1	0.1001	0.7450	0.9995	0.5387	0.0745	0.2284	0.7907	0.0087	0.0478
Ecm1	0.0060	0.9866	0.9995	1.2571	0.0015	0.0228	0.7906	0.0216	0.0863
Fxr2	-0.0446	0.8201	0.9995	0.7640	0.0010	0.0186	0.7899	0.0004	0.0067
Kin	-0.2498	0.2920	0.9995	1.0092	0.0003	0.0106	0.7890	0.0012	0.0134
2310061I04Rik	0.5298	0.0494	0.9995	0.2810	0.2474	0.4717	0.7882	0.0034	0.0254
Ddx52	0.1084	0.6360	0.9995	0.3511	0.1337	0.3265	0.7877	0.0015	0.0158
Impdh2	0.4218	0.0891	0.9995	0.4908	0.0486	0.1776	0.7867	0.0023	0.0198
Mpc2	0.1211	0.6500	0.9995	0.7381	0.0089	0.0640	0.7866	0.0040	0.0284
Polr1a	0.0347	0.8872	0.9995	0.8193	0.0028	0.0326	0.7854	0.0023	0.0200
Pole4	0.2721	0.2677	0.9995	0.7669	0.0036	0.0375	0.7847	0.0022	0.0193
Nudt19	0.4930	0.1086	0.9995	0.4304	0.1245	0.3133	0.7826	0.0092	0.0496
Armc1	0.5502	0.0465	0.9995	0.2381	0.3536	0.5806	0.7806	0.0046	0.0314
Ciao2a	0.1669	0.5457	0.9995	0.7018	0.0144	0.0856	0.7795	0.0056	0.0357
Tmf1	0.0524	0.7903	0.9995	0.2610	0.1947	0.4090	0.7777	0.0005	0.0077
B3gat3	-0.1007	0.6975	0.9995	0.8909	0.0019	0.0257	0.7766	0.0029	0.0229
Leprotl1	-0.0471	0.8220	0.9995	0.7799	0.0012	0.0209	0.7764	0.0007	0.0092
A630072M18Rik	-0.7658	0.0791	0.9995	1.4448	0.0017	0.0244	0.7754	0.0258	0.0968
Grpel1	0.0157	0.9385	0.9995	0.9246	0.0002	0.0083	0.7749	0.0006	0.0084
Clic4	-0.1418	0.5440	0.9995	0.7113	0.0062	0.0516	0.7735	0.0020	0.0182
Scamp3	-0.0011	0.9958	0.9995	0.8753	0.0005	0.0123	0.7705	0.0008	0.0102
Map6	-0.5624	0.1851	0.9995	1.4654	0.0016	0.0236	0.7674	0.0333	0.1133
Eif2b4	0.3288	0.1572	0.9995	0.6568	0.0071	0.0556	0.7672	0.0016	0.0166
Snhg5	0.1151	0.6159	0.9995	0.7672	0.0027	0.0320	0.7671	0.0017	0.0168
Pde4dip	-0.2212	0.4807	0.9995	0.9347	0.0056	0.0491	0.7666	0.0112	0.0561
Adgrl1	0.0874	0.7638	0.9995	0.5083	0.0833	0.2461	0.7661	0.0090	0.0487

Ahr	-0.1290	0.6766	0.9995	1.1403	0.0009	0.0182	0.7629	0.0105	0.0542
Nuf2	0.3007	0.2826	0.9995	0.4346	0.1150	0.2976	0.7613	0.0073	0.0425
Foxm1	0.1832	0.3971	0.9995	0.4889	0.0301	0.1324	0.7552	0.0012	0.0136
1700094D03Rik	-0.4639	0.3056	0.9995	1.5810	0.0010	0.0191	0.7526	0.0470	0.1429
Cldnd1	-0.7165	0.0439	0.9995	1.5577	0.0001	0.0069	0.7513	0.0159	0.0708
Arl6ip1	0.2183	0.3931	0.9995	0.3121	0.2261	0.4481	0.7511	0.0051	0.0337
Lmo7	0.0232	0.9128	0.9995	0.1500	0.4816	0.6879	0.7487	0.0012	0.0130
Ahsa1	-0.0510	0.8426	0.9995	1.0026	0.0010	0.0183	0.7475	0.0053	0.0346
Mitf	-0.3801	0.3700	0.9995	1.5876	0.0006	0.0141	0.7470	0.0381	0.1236
2810004N23Rik	0.0368	0.8842	0.9995	0.5984	0.0279	0.1268	0.7461	0.0053	0.0343
Tiparp	-0.0460	0.8447	0.9995	0.6959	0.0071	0.0558	0.7460	0.0026	0.0217
Ppp1r8	0.1915	0.4278	0.9995	0.8739	0.0012	0.0208	0.7455	0.0030	0.0235
Med20	-0.3943	0.2356	0.9995	1.1108	0.0023	0.0286	0.7450	0.0152	0.0690
Ubr7	0.0197	0.9345	0.9995	0.7288	0.0055	0.0483	0.7449	0.0029	0.0231
Cluh	0.0593	0.8118	0.9995	0.9666	0.0009	0.0174	0.7446	0.0041	0.0292
Mphosph6	0.0771	0.7553	0.9995	0.5796	0.0250	0.1190	0.7445	0.0036	0.0265
Errfi1	-0.1390	0.6086	0.9995	1.1663	0.0003	0.0104	0.7443	0.0061	0.0380
Zwint	0.0465	0.8372	0.9995	0.5678	0.0188	0.0995	0.7424	0.0020	0.0186
Ormdl3	-0.4329	0.1650	0.9995	1.2864	0.0003	0.0101	0.7423	0.0080	0.0455
Ggps1	0.3917	0.1496	0.9995	0.1763	0.4843	0.6900	0.7415	0.0062	0.0384
Edc4	-0.3112	0.3441	0.9995	1.0814	0.0025	0.0304	0.7411	0.0166	0.0725
Imp4	-0.1226	0.6660	0.9995	0.8861	0.0046	0.0435	0.7406	0.0085	0.0468
Dcun1d5	0.3146	0.2306	0.9995	0.3555	0.1628	0.3687	0.7405	0.0055	0.0352
Nfx1	-0.0102	0.9683	0.9995	0.5780	0.0298	0.1317	0.7387	0.0045	0.0310
Topors	-0.0162	0.9472	0.9995	0.3957	0.1160	0.2992	0.7368	0.0040	0.0284
6330403K07Rik	1.1032	0.4220	0.9995	3.4849	0.0043	0.0417	0.7363	0.5708	0.7157
Elob	0.1950	0.4181	0.9995	0.5817	0.0234	0.1146	0.7343	0.0039	0.0279
Katnbl1	0.5289	0.0441	0.9995	0.0592	0.8041	0.8993	0.7322	0.0049	0.0328
Nrbf2	-0.0829	0.7520	0.9995	0.9776	0.0011	0.0192	0.7311	0.0054	0.0350
Tfam	0.0939	0.6513	0.9995	0.1099	0.5943	0.7706	0.7311	0.0012	0.0131
Nup155	0.2166	0.3398	0.9995	0.4992	0.0322	0.1386	0.7309	0.0022	0.0196
Trappc3	0.2578	0.3153	0.9995	0.5945	0.0230	0.1136	0.7285	0.0051	0.0338
Pdcd11	-0.0357	0.8884	0.9995	0.7003	0.0115	0.0754	0.7273	0.0058	0.0365
Ttf2	0.2845	0.2849	0.9995	0.6735	0.0128	0.0799	0.7272	0.0064	0.0391
Atp6v1d	0.0408	0.8559	0.9995	0.6137	0.0129	0.0803	0.7271	0.0027	0.0220
Atp6v1f	0.2265	0.3014	0.9995	0.4414	0.0526	0.1865	0.7270	0.0020	0.0184
Psmd3	0.3498	0.1546	0.9995	0.2633	0.2728	0.4986	0.7269	0.0042	0.0294
Ctps	-0.0012	0.9966	0.9995	1.1087	0.0005	0.0129	0.7265	0.0080	0.0455
Fam136a	0.1635	0.6070	0.9995	1.0301	0.0022	0.0278	0.7255	0.0194	0.0803
Ppil1	0.3712	0.1241	0.9995	0.2714	0.2366	0.4605	0.7251	0.0032	0.0247
Lin9	-0.1385	0.6224	0.9995	1.0448	0.0010	0.0190	0.7246	0.0087	0.0478
4921524J17Rik	0.1122	0.6291	0.9995	0.4122	0.0826	0.2449	0.7232	0.0029	0.0233
Tubg1	0.0038	0.9898	0.9995	0.9916	0.0025	0.0303	0.7231	0.0129	0.0623
Stx3	0.3529	0.1700	0.9995	0.1236	0.6166	0.7859	0.7230	0.0058	0.0368
Nfkb2	0.0554	0.8337	0.9995	0.6733	0.0177	0.0961	0.7203	0.0080	0.0454
Eif3j2	0.4346	0.0870	0.9995	0.1081	0.6535	0.8083	0.7203	0.0051	0.0335
Gcsh	0.1471	0.6124	0.9995	1.0418	0.0015	0.0226	0.7202	0.0132	0.0628
Pin1	0.1247	0.6639	0.9995	1.1092	0.0007	0.0151	0.7202	0.0115	0.0574
Erfe	0.0004	0.9989	0.9997	1.0359	0.0006	0.0141	0.7186	0.0064	0.0392
Mecr	-0.1287	0.7753	0.9995	1.3991	0.0032	0.0348	0.7184	0.0738	0.1924
Zfp955b	-0.1633	0.4631	0.9995	0.3616	0.1121	0.2933	0.7173	0.0018	0.0173

Nutf2-ps1	0.4044	0.1244	0.9995	0.2571	0.3146	0.5412	0.7164	0.0071	0.0417
Serp1	-0.1366	0.6460	0.9995	1.1068	0.0012	0.0206	0.7126	0.0151	0.0689
Selenoi	0.4870	0.0780	0.9995	0.2952	0.2491	0.4733	0.7120	0.0088	0.0479
Fbl	0.6287	0.0240	0.9995	0.2206	0.3813	0.6042	0.7105	0.0082	0.0459
Mlx	-0.1549	0.6201	0.9995	1.1800	0.0008	0.0166	0.7093	0.0165	0.0723
Ndufb9	0.0917	0.6738	0.9995	0.7363	0.0032	0.0348	0.7089	0.0026	0.0216
Ccdc174	0.0062	0.9814	0.9995	0.2182	0.4110	0.6305	0.7071	0.0081	0.0457
Mrps7	0.0309	0.9094	0.9995	0.7763	0.0078	0.0591	0.7037	0.0092	0.0496
Eloa	-0.2018	0.3751	0.9995	0.6065	0.0134	0.0819	0.7024	0.0027	0.0222
Snrpb	0.2892	0.2186	0.9995	0.4426	0.0636	0.2069	0.7021	0.0040	0.0286
Ppp1r10	0.2307	0.2523	0.9995	0.4397	0.0339	0.1427	0.7014	0.0012	0.0132
Zfp809	-0.0259	0.9228	0.9995	0.6176	0.0266	0.1235	0.6991	0.0083	0.0464
5-Mar	0.0636	0.7826	0.9995	0.3753	0.1135	0.2954	0.6988	0.0041	0.0289
Rab21	-0.0257	0.8956	0.9995	0.7253	0.0014	0.0222	0.6985	0.0011	0.0124
Hs6st1	-0.3358	0.1529	0.9995	0.6273	0.0118	0.0764	0.6968	0.0030	0.0233
Pycrl	0.0274	0.9355	0.9995	0.9886	0.0049	0.0452	0.6935	0.0291	0.1041
Rnf14	-0.1541	0.5132	0.9995	0.5254	0.0348	0.1447	0.6931	0.0044	0.0302
Dimt1	-0.3971	0.4285	0.9995	1.6269	0.0020	0.0263	0.6875	0.0967	0.2303
Ubn1	0.1348	0.4835	0.9995	0.1510	0.4319	0.6478	0.6846	0.0012	0.0133
Rad17	0.0862	0.7291	0.9995	0.6878	0.0096	0.0665	0.6831	0.0068	0.0405
Mdn1	0.0050	0.9852	0.9995	0.8350	0.0055	0.0482	0.6825	0.0127	0.0614
Prps1l3	0.1713	0.4474	0.9995	0.8006	0.0015	0.0228	0.6807	0.0036	0.0264
Sdad1	0.1989	0.3651	0.9995	0.4285	0.0584	0.1973	0.6780	0.0034	0.0253
Prr13	0.2945	0.2235	0.9995	0.5211	0.0366	0.1493	0.6778	0.0063	0.0386
Fbxo28	0.1181	0.5931	0.9995	0.4833	0.0355	0.1470	0.6773	0.0034	0.0255
Eri1	0.2291	0.3061	0.9995	0.4905	0.0346	0.1442	0.6759	0.0038	0.0277
Surf4	0.2530	0.2877	0.9995	0.7185	0.0046	0.0433	0.6751	0.0053	0.0346
Nans	0.1402	0.5020	0.9995	0.5762	0.0101	0.0695	0.6751	0.0022	0.0196
Snapc1	-0.0732	0.7909	0.9995	0.9202	0.0026	0.0311	0.6719	0.0126	0.0613
Nploc4	0.4384	0.0779	0.9995	0.3873	0.0997	0.2737	0.6716	0.0064	0.0391
Prim1	-0.0693	0.7994	0.9995	0.8887	0.0031	0.0342	0.6710	0.0124	0.0603
Aagab	-0.0540	0.8169	0.9995	0.6740	0.0076	0.0579	0.6706	0.0047	0.0320
Skp2	0.3803	0.1245	0.9995	0.5405	0.0263	0.1226	0.6705	0.0066	0.0398
Nif3l1	0.3718	0.1845	0.9995	0.7986	0.0047	0.0439	0.6705	0.0144	0.0668
Cpsf2	0.0618	0.7769	0.9995	0.6255	0.0093	0.0658	0.6703	0.0038	0.0274
Klhdc3	0.1338	0.6770	0.9995	0.9613	0.0038	0.0390	0.6702	0.0284	0.1025
Hspa5	0.0727	0.7538	0.9995	0.4565	0.0616	0.2039	0.6683	0.0062	0.0383
Pak1ip1	0.0958	0.6860	0.9995	0.3931	0.1061	0.2837	0.6665	0.0066	0.0399
Gins4	0.2868	0.2539	0.9995	0.4709	0.0581	0.1970	0.6648	0.0081	0.0457
Txndc9	0.2932	0.1658	0.9995	0.3694	0.0815	0.2423	0.6620	0.0027	0.0223
Cstf1	-0.1648	0.6012	0.9995	0.9720	0.0044	0.0421	0.6607	0.0244	0.0933
Mtf1	-0.4618	0.0720	0.9995	1.0046	0.0005	0.0130	0.6592	0.0052	0.0340
Heatr1	-0.0536	0.8651	0.9995	1.0927	0.0019	0.0257	0.6578	0.0294	0.1044
Fam126b	-0.2351	0.3487	0.9995	0.9644	0.0008	0.0166	0.6576	0.0072	0.0423
Zfp24	-0.1977	0.4475	0.9995	0.8432	0.0032	0.0349	0.6564	0.0088	0.0480
G3bp2	0.1848	0.3929	0.9995	0.3504	0.1135	0.2954	0.6561	0.0040	0.0287
Cfap298	0.3115	0.1488	0.9995	0.3626	0.0897	0.2569	0.6560	0.0032	0.0247
Caap1	-0.0180	0.9561	0.9995	1.0147	0.0037	0.0383	0.6558	0.0336	0.1140
Msantd4	0.0305	0.9104	0.9995	0.8965	0.0031	0.0341	0.6552	0.0159	0.0706
Dynll2	0.0359	0.8807	0.9995	0.6124	0.0178	0.0965	0.6550	0.0081	0.0458
Larp7	0.0879	0.6581	0.9995	0.2849	0.1612	0.3667	0.6518	0.0024	0.0204

Mphosph8	0.3357	0.1232	0.9995	-0.1417	0.5007	0.7016	0.6504	0.0037	0.0269
Atp6v1e1	-0.0426	0.8395	0.9995	0.6733	0.0045	0.0430	0.6489	0.0036	0.0263
Eea1	-0.1982	0.4956	0.9995	0.9202	0.0049	0.0452	0.6488	0.0253	0.0956
Zc3h18	0.0577	0.7856	0.9995	0.6258	0.0077	0.0582	0.6475	0.0039	0.0280
Yars	-0.1642	0.5919	0.9995	1.0473	0.0021	0.0272	0.6471	0.0259	0.0970
Cct6a	0.1531	0.4544	0.9995	0.6806	0.0031	0.0341	0.6452	0.0029	0.0232
Cdca8	0.3530	0.0865	0.9995	0.2882	0.1510	0.3514	0.6451	0.0025	0.0209
Pigl	-3.2846	0.0136	0.9995	4.4442	0.0013	0.0209	0.6440	0.4048	0.5748
Rpl7l1	0.2128	0.2993	0.9995	0.5484	0.0120	0.0773	0.6439	0.0028	0.0225
BC005624	0.2443	0.3071	0.9995	0.3685	0.1152	0.2980	0.6428	0.0075	0.0435
Zfp275	0.0641	0.7864	0.9995	0.6159	0.0135	0.0826	0.6410	0.0073	0.0423
Ncapg2	0.1176	0.6166	0.9995	0.6094	0.0156	0.0896	0.6399	0.0080	0.0455
Eif3g	0.0422	0.8461	0.9995	0.8039	0.0015	0.0232	0.6395	0.0052	0.0342
Tnfaip1	0.2441	0.2927	0.9995	0.6133	0.0136	0.0828	0.6385	0.0074	0.0429
Fam192a	0.1169	0.5536	0.9995	0.3998	0.0514	0.1835	0.6376	0.0024	0.0206
Mybl1	-0.2658	0.4000	0.9995	1.2043	0.0007	0.0155	0.6340	0.0276	0.1007
Mrpl13	0.1553	0.4379	0.9995	0.6110	0.0059	0.0503	0.6306	0.0030	0.0237
Purb	0.0619	0.7177	0.9995	0.4021	0.0288	0.1290	0.6298	0.0010	0.0116
Rps27l	0.0509	0.8287	0.9995	0.7590	0.0042	0.0412	0.6296	0.0095	0.0505
Tmem33	0.4459	0.0656	0.9995	0.2891	0.1987	0.4137	0.6287	0.0082	0.0461
Csnk2b	0.0453	0.8460	0.9995	0.6167	0.0130	0.0809	0.6281	0.0076	0.0436
Tamm41	-0.2418	0.4102	0.9995	0.8992	0.0051	0.0462	0.6264	0.0239	0.0921
Vcpkmt	-0.3052	0.4593	0.9995	1.2477	0.0041	0.0408	0.6243	0.0807	0.2044
Emc6	0.3260	0.0866	0.9995	0.5130	0.0102	0.0698	0.6238	0.0017	0.0169
Senp1	-0.0384	0.9004	0.9995	1.0519	0.0020	0.0264	0.6220	0.0351	0.1168
Utp23	0.0868	0.7124	0.9995	0.5506	0.0249	0.1189	0.6212	0.0087	0.0479
Utp14a	-0.0978	0.6829	0.9995	0.8333	0.0019	0.0257	0.6207	0.0088	0.0482
Secisbp2	-0.3171	0.3738	0.9995	1.1259	0.0034	0.0362	0.6201	0.0490	0.1465
Fam98b	-0.0525	0.8225	0.9995	0.6812	0.0078	0.0587	0.6191	0.0090	0.0490
Smc2	0.1526	0.4873	0.9995	0.1953	0.3749	0.5991	0.6157	0.0071	0.0417
Rbm27	0.4174	0.0721	0.9995	0.1050	0.6305	0.7942	0.6154	0.0076	0.0438
Dda1	-0.1949	0.5346	0.9995	1.2069	0.0008	0.0164	0.6144	0.0367	0.1204
Med8	-0.0325	0.8824	0.9995	0.9514	0.0003	0.0104	0.6122	0.0062	0.0384
Drg2	-0.0811	0.7684	0.9995	0.9457	0.0018	0.0255	0.6119	0.0201	0.0822
Tuft1	-0.3396	0.3154	0.9995	1.0227	0.0049	0.0452	0.6106	0.0448	0.1384
Dbf4	0.2490	0.1742	0.9995	0.2012	0.2652	0.4905	0.6083	0.0018	0.0173
Kars	0.2930	0.1783	0.9995	0.7258	0.0027	0.0321	0.6071	0.0064	0.0391
Samd8	-0.0664	0.8088	0.9995	0.8927	0.0033	0.0355	0.6064	0.0225	0.0887
Nol6	0.0613	0.8304	0.9995	1.0828	0.0008	0.0161	0.6055	0.0280	0.1016
Sbds	0.0892	0.6748	0.9995	0.4714	0.0362	0.1484	0.6029	0.0064	0.0393
Pcolce2	-0.3201	0.3813	0.9995	1.1550	0.0041	0.0407	0.6001	0.0759	0.1962
Dusp3	-0.3444	0.3003	0.9995	1.1321	0.0023	0.0284	0.5995	0.0503	0.1495
Npm3-ps1	-0.1403	0.6344	0.9995	0.9058	0.0058	0.0498	0.5989	0.0383	0.1240
Il17d	-1.8545	0.0644	0.9995	3.1244	0.0022	0.0284	0.5985	0.3748	0.5478
Akap11	0.0476	0.8133	0.9995	0.5993	0.0069	0.0548	0.5975	0.0045	0.0307
Npm3	-0.1019	0.6194	0.9995	0.7104	0.0025	0.0307	0.5943	0.0055	0.0353
Eif3c	-0.0041	0.9834	0.9995	0.5085	0.0175	0.0958	0.5891	0.0046	0.0315
Nol11	-0.0145	0.9493	0.9995	1.0234	0.0003	0.0097	0.5887	0.0117	0.0582
Dusp14	-0.0160	0.9591	0.9995	1.1636	0.0009	0.0177	0.5839	0.0484	0.1453
Smap2	-0.1697	0.5426	0.9995	0.8718	0.0045	0.0428	0.5832	0.0295	0.1046
Ell2	-0.2036	0.4700	0.9995	1.1756	0.0003	0.0106	0.5827	0.0269	0.0994

Cox6a1	0.1682	0.3671	0.9995	0.5646	0.0064	0.0524	0.5813	0.0033	0.0252
Rwdd1	0.2242	0.2806	0.9995	0.2727	0.1902	0.4029	0.5786	0.0069	0.0408
Gps1	-0.0208	0.9479	0.9995	0.9903	0.0050	0.0461	0.5736	0.0620	0.1707
Sec23ip	-0.2074	0.4541	0.9995	0.8593	0.0045	0.0431	0.5724	0.0295	0.1046
Tox4	-0.1764	0.4947	0.9995	0.8674	0.0025	0.0301	0.5688	0.0213	0.0852
Brd2	0.4008	0.0438	0.9995	-0.1657	0.3784	0.6017	0.5636	0.0045	0.0312
Mta2	-0.0446	0.8528	0.9995	0.7465	0.0051	0.0461	0.5632	0.0186	0.0779
Gm13552	-0.1633	0.5561	0.9995	0.9199	0.0030	0.0338	0.5627	0.0345	0.1156
Polb	-0.3796	0.1794	0.9995	1.0349	0.0012	0.0207	0.5618	0.0325	0.1116
Exoc8	-0.2697	0.4514	0.9995	1.0708	0.0056	0.0491	0.5611	0.0818	0.2062
Cct3	0.2584	0.1812	0.9995	0.5638	0.0072	0.0561	0.5608	0.0049	0.0329
Tomm6	0.0436	0.8572	0.9995	0.9380	0.0010	0.0189	0.5588	0.0221	0.0873
Twf1	-0.0331	0.9101	0.9995	1.0111	0.0019	0.0258	0.5530	0.0477	0.1443
Guk1	-0.2871	0.2837	0.9995	0.9628	0.0015	0.0230	0.5524	0.0293	0.1043
Tma16	-0.1332	0.5655	0.9995	0.7115	0.0056	0.0491	0.5524	0.0163	0.0718
Cyc1	-0.1254	0.5803	0.9995	1.0270	0.0002	0.0081	0.5429	0.0151	0.0688
Ipo13	-0.4655	0.1416	0.9995	1.1553	0.0010	0.0188	0.5410	0.0469	0.1427
Bcl10	-0.0614	0.8226	0.9995	0.9140	0.0028	0.0322	0.5355	0.0421	0.1324
Blcap	0.0912	0.7251	0.9995	0.7884	0.0052	0.0465	0.5352	0.0339	0.1145
Lin7c	0.0253	0.9213	0.9995	0.8166	0.0040	0.0403	0.5346	0.0335	0.1137
Clptm1	-0.1873	0.4589	0.9995	0.8995	0.0016	0.0234	0.5294	0.0270	0.0996
Zfp599	-0.2242	0.4473	0.9995	1.3422	0.0001	0.0069	0.5281	0.0528	0.1545
Dnajc5	-0.0503	0.7961	0.9995	0.4019	0.0500	0.1808	0.5238	0.0088	0.0479
Fancm	-0.3978	0.3326	0.9995	1.2675	0.0042	0.0414	0.5231	0.1398	0.2903
Armc6	-0.2008	0.6382	0.9995	1.4117	0.0018	0.0255	0.5193	0.1648	0.3221
Zmat3	-0.3259	0.3948	0.9995	1.1764	0.0041	0.0408	0.5122	0.1264	0.2727
Slc39a13	0.0903	0.7190	0.9995	0.8574	0.0021	0.0271	0.4952	0.0422	0.1327
Clock	-0.6906	0.0443	0.9995	1.2194	0.0012	0.0201	0.4941	0.0826	0.2078
Rtca	-0.2287	0.3714	0.9995	0.9351	0.0013	0.0212	0.4913	0.0407	0.1293
Wdr18	0.0407	0.8837	0.9995	0.8385	0.0056	0.0487	0.4887	0.0652	0.1769
Lars	-0.1183	0.6340	0.9995	0.9154	0.0014	0.0223	0.4859	0.0448	0.1382
2500004C02Rik	-0.7038	0.2397	0.9995	1.6796	0.0054	0.0478	0.4778	0.3081	0.4840
Il1rl1	-2.3933	0.0981	0.9995	4.7873	0.0011	0.0199	0.4712	0.6601	0.7834
Map7d1	0.1086	0.6068	0.9995	0.6554	0.0049	0.0455	0.4685	0.0252	0.0955
Xrcc1	-0.3296	0.3560	0.9995	1.0865	0.0047	0.0441	0.4645	0.1444	0.2967
Eif3b	-0.1272	0.6423	0.9995	0.9364	0.0028	0.0322	0.4602	0.0824	0.2074
Ppp4r2	0.2104	0.2229	0.9995	0.1926	0.2598	0.4847	0.4590	0.0089	0.0486
Snhg9	-0.3865	0.2299	0.9995	1.3663	0.0003	0.0110	0.4574	0.1197	0.2639
Nab1	-0.3395	0.1531	0.9995	1.0566	0.0002	0.0081	0.4492	0.0417	0.1316
Wdr76	-0.1084	0.7251	0.9995	0.9386	0.0049	0.0455	0.4413	0.1211	0.2656
Pgp	-0.3292	0.2587	0.9995	1.0261	0.0016	0.0233	0.4401	0.0908	0.2212
Timp1	0.0065	0.9778	0.9995	1.2527	0.0000	0.0029	0.4338	0.0530	0.1548
9130401M01Rik	-0.0688	0.7239	0.9995	0.6511	0.0033	0.0353	0.4281	0.0273	0.1001
1-Sep	-1.3491	0.3534	0.9995	4.0215	0.0049	0.0455	0.4267	0.7201	0.8286
Tbc1d2b	-0.3082	0.4166	0.9995	1.1708	0.0032	0.0351	0.4175	0.2029	0.3682
Eml6	1.2706	0.0817	0.9995	-5.7274	0.0001	0.0049	0.4134	0.5540	0.7017
Psat1	-0.0893	0.7034	0.9995	0.8484	0.0018	0.0255	0.4098	0.0729	0.1910
Ncbp2	-0.4152	0.1727	0.9995	0.9863	0.0032	0.0351	0.4093	0.1344	0.2828
Aimp2	-0.2924	0.3626	0.9995	0.9961	0.0047	0.0441	0.3893	0.1807	0.3413
Mrpl38	-0.1611	0.5244	0.9995	0.7719	0.0051	0.0461	0.3836	0.1010	0.2369
Acbd7	-1.0239	0.5228	0.9995	4.9621	0.0013	0.0217	0.3784	0.7818	0.8700

Id2	1.3473	0.0001	0.3624	-1.2997	0.0002	0.0081	0.3767	0.1657	0.3234
Eif2d	-0.1307	0.6530	0.9995	0.8931	0.0044	0.0426	0.3704	0.1658	0.3234
Tmem167	-0.0548	0.8101	0.9995	0.7157	0.0047	0.0441	0.3626	0.0954	0.2282
Tor1b	-0.4279	0.1516	0.9995	0.9870	0.0028	0.0326	0.3214	0.2303	0.3999
Pphln1	-0.1488	0.5589	0.9995	0.8515	0.0027	0.0320	0.3115	0.1833	0.3443
1110008P14Rik	0.3608	0.4588	0.9995	1.7186	0.0009	0.0177	0.3039	0.4997	0.6572
Kctd3	-0.2721	0.3285	0.9995	1.1752	0.0003	0.0100	0.2827	0.2527	0.4255
Zfp703	-0.2749	0.2094	0.9995	0.8534	0.0007	0.0159	0.2668	0.1789	0.3392
Nop10	0.0442	0.8249	0.9995	0.8530	0.0004	0.0120	0.2481	0.1924	0.3552
Naa60	-0.2690	0.3885	0.9995	0.9509	0.0050	0.0459	0.2424	0.3822	0.5546
Mrm2	-0.7426	0.0963	0.9995	1.3637	0.0038	0.0390	0.2407	0.4783	0.6387
Chst11	-0.8401	0.0888	0.9995	1.7094	0.0011	0.0195	0.2108	0.5720	0.7167
Fsd1l	-3.3206	0.0050	0.9995	3.7537	0.0016	0.0237	0.2032	0.7457	0.8448
Tmem86a	0.3834	0.1762	0.9995	-1.2869	0.0004	0.0118	0.1955	0.4547	0.6187
Vav2	-3.8672	0.0069	0.9995	5.4703	0.0003	0.0097	0.1862	0.8248	0.8967
Tfb2m	-0.1267	0.6047	0.9995	0.7887	0.0036	0.0373	0.1846	0.4098	0.5799
Lims2	1.6725	0.0880	0.9995	-4.8195	0.0010	0.0191	0.1824	0.8504	0.9120
Pes1	-0.2873	0.3068	0.9995	0.9402	0.0027	0.0321	0.1787	0.4781	0.6385
Tmem115	-0.3281	0.3584	0.9995	1.2973	0.0010	0.0186	0.1608	0.5958	0.7358
Itgb7	-3.0391	0.0213	0.9995	3.7932	0.0043	0.0414	0.1578	0.8511	0.9123
Vpreb1	0.7862	0.4373	0.9995	-4.1936	0.0040	0.0403	0.1046	0.9149	0.9508
Il23a	-0.1517	0.6239	0.9995	1.0654	0.0021	0.0272	0.1038	0.7116	0.8224
Mlxip	-0.0059	0.9871	0.9995	1.0638	0.0050	0.0461	0.0886	0.7906	0.8759
Frk	0.7098	0.0032	0.9995	-0.7910	0.0015	0.0224	0.0356	0.8594	0.9170
Epha4	-0.9401	0.0139	0.9995	1.4966	0.0003	0.0109	0.0212	0.9417	0.9671
Upk3bl	1.0141	0.0075	0.9995	-1.2265	0.0025	0.0302	0.0003	0.9994	0.9998
Ccdc34	0.4019	0.2105	0.9995	-1.0750	0.0033	0.0356	-0.0579	0.8439	0.9083
Lgals8	0.1314	0.5507	0.9995	-0.9635	0.0006	0.0145	-0.0867	0.6739	0.7936
Prkce	0.3957	0.2308	0.9995	-1.1014	0.0054	0.0481	-0.0960	0.7591	0.8544
Gnpda2	0.4241	0.2511	0.9995	-1.2073	0.0055	0.0485	-0.1028	0.7680	0.8606
Rnf214	0.0799	0.7744	0.9995	-1.0024	0.0031	0.0343	-0.1169	0.6550	0.7794
Ppp4r1l-ps	1.0158	0.1302	0.9995	-2.4481	0.0051	0.0465	-0.1240	0.8521	0.9128
Mtdh	0.3063	0.1648	0.9995	-0.7241	0.0034	0.0362	-0.1340	0.5066	0.6625
Top1	0.2093	0.3872	0.9995	-0.7999	0.0038	0.0388	-0.1612	0.4755	0.6367
Malat1	-0.0549	0.8434	0.9995	-0.8993	0.0047	0.0441	-0.1630	0.5329	0.6839
Tcerg1	0.3193	0.1065	0.9995	-0.7439	0.0011	0.0198	-0.1974	0.2768	0.4519
Coro1b	0.2424	0.2918	0.9995	-0.7613	0.0038	0.0390	-0.2047	0.3422	0.5177
Ei24	0.2388	0.2716	0.9995	-0.9238	0.0007	0.0154	-0.2068	0.3150	0.4905
Tmem231	0.6138	0.2887	0.9995	-2.5048	0.0032	0.0349	-0.2205	0.6986	0.8126
Brd4	0.3791	0.2034	0.9995	-1.1785	0.0010	0.0185	-0.2214	0.4230	0.5916
Nexn	0.4100	0.1341	0.9995	-1.2869	0.0004	0.0114	-0.2246	0.3862	0.5579
Clmn	-0.4567	0.5392	0.9995	-4.5481	0.0012	0.0204	-0.2260	0.7303	0.8357
Ung	0.5647	0.1707	0.9995	-1.3844	0.0048	0.0444	-0.2275	0.5639	0.7099
Pwwp2b	-0.0911	0.6841	0.9995	-0.9899	0.0009	0.0174	-0.2369	0.2648	0.4386
Mmp14	-0.1350	0.5737	0.9995	-0.9552	0.0012	0.0201	-0.2414	0.2879	0.4636
Rpl28	0.5382	0.0464	0.9995	-0.8398	0.0040	0.0399	-0.2419	0.3172	0.4925
Ptdss2	0.3072	0.3303	0.9995	-1.1361	0.0038	0.0390	-0.2572	0.3991	0.5698
Rpl15	0.5023	0.0835	0.9995	-0.9620	0.0028	0.0322	-0.2596	0.3242	0.5000
Ywhae	0.4860	0.0596	0.9995	-1.0357	0.0005	0.0133	-0.2636	0.2584	0.4316
Snx14	0.0435	0.8863	0.9995	-1.0888	0.0050	0.0459	-0.2639	0.3680	0.5419
Srek1ip1	0.2858	0.2210	0.9995	-0.8511	0.0020	0.0264	-0.2728	0.2192	0.3864

6-Sep	0.3512	0.1288	0.9995	-0.7492	0.0040	0.0403	-0.2876	0.1864	0.3481
Rbm6	0.0550	0.8101	0.9995	-0.7864	0.0037	0.0384	-0.2921	0.1859	0.3477
Use1	0.4129	0.1851	0.9995	-1.0967	0.0027	0.0318	-0.2939	0.3180	0.4935
Zfp316	0.3084	0.3728	0.9995	-1.2572	0.0042	0.0410	-0.2941	0.3792	0.5521
Smc4	0.3829	0.1656	0.9995	-1.0367	0.0012	0.0206	-0.3012	0.2410	0.4129
Nampt	0.0329	0.8773	0.9995	-0.6849	0.0054	0.0480	-0.3210	0.1226	0.2679
Slc39a11	0.5982	0.0426	0.9995	-1.1893	0.0008	0.0165	-0.3225	0.2498	0.4225
Htatsf1	0.1934	0.3201	0.9995	-0.6548	0.0036	0.0375	-0.3296	0.0828	0.2079
A830010M20Rik	0.3416	0.3283	0.9995	-1.7398	0.0008	0.0162	-0.3357	0.3331	0.5088
Zmynd11	0.1786	0.4207	0.9995	-0.7078	0.0052	0.0467	-0.3457	0.1083	0.2483
Dnal4	0.7399	0.0948	0.9995	-1.7205	0.0018	0.0249	-0.3562	0.4200	0.5891
Hist1h2bc	0.7702	0.2070	0.9995	-2.9393	0.0017	0.0246	-0.3564	0.5704	0.7154
Ankhd1	0.1752	0.4845	0.9995	-1.0954	0.0005	0.0122	-0.3570	0.1400	0.2905
Ctdsp1	0.1914	0.4007	0.9995	-0.7949	0.0036	0.0379	-0.3604	0.1105	0.2513
Ppp6r3	0.5669	0.0347	0.9995	-1.0921	0.0005	0.0124	-0.3641	0.1377	0.2874
Gpam	1.1744	0.0995	0.9995	-3.9822	0.0004	0.0115	-0.3675	0.6113	0.7467
Golga4	-0.0161	0.9371	0.9995	-0.7724	0.0016	0.0236	-0.3698	0.0675	0.1812
Cir1	0.0834	0.6963	0.9995	-0.7485	0.0030	0.0334	-0.3699	0.0792	0.2016
Ccl3	0.2227	0.8717	0.9995	3.7644	0.0032	0.0350	-0.3725	0.7778	0.8677
Rps6	0.4459	0.1147	0.9995	-1.1787	0.0004	0.0122	-0.3737	0.1553	0.3105
Naca	0.3287	0.1959	0.9995	-0.7965	0.0050	0.0457	-0.3801	0.1162	0.2594
Rpl18a	0.5131	0.1616	0.9995	-1.4259	0.0009	0.0177	-0.3829	0.2599	0.4331
Prnp	0.3169	0.2052	0.9995	-0.9815	0.0011	0.0198	-0.3850	0.1130	0.2549
Zfas1	0.5690	0.0985	0.9995	-1.6520	0.0001	0.0065	-0.3871	0.2236	0.3923
Scamp1	0.7898	0.0642	0.9995	-1.5556	0.0018	0.0252	-0.3873	0.3349	0.5103
Ufsp2	0.1669	0.3676	0.9995	-0.6308	0.0033	0.0354	-0.3983	0.0324	0.1113
Stxbp2	0.0800	0.6711	0.9995	-0.6995	0.0019	0.0260	-0.4061	0.0333	0.1133
Ktn1	0.0655	0.7148	0.9995	-0.6371	0.0024	0.0295	-0.4078	0.0250	0.0950
Rpl13-ps6	0.3865	0.2596	0.9995	-1.2122	0.0022	0.0278	-0.4124	0.2029	0.3682
Arl5a	0.2314	0.3505	0.9995	-0.8058	0.0046	0.0438	-0.4181	0.0847	0.2111
Pias3	0.7415	0.0923	0.9995	-1.4744	0.0043	0.0421	-0.4196	0.3428	0.5180
Rps19	0.2227	0.4321	0.9995	-1.0655	0.0014	0.0223	-0.4248	0.1206	0.2651
Sox4	0.3876	0.1197	0.9995	-1.1324	0.0002	0.0084	-0.4273	0.0718	0.1889
Abca2	0.3198	0.1968	0.9995	-0.9873	0.0011	0.0199	-0.4284	0.0814	0.2057
Rpl22	0.2569	0.2193	0.9995	-0.6474	0.0054	0.0478	-0.4291	0.0369	0.1209
Hmga1b	0.9343	0.0026	0.9995	-1.1395	0.0005	0.0129	-0.4300	0.1009	0.2369
Faah	1.1125	0.0133	0.9995	-1.7233	0.0012	0.0203	-0.4336	0.3393	0.5149
Hivep1	0.2634	0.3229	0.9995	-0.9377	0.0033	0.0358	-0.4350	0.1033	0.2402
Impact	-0.6071	0.0998	0.9995	1.0736	0.0053	0.0474	-0.4354	0.1786	0.3388
Hmga1	0.9328	0.0025	0.9995	-1.1450	0.0004	0.0122	-0.4355	0.0949	0.2272
Rps26	0.4441	0.1936	0.9995	-1.2792	0.0013	0.0209	-0.4398	0.1704	0.3285
Gm10224	0.3831	0.1154	0.9995	-0.8882	0.0014	0.0221	-0.4411	0.0580	0.1636
Tfap2a	-0.0245	0.9113	0.9995	-1.0622	0.0003	0.0109	-0.4467	0.0450	0.1386
Wac	0.2706	0.1642	0.9995	-0.7219	0.0015	0.0228	-0.4521	0.0208	0.0841
Asxl2	0.1445	0.4746	0.9995	-0.7559	0.0021	0.0268	-0.4534	0.0291	0.1041
Smarca2	0.1711	0.4360	0.9995	-1.0979	0.0001	0.0060	-0.4590	0.0364	0.1197
Rab2a	0.1556	0.4807	0.9995	-0.7582	0.0031	0.0342	-0.4608	0.0372	0.1215
Ddx10	0.2624	0.2639	0.9995	-0.9162	0.0012	0.0201	-0.4621	0.0471	0.1431
Ncoa6	0.1641	0.4963	0.9995	-0.9095	0.0019	0.0259	-0.4645	0.0558	0.1595
Rp9	0.1915	0.4009	0.9995	-0.8288	0.0020	0.0264	-0.4711	0.0385	0.1244
Hist3h2a	0.0573	0.8795	0.9995	-1.9935	0.0009	0.0182	-0.4752	0.2064	0.3722

Cavin2	0.4692	0.1022	0.9995	-1.2949	0.0002	0.0088	-0.4776	0.0796	0.2025
Mbtps1	-0.0367	0.8327	0.9995	-0.5417	0.0064	0.0524	-0.4812	0.0084	0.0468
Nrp2	1.1313	0.0008	0.7431	-1.5925	0.0000	0.0030	-0.4950	0.0801	0.2033
Rab10	-0.0120	0.9468	0.9995	-0.4985	0.0125	0.0789	-0.4975	0.0083	0.0464
Gls	-0.0086	0.9608	0.9995	-0.1428	0.4218	0.6401	-0.5010	0.0070	0.0413
Shtn1	-0.0317	0.8756	0.9995	-0.7665	0.0018	0.0255	-0.5032	0.0164	0.0720
Tbx15	0.1078	0.7906	0.9995	-1.8568	0.0023	0.0289	-0.5035	0.2225	0.3907
Acp2	0.1767	0.5410	0.9995	-1.0475	0.0040	0.0401	-0.5047	0.0857	0.2128
Phf2	0.2914	0.2343	0.9995	-0.8688	0.0029	0.0332	-0.5098	0.0433	0.1349
Xrn2	-0.0438	0.8321	0.9995	-0.7211	0.0028	0.0323	-0.5107	0.0162	0.0718
Glrx	0.3908	0.2114	0.9995	-1.2609	0.0013	0.0213	-0.5112	0.1043	0.2416
Acadl	0.1099	0.6080	0.9995	-0.9844	0.0004	0.0115	-0.5126	0.0207	0.0838
Hspa4l	0.4786	0.0598	0.9995	-1.1386	0.0003	0.0104	-0.5127	0.0420	0.1322
Rps10	0.4533	0.1112	0.9995	-0.9620	0.0026	0.0308	-0.5128	0.0592	0.1654
Vim	0.2636	0.2501	0.9995	-0.9217	0.0007	0.0157	-0.5172	0.0237	0.0917
Pds5a	-0.0601	0.7631	0.9995	-0.7748	0.0017	0.0246	-0.5206	0.0128	0.0618
Abcb7	0.0996	0.7556	0.9995	-1.4545	0.0012	0.0203	-0.5213	0.1093	0.2498
Setd2	-0.0735	0.6674	0.9995	-0.6274	0.0020	0.0267	-0.5228	0.0044	0.0302
Nipal3	0.1448	0.6960	0.9995	-1.9172	0.0007	0.0159	-0.5282	0.1613	0.3181
Ufl1	0.0599	0.7937	0.9995	-1.1204	0.0003	0.0108	-0.5317	0.0260	0.0970
Xpa	-0.0423	0.8589	0.9995	-1.1700	0.0004	0.0114	-0.5318	0.0306	0.1071
Ube3a	0.0256	0.8828	0.9995	-0.4929	0.0116	0.0756	-0.5355	0.0043	0.0298
Thrap3	-0.1054	0.6237	0.9995	-0.6946	0.0050	0.0459	-0.5365	0.0153	0.0691
Cdr2l	0.5187	0.2206	0.9995	-1.6968	0.0028	0.0326	-0.5400	0.2190	0.3862
Rpl31	0.2033	0.3293	0.9995	-0.6733	0.0043	0.0414	-0.5449	0.0109	0.0553
Zfp580	0.1297	0.7101	0.9995	-1.4950	0.0022	0.0278	-0.5450	0.1294	0.2765
Anxa3	0.1991	0.3356	0.9995	-0.6798	0.0038	0.0390	-0.5476	0.0102	0.0532
Eif1b	0.2967	0.2070	0.9995	-1.2110	0.0001	0.0055	-0.5514	0.0211	0.0847
Hectd1	0.1133	0.5461	0.9995	-0.8665	0.0003	0.0094	-0.5542	0.0054	0.0351
Atpif1	-0.0563	0.7722	0.9995	-0.5070	0.0180	0.0970	-0.5548	0.0069	0.0409
Gm6654	0.3457	0.2217	0.9995	-1.2066	0.0004	0.0120	-0.5550	0.0449	0.1385
Card10	-0.0185	0.9418	0.9995	-0.9287	0.0024	0.0297	-0.5566	0.0309	0.1078
Bzw2	0.0316	0.8668	0.9995	-0.2081	0.2801	0.5065	-0.5595	0.0055	0.0351
Tpm4	0.0580	0.7777	0.9995	-0.5397	0.0172	0.0949	-0.5626	0.0091	0.0490
Cenpc1	0.2429	0.3663	0.9995	-1.0060	0.0021	0.0271	-0.5646	0.0397	0.1274
Parn	0.0725	0.7849	0.9995	-0.9689	0.0031	0.0340	-0.5677	0.0393	0.1263
Ezh2	0.0646	0.7431	0.9995	-0.9141	0.0003	0.0094	-0.5681	0.0065	0.0396
Gm6252	-0.0258	0.8904	0.9995	-0.6340	0.0035	0.0368	-0.5689	0.0046	0.0314
Ino80	-0.0382	0.8587	0.9995	-0.7348	0.0045	0.0430	-0.5722	0.0118	0.0586
Pcmtd1	0.0160	0.9431	0.9995	-0.8021	0.0027	0.0321	-0.5786	0.0136	0.0644
Rtn4	0.2270	0.2831	0.9995	-0.7339	0.0025	0.0308	-0.5818	0.0080	0.0454
Caprin1	-0.0041	0.9815	0.9995	-0.6781	0.0014	0.0219	-0.5823	0.0026	0.0217
Chd2	-0.0271	0.9011	0.9995	-0.9187	0.0007	0.0155	-0.5830	0.0108	0.0550
Hnrnpa3	0.0818	0.7140	0.9995	-0.8694	0.0012	0.0201	-0.5849	0.0117	0.0581
Tpt1	0.0531	0.8596	0.9995	-1.0529	0.0043	0.0415	-0.5854	0.0554	0.1589
Mien1	-0.0346	0.8880	0.9995	-0.9360	0.0021	0.0270	-0.5873	0.0211	0.0847
Atp5mpl	-0.0713	0.7226	0.9995	-0.2272	0.2684	0.4944	-0.5878	0.0059	0.0369
Gtf2a1	-0.0833	0.6859	0.9995	-0.6602	0.0061	0.0513	-0.5925	0.0070	0.0411
Cdc25b	0.3703	0.2470	0.9995	-1.0250	0.0053	0.0472	-0.5934	0.0618	0.1704
Jak2	-0.6852	0.0058	0.9995	0.2027	0.3681	0.5926	-0.5947	0.0087	0.0477
Rpl37rt	0.0676	0.7539	0.9995	-0.5090	0.0291	0.1296	-0.5953	0.0085	0.0470

Pls3	0.2872	0.2369	0.9995	-0.7574	0.0055	0.0486	-0.5969	0.0158	0.0706
Rpl24	0.0878	0.6274	0.9995	-0.4831	0.0152	0.0881	-0.5969	0.0024	0.0208
Sugt1	-0.1718	0.4317	0.9995	-0.3738	0.1020	0.2777	-0.5973	0.0086	0.0475
Rpl7	0.2528	0.1929	0.9995	-0.8263	0.0004	0.0118	-0.5991	0.0034	0.0253
Psme4	-0.1183	0.5203	0.9995	-0.5485	0.0088	0.0634	-0.6000	0.0027	0.0223
Arid4b	-0.0175	0.9280	0.9995	-0.9660	0.0001	0.0068	-0.6007	0.0040	0.0284
Sp3	0.0325	0.8725	0.9995	-0.5407	0.0175	0.0959	-0.6023	0.0058	0.0368
Zmym4	-0.0698	0.7804	0.9995	-0.9327	0.0032	0.0351	-0.6023	0.0218	0.0868
Smchd1	0.0034	0.9858	0.9995	-0.2876	0.1488	0.3485	-0.6049	0.0036	0.0264
Cox7a2l	0.3124	0.2086	0.9995	-0.9533	0.0011	0.0196	-0.6055	0.0158	0.0705
Gapvd1	-0.1867	0.4034	0.9995	-0.4102	0.0840	0.2472	-0.6066	0.0092	0.0494
Dync1i2	0.1142	0.6210	0.9995	-0.8856	0.0013	0.0213	-0.6095	0.0111	0.0561
Rpl23	0.3656	0.1078	0.9995	-1.1057	0.0001	0.0055	-0.6110	0.0078	0.0447
Ick	-0.0365	0.8988	0.9995	-1.1941	0.0017	0.0243	-0.6114	0.0398	0.1274
Rev3l	-0.2358	0.2968	0.9995	-0.2817	0.2324	0.4560	-0.6123	0.0092	0.0496
Dusp11	-0.0574	0.7884	0.9995	-0.7147	0.0049	0.0455	-0.6163	0.0072	0.0421
Nudt13	-0.1893	0.4836	0.9995	-1.0626	0.0025	0.0303	-0.6183	0.0258	0.0967
Ube2h	-0.2880	0.2047	0.9995	-0.5486	0.0249	0.1190	-0.6193	0.0079	0.0450
Gkap1	-0.0483	0.8168	0.9995	-0.4482	0.0475	0.1749	-0.6234	0.0054	0.0348
Add3	-0.2205	0.3083	0.9995	-0.2868	0.1965	0.4114	-0.6242	0.0059	0.0373
Sec63	-0.0843	0.7113	0.9995	-0.7331	0.0055	0.0485	-0.6258	0.0090	0.0487
Phip	-0.1284	0.5527	0.9995	-0.9876	0.0004	0.0113	-0.6305	0.0060	0.0375
Usp47	0.0134	0.9441	0.9995	-0.6498	0.0040	0.0397	-0.6308	0.0028	0.0225
S100a3	0.8601	0.1096	0.9995	-2.4297	0.0023	0.0288	-0.6316	0.2757	0.4507
Prdx2	-0.1165	0.6019	0.9995	-0.3204	0.1649	0.3713	-0.6327	0.0071	0.0417
Uvssa	-0.2074	0.3688	0.9995	-0.3692	0.1349	0.3283	-0.6330	0.0090	0.0487
Setd1b	0.2566	0.3404	0.9995	-0.9451	0.0031	0.0340	-0.6345	0.0229	0.0897
Cul3	0.0270	0.9021	0.9995	-0.4497	0.0580	0.1969	-0.6365	0.0070	0.0413
Ralgds	0.3215	0.2606	0.9995	-0.9094	0.0058	0.0499	-0.6382	0.0308	0.1076
Adgrg6	-0.0655	0.7659	0.9995	0.0158	0.9430	0.9760	-0.6385	0.0069	0.0409
Iqgap1	-0.0628	0.7281	0.9995	-0.3891	0.0439	0.1669	-0.6387	0.0014	0.0150
Anp32b	0.1361	0.6407	0.9995	-0.9943	0.0032	0.0350	-0.6387	0.0300	0.1058
Rpsa	0.1009	0.6335	0.9995	-0.6614	0.0058	0.0498	-0.6389	0.0046	0.0315
Map7	0.0524	0.8731	0.9995	-1.2402	0.0034	0.0364	-0.6432	0.0591	0.1654
Gnb1	-0.0447	0.8330	0.9995	-0.4262	0.0603	0.2015	-0.6437	0.0047	0.0320
Itfg1	0.1342	0.5907	0.9995	-0.8784	0.0035	0.0372	-0.6506	0.0148	0.0679
Rps16	0.2741	0.2121	0.9995	-0.6866	0.0050	0.0458	-0.6508	0.0046	0.0314
Sp1	0.3615	0.1870	0.9995	-1.0073	0.0016	0.0239	-0.6524	0.0185	0.0777
Tslp	0.2443	0.4185	0.9995	-1.0609	0.0033	0.0352	-0.6529	0.0360	0.1189
Prkacb	0.1559	0.4613	0.9995	-0.6062	0.0105	0.0710	-0.6530	0.0042	0.0294
Rps8	0.1222	0.4850	0.9995	-0.7775	0.0003	0.0109	-0.6531	0.0009	0.0109
Wipf2	-0.2497	0.2761	0.9995	-0.6785	0.0105	0.0710	-0.6541	0.0066	0.0397
Kif13a	0.2593	0.4051	0.9995	-1.1426	0.0027	0.0321	-0.6556	0.0433	0.1350
Rab14	-0.3105	0.1877	0.9995	-0.2016	0.3868	0.6085	-0.6567	0.0067	0.0403
Med13	-0.2797	0.1808	0.9995	-0.3580	0.0991	0.2727	-0.6573	0.0030	0.0233
Ankrd17	0.1388	0.4589	0.9995	-0.9941	0.0001	0.0042	-0.6577	0.0015	0.0155
Cyfip1	-0.0542	0.8124	0.9995	-0.4640	0.0598	0.2006	-0.6593	0.0070	0.0412
Ptbp3	0.1388	0.5628	0.9995	-0.7470	0.0063	0.0517	-0.6593	0.0089	0.0484
Sfpq	0.0346	0.8899	0.9995	-0.8752	0.0028	0.0322	-0.6599	0.0114	0.0569
Mkln1	-0.0794	0.7243	0.9995	-0.5513	0.0293	0.1302	-0.6604	0.0068	0.0404
Smurf2	0.0146	0.9342	0.9995	-0.2882	0.1202	0.3063	-0.6613	0.0010	0.0120

Rpl6	-0.0075	0.9669	0.9995	-0.6436	0.0024	0.0295	-0.6666	0.0010	0.0122
Dap	0.4243	0.1217	0.9995	-0.9363	0.0027	0.0319	-0.6666	0.0165	0.0722
Nipsnap1	-0.1916	0.3433	0.9995	-0.5594	0.0139	0.0837	-0.6669	0.0024	0.0206
Kmt5b	-0.0482	0.8260	0.9995	-0.4870	0.0437	0.1667	-0.6677	0.0053	0.0344
Ilf3	-0.0501	0.8112	0.9995	-0.7853	0.0018	0.0251	-0.6677	0.0033	0.0253
Ltbp4	0.4269	0.1441	0.9995	-1.3302	0.0004	0.0119	-0.6684	0.0302	0.1063
Ap1g1	0.0062	0.9754	0.9995	-0.7053	0.0032	0.0351	-0.6688	0.0027	0.0222
Hnrnpm	0.1078	0.6621	0.9995	-0.8567	0.0028	0.0324	-0.6695	0.0094	0.0504
Hmgb2	-0.1214	0.5978	0.9995	-0.4516	0.0631	0.2061	-0.6699	0.0059	0.0370
Mpp1	0.2361	0.4060	0.9995	-1.4060	0.0002	0.0088	-0.6722	0.0240	0.0923
Rad21	-0.1187	0.6101	0.9995	-0.4599	0.0622	0.2051	-0.6726	0.0063	0.0385
Anxa4	-0.0332	0.8784	0.9995	-0.6270	0.0110	0.0731	-0.6730	0.0042	0.0296
Col4a1	-0.1602	0.4220	0.9995	-0.4304	0.0447	0.1688	-0.6741	0.0020	0.0183
Map2k4	0.0249	0.9073	0.9995	-1.0650	0.0002	0.0088	-0.6775	0.0042	0.0296
Ptpra	-0.1665	0.4068	0.9995	-0.5983	0.0093	0.0658	-0.6780	0.0021	0.0189
Spag9	-0.0579	0.7823	0.9995	-0.5956	0.0122	0.0778	-0.6833	0.0029	0.0232
Sh3bgrl	-0.1261	0.5681	0.9995	-0.2717	0.2331	0.4564	-0.6841	0.0041	0.0292
Mfn1	-0.3849	0.1293	0.9995	-0.5048	0.0685	0.2159	-0.6846	0.0080	0.0454
Rpl17-ps10	0.0726	0.8236	0.9995	-1.2907	0.0019	0.0260	-0.6849	0.0424	0.1328
Map4	0.2122	0.4340	0.9995	-1.0255	0.0018	0.0249	-0.6870	0.0155	0.0698
Ube4b	0.0473	0.8096	0.9995	-0.6276	0.0059	0.0503	-0.6871	0.0018	0.0173
Smco4	0.0856	0.7593	0.9995	-1.0494	0.0028	0.0323	-0.6885	0.0213	0.0851
Crebbp	-0.2082	0.3486	0.9995	-0.5945	0.0155	0.0890	-0.6897	0.0036	0.0265
Ctnnal1	-0.2896	0.1622	0.9995	-0.5577	0.0159	0.0902	-0.6905	0.0019	0.0180
Sms	0.0555	0.8223	0.9995	-0.3840	0.1360	0.3296	-0.6910	0.0082	0.0459
Krt8	-0.1355	0.4915	0.9995	-0.4811	0.0246	0.1181	-0.6914	0.0015	0.0153
Csnk1g1	-0.1274	0.6108	0.9995	-0.4779	0.0788	0.2369	-0.6950	0.0092	0.0495
Mrpl48	0.0987	0.6382	0.9995	-0.9922	0.0004	0.0111	-0.6955	0.0031	0.0239
Fn1	-0.3314	0.1444	0.9995	-0.1752	0.4312	0.6474	-0.6960	0.0033	0.0252
Matr3	0.0792	0.6758	0.9995	-0.7296	0.0013	0.0215	-0.6962	0.0011	0.0125
Itgb1	-0.1296	0.4535	0.9995	-0.4438	0.0185	0.0988	-0.6965	0.0004	0.0071
Copb1	-0.3416	0.1883	0.9995	-0.1619	0.5298	0.7244	-0.6972	0.0086	0.0475
Micu2	-0.2039	0.4029	0.9995	-0.9066	0.0028	0.0322	-0.6992	0.0070	0.0411
Mnat1	-0.0729	0.6969	0.9995	-0.6153	0.0049	0.0452	-0.6994	0.0010	0.0118
Zdhhc2	-0.0819	0.7328	0.9995	-0.1056	0.6638	0.8148	-0.7010	0.0069	0.0408
Crebrf	-0.2819	0.2435	0.9995	-0.6784	0.0126	0.0794	-0.7030	0.0055	0.0352
Arhgef9	0.2699	0.5106	0.9995	-2.2495	0.0012	0.0207	-0.7048	0.1159	0.2589
Ctdspl	0.0903	0.7136	0.9995	-0.6990	0.0137	0.0829	-0.7056	0.0086	0.0472
Idh3g	-0.1731	0.4958	0.9995	-0.2834	0.2812	0.5080	-0.7057	0.0085	0.0471
Uqcc3	-0.2157	0.3934	0.9995	-0.5087	0.0667	0.2125	-0.7065	0.0081	0.0455
Nbeal1	-0.1240	0.6109	0.9995	-0.5638	0.0375	0.1516	-0.7068	0.0067	0.0404
Odf2	-0.1182	0.6376	0.9995	-0.3814	0.1511	0.3515	-0.7076	0.0082	0.0459
Zfand6	0.1436	0.5045	0.9995	-0.6432	0.0085	0.0621	-0.7077	0.0028	0.0228
Myo6	0.1297	0.6542	0.9995	-1.4276	0.0003	0.0103	-0.7103	0.0198	0.0815
Dnajc1	0.1401	0.4909	0.9995	-0.7905	0.0014	0.0221	-0.7119	0.0018	0.0174
Zfp148	-0.1323	0.5506	0.9995	-0.3035	0.1869	0.3987	-0.7129	0.0031	0.0239
Kdm3a	-0.0890	0.7582	0.9995	-1.0661	0.0030	0.0336	-0.7151	0.0186	0.0780
Chchd3	0.2007	0.3915	0.9995	-0.8747	0.0021	0.0269	-0.7196	0.0052	0.0339
Ube2e3	0.1989	0.3254	0.9995	-1.0588	0.0001	0.0055	-0.7200	0.0016	0.0162
Apln	0.3476	0.1360	0.9995	-0.9034	0.0012	0.0201	-0.7212	0.0044	0.0302
Afdn	-0.0424	0.8293	0.9995	-0.6337	0.0051	0.0461	-0.7217	0.0011	0.0125

Gtf3c3	-0.1237	0.6251	0.9995	-0.5054	0.0718	0.2226	-0.7242	0.0082	0.0459
Atp2b1	0.1185	0.5354	0.9995	-0.5643	0.0084	0.0614	-0.7247	0.0008	0.0103
Abcc1	0.2449	0.3209	0.9995	-0.5916	0.0270	0.1246	-0.7263	0.0065	0.0395
Wipf1	-0.0015	0.9952	0.9995	-0.6321	0.0270	0.1246	-0.7283	0.0080	0.0454
Fkbp1a	-0.0775	0.7613	0.9995	-0.3054	0.2423	0.4669	-0.7287	0.0069	0.0408
Mboat1	-0.0129	0.9660	0.9995	-1.0741	0.0037	0.0383	-0.7288	0.0218	0.0868
Arl3	-0.0981	0.6295	0.9995	-0.4794	0.0313	0.1358	-0.7292	0.0014	0.0145
Flna	0.0947	0.6064	0.9995	-0.5863	0.0052	0.0470	-0.7305	0.0005	0.0081
Cdc42bpa	0.0311	0.8801	0.9995	-0.6505	0.0067	0.0540	-0.7319	0.0017	0.0168
Cnn3	-0.2143	0.4055	0.9995	-0.5521	0.0466	0.1732	-0.7334	0.0068	0.0405
Rpl37a	0.2329	0.2199	0.9995	-0.8776	0.0002	0.0081	-0.7354	0.0006	0.0082
Nhsl1	-0.4411	0.1045	0.9995	-0.1992	0.4651	0.6764	-0.7357	0.0076	0.0439
Hk2	0.4993	0.0482	0.9995	-0.6943	0.0092	0.0650	-0.7357	0.0043	0.0299
Acadm	-0.1229	0.6262	0.9995	-0.5753	0.0388	0.1550	-0.7381	0.0064	0.0392
Vwa5a	-0.0373	0.8801	0.9995	-1.2831	0.0001	0.0069	-0.7382	0.0058	0.0368
Hnrnpr	-0.0834	0.7338	0.9995	-0.8340	0.0038	0.0390	-0.7395	0.0050	0.0330
Nrf1	0.0112	0.9672	0.9995	-1.0175	0.0034	0.0362	-0.7404	0.0130	0.0625
Tspo	0.0502	0.8480	0.9995	-0.6461	0.0254	0.1201	-0.7408	0.0079	0.0451
Runx1	-0.4662	0.0811	0.9995	-0.1547	0.5581	0.7467	-0.7410	0.0062	0.0382
Kdm1a	-0.2500	0.3492	0.9995	-0.3110	0.2577	0.4824	-0.7425	0.0079	0.0452
Lbh	-0.2906	0.2424	0.9995	-0.8144	0.0066	0.0534	-0.7435	0.0049	0.0325
Ubxn7	-0.0865	0.7307	0.9995	-0.9137	0.0030	0.0336	-0.7440	0.0064	0.0391
Acss2	0.3726	0.3116	0.9995	-1.4053	0.0027	0.0321	-0.7462	0.0615	0.1699
Myo5a	-0.1462	0.5545	0.9995	-0.5354	0.0463	0.1727	-0.7462	0.0049	0.0328
Zfp654	-0.0621	0.8459	0.9995	-1.6456	0.0005	0.0130	-0.7465	0.0284	0.1026
Pcnx	0.2245	0.3704	0.9995	-0.8513	0.0049	0.0454	-0.7471	0.0079	0.0451
Dars	-0.2664	0.2291	0.9995	-0.3883	0.0958	0.2670	-0.7471	0.0019	0.0178
Nr2f1	-0.3741	0.1708	0.9995	-0.4444	0.1301	0.3204	-0.7480	0.0080	0.0454
Mrpl23	0.2111	0.4222	0.9995	-1.0275	0.0016	0.0234	-0.7483	0.0091	0.0493
Tulp4	-0.4952	0.0825	0.9995	0.1219	0.6593	0.8120	-0.7495	0.0085	0.0471
Zbtb4	0.0433	0.8853	0.9995	-1.1670	0.0025	0.0302	-0.7495	0.0200	0.0821
Gdpd5	1.0340	0.0031	0.9995	-1.3661	0.0004	0.0114	-0.7496	0.0299	0.1054
Gm13160	-0.0830	0.7399	0.9995	-0.5683	0.0390	0.1553	-0.7518	0.0054	0.0348
Arhgef12	-0.3762	0.1174	0.9995	-0.5867	0.0246	0.1182	-0.7547	0.0028	0.0224
Mbtd1	-0.1882	0.4105	0.9995	-0.0530	0.8177	0.9086	-0.7585	0.0026	0.0216
Gcat	-0.1007	0.6536	0.9995	-0.0415	0.8540	0.9294	-0.7589	0.0023	0.0200
Kmt2c	-0.2030	0.3834	0.9995	-0.9211	0.0015	0.0226	-0.7594	0.0028	0.0224
Stx8	-0.3773	0.1706	0.9995	0.0954	0.7278	0.8546	-0.7611	0.0078	0.0447
Map4k4	-0.1725	0.3952	0.9995	-0.1842	0.3668	0.5917	-0.7633	0.0008	0.0103
Hltf	-0.0177	0.9478	0.9995	-0.6786	0.0269	0.1245	-0.7640	0.0089	0.0484
Galnt7	0.1658	0.4692	0.9995	-0.4393	0.0711	0.2217	-0.7645	0.0029	0.0229
Rpl35	-0.0267	0.9153	0.9995	-0.7377	0.0088	0.0634	-0.7652	0.0044	0.0304
Xrn1	-0.4124	0.1289	0.9995	-0.5574	0.0560	0.1936	-0.7652	0.0061	0.0378
Gm7285	-0.1363	0.5534	0.9995	-0.4801	0.0494	0.1792	-0.7652	0.0022	0.0197
Zfand3	0.0614	0.7842	0.9995	-0.8377	0.0023	0.0285	-0.7656	0.0025	0.0212
Pcm1	-0.1943	0.3342	0.9995	-0.6242	0.0059	0.0503	-0.7689	0.0007	0.0091
Rnaseh2c	0.1998	0.3816	0.9995	-0.6611	0.0104	0.0705	-0.7728	0.0025	0.0212
Atraid	-0.2415	0.3039	0.9995	-0.6778	0.0126	0.0794	-0.7729	0.0026	0.0216
Slc4a7	-0.2844	0.2785	0.9995	-0.0344	0.8957	0.9510	-0.7730	0.0054	0.0350
Prkd3	-0.1096	0.6729	0.9995	-0.5403	0.0569	0.1950	-0.7751	0.0060	0.0375
Fmnl2	0.1447	0.5004	0.9995	-0.6822	0.0063	0.0517	-0.7754	0.0016	0.0161

Rplp2	-0.0407	0.8556	0.9995	-0.6066	0.0142	0.0848	-0.7760	0.0017	0.0167
Rpl21	0.1082	0.6524	0.9995	-1.0629	0.0004	0.0115	-0.7783	0.0029	0.0229
Fam193a	0.1484	0.6178	0.9995	-1.0410	0.0034	0.0362	-0.7800	0.0129	0.0622
Dlg1	0.0713	0.7522	0.9995	-0.8779	0.0015	0.0229	-0.7801	0.0021	0.0188
Gpn3	-3.2176	0.0151	0.9995	3.8029	0.0050	0.0460	-0.7802	0.3606	0.5350
Dnmt3a	-0.1328	0.6156	0.9995	-0.5186	0.0749	0.2294	-0.7812	0.0065	0.0394
Ppp1r12a	-0.1199	0.6040	0.9995	-0.9459	0.0009	0.0177	-0.7828	0.0021	0.0188
Rpl36-ps12	0.1915	0.4787	0.9995	-1.1237	0.0009	0.0177	-0.7830	0.0071	0.0417
Cd47	-0.0617	0.8283	0.9995	-0.9332	0.0050	0.0461	-0.7853	0.0090	0.0487
Gm6485	-0.1355	0.5357	0.9995	-0.6873	0.0059	0.0502	-0.7862	0.0012	0.0136
Thoc2	0.0258	0.8892	0.9995	-1.0563	0.0000	0.0030	-0.7864	0.0003	0.0058
Pik3r2	-0.2743	0.3174	0.9995	-0.1501	0.5915	0.7684	-0.7866	0.0069	0.0409
Smc5	-0.2922	0.1691	0.9995	-0.4935	0.0299	0.1320	-0.7867	0.0008	0.0103
Cnot6l	-0.1322	0.5522	0.9995	-0.7060	0.0064	0.0526	-0.7890	0.0015	0.0158
Rock2	-0.1317	0.5033	0.9995	-0.6601	0.0036	0.0375	-0.7894	0.0005	0.0074
Gli3	0.1397	0.6092	0.9995	-1.1135	0.0016	0.0233	-0.7909	0.0093	0.0497
Anp32a	0.1255	0.5858	0.9995	-0.4618	0.0597	0.2004	-0.7919	0.0020	0.0181
Ift43	0.1000	0.6999	0.9995	-0.7509	0.0115	0.0754	-0.7920	0.0057	0.0360
Rhoq	-0.2190	0.4353	0.9995	-0.7763	0.0148	0.0873	-0.7936	0.0074	0.0431
Stx17	0.1480	0.5207	0.9995	-0.8289	0.0030	0.0340	-0.7945	0.0025	0.0210
Tmx4	0.0782	0.7129	0.9995	-0.6291	0.0104	0.0705	-0.7946	0.0013	0.0138
Cdkn1b	0.1485	0.6414	0.9995	-1.1507	0.0027	0.0320	-0.7949	0.0172	0.0742
Pfdn5	-0.0815	0.7194	0.9995	-0.8607	0.0019	0.0257	-0.7950	0.0018	0.0171
Sesn1	0.2381	0.5484	0.9995	-2.0055	0.0012	0.0204	-0.7968	0.0713	0.1877
Nxn	-0.1335	0.5739	0.9995	-0.8606	0.0029	0.0332	-0.7976	0.0024	0.0208
Flii	-0.0849	0.7450	0.9995	-0.6500	0.0259	0.1216	-0.7983	0.0048	0.0322
Gstp1	0.1319	0.6099	0.9995	-0.6173	0.0277	0.1262	-0.7986	0.0041	0.0289
Mpp6	-0.0005	0.9985	0.9995	-0.7809	0.0076	0.0579	-0.7992	0.0039	0.0280
Cpe	-0.1564	0.5170	0.9995	-0.7667	0.0056	0.0489	-0.8019	0.0023	0.0202
Fibp	-0.2010	0.4449	0.9995	-0.6278	0.0359	0.1474	-0.8023	0.0050	0.0332
Clcn3	-0.2415	0.3016	0.9995	-0.5171	0.0390	0.1554	-0.8027	0.0017	0.0166
Gm9320	-0.3145	0.2353	0.9995	-0.1998	0.4578	0.6703	-0.8029	0.0043	0.0301
Cdk5rap2	-0.1908	0.4991	0.9995	-0.5379	0.0818	0.2432	-0.8034	0.0077	0.0444
Mbnl3	-0.1352	0.5561	0.9995	-0.4175	0.0839	0.2471	-0.8036	0.0016	0.0159
Dynlt3	-0.1512	0.4532	0.9995	-0.6591	0.0044	0.0421	-0.8036	0.0005	0.0077
Ptprs	0.3722	0.1082	0.9995	-1.0914	0.0002	0.0081	-0.8037	0.0018	0.0173
Kifap3	0.1197	0.6167	0.9995	-0.9149	0.0018	0.0250	-0.8039	0.0026	0.0218
Rab12	-0.0956	0.7015	0.9995	-0.6639	0.0177	0.0962	-0.8056	0.0032	0.0242
Cyb5a	-0.2817	0.3103	0.9995	-0.8053	0.0157	0.0899	-0.8069	0.0066	0.0398
Urah	-0.0600	0.8146	0.9995	0.4660	0.0756	0.2303	-0.8073	0.0049	0.0326
Maf1	-0.0685	0.7647	0.9995	-0.8997	0.0019	0.0255	-0.8080	0.0020	0.0182
Sfr1	-0.0245	0.8925	0.9995	-0.7620	0.0006	0.0141	-0.8087	0.0002	0.0042
Gstp2	0.1267	0.6274	0.9995	-0.6176	0.0290	0.1294	-0.8089	0.0040	0.0285
Dyrk1a	-0.0802	0.6976	0.9995	-0.7223	0.0037	0.0380	-0.8090	0.0008	0.0100
Tjp1	-0.4140	0.1320	0.9995	-0.0866	0.7502	0.8698	-0.8091	0.0046	0.0314
Rufy2	-0.4164	0.1471	0.9995	-0.5402	0.0836	0.2466	-0.8096	0.0064	0.0391
Ppp2r5e	-0.2923	0.2970	0.9995	-0.5001	0.0928	0.2624	-0.8100	0.0060	0.0376
Pdk3	-0.3194	0.1592	0.9995	0.2020	0.3672	0.5921	-0.8100	0.0012	0.0132
Eps15	-0.0582	0.7965	0.9995	-0.6727	0.0102	0.0698	-0.8108	0.0016	0.0161
Wdr78	0.0916	0.8151	0.9995	-1.7910	0.0026	0.0312	-0.8114	0.0579	0.1634
Ndufs4	-0.0365	0.8708	0.9995	-0.5253	0.0338	0.1425	-0.8122	0.0014	0.0148

Creb1	0.0544	0.7922	0.9995	-0.6494	0.0063	0.0519	-0.8131	0.0007	0.0090
Wls	-0.1999	0.3997	0.9995	-1.0096	0.0007	0.0157	-0.8135	0.0019	0.0177
Cask	0.1821	0.4126	0.9995	-1.1547	0.0002	0.0074	-0.8138	0.0017	0.0168
Wwp1	-0.4226	0.1216	0.9995	-0.4146	0.1496	0.3492	-0.8141	0.0043	0.0300
Zcchc7	0.2414	0.3788	0.9995	-0.9965	0.0028	0.0321	-0.8145	0.0070	0.0411
Add1	0.0237	0.9018	0.9995	-0.5755	0.0086	0.0624	-0.8159	0.0003	0.0061
Parp4	-0.2791	0.2799	0.9995	-0.5791	0.0450	0.1695	-0.8171	0.0036	0.0265
Atrx	-0.1456	0.4292	0.9995	-0.8698	0.0002	0.0080	-0.8173	0.0002	0.0041
Nek1	0.0373	0.8977	0.9995	-1.3178	0.0007	0.0155	-0.8174	0.0095	0.0505
Glb1	0.5034	0.0974	0.9995	-2.0445	0.0000	0.0021	-0.8177	0.0130	0.0625
Cpne8	0.2055	0.4523	0.9995	-1.1061	0.0011	0.0192	-0.8188	0.0056	0.0360
Slc48a1	0.3041	0.2636	0.9995	-1.0503	0.0014	0.0220	-0.8205	0.0053	0.0345
Pip4k2a	-0.2129	0.4340	0.9995	0.1337	0.6229	0.7903	-0.8209	0.0058	0.0366
Ccdc82	-0.3849	0.0784	0.9995	-0.6859	0.0053	0.0473	-0.8224	0.0006	0.0084
Cebpzos	0.2911	0.3461	0.9995	-1.5045	0.0004	0.0117	-0.8228	0.0162	0.0718
Slain2	-0.2613	0.3019	0.9995	-0.2908	0.2613	0.4863	-0.8229	0.0026	0.0218
Cwc27	-0.4169	0.1094	0.9995	-0.6454	0.0277	0.1263	-0.8241	0.0027	0.0224
Nap1l1	-0.1807	0.4314	0.9995	-0.5786	0.0202	0.1043	-0.8241	0.0012	0.0132
Cds2	-0.0801	0.7014	0.9995	-0.7292	0.0038	0.0390	-0.8241	0.0007	0.0099
Gigyf2	-0.0925	0.7531	0.9995	-0.8416	0.0127	0.0797	-0.8248	0.0085	0.0469
Gstp-ps	0.0590	0.8157	0.9995	-0.5377	0.0473	0.1745	-0.8252	0.0028	0.0226
Mif	0.3264	0.1961	0.9995	-0.7393	0.0076	0.0579	-0.8253	0.0022	0.0197
Frmd4a	-0.2576	0.3667	0.9995	-0.2392	0.4168	0.6356	-0.8256	0.0067	0.0404
Pak3	-0.0734	0.7718	0.9995	-0.6302	0.0282	0.1276	-0.8264	0.0036	0.0265
D5Ertd579e	-0.1403	0.5783	0.9995	-0.8897	0.0035	0.0372	-0.8266	0.0029	0.0233
Rsf1	-0.3035	0.2465	0.9995	-0.6967	0.0169	0.0939	-0.8275	0.0031	0.0239
Ecpas	-0.0662	0.7882	0.9995	-0.9499	0.0019	0.0257	-0.8292	0.0026	0.0218
Fer	0.1334	0.6463	0.9995	-0.9810	0.0057	0.0494	-0.8297	0.0100	0.0523
Rapgef1	0.1037	0.6060	0.9995	-1.1111	0.0001	0.0039	-0.8301	0.0004	0.0070
Fau	-0.1780	0.4791	0.9995	-0.5457	0.0416	0.1618	-0.8309	0.0023	0.0202
Gm9794	0.1144	0.6196	0.9995	-0.8763	0.0014	0.0217	-0.8319	0.0012	0.0132
Sdccag8	-0.0646	0.7863	0.9995	-0.7921	0.0058	0.0498	-0.8322	0.0021	0.0186
Eci2	-0.1403	0.5984	0.9995	-0.8683	0.0071	0.0556	-0.8323	0.0045	0.0311
Mrpl58	-0.0036	0.9902	0.9995	-0.9138	0.0094	0.0661	-0.8327	0.0087	0.0477
Vps41	-0.3703	0.2075	0.9995	-0.4173	0.1799	0.3899	-0.8345	0.0066	0.0398
Macf1	-0.3060	0.1729	0.9995	-0.3947	0.0909	0.2592	-0.8346	0.0008	0.0103
Rab26os	-0.3942	0.1114	0.9995	-0.0493	0.8410	0.9228	-0.8369	0.0017	0.0168
Sppl3	-0.1729	0.4759	0.9995	-0.7592	0.0074	0.0569	-0.8370	0.0020	0.0181
Rps11	-0.0880	0.7416	0.9995	-0.4466	0.1090	0.2877	-0.8376	0.0036	0.0265
Hmgb3	0.0599	0.7762	0.9995	-0.7417	0.0028	0.0326	-0.8384	0.0006	0.0084
Foxc1	0.0197	0.9423	0.9995	-1.1186	0.0011	0.0199	-0.8399	0.0049	0.0329
Msi2	-0.0405	0.8901	0.9995	-0.7924	0.0189	0.0999	-0.8403	0.0086	0.0472
Hmg20a	0.4012	0.2526	0.9995	-1.2879	0.0030	0.0336	-0.8406	0.0269	0.0994
Dhrs7	0.2733	0.4026	0.9995	-1.1084	0.0049	0.0455	-0.8421	0.0188	0.0784
Gnai2	-0.1172	0.5698	0.9995	-0.7102	0.0029	0.0333	-0.8428	0.0004	0.0068
Vangl1	-0.6153	0.0495	0.9995	-0.3589	0.2557	0.4802	-0.8429	0.0066	0.0398
Ywhaz	-0.3324	0.2574	0.9995	-0.3842	0.1965	0.4114	-0.8448	0.0057	0.0364
Pck2	-0.3846	0.2179	0.9995	-0.3981	0.2295	0.4520	-0.8452	0.0091	0.0490
Tlk1	-0.0616	0.7884	0.9995	-1.0255	0.0005	0.0124	-0.8458	0.0011	0.0129
Acadsb	0.0128	0.9653	0.9995	-0.3243	0.2907	0.5189	-0.8460	0.0088	0.0480
Afg3l1	-0.0352	0.8574	0.9995	-0.8629	0.0005	0.0131	-0.8477	0.0003	0.0056

Rpl26	0.0787	0.7816	0.9995	-1.0178	0.0023	0.0285	-0.8482	0.0052	0.0339
Zfp318	-0.0207	0.9270	0.9995	-1.2843	0.0001	0.0043	-0.8483	0.0011	0.0129
Sema6d	-2.0964	0.0192	0.9995	2.8133	0.0020	0.0267	-0.8498	0.1931	0.3559
Angptl2	0.1870	0.6068	0.9995	-1.6016	0.0019	0.0260	-0.8503	0.0318	0.1098
Dock9	0.3618	0.2911	0.9995	-1.6444	0.0004	0.0118	-0.8510	0.0226	0.0891
Chst14	0.2154	0.4995	0.9995	-1.2166	0.0030	0.0339	-0.8514	0.0168	0.0731
Zcchc24	0.0526	0.8964	0.9995	-1.4369	0.0050	0.0458	-0.8516	0.0447	0.1382
Ift74	-0.1243	0.5764	0.9995	-1.1405	0.0002	0.0080	-0.8530	0.0009	0.0112
Birc6	-0.1920	0.3487	0.9995	-0.6306	0.0067	0.0540	-0.8534	0.0003	0.0060
Rbl2	0.2176	0.5818	0.9995	-1.5533	0.0027	0.0320	-0.8565	0.0444	0.1375
Pqlc3	0.2664	0.4482	0.9995	-1.4585	0.0020	0.0260	-0.8570	0.0308	0.1077
Bloc1s5	-0.1521	0.6029	0.9995	-0.6881	0.0438	0.1668	-0.8572	0.0080	0.0454
Ehmt1	-0.0444	0.8254	0.9995	-0.8120	0.0011	0.0199	-0.8575	0.0003	0.0062
Tpi1	0.2034	0.5294	0.9995	-1.2237	0.0016	0.0234	-0.8582	0.0112	0.0561
Rpl27a	0.0908	0.6377	0.9995	-0.8812	0.0003	0.0096	-0.8599	0.0002	0.0041
Galnt2	-0.2931	0.2694	0.9995	-0.9100	0.0040	0.0401	-0.8608	0.0028	0.0224
Jkamp	-0.2158	0.3483	0.9995	-0.3258	0.1782	0.3882	-0.8621	0.0010	0.0120
Pttg1ip	0.2319	0.2992	0.9995	-1.0681	0.0002	0.0071	-0.8622	0.0006	0.0087
Nfix	0.0839	0.7016	0.9995	-1.2924	0.0000	0.0025	-0.8626	0.0006	0.0084
Akap7	0.1434	0.4863	0.9995	-0.8472	0.0009	0.0179	-0.8639	0.0004	0.0071
Lmna	-0.1210	0.6878	0.9995	-0.4273	0.1697	0.3768	-0.8648	0.0066	0.0399
Dcaf8	-0.1894	0.4419	0.9995	-0.6891	0.0140	0.0841	-0.8656	0.0017	0.0166
Casd1	0.0601	0.8151	0.9995	-0.7127	0.0159	0.0903	-0.8666	0.0032	0.0246
Utrn	-0.0985	0.6635	0.9995	-0.9461	0.0008	0.0167	-0.8668	0.0008	0.0104
Ppdpf	0.1274	0.6485	0.9995	-1.0562	0.0033	0.0352	-0.8670	0.0067	0.0404
Gm10051	-0.2018	0.5167	0.9995	-0.5656	0.0839	0.2471	-0.8688	0.0078	0.0445
Anxa5	-0.1102	0.6944	0.9995	-0.5170	0.0953	0.2662	-0.8697	0.0052	0.0340
Ppa2	-0.0752	0.7476	0.9995	-0.6688	0.0132	0.0816	-0.8704	0.0012	0.0136
Kdm6a	-0.2980	0.3153	0.9995	-0.3455	0.2795	0.5062	-0.8716	0.0068	0.0404
Krt18	-0.2013	0.4688	0.9995	-1.2376	0.0004	0.0119	-0.8722	0.0035	0.0261
Diaph3	-0.0731	0.7791	0.9995	-0.7686	0.0125	0.0789	-0.8732	0.0029	0.0232
Gm11478	-0.1212	0.6267	0.9995	-0.7610	0.0070	0.0551	-0.8732	0.0015	0.0158
Sorbs2	-0.7487	0.0209	0.9995	0.0699	0.8197	0.9099	-0.8735	0.0054	0.0349
Rpl8	-0.3771	0.2369	0.9995	-0.2689	0.3956	0.6167	-0.8745	0.0077	0.0444
Erbin	-0.1777	0.4181	0.9995	-0.7548	0.0037	0.0383	-0.8749	0.0006	0.0082
Rnf169	-0.1216	0.6738	0.9995	-0.7622	0.0250	0.1191	-0.8754	0.0062	0.0384
Glg1	0.1668	0.4829	0.9995	-1.1712	0.0001	0.0066	-0.8760	0.0010	0.0120
Ergic3	-0.3419	0.2148	0.9995	-0.7996	0.0120	0.0770	-0.8769	0.0030	0.0236
Serf1	0.1147	0.7154	0.9995	-1.2524	0.0021	0.0271	-0.8771	0.0119	0.0588
Sap30l	-0.2893	0.3463	0.9995	-0.7516	0.0297	0.1315	-0.8776	0.0069	0.0409
Fubp1	-0.0213	0.9115	0.9995	-1.0010	0.0001	0.0051	-0.8776	0.0001	0.0037
Gm8618	-0.3783	0.0993	0.9995	-0.4619	0.0560	0.1936	-0.8776	0.0006	0.0083
Fgfrl1	0.0425	0.8913	0.9995	-1.3352	0.0017	0.0245	-0.8776	0.0122	0.0598
Tbl1xr1	0.1865	0.4631	0.9995	-1.2426	0.0002	0.0086	-0.8793	0.0021	0.0191
Ehmt2	-0.4903	0.1176	0.9995	-0.0538	0.8614	0.9340	-0.8794	0.0061	0.0381
Senp6	-0.1167	0.5984	0.9995	-0.7839	0.0030	0.0336	-0.8795	0.0006	0.0085
Zmym2	-0.0602	0.7643	0.9995	-0.5682	0.0140	0.0841	-0.8811	0.0003	0.0058
Phlpp1	0.0229	0.9248	0.9995	-1.0161	0.0015	0.0231	-0.8821	0.0020	0.0183
Tnrc18	-0.2639	0.2668	0.9995	-0.9568	0.0012	0.0203	-0.8834	0.0009	0.0112
Samd1	-0.1507	0.5782	0.9995	-0.3824	0.1814	0.3918	-0.8844	0.0034	0.0254
Cluap1	0.0287	0.9251	0.9995	-0.9953	0.0080	0.0602	-0.8858	0.0084	0.0467

Gsk3b	-0.2087	0.3174	0.9995	-0.4503	0.0425	0.1639	-0.8876	0.0003	0.0053
Maml2	0.2422	0.3330	0.9995	-1.1251	0.0007	0.0158	-0.8881	0.0025	0.0212
Ncor1	-0.0583	0.7700	0.9995	-1.1165	0.0000	0.0032	-0.8883	0.0002	0.0042
Cyp4v3	0.0792	0.8249	0.9995	-2.1467	0.0004	0.0121	-0.8888	0.0263	0.0978
Dgkd	-0.2407	0.3943	0.9995	-0.4424	0.1376	0.3323	-0.8889	0.0037	0.0271
Rhobtb3	-0.0803	0.7667	0.9995	-0.7941	0.0111	0.0735	-0.8891	0.0031	0.0239
Gdpd1	0.0229	0.9398	0.9995	-0.5141	0.1139	0.2959	-0.8894	0.0079	0.0452
Fbxo11	-0.1894	0.3718	0.9995	-0.5781	0.0163	0.0918	-0.8897	0.0004	0.0064
Ctcf	-0.1375	0.6197	0.9995	-0.9032	0.0050	0.0459	-0.8903	0.0031	0.0239
Ckap5	-0.2891	0.2761	0.9995	-0.3810	0.1636	0.3698	-0.8912	0.0020	0.0183
2610002M06Rik	0.2083	0.4203	0.9995	-1.2161	0.0004	0.0121	-0.8915	0.0027	0.0222
G2e3	-0.2545	0.3895	0.9995	-0.4349	0.1578	0.3616	-0.8918	0.0047	0.0319
Myo1e	-0.2239	0.3882	0.9995	-0.6934	0.0214	0.1078	-0.8920	0.0021	0.0189
Sorl1	-0.2476	0.4284	0.9995	-1.1657	0.0041	0.0405	-0.8928	0.0083	0.0463
Dennd1b	-0.0602	0.8152	0.9995	-0.6798	0.0222	0.1104	-0.8938	0.0025	0.0212
Gm9385	-0.3045	0.2875	0.9995	-0.2875	0.3161	0.5421	-0.8940	0.0033	0.0250
Btbd7	-0.0732	0.7944	0.9995	-0.6359	0.0436	0.1666	-0.8942	0.0042	0.0294
Ltbp3	0.1764	0.6370	0.9995	-1.5387	0.0015	0.0226	-0.8947	0.0252	0.0956
Npepps	0.0715	0.8111	0.9995	-1.0256	0.0037	0.0383	-0.8951	0.0056	0.0357
Krtcap3	0.5179	0.0967	0.9995	-1.3382	0.0005	0.0130	-0.8966	0.0077	0.0444
Trim37	-0.2664	0.3664	0.9995	-0.3833	0.2073	0.4246	-0.8966	0.0044	0.0302
Bbx	-0.1885	0.3368	0.9995	-0.8343	0.0006	0.0142	-0.8974	0.0001	0.0036
Gnpat	-0.1093	0.6607	0.9995	-0.7286	0.0129	0.0808	-0.8983	0.0017	0.0169
Wdr36	0.3566	0.1449	0.9995	-1.2275	0.0001	0.0051	-0.8989	0.0009	0.0108
Pbx2	0.0345	0.8871	0.9995	-0.7578	0.0090	0.0645	-0.8992	0.0016	0.0162
Hsp90ab1	-0.1204	0.6620	0.9995	-0.8678	0.0054	0.0481	-0.8996	0.0026	0.0216
Tmem141	0.2173	0.5754	0.9995	-1.4466	0.0043	0.0414	-0.9004	0.0367	0.1204
Ralgapa1	-0.0179	0.9383	0.9995	-0.5945	0.0233	0.1143	-0.9014	0.0009	0.0113
Lrrcc1	-0.0338	0.9136	0.9995	-1.2001	0.0019	0.0258	-0.9015	0.0072	0.0420
Actb	-0.3631	0.2152	0.9995	-0.1208	0.6737	0.8209	-0.9016	0.0034	0.0257
Mcm9	0.2602	0.5433	0.9995	-1.7073	0.0055	0.0485	-0.9017	0.0609	0.1689
Trip12	-0.2761	0.2213	0.9995	-0.7842	0.0026	0.0315	-0.9040	0.0004	0.0069
Ap2b1	-0.2108	0.3632	0.9995	-0.7081	0.0072	0.0561	-0.9046	0.0006	0.0085
Rala	-0.2509	0.3267	0.9995	-0.7234	0.0111	0.0732	-0.9066	0.0013	0.0140
Mcrip1	-0.3558	0.1877	0.9995	-1.1182	0.0020	0.0261	-0.9096	0.0023	0.0198
Cdc14a	-0.4314	0.1879	0.9995	-1.1950	0.0058	0.0499	-0.9101	0.0083	0.0466
Rnf38	-0.3361	0.3058	0.9995	-1.2488	0.0046	0.0438	-0.9107	0.0094	0.0504
Mkl2	-0.1851	0.4643	0.9995	-0.7905	0.0088	0.0633	-0.9112	0.0016	0.0160
Dlg5	-0.3460	0.2587	0.9995	-0.4881	0.1312	0.3220	-0.9113	0.0050	0.0331
Adarb1	-0.4261	0.1959	0.9995	-0.1309	0.6989	0.8380	-0.9115	0.0085	0.0470
Cyb561	-0.1392	0.6196	0.9995	-1.2917	0.0012	0.0201	-0.9115	0.0042	0.0294
Cuedc1	0.2004	0.4713	0.9995	-1.1140	0.0018	0.0249	-0.9124	0.0049	0.0325
Prrc1	-0.1305	0.6639	0.9995	-0.4200	0.1968	0.4115	-0.9125	0.0068	0.0404
Sptbn1	-0.2589	0.3075	0.9995	-0.4916	0.0639	0.2072	-0.9136	0.0011	0.0125
Akap9	-0.1209	0.6527	0.9995	-1.0760	0.0010	0.0186	-0.9146	0.0020	0.0182
Npr3	-0.0250	0.9361	0.9995	-0.6185	0.0755	0.2302	-0.9147	0.0084	0.0467
Capg	-0.2567	0.3826	0.9995	-0.2903	0.3318	0.5571	-0.9153	0.0037	0.0272
Nav1	-0.3807	0.1247	0.9995	-0.2216	0.3749	0.5991	-0.9159	0.0008	0.0106
Rpl14	-0.2260	0.3623	0.9995	-0.4847	0.0623	0.2052	-0.9166	0.0009	0.0112
S100a6	0.0529	0.7819	0.9995	-0.8373	0.0004	0.0117	-0.9186	0.0001	0.0026
Dhx40	-0.0222	0.9221	0.9995	-1.1500	0.0002	0.0076	-0.9193	0.0006	0.0084

Plekha5	-0.2108	0.4889	0.9995	-1.1221	0.0052	0.0470	-0.9222	0.0065	0.0393
Golph3l	-0.0468	0.8713	0.9995	-0.4951	0.1135	0.2954	-0.9253	0.0047	0.0320
Lpgat1	0.2123	0.4067	0.9995	-0.7247	0.0110	0.0732	-0.9272	0.0013	0.0139
Plbd2	0.1999	0.5119	0.9995	-1.2737	0.0011	0.0198	-0.9291	0.0061	0.0377
Myo5b	-0.3229	0.4096	0.9995	-1.9663	0.0020	0.0268	-0.9295	0.0253	0.0957
Rnf217	-0.1671	0.5329	0.9995	-0.6986	0.0243	0.1173	-0.9296	0.0022	0.0196
Pank2	-0.3930	0.1702	0.9995	-0.2364	0.4219	0.6401	-0.9304	0.0026	0.0218
Lipa	0.0603	0.8261	0.9995	-1.2458	0.0008	0.0168	-0.9318	0.0035	0.0263
Calcoco1	0.0918	0.7952	0.9995	-1.3164	0.0031	0.0345	-0.9327	0.0153	0.0692
Fndc3b	-0.3378	0.2380	0.9995	-0.2050	0.4787	0.6866	-0.9330	0.0026	0.0219
Asap1	-0.0429	0.8397	0.9995	-1.1970	0.0000	0.0032	-0.9334	0.0002	0.0047
Srbd1	0.0701	0.7948	0.9995	-1.3263	0.0003	0.0111	-0.9369	0.0025	0.0213
Ltbp1	-0.1249	0.6261	0.9995	-0.5894	0.0375	0.1516	-0.9373	0.0013	0.0141
Epb41l4b	-0.4660	0.0940	0.9995	-0.6041	0.0577	0.1965	-0.9383	0.0018	0.0171
Rabac1	0.1621	0.4773	0.9995	-0.7695	0.0043	0.0418	-0.9385	0.0006	0.0087
Scara3	-0.5484	0.0829	0.9995	-0.5241	0.1254	0.3142	-0.9393	0.0042	0.0294
Zfp608	-0.1242	0.6318	0.9995	-0.7515	0.0131	0.0810	-0.9395	0.0015	0.0157
Cic	-0.5151	0.1032	0.9995	-0.3073	0.3421	0.5679	-0.9397	0.0042	0.0296
Fbxo3	0.2673	0.2620	0.9995	-1.0081	0.0007	0.0156	-0.9400	0.0009	0.0109
Gm15772	-0.1251	0.6384	0.9995	-0.8794	0.0040	0.0401	-0.9405	0.0014	0.0150
Nfib	0.2512	0.2378	0.9995	-0.9886	0.0002	0.0081	-0.9443	0.0002	0.0039
Pds5b	0.0365	0.8672	0.9995	-0.9560	0.0005	0.0130	-0.9446	0.0003	0.0056
C230062I16Rik	0.2221	0.3422	0.9995	-0.7027	0.0070	0.0552	-0.9451	0.0004	0.0069
Eif4b	0.1799	0.3898	0.9995	-1.1833	0.0000	0.0029	-0.9464	0.0001	0.0037
Rpl10a	0.1295	0.5874	0.9995	-0.9835	0.0007	0.0154	-0.9468	0.0005	0.0080
Itsn1	-0.1067	0.7530	0.9995	-0.7727	0.0422	0.1633	-0.9488	0.0092	0.0497
Eif3h	-0.0647	0.8040	0.9995	-0.7197	0.0133	0.0818	-0.9511	0.0012	0.0130
Saal1	-0.3186	0.3166	0.9995	-0.1416	0.6684	0.8178	-0.9519	0.0060	0.0374
Gm3650	-0.1008	0.7095	0.9995	-0.6628	0.0323	0.1388	-0.9520	0.0022	0.0197
Ier3	0.2600	0.3476	0.9995	-0.3791	0.1782	0.3882	-0.9521	0.0017	0.0169
Capn5	0.0169	0.9512	0.9995	-0.8952	0.0078	0.0592	-0.9523	0.0031	0.0238
Kdelc2	0.2041	0.4759	0.9995	-1.4753	0.0002	0.0088	-0.9525	0.0038	0.0276
Gm15501	-0.2260	0.3276	0.9995	-0.5802	0.0201	0.1037	-0.9527	0.0003	0.0061
Dock5	0.3472	0.1838	0.9995	-0.8925	0.0033	0.0355	-0.9535	0.0015	0.0155
Pdlim2	-0.2045	0.5417	0.9995	-1.4688	0.0007	0.0154	-0.9546	0.0073	0.0424
Arrdc1	-0.3214	0.2563	0.9995	-0.8165	0.0138	0.0833	-0.9554	0.0022	0.0194
Rpl29	-0.2126	0.4888	0.9995	-0.5365	0.0952	0.2661	-0.9557	0.0037	0.0271
Sh3kbp1	-0.3708	0.1197	0.9995	-0.4799	0.0605	0.2018	-0.9560	0.0004	0.0071
Eef2kmt	0.5686	0.1565	0.9995	-2.0929	0.0004	0.0116	-0.9564	0.0312	0.1086
Ngef	1.0179	0.0588	0.9995	-2.5619	0.0005	0.0131	-0.9581	0.1112	0.2523
Csad	-0.2971	0.3385	0.9995	-0.4387	0.1896	0.4020	-0.9588	0.0047	0.0319
Ube2q2	-0.2524	0.2938	0.9995	-0.7701	0.0063	0.0521	-0.9590	0.0005	0.0081
Gale	0.0786	0.8067	0.9995	-1.1925	0.0029	0.0330	-0.9591	0.0068	0.0405
Hacd4	-0.5247	0.0550	0.9995	-1.5898	0.0001	0.0053	-0.9618	0.0010	0.0120
Vps51	-0.3420	0.2963	0.9995	-0.7359	0.0459	0.1715	-0.9619	0.0060	0.0376
Srxn1	0.3716	0.2528	0.9995	-1.7326	0.0001	0.0066	-0.9627	0.0076	0.0440
Rps11-ps1	-0.2036	0.5071	0.9995	-0.4060	0.1965	0.4114	-0.9639	0.0035	0.0258
Pdlim7	-0.1594	0.5235	0.9995	-0.5683	0.0396	0.1571	-0.9643	0.0008	0.0105
Nudt14	-0.0621	0.7889	0.9995	-1.1946	0.0003	0.0103	-0.9652	0.0006	0.0088
Ptpru	-0.3878	0.5085	0.9995	-4.4529	0.0017	0.0246	-0.9652	0.1136	0.2555
Nav2	-0.6384	0.0335	0.9995	-0.4571	0.1447	0.3426	-0.9656	0.0018	0.0171

Tnks	-0.2812	0.4120	0.9995	-0.4388	0.2313	0.4545	-0.9661	0.0084	0.0467
Ing4	-0.3610	0.2004	0.9995	-0.6177	0.0524	0.1860	-0.9666	0.0019	0.0179
Camk2d	-0.1369	0.6609	0.9995	-0.7009	0.0440	0.1670	-0.9689	0.0049	0.0326
Zfyve16	-0.2278	0.4647	0.9995	-1.0339	0.0089	0.0639	-0.9689	0.0053	0.0346
A930004D18Rik	-0.3865	0.1787	0.9995	0.1525	0.5937	0.7700	-0.9695	0.0023	0.0202
Rps14	0.1489	0.5833	0.9995	-0.9501	0.0026	0.0309	-0.9705	0.0013	0.0139
Pdzrn3	-0.1157	0.6854	0.9995	-0.3113	0.2976	0.5242	-0.9705	0.0028	0.0226
Usp3	-0.1380	0.6449	0.9995	-1.1204	0.0032	0.0349	-0.9738	0.0036	0.0266
Pak1	0.1871	0.4885	0.9995	-0.9708	0.0030	0.0333	-0.9756	0.0017	0.0168
Chchd5	-0.2470	0.3645	0.9995	-0.8571	0.0102	0.0696	-0.9763	0.0017	0.0169
Mir682	0.0940	0.7091	0.9995	-0.7860	0.0061	0.0510	-0.9764	0.0007	0.0092
Bmp1	-0.3547	0.1346	0.9995	-0.7773	0.0055	0.0482	-0.9768	0.0003	0.0062
Pola1	-0.1897	0.5333	0.9995	-0.6214	0.0600	0.2009	-0.9769	0.0034	0.0253
Cop1	0.0236	0.9029	0.9995	-0.8286	0.0007	0.0155	-0.9770	0.0001	0.0024
Itgav	-0.2223	0.4810	0.9995	-0.2190	0.4973	0.6992	-0.9771	0.0046	0.0313
Inf2	0.0429	0.8574	0.9995	-0.8283	0.0039	0.0394	-0.9775	0.0006	0.0084
Sh3pxd2b	-0.1642	0.4159	0.9995	-0.7414	0.0023	0.0289	-0.9780	0.0001	0.0027
Sclt1	-0.2625	0.3383	0.9995	-1.3733	0.0005	0.0130	-0.9780	0.0018	0.0170
Krt7	-0.2447	0.1824	0.9995	-0.6466	0.0021	0.0272	-0.9794	0.0000	0.0011
Ttc21b	-0.3591	0.2553	0.9995	-1.3069	0.0025	0.0306	-0.9796	0.0046	0.0313
Lyrm2	-0.2111	0.3831	0.9995	-0.4932	0.0638	0.2072	-0.9801	0.0006	0.0084
Ivd	-0.2794	0.3677	0.9995	-0.9988	0.0110	0.0730	-0.9806	0.0043	0.0298
Man2c1	-0.2234	0.4142	0.9995	-0.8353	0.0130	0.0808	-0.9816	0.0019	0.0179
Vps13a	-0.1954	0.4171	0.9995	-0.6753	0.0134	0.0822	-0.9816	0.0005	0.0074
Ppp2r3a	-0.0903	0.6817	0.9995	-0.9130	0.0012	0.0201	-0.9820	0.0003	0.0055
0610010F05Rik	-0.1536	0.5837	0.9995	-0.9187	0.0077	0.0582	-0.9825	0.0023	0.0201
Plpp2	-0.1584	0.5972	0.9995	-0.8579	0.0121	0.0777	-0.9827	0.0028	0.0228
Rasa1	-0.6265	0.0223	0.9995	-0.2441	0.3474	0.5736	-0.9833	0.0006	0.0087
Irak4	-0.0253	0.9390	0.9995	-0.8266	0.0329	0.1402	-0.9838	0.0082	0.0459
Socs2	0.2891	0.2624	0.9995	-1.1613	0.0004	0.0121	-0.9840	0.0012	0.0131
Thra	-0.5216	0.0759	0.9995	-0.6240	0.0604	0.2018	-0.9855	0.0018	0.0175
Rpl17	-0.0201	0.9270	0.9995	-1.3143	0.0000	0.0022	-0.9891	0.0002	0.0040
Plcb3	-0.0049	0.9880	0.9995	-0.6952	0.0532	0.1878	-0.9933	0.0057	0.0363
Lrrfip1	-0.1725	0.5604	0.9995	-0.9668	0.0049	0.0454	-0.9940	0.0022	0.0197
Cox20	0.1360	0.5634	0.9995	-1.4452	0.0000	0.0025	-0.9965	0.0004	0.0065
Cplane1	-0.3124	0.2753	0.9995	-0.5537	0.0733	0.2255	-0.9970	0.0017	0.0167
Elk3	0.0555	0.7860	0.9995	-1.1830	0.0000	0.0032	-0.9974	0.0001	0.0028
Wdfy3	-0.1192	0.5952	0.9995	-0.6853	0.0089	0.0641	-0.9976	0.0003	0.0053
2610528A11Rik	0.1866	0.7984	0.9995	-4.0174	0.0046	0.0438	-0.9984	0.2194	0.3867
Rpl3	0.2205	0.2887	0.9995	-1.4640	0.0000	0.0008	-1.0007	0.0001	0.0024
Lamc1	-0.1375	0.5879	0.9995	-0.7208	0.0125	0.0791	-1.0021	0.0007	0.0091
Zfp523	0.0623	0.8510	0.9995	-1.3852	0.0025	0.0306	-1.0033	0.0101	0.0528
Atxn10	-0.2997	0.2850	0.9995	-0.5559	0.0632	0.2061	-1.0037	0.0013	0.0137
Rpl23a	-0.1649	0.4602	0.9995	-0.5531	0.0220	0.1099	-1.0044	0.0002	0.0038
Dnajb14	-0.5147	0.0798	0.9995	-0.2032	0.4929	0.6962	-1.0062	0.0014	0.0150
Dele1	-0.2394	0.4671	0.9995	-0.9700	0.0181	0.0974	-1.0078	0.0057	0.0363
4930523C07Rik	-0.4015	0.2279	0.9995	-1.2771	0.0034	0.0362	-1.0083	0.0052	0.0340
Trerf1	-0.3139	0.5608	0.9995	-2.5051	0.0058	0.0499	-1.0084	0.0774	0.1986
Grcc10	-0.3977	0.1880	0.9995	-0.6270	0.0518	0.1844	-1.0084	0.0020	0.0182
Ehbp1	-0.4819	0.0870	0.9995	-0.1492	0.6024	0.7756	-1.0096	0.0011	0.0126
Cobl	-0.0664	0.8271	0.9995	-0.3765	0.2469	0.4715	-1.0119	0.0039	0.0282

Wnk1	-0.1388	0.5309	0.9995	-0.8738	0.0010	0.0191	-1.0120	0.0001	0.0036
Tgfa	-0.1041	0.7553	0.9995	-0.6571	0.0846	0.2483	-1.0121	0.0089	0.0484
Myh10	-0.1305	0.5337	0.9995	-0.7881	0.0016	0.0234	-1.0138	0.0001	0.0025
Slc26a10	0.9324	0.2277	0.9995	-4.2281	0.0008	0.0162	-1.0151	0.2240	0.3927
Tcf4	-0.1218	0.5301	0.9995	-1.0296	0.0001	0.0043	-1.0152	0.0000	0.0015
Micu1	0.1508	0.5297	0.9995	-1.0386	0.0007	0.0151	-1.0159	0.0004	0.0072
Ica1	0.1349	0.6299	0.9995	-1.3934	0.0005	0.0126	-1.0172	0.0026	0.0216
Chmp2a	-0.3096	0.2248	0.9995	-1.3530	0.0002	0.0078	-1.0181	0.0006	0.0084
Rpl36	0.0042	0.9865	0.9995	-0.9770	0.0012	0.0205	-1.0182	0.0005	0.0073
Fam168a	-0.0980	0.6780	0.9995	-0.7377	0.0074	0.0569	-1.0188	0.0003	0.0060
Bcr	-0.2606	0.4024	0.9995	-0.4319	0.1915	0.4044	-1.0191	0.0031	0.0239
Dstn	-0.3139	0.3274	0.9995	-0.1489	0.6393	0.7988	-1.0192	0.0030	0.0233
Homer1	0.0123	0.9645	0.9995	-1.2398	0.0010	0.0186	-1.0212	0.0018	0.0175
Grhpr	-0.0775	0.8053	0.9995	-1.1160	0.0057	0.0495	-1.0226	0.0045	0.0309
Ank3	-0.4850	0.0723	0.9995	-0.6931	0.0222	0.1104	-1.0228	0.0006	0.0089
Epb41	-0.3993	0.1496	0.9995	-0.6704	0.0288	0.1290	-1.0256	0.0009	0.0110
Plec	-0.1084	0.7290	0.9995	-0.6400	0.0559	0.1935	-1.0265	0.0027	0.0221
Pisd-ps1	-0.4267	0.1174	0.9995	-0.8868	0.0078	0.0587	-1.0267	0.0008	0.0105
Ppl	0.3174	0.1872	0.9995	-1.3559	0.0000	0.0034	-1.0272	0.0003	0.0061
Naxe	-0.0346	0.8979	0.9995	-0.9300	0.0041	0.0405	-1.0280	0.0010	0.0120
Zfp30	0.2642	0.4093	0.9995	-1.6400	0.0004	0.0118	-1.0285	0.0060	0.0376
Gm5453	-0.0906	0.6710	0.9995	-0.9463	0.0004	0.0113	-1.0310	0.0001	0.0025
Pard3	0.1407	0.5026	0.9995	-1.0366	0.0002	0.0072	-1.0317	0.0001	0.0026
Hk1	-0.1259	0.6422	0.9995	-1.0329	0.0019	0.0256	-1.0323	0.0009	0.0111
Lamb2	-0.4495	0.1933	0.9995	-0.5684	0.1287	0.3183	-1.0330	0.0049	0.0325
Pcif1	0.7722	0.0996	0.9995	-2.0208	0.0016	0.0236	-1.0340	0.0618	0.1704
Dip2b	-0.5867	0.0558	0.9995	-0.4804	0.1427	0.3393	-1.0346	0.0015	0.0157
Ttc3	-0.0988	0.6498	0.9995	-1.1702	0.0001	0.0040	-1.0349	0.0001	0.0028
Siae	-0.0041	0.9867	0.9995	-1.1757	0.0004	0.0111	-1.0350	0.0005	0.0075
Gm6023	-0.2332	0.5031	0.9995	-0.3765	0.3195	0.5461	-1.0358	0.0074	0.0428
Prkar2b	0.2156	0.3762	0.9995	-0.7323	0.0081	0.0605	-1.0374	0.0004	0.0068
Pdgfra	-0.1370	0.6886	0.9995	-1.0204	0.0159	0.0903	-1.0383	0.0074	0.0428
Igbp1	-0.1659	0.5463	0.9995	-1.3064	0.0005	0.0130	-1.0387	0.0012	0.0134
Nek7	-0.2900	0.1877	0.9995	-0.4467	0.0570	0.1950	-1.0403	0.0001	0.0028
Met	-0.1127	0.5525	0.9995	-0.6647	0.0027	0.0321	-1.0419	0.0000	0.0011
Fam171a1	0.0863	0.7018	0.9995	-0.8943	0.0014	0.0217	-1.0430	0.0002	0.0042
Usf3	-0.0628	0.7596	0.9995	-0.9859	0.0003	0.0109	-1.0442	0.0001	0.0025
H2afx	0.1953	0.5563	0.9995	-1.7327	0.0002	0.0083	-1.0459	0.0056	0.0357
Phpt1	0.1406	0.5734	0.9995	-1.1494	0.0003	0.0109	-1.0459	0.0004	0.0068
Pbrm1	-0.0215	0.9094	0.9995	-1.0671	0.0000	0.0031	-1.0475	0.0000	0.0011
Dcbld2	-0.0524	0.8084	0.9995	-0.6020	0.0126	0.0794	-1.0479	0.0001	0.0026
Prmt2	-0.4497	0.1865	0.9995	-0.1641	0.6374	0.7978	-1.0493	0.0041	0.0288
Exoc4	0.2508	0.4820	0.9995	-1.4736	0.0022	0.0276	-1.0496	0.0096	0.0510
Ulk2	0.2638	0.5014	0.9995	-1.3961	0.0043	0.0418	-1.0507	0.0177	0.0754
B4gat1	0.0729	0.8219	0.9995	-1.3332	0.0020	0.0268	-1.0508	0.0047	0.0319
Itgb4	-0.0482	0.8376	0.9995	-1.3382	0.0001	0.0047	-1.0516	0.0003	0.0053
Furin	-0.3743	0.2680	0.9995	-0.6151	0.0877	0.2536	-1.0526	0.0037	0.0269
Igf2r	-0.4166	0.0942	0.9995	-0.5187	0.0483	0.1767	-1.0528	0.0002	0.0048
Wee1	-0.4513	0.1644	0.9995	-0.4843	0.1559	0.3584	-1.0535	0.0027	0.0219
Htra1	-0.0073	0.9715	0.9995	-1.0315	0.0001	0.0064	-1.0541	0.0000	0.0018
Padi4	0.0349	0.9183	0.9995	-1.0492	0.0086	0.0624	-1.0551	0.0052	0.0342

Atp11b	-0.2929	0.2468	0.9995	-0.7076	0.0134	0.0820	-1.0552	0.0003	0.0062
Notch1	-0.5900	0.0529	0.9995	-0.8029	0.0226	0.1118	-1.0558	0.0013	0.0138
5730596B20Rik	-0.1415	0.8387	0.9995	-5.3055	0.0010	0.0191	-1.0565	0.1526	0.3071
Col4a3bp	-0.2486	0.3264	0.9995	-0.8148	0.0075	0.0575	-1.0588	0.0005	0.0072
Cryz	0.2316	0.5043	0.9995	-1.7145	0.0005	0.0130	-1.0589	0.0077	0.0444
Eml4	-0.3375	0.1636	0.9995	-0.3252	0.1928	0.4064	-1.0599	0.0002	0.0046
Sccpdh	-0.1075	0.6847	0.9995	-0.5311	0.0691	0.2171	-1.0603	0.0008	0.0103
Atrnl1	0.4287	0.3257	0.9995	-2.1092	0.0007	0.0154	-1.0616	0.0335	0.1138
Rai14	-0.2175	0.4209	0.9995	-0.4106	0.1447	0.3426	-1.0616	0.0006	0.0086
Osbpl1a	-0.2467	0.4292	0.9995	-0.5906	0.0926	0.2623	-1.0629	0.0028	0.0224
Usp34	-0.1120	0.6060	0.9995	-0.9449	0.0005	0.0130	-1.0654	0.0001	0.0024
Setd5	-0.0326	0.8875	0.9995	-1.2614	0.0001	0.0043	-1.0658	0.0001	0.0037
Map3k9	-0.4452	0.1690	0.9995	-0.4246	0.2260	0.4481	-1.0663	0.0028	0.0224
Mmgt2	-0.0628	0.8075	0.9995	-0.7113	0.0193	0.1011	-1.0676	0.0007	0.0091
Tbc1d5	-0.0918	0.7644	0.9995	-1.1519	0.0048	0.0450	-1.0676	0.0029	0.0232
Syngr1	-0.3516	0.3259	0.9995	-1.4962	0.0015	0.0231	-1.0692	0.0055	0.0353
Pbx1	-0.1402	0.5962	0.9995	-0.9511	0.0031	0.0340	-1.0697	0.0006	0.0085
Arfgap3	-0.2156	0.5167	0.9995	-0.3078	0.3837	0.6066	-1.0700	0.0048	0.0322
Pik3ip1	0.1338	0.7185	0.9995	-1.5311	0.0043	0.0419	-1.0722	0.0151	0.0689
Myof	0.0888	0.6174	0.9995	-0.9121	0.0001	0.0051	-1.0722	0.0000	0.0006
Myo9a	-0.2136	0.3990	0.9995	-0.8255	0.0062	0.0514	-1.0730	0.0004	0.0064
Arhgap21	-0.3748	0.1188	0.9995	-0.5748	0.0263	0.1226	-1.0734	0.0001	0.0036
Anxa6	-0.4349	0.2744	0.9995	-0.3417	0.3928	0.6141	-1.0742	0.0090	0.0487
Ahnak	-0.4399	0.0663	0.9995	-0.6316	0.0124	0.0789	-1.0751	0.0001	0.0028
Vezt	-0.0501	0.8445	0.9995	-1.1157	0.0011	0.0192	-1.0762	0.0006	0.0084
Bmpr1a	-0.0839	0.7597	0.9995	-1.0822	0.0019	0.0255	-1.0772	0.0009	0.0110
Ppp3cb	-0.1829	0.5613	0.9995	-0.8422	0.0217	0.1089	-1.0773	0.0028	0.0224
Fbxl19	0.3590	0.2349	0.9995	-0.8064	0.0192	0.1009	-1.0783	0.0038	0.0272
Lcorl	-0.1852	0.5518	0.9995	-0.7081	0.0462	0.1723	-1.0809	0.0024	0.0206
Nacc2	0.6920	0.1074	0.9995	-2.1297	0.0012	0.0205	-1.0811	0.0351	0.1168
Rpl5-ps2	-0.1713	0.5316	0.9995	-0.6658	0.0249	0.1190	-1.0835	0.0005	0.0081
Gm8451	-0.1671	0.5221	0.9995	-0.6715	0.0185	0.0988	-1.0841	0.0003	0.0062
S100a4	0.3471	0.1738	0.9995	-1.3178	0.0001	0.0043	-1.0845	0.0002	0.0049
Gm10045	-0.1871	0.5420	0.9995	-1.1985	0.0018	0.0255	-1.0846	0.0018	0.0171
Asah2	-0.3092	0.3368	0.9995	-0.8290	0.0291	0.1296	-1.0858	0.0027	0.0222
Ids	-0.1062	0.7254	0.9995	-0.9607	0.0095	0.0663	-1.0882	0.0020	0.0183
Igsf3	0.3624	0.2821	0.9995	-1.3696	0.0015	0.0226	-1.0894	0.0054	0.0350
Fam117b	-0.0422	0.8589	0.9995	-0.9633	0.0013	0.0213	-1.0899	0.0002	0.0047
Elmo3	-0.0942	0.7866	0.9995	-1.6883	0.0010	0.0184	-1.0904	0.0065	0.0394
Gm13232	-0.5711	0.1564	0.9995	-0.2167	0.5833	0.7631	-1.0905	0.0079	0.0453
Zfp609	-0.3598	0.2879	0.9995	-0.6848	0.0710	0.2213	-1.0906	0.0034	0.0256
Gm13167	-0.5695	0.1576	0.9995	-0.2175	0.5820	0.7626	-1.0910	0.0079	0.0453
Arid2	0.0790	0.8039	0.9995	-1.7731	0.0001	0.0067	-1.0913	0.0031	0.0238
Gmds	-0.2199	0.5005	0.9995	-0.3695	0.2931	0.5209	-1.0918	0.0038	0.0275
Sord	0.0050	0.9867	0.9995	-1.7886	0.0002	0.0075	-1.0923	0.0026	0.0217
Vars2	0.1473	0.6693	0.9995	-1.3729	0.0045	0.0430	-1.0928	0.0088	0.0479
Sestd1	-0.4465	0.1315	0.9995	-0.5869	0.0605	0.2020	-1.0931	0.0008	0.0105
Hoga1	-0.4816	0.2589	0.9995	-1.8626	0.0040	0.0402	-1.0942	0.0161	0.0714
Sema5a	-0.2022	0.4891	0.9995	-0.5605	0.0931	0.2631	-1.0947	0.0015	0.0156
Chd6	0.0354	0.8866	0.9995	-0.8097	0.0063	0.0518	-1.0948	0.0003	0.0062
Cast	-0.1052	0.5828	0.9995	-0.8741	0.0003	0.0106	-1.0950	0.0000	0.0010

Ephx1	1.4271	0.0030	0.9995	-2.5105	0.0001	0.0051	-1.0975	0.0445	0.1378
Fundc2	0.2237	0.3603	0.9995	-1.3079	0.0001	0.0041	-1.0976	0.0002	0.0040
Fmn1	-0.0639	0.8183	0.9995	-1.3518	0.0005	0.0130	-1.0982	0.0010	0.0117
Cd99l2	-0.1793	0.5704	0.9995	-0.4123	0.2194	0.4395	-1.0987	0.0023	0.0202
Arrb1	-0.0333	0.9581	0.9995	-3.3524	0.0054	0.0477	-1.0988	0.1131	0.2549
Tmtc4	-0.5005	0.1446	0.9995	-0.3288	0.3540	0.5806	-1.0991	0.0030	0.0233
Zcchc3	-0.5217	0.1657	0.9995	-0.2111	0.5956	0.7711	-1.0991	0.0062	0.0383
Cd151	0.0779	0.7110	0.9995	-0.9940	0.0002	0.0092	-1.1005	0.0000	0.0018
Mms19	-0.1508	0.5948	0.9995	-1.1322	0.0019	0.0258	-1.1013	0.0009	0.0114
Zfp606	-0.2259	0.5013	0.9995	-2.1634	0.0002	0.0086	-1.1028	0.0045	0.0309
Phyhd1	-0.2186	0.5430	0.9995	-1.3048	0.0085	0.0618	-1.1041	0.0071	0.0417
Ncoa7	0.0638	0.7638	0.9995	-0.9686	0.0005	0.0124	-1.1042	0.0001	0.0024
Insr	-0.1019	0.7126	0.9995	-1.3454	0.0006	0.0147	-1.1054	0.0009	0.0112
Arid1b	0.0769	0.7344	0.9995	-1.5072	0.0000	0.0021	-1.1059	0.0001	0.0034
Tcf7l2	0.0771	0.6958	0.9995	-0.9540	0.0002	0.0087	-1.1073	0.0000	0.0012
Tns4	0.4829	0.1148	0.9995	-0.7720	0.0173	0.0952	-1.1082	0.0010	0.0117
Lrrk1	-0.3723	0.1395	0.9995	-0.7141	0.0122	0.0780	-1.1121	0.0002	0.0042
Lad1	-0.4613	0.0710	0.9995	-0.6768	0.0149	0.0873	-1.1128	0.0002	0.0038
Phf14	0.0037	0.9849	0.9995	-1.4374	0.0000	0.0009	-1.1133	0.0000	0.0010
Ccny	-0.1280	0.5795	0.9995	-0.8304	0.0037	0.0385	-1.1137	0.0001	0.0037
Wdr83os	0.2509	0.3198	0.9995	-0.9426	0.0019	0.0260	-1.1153	0.0004	0.0063
Pcyt1b	-0.0382	0.8881	0.9995	-1.0194	0.0025	0.0301	-1.1191	0.0006	0.0083
Lrrc1	-0.2451	0.3892	0.9995	-0.9632	0.0069	0.0549	-1.1199	0.0009	0.0113
Hspa8	-0.2510	0.4092	0.9995	-0.6933	0.0338	0.1424	-1.1202	0.0010	0.0116
Phf21a	-0.1425	0.6021	0.9995	-1.0079	0.0035	0.0372	-1.1213	0.0007	0.0092
Lpp	-0.1499	0.4927	0.9995	-0.9298	0.0006	0.0145	-1.1214	0.0000	0.0018
Flot1	-0.0208	0.9444	0.9995	-1.0501	0.0051	0.0465	-1.1217	0.0016	0.0161
BC052040	0.1128	0.7314	0.9995	-1.2304	0.0038	0.0386	-1.1217	0.0038	0.0272
Morn1	0.0802	0.9105	0.9995	-3.8217	0.0022	0.0277	-1.1229	0.1463	0.2991
Npm1	0.0242	0.9079	0.9995	-1.2478	0.0000	0.0022	-1.1231	0.0000	0.0013
Nop53	-0.2839	0.4041	0.9995	-0.7564	0.0404	0.1591	-1.1247	0.0024	0.0208
Il18	-0.4770	0.2524	0.9995	-1.8982	0.0047	0.0439	-1.1253	0.0122	0.0598
Tmem40	-0.5692	0.1550	0.9995	-0.0010	0.9980	0.9988	-1.1255	0.0074	0.0429
Morc4	-0.3246	0.2071	0.9995	-0.7805	0.0069	0.0549	-1.1271	0.0002	0.0042
3110040N11Rik	-0.4807	0.1984	0.9995	0.1654	0.6651	0.8155	-1.1271	0.0064	0.0392
Vegfb	-0.1748	0.5331	0.9995	-1.1798	0.0009	0.0179	-1.1280	0.0006	0.0084
Tgm2	0.0562	0.8381	0.9995	-1.1003	0.0011	0.0195	-1.1283	0.0005	0.0073
Map3k1	-0.3996	0.2169	0.9995	-0.7054	0.0505	0.1818	-1.1283	0.0017	0.0169
Mtss1	-0.1519	0.4340	0.9995	-1.1919	0.0000	0.0022	-1.1291	0.0000	0.0008
Rarg	-0.2875	0.2433	0.9995	-1.0573	0.0007	0.0155	-1.1317	0.0001	0.0036
Impa2	0.0781	0.8120	0.9995	-1.6952	0.0004	0.0117	-1.1318	0.0034	0.0254
Tmem135	0.0248	0.9250	0.9995	-0.9981	0.0029	0.0331	-1.1320	0.0007	0.0091
Vegfa	0.1028	0.7038	0.9995	-1.7081	0.0000	0.0032	-1.1346	0.0007	0.0090
Tubb5	0.0189	0.9449	0.9995	-1.1542	0.0009	0.0182	-1.1348	0.0006	0.0084
Dst	-0.4055	0.1631	0.9995	-0.3439	0.2383	0.4619	-1.1383	0.0005	0.0076
Atg10	0.1359	0.8224	0.9995	-2.7565	0.0035	0.0372	-1.1384	0.0912	0.2217
Ank	-0.7027	0.0390	0.9995	-0.5378	0.1189	0.3042	-1.1386	0.0014	0.0144
B230118H07Rik	-0.2847	0.3854	0.9995	-0.8952	0.0221	0.1100	-1.1411	0.0023	0.0202
Fndc3a	0.1094	0.6561	0.9995	-1.5323	0.0000	0.0025	-1.1414	0.0002	0.0041
Celf2	0.0091	0.9607	0.9995	-1.2586	0.0000	0.0012	-1.1415	0.0000	0.0006
Usp9x	-0.1261	0.5366	0.9995	-0.8838	0.0006	0.0135	-1.1428	0.0000	0.0011

Farp1	-0.1957	0.4920	0.9995	-0.8637	0.0126	0.0794	-1.1462	0.0009	0.0111
Lrp1	-0.2022	0.5288	0.9995	-0.8180	0.0276	0.1260	-1.1463	0.0020	0.0182
Col5a1	-0.4455	0.0714	0.9995	-0.5930	0.0274	0.1256	-1.1469	0.0001	0.0027
Adgrl2	-0.0287	0.9114	0.9995	-0.7951	0.0094	0.0659	-1.1470	0.0004	0.0064
Itpr2	-0.5564	0.0458	0.9995	-0.7243	0.0195	0.1018	-1.1479	0.0003	0.0053
Afap1	-0.3551	0.2565	0.9995	-0.5752	0.0808	0.2412	-1.1481	0.0010	0.0118
Akt3	0.0408	0.8536	0.9995	-0.9980	0.0006	0.0137	-1.1482	0.0001	0.0024
Mast4	-0.1736	0.4748	0.9995	-0.7386	0.0090	0.0645	-1.1483	0.0001	0.0036
Ctps2	-0.1003	0.7143	0.9995	-0.6276	0.0451	0.1695	-1.1495	0.0007	0.0092
Rps20	-0.2207	0.4535	0.9995	-0.9093	0.0061	0.0512	-1.1513	0.0006	0.0084
Ndrg1	0.1770	0.6526	0.9995	-1.5238	0.0013	0.0211	-1.1520	0.0060	0.0374
Ugp2	-0.0690	0.8112	0.9995	-0.8370	0.0130	0.0809	-1.1541	0.0009	0.0110
Gm3940	-0.2389	0.3956	0.9995	-0.7117	0.0255	0.1204	-1.1547	0.0005	0.0079
Tead2	-0.1228	0.6402	0.9995	-1.2507	0.0005	0.0124	-1.1597	0.0003	0.0062
Col16a1	0.1212	0.6746	0.9995	-1.1722	0.0013	0.0213	-1.1598	0.0008	0.0104
Tbca	-0.1304	0.5743	0.9995	-0.7949	0.0032	0.0348	-1.1620	0.0001	0.0020
Mettl8	-0.3759	0.3060	0.9995	-0.7194	0.0941	0.2648	-1.1622	0.0052	0.0343
Cep350	-0.1491	0.5706	0.9995	-1.6589	0.0000	0.0030	-1.1632	0.0003	0.0056
Slc37a4	-0.1129	0.7608	0.9995	-1.4435	0.0055	0.0482	-1.1634	0.0072	0.0420
Trpv4	-0.1862	0.5190	0.9995	-1.2184	0.0016	0.0236	-1.1643	0.0008	0.0103
Dnpep	0.1850	0.4532	0.9995	-0.8125	0.0057	0.0495	-1.1644	0.0003	0.0053
Stk3	0.0615	0.7771	0.9995	-1.2978	0.0000	0.0034	-1.1646	0.0001	0.0020
Ttyh3	0.4331	0.3043	0.9995	-1.6967	0.0015	0.0227	-1.1647	0.0134	0.0639
Cacfd1	0.0691	0.7865	0.9995	-1.3690	0.0002	0.0085	-1.1652	0.0004	0.0066
Capn2	-0.2720	0.3382	0.9995	-0.8086	0.0115	0.0754	-1.1668	0.0004	0.0067
Plekhg5	-0.7216	0.0829	0.9995	-0.2848	0.5194	0.7163	-1.1676	0.0064	0.0392
Tmem205	0.0590	0.8530	0.9995	-0.7781	0.0339	0.1427	-1.1701	0.0024	0.0206
Gm11826	-0.5652	0.1928	0.9995	-0.6332	0.1508	0.3509	-1.1707	0.0084	0.0467
Hddc3	0.4721	0.2927	0.9995	-2.6220	0.0006	0.0135	-1.1710	0.0315	0.1091
Pcmtd2	-0.1182	0.6690	0.9995	-1.2616	0.0008	0.0165	-1.1712	0.0005	0.0081
Kmt2e	0.0577	0.8004	0.9995	-1.5064	0.0000	0.0013	-1.1721	0.0000	0.0018
Cep89	-0.2469	0.4287	0.9995	-0.7944	0.0304	0.1336	-1.1729	0.0013	0.0142
Nipbl	-0.0568	0.8051	0.9995	-1.1119	0.0002	0.0075	-1.1743	0.0000	0.0018
Baz2b	-0.2758	0.1834	0.9995	-0.9418	0.0003	0.0104	-1.1757	0.0000	0.0009
Hdac7	0.0793	0.8179	0.9995	-1.0399	0.0081	0.0605	-1.1758	0.0021	0.0189
Upp1	0.3595	0.3256	0.9995	-0.2542	0.4865	0.6913	-1.1768	0.0087	0.0477
Rxra	0.0134	0.9634	0.9995	-1.5282	0.0003	0.0106	-1.1784	0.0010	0.0116
Slc39a10	-0.2736	0.3136	0.9995	-0.6993	0.0265	0.1229	-1.1789	0.0003	0.0062
Afap1l2	-0.2569	0.4338	0.9995	-1.5840	0.0015	0.0231	-1.1793	0.0023	0.0202
Ilvbl	-0.2842	0.3603	0.9995	-0.7037	0.0476	0.1750	-1.1794	0.0012	0.0136
Ptma	-0.3140	0.3782	0.9995	-0.9530	0.0145	0.0861	-1.1807	0.0022	0.0195
Aig1	-0.3191	0.2152	0.9995	-0.7495	0.0135	0.0825	-1.1808	0.0002	0.0042
Fbxo6	-0.0900	0.7580	0.9995	-1.3218	0.0012	0.0202	-1.1819	0.0009	0.0114
Kif3a	-0.0099	0.9631	0.9995	-1.4110	0.0000	0.0017	-1.1827	0.0000	0.0012
Efna5	-0.0462	0.8339	0.9995	-0.9625	0.0006	0.0148	-1.1848	0.0000	0.0018
Rpl17-ps9	-0.1385	0.5114	0.9995	-1.2720	0.0000	0.0021	-1.1858	0.0000	0.0010
Adam10	-0.2668	0.3650	0.9995	-0.7379	0.0273	0.1254	-1.1860	0.0006	0.0085
Rbl1	-0.1997	0.4420	0.9995	-1.3340	0.0003	0.0106	-1.1875	0.0002	0.0050
Hook3	-0.1724	0.4227	0.9995	-0.8516	0.0011	0.0196	-1.1891	0.0000	0.0011
Map3k5	0.0438	0.8703	0.9995	-1.2880	0.0004	0.0117	-1.1906	0.0003	0.0062
Kcnn4	-0.3122	0.3853	0.9995	-0.5348	0.1711	0.3786	-1.1914	0.0031	0.0238

Arhgef39	-0.2648	0.5129	0.9995	-0.9593	0.0470	0.1739	-1.1918	0.0090	0.0486
Uqcrh	-0.1632	0.5882	0.9995	-0.5641	0.0768	0.2330	-1.1921	0.0006	0.0084
Exoc6b	0.3570	0.2390	0.9995	-1.4746	0.0005	0.0124	-1.1921	0.0015	0.0155
Dsp	-0.2801	0.2645	0.9995	-1.1647	0.0003	0.0103	-1.1958	0.0001	0.0027
Cdk19	-0.3586	0.3196	0.9995	-0.8295	0.0382	0.1532	-1.1967	0.0025	0.0214
Magi3	-0.1490	0.4767	0.9995	-1.2728	0.0000	0.0029	-1.1969	0.0000	0.0011
Ift81	-0.3101	0.3024	0.9995	-0.7300	0.0360	0.1478	-1.1973	0.0007	0.0098
Myh14	-0.7465	0.0409	0.9995	-1.2581	0.0057	0.0494	-1.2012	0.0017	0.0166
Trappc6a	-0.3396	0.2627	0.9995	-1.2569	0.0014	0.0219	-1.2020	0.0007	0.0090
Stag2	-0.1598	0.4425	0.9995	-0.7618	0.0021	0.0271	-1.2028	0.0000	0.0009
Atp2c2	0.1374	0.7050	0.9995	-1.0512	0.0156	0.0894	-1.2031	0.0069	0.0409
AI429214	-0.5091	0.1848	0.9995	-0.1256	0.7527	0.8710	-1.2034	0.0046	0.0313
Ptpn4	-0.4475	0.2032	0.9995	-0.8645	0.0405	0.1594	-1.2036	0.0028	0.0224
Ptms	-0.1644	0.6293	0.9995	-1.3305	0.0017	0.0243	-1.2051	0.0017	0.0168
Gm10335	-0.4524	0.1654	0.9995	-0.4869	0.1389	0.3344	-1.2056	0.0008	0.0102
Clybl	0.3356	0.4121	0.9995	-1.5197	0.0045	0.0430	-1.2059	0.0119	0.0587
Dhcr7	-0.3305	0.4230	0.9995	-0.9779	0.0471	0.1741	-1.2066	0.0088	0.0479
Slc4a11	-0.3689	0.2657	0.9995	-1.2166	0.0039	0.0397	-1.2069	0.0014	0.0144
R3hdm2	-0.1655	0.4618	0.9995	-1.1418	0.0002	0.0070	-1.2069	0.0000	0.0015
Bnip1	0.2412	0.5102	0.9995	-1.4316	0.0026	0.0313	-1.2070	0.0062	0.0381
Gm10132	-0.4531	0.1645	0.9995	-0.4844	0.1406	0.3368	-1.2073	0.0008	0.0100
Itgb3bp	-0.5770	0.0813	0.9995	-0.6373	0.0945	0.2653	-1.2096	0.0010	0.0120
Itpr3	-0.4117	0.0785	0.9995	-0.6011	0.0176	0.0959	-1.2098	0.0000	0.0014
Narf	0.1844	0.6008	0.9995	-1.2740	0.0051	0.0462	-1.2105	0.0042	0.0296
Plekhn1	-0.2983	0.4224	0.9995	-0.8481	0.0572	0.1956	-1.2111	0.0041	0.0288
Eya1	-0.1520	0.6875	0.9995	-1.3232	0.0087	0.0627	-1.2117	0.0058	0.0365
R3hcc1	0.0319	0.9147	0.9995	-1.5736	0.0003	0.0096	-1.2136	0.0008	0.0104
Lancl2	0.4473	0.1748	0.9995	-1.4449	0.0010	0.0185	-1.2140	0.0033	0.0251
Dhrs9	-0.6603	0.0918	0.9995	-0.9875	0.0451	0.1695	-1.2154	0.0036	0.0265
Btrc	-0.5452	0.1778	0.9995	-0.1347	0.7495	0.8691	-1.2185	0.0056	0.0357
Mllt10	-0.2236	0.3266	0.9995	-0.7054	0.0070	0.0551	-1.2220	0.0000	0.0014
Mob3b	-0.0111	0.9760	0.9995	-1.4599	0.0029	0.0333	-1.2221	0.0055	0.0351
Atp8b1	-0.0729	0.8427	0.9995	-0.9335	0.0390	0.1555	-1.2242	0.0064	0.0390
Myh9	-0.4998	0.0573	0.9995	-0.7074	0.0112	0.0738	-1.2246	0.0001	0.0022
Appl2	-0.0844	0.7959	0.9995	-1.0396	0.0099	0.0685	-1.2248	0.0020	0.0185
Ogt	-0.4821	0.1895	0.9995	-0.4421	0.2554	0.4798	-1.2270	0.0025	0.0213
Kat6b	-0.5466	0.1471	0.9995	-0.7639	0.0817	0.2428	-1.2288	0.0029	0.0232
Smim19	-0.3637	0.2026	0.9995	-0.8650	0.0104	0.0705	-1.2296	0.0003	0.0057
Svil	-0.2194	0.3688	0.9995	-1.2967	0.0002	0.0072	-1.2334	0.0001	0.0025
Gm15466	-0.1198	0.7967	0.9995	-3.3026	0.0005	0.0123	-1.2342	0.0236	0.0914
Tmem71	-0.2412	0.4260	0.9995	-1.4990	0.0004	0.0111	-1.2349	0.0006	0.0085
Bmpr2	-0.2347	0.5493	0.9995	-0.7639	0.0915	0.2604	-1.2362	0.0061	0.0377
Serinc2	-0.6265	0.1433	0.9995	-0.4628	0.2941	0.5216	-1.2367	0.0057	0.0363
Arhgef25	-0.0135	0.9643	0.9995	-1.0946	0.0039	0.0395	-1.2371	0.0009	0.0110
Dhx57	-0.1876	0.5578	0.9995	-0.5710	0.1068	0.2850	-1.2385	0.0011	0.0130
Camsap3	-0.7557	0.0308	0.9995	-1.1989	0.0106	0.0711	-1.2409	0.0010	0.0118
Hoxa5	0.1685	0.5746	0.9995	-1.6260	0.0001	0.0040	-1.2409	0.0004	0.0067
Meis1	-0.3903	0.3018	0.9995	-0.4761	0.2599	0.4847	-1.2450	0.0044	0.0302
Nme4	0.2434	0.5463	0.9995	-1.4872	0.0044	0.0426	-1.2458	0.0085	0.0468
Prkca	-0.1517	0.5526	0.9995	-0.8379	0.0065	0.0527	-1.2462	0.0001	0.0033
Lrba	-0.5448	0.0788	0.9995	-1.0846	0.0064	0.0523	-1.2473	0.0005	0.0073

Itpk1	0.1810	0.6449	0.9995	-1.5560	0.0037	0.0383	-1.2477	0.0073	0.0426
Lst1	0.3695	0.2989	0.9995	-1.2753	0.0042	0.0414	-1.2497	0.0040	0.0283
Dock7	-0.1374	0.5766	0.9995	-1.5928	0.0000	0.0029	-1.2501	0.0001	0.0027
Tcf12	0.0628	0.7937	0.9995	-1.0099	0.0008	0.0171	-1.2506	0.0001	0.0021
Zmiz1	-0.2319	0.4135	0.9995	-1.2674	0.0008	0.0167	-1.2511	0.0003	0.0059
Tbc1d32	-0.5656	0.0867	0.9995	-0.9454	0.0189	0.0999	-1.2526	0.0008	0.0108
Sema3f	0.0318	0.9230	0.9995	-1.2031	0.0053	0.0471	-1.2530	0.0018	0.0173
Poc1b	0.0407	0.8629	0.9995	-1.3024	0.0001	0.0063	-1.2530	0.0001	0.0023
Pkp3	-0.1785	0.6326	0.9995	-1.5197	0.0014	0.0218	-1.2546	0.0025	0.0214
Col4a5	-0.6142	0.1064	0.9995	-0.8317	0.0594	0.1997	-1.2548	0.0027	0.0223
Echdc3	0.4736	0.3807	0.9995	-2.4204	0.0018	0.0251	-1.2553	0.0449	0.1384
Igf1r	0.1329	0.6339	0.9995	-1.3750	0.0002	0.0076	-1.2555	0.0002	0.0046
Atp11c	0.1001	0.7318	0.9995	-1.2254	0.0015	0.0228	-1.2556	0.0006	0.0085
Rps15	0.1043	0.6170	0.9995	-1.1426	0.0000	0.0034	-1.2557	0.0000	0.0006
Gm12504	-0.5007	0.2532	0.9995	-0.7247	0.1217	0.3088	-1.2561	0.0067	0.0401
Atp9a	-0.4084	0.3044	0.9995	-1.0096	0.0236	0.1151	-1.2566	0.0034	0.0257
Arrdc3	-0.1822	0.6479	0.9995	-0.5979	0.1738	0.3828	-1.2584	0.0082	0.0460
Pitpnc1	0.0327	0.8861	0.9995	-0.7888	0.0040	0.0401	-1.2585	0.0000	0.0017
Skap2	0.0551	0.8494	0.9995	-1.4405	0.0002	0.0075	-1.2619	0.0003	0.0056
Gm8692	0.1007	0.7427	0.9995	-1.1600	0.0029	0.0327	-1.2628	0.0008	0.0107
Etl4	-0.3775	0.1813	0.9995	-0.7179	0.0236	0.1151	-1.2636	0.0002	0.0043
Nf1	-0.5465	0.0521	0.9995	-0.7645	0.0188	0.0997	-1.2666	0.0001	0.0036
Prss22	0.4464	0.2244	0.9995	-1.4481	0.0017	0.0243	-1.2681	0.0049	0.0326
Xylt2	0.5295	0.0559	0.9995	-2.3176	0.0000	0.0010	-1.2700	0.0004	0.0071
Trappc12	-0.1894	0.5204	0.9995	-0.6162	0.0652	0.2096	-1.2701	0.0005	0.0081
Pink1	-0.2057	0.5616	0.9995	-1.0955	0.0138	0.0834	-1.2712	0.0026	0.0216
Rnf13	-0.2545	0.5329	0.9995	-1.4292	0.0075	0.0575	-1.2718	0.0075	0.0434
Pcbp3	-0.3457	0.3664	0.9995	-0.0494	0.9013	0.9542	-1.2721	0.0050	0.0332
Jak3	-0.0258	0.9450	0.9995	-1.3257	0.0094	0.0661	-1.2728	0.0059	0.0369
Gstm5	0.2078	0.5714	0.9995	-1.6771	0.0015	0.0226	-1.2738	0.0057	0.0362
Slc10a7	-0.5745	0.1118	0.9995	-1.1602	0.0105	0.0709	-1.2761	0.0016	0.0161
Sp3os	-0.1809	0.5827	0.9995	-1.3600	0.0035	0.0372	-1.2774	0.0019	0.0178
Myo18a	-0.3515	0.2500	0.9995	-0.9401	0.0070	0.0552	-1.2804	0.0003	0.0058
Idh2	0.1139	0.6038	0.9995	-0.9532	0.0006	0.0137	-1.2807	0.0000	0.0010
Tmem94	-0.3513	0.3769	0.9995	-0.4779	0.2785	0.5051	-1.2822	0.0050	0.0331
Rhox4f	0.0532	0.8787	0.9995	-1.1906	0.0093	0.0658	-1.2834	0.0029	0.0231
Vamp4	-0.1531	0.6505	0.9995	-1.2315	0.0071	0.0558	-1.2840	0.0023	0.0200
Zer1	-0.2709	0.4576	0.9995	-1.5324	0.0042	0.0409	-1.2850	0.0029	0.0229
Ap3b1	-0.1986	0.2937	0.9995	-0.9856	0.0001	0.0054	-1.2851	0.0000	0.0003
Tes	-0.4534	0.2057	0.9995	-0.4487	0.2178	0.4381	-1.2858	0.0011	0.0128
Tep1	-0.1780	0.5206	0.9995	-0.8245	0.0140	0.0841	-1.2872	0.0003	0.0053
Rpl41	-0.2721	0.3149	0.9995	-0.7707	0.0097	0.0671	-1.2876	0.0001	0.0025
Ccdc167	-0.0177	0.9647	0.9995	-0.7872	0.1024	0.2784	-1.2885	0.0080	0.0455
Eid1	0.2161	0.3051	0.9995	-1.3362	0.0000	0.0018	-1.2902	0.0000	0.0006
Stard10	-0.0431	0.8564	0.9995	-1.0653	0.0006	0.0141	-1.2911	0.0000	0.0018
Srgap3	-0.3992	0.1809	0.9995	-0.8744	0.0128	0.0801	-1.2914	0.0003	0.0058
Gprasp1	0.1442	0.7108	0.9995	-1.0372	0.0335	0.1419	-1.2927	0.0067	0.0401
Huwe1	-0.3131	0.2121	0.9995	-0.9668	0.0013	0.0210	-1.2943	0.0000	0.0016
Rlf	-0.3480	0.1942	0.9995	-0.8730	0.0057	0.0495	-1.2944	0.0001	0.0027
Asb2	2.2856	0.0012	0.9711	-2.2109	0.0028	0.0322	-1.2965	0.1276	0.2743
Man2a1	-0.2821	0.2856	0.9995	-1.0338	0.0020	0.0263	-1.3003	0.0001	0.0029

Dph6	0.2047	0.2983	0.9995	-1.1969	0.0000	0.0026	-1.3011	0.0000	0.0005
Ezh1	-0.2719	0.4626	0.9995	-0.9179	0.0402	0.1587	-1.3015	0.0028	0.0225
Tmprss11e	0.3375	0.1538	0.9995	-1.4641	0.0000	0.0018	-1.3041	0.0000	0.0011
Pknox1	-0.5393	0.0551	0.9995	-0.5062	0.0918	0.2609	-1.3051	0.0001	0.0029
Ltbp2	0.4284	0.2544	0.9995	-0.9612	0.0234	0.1146	-1.3096	0.0037	0.0271
Hoxaas3	-0.0162	0.9750	0.9995	-2.4362	0.0045	0.0430	-1.3097	0.0305	0.1068
Vti1a	-0.1955	0.5647	0.9995	-1.0669	0.0165	0.0927	-1.3153	0.0017	0.0169
Aldh3b1	0.1104	0.7028	0.9995	-1.3560	0.0008	0.0161	-1.3163	0.0006	0.0084
Zfp292	-0.0688	0.7870	0.9995	-1.4372	0.0000	0.0036	-1.3201	0.0000	0.0018
Anxa2	-0.3699	0.2553	0.9995	-0.6472	0.0569	0.1950	-1.3223	0.0004	0.0064
Strbp	-0.3592	0.2558	0.9995	-0.9304	0.0085	0.0620	-1.3225	0.0003	0.0057
Camk2n1	-0.1790	0.6232	0.9995	-1.5246	0.0031	0.0340	-1.3255	0.0025	0.0209
Mipol1	-0.0022	0.9937	0.9995	-1.4134	0.0007	0.0151	-1.3279	0.0004	0.0068
Tbl1x	-0.2273	0.3179	0.9995	-0.9037	0.0017	0.0240	-1.3279	0.0000	0.0011
Borcs7	-0.0011	0.9977	0.9995	-1.9415	0.0007	0.0160	-1.3286	0.0040	0.0284
Faf1	-0.1984	0.5042	0.9995	-1.4279	0.0005	0.0132	-1.3297	0.0003	0.0060
Arhgap18	0.1252	0.6963	0.9995	-1.3395	0.0011	0.0196	-1.3298	0.0006	0.0090
Col18a1	0.1127	0.6324	0.9995	-1.4010	0.0000	0.0030	-1.3318	0.0000	0.0012
Zc3h6	0.1055	0.7695	0.9995	-2.0806	0.0002	0.0085	-1.3325	0.0025	0.0209
Akr7a5	-0.1920	0.5802	0.9995	-0.7751	0.0499	0.1805	-1.3335	0.0013	0.0138
Zfp260	-0.2589	0.2612	0.9995	-1.2163	0.0001	0.0069	-1.3342	0.0000	0.0010
Hcfc1r1	0.0248	0.9129	0.9995	-1.7934	0.0000	0.0005	-1.3360	0.0000	0.0008
Ogfrl1	-0.0467	0.8456	0.9995	-1.5620	0.0000	0.0034	-1.3367	0.0000	0.0019
Nsmce1	-0.0520	0.8420	0.9995	-0.3882	0.1626	0.3684	-1.3368	0.0001	0.0028
Zfp236	-0.2588	0.4993	0.9995	-0.9315	0.0517	0.1843	-1.3380	0.0031	0.0239
Slc40a1	-0.7778	0.1059	0.9995	-0.5108	0.3352	0.5606	-1.3381	0.0081	0.0459
Sfmbt2	-0.1493	0.5655	0.9995	-0.9328	0.0045	0.0432	-1.3386	0.0001	0.0032
Smyd3	0.1108	0.6778	0.9995	-1.3115	0.0006	0.0135	-1.3398	0.0002	0.0049
Zfp358	0.0532	0.8952	0.9995	-1.2880	0.0119	0.0768	-1.3423	0.0057	0.0360
Polr3gl	-0.2119	0.6155	0.9995	-1.6153	0.0069	0.0549	-1.3430	0.0073	0.0426
Cotl1	-0.5002	0.1154	0.9995	-0.5644	0.0965	0.2681	-1.3446	0.0003	0.0057
Slc2a9	0.1347	0.6937	0.9995	-2.3206	0.0001	0.0052	-1.3447	0.0019	0.0176
Ascc1	-0.4735	0.1644	0.9995	-0.7461	0.0577	0.1964	-1.3468	0.0008	0.0102
Palld	-0.8529	0.0351	0.9995	-0.1709	0.6793	0.8239	-1.3472	0.0015	0.0155
Lama5	-0.0495	0.8484	0.9995	-1.3213	0.0002	0.0070	-1.3494	0.0001	0.0020
Btbd2	0.0558	0.9038	0.9995	-2.2839	0.0015	0.0230	-1.3502	0.0135	0.0639
Ttc26	0.5112	0.2813	0.9995	-1.8301	0.0046	0.0433	-1.3504	0.0219	0.0871
Kxd1	0.1869	0.5980	0.9995	-1.2971	0.0041	0.0406	-1.3516	0.0021	0.0191
Ahnak2	0.0249	0.9131	0.9995	-1.5111	0.0000	0.0017	-1.3563	0.0000	0.0009
Tcf7l1	-0.0339	0.8954	0.9995	-1.7558	0.0000	0.0029	-1.3565	0.0001	0.0028
Baiap2	-0.2792	0.2722	0.9995	-0.9713	0.0018	0.0254	-1.3566	0.0000	0.0016
Mturn	0.0708	0.9130	0.9995	-3.0390	0.0049	0.0454	-1.3574	0.0615	0.1698
Glmn	-0.2252	0.5624	0.9995	-0.3799	0.3570	0.5832	-1.3580	0.0036	0.0267
Emp2	-0.1510	0.5353	0.9995	-1.2977	0.0001	0.0048	-1.3585	0.0000	0.0011
Lipe	-0.2466	0.4637	0.9995	-1.1554	0.0081	0.0604	-1.3597	0.0009	0.0113
Ankzf1	-0.1006	0.7935	0.9995	-1.0868	0.0282	0.1276	-1.3608	0.0036	0.0266
Suclg2	-0.0540	0.8290	0.9995	-1.2233	0.0004	0.0114	-1.3618	0.0001	0.0019
Igfbp4	0.2926	0.2039	0.9995	-1.2605	0.0000	0.0031	-1.3623	0.0000	0.0006
Sgsm2	-0.1102	0.6988	0.9995	-1.3963	0.0005	0.0135	-1.3625	0.0002	0.0051
Tenm4	-0.2328	0.4170	0.9995	-1.5046	0.0003	0.0107	-1.3632	0.0002	0.0041
Eml5	-0.5077	0.2302	0.9995	-0.4128	0.3718	0.5962	-1.3654	0.0043	0.0301

4932438A13Rik	-0.4586	0.0996	0.9995	-1.2053	0.0009	0.0172	-1.3659	0.0001	0.0023
Xrcc4	-0.3427	0.3734	0.9995	-1.4883	0.0045	0.0430	-1.3666	0.0027	0.0220
Gm10052	0.1042	0.6928	0.9995	-1.1498	0.0009	0.0174	-1.3670	0.0001	0.0032
Zscan21	-0.2526	0.4302	0.9995	-0.9962	0.0166	0.0927	-1.3700	0.0007	0.0098
Nedd4	-0.2296	0.3901	0.9995	-1.0209	0.0013	0.0215	-1.3715	0.0000	0.0017
Pgm2	-0.6346	0.1559	0.9995	0.1246	0.7838	0.8881	-1.3741	0.0049	0.0327
Erbb2	-0.5908	0.0939	0.9995	-0.8066	0.0540	0.1896	-1.3785	0.0008	0.0100
Stoml1	0.4129	0.2268	0.9995	-1.0882	0.0081	0.0605	-1.3785	0.0020	0.0183
Ppp1r9a	-0.1637	0.4922	0.9995	-1.3389	0.0001	0.0062	-1.3803	0.0000	0.0014
Stard4	-0.3486	0.4408	0.9995	-0.8883	0.0827	0.2450	-1.3819	0.0065	0.0393
Zbtb12	0.0864	0.8279	0.9995	-1.8902	0.0010	0.0191	-1.3842	0.0032	0.0242
Magi1	0.1849	0.5308	0.9995	-1.6961	0.0001	0.0059	-1.3843	0.0003	0.0059
Fbxl17	0.2005	0.5549	0.9995	-1.3334	0.0028	0.0324	-1.3859	0.0017	0.0168
Acads	-0.2765	0.5115	0.9995	-0.9691	0.0449	0.1692	-1.3869	0.0037	0.0269
Gm4997	-0.6899	0.1694	0.9995	-0.5620	0.2654	0.4907	-1.3888	0.0071	0.0417
Ctxn1	-0.5056	0.2211	0.9995	-0.5511	0.2238	0.4452	-1.3914	0.0029	0.0228
Top2b	0.2417	0.2250	0.9995	-1.4776	0.0000	0.0007	-1.3929	0.0000	0.0002
Rbpj	0.1858	0.3513	0.9995	-1.4150	0.0000	0.0008	-1.3935	0.0000	0.0002
Smim22	-0.1804	0.5238	0.9995	-1.6234	0.0002	0.0085	-1.3947	0.0002	0.0042
Sec24d	0.0159	0.9608	0.9995	-0.7784	0.0369	0.1500	-1.3954	0.0010	0.0120
Kctd2	-0.2380	0.5528	0.9995	-1.7514	0.0021	0.0271	-1.3959	0.0029	0.0231
Pafah1b3	0.2328	0.3578	0.9995	-1.2801	0.0001	0.0065	-1.3985	0.0000	0.0014
Itga3	-0.1904	0.5154	0.9995	-0.7106	0.0263	0.1226	-1.3998	0.0001	0.0028
AU040320	-0.3231	0.2290	0.9995	-0.6937	0.0246	0.1183	-1.4014	0.0001	0.0022
Mvb12b	0.0785	0.7893	0.9995	-1.5096	0.0003	0.0109	-1.4025	0.0003	0.0059
Akr1e1	0.0126	0.9719	0.9995	-1.6709	0.0013	0.0211	-1.4035	0.0021	0.0191
Gm5643	0.3546	0.2090	0.9995	-1.5570	0.0000	0.0030	-1.4059	0.0000	0.0019
Ldha	-0.1225	0.4807	0.9995	-1.2340	0.0000	0.0008	-1.4081	0.0000	0.0001
Lrrc61	-0.0388	0.9365	0.9995	-3.5467	0.0006	0.0135	-1.4084	0.0179	0.0761
Hnrnpa1	0.3213	0.2075	0.9995	-1.5513	0.0000	0.0018	-1.4113	0.0000	0.0010
Pgam1	-0.2443	0.3838	0.9995	-1.0649	0.0013	0.0213	-1.4123	0.0000	0.0018
Sema4d	-0.2267	0.5779	0.9995	-1.2989	0.0160	0.0906	-1.4133	0.0047	0.0320
Nr2c2	-0.2921	0.3620	0.9995	-1.2681	0.0017	0.0247	-1.4133	0.0003	0.0054
Slc25a13	-0.2937	0.2402	0.9995	-1.0084	0.0013	0.0213	-1.4136	0.0000	0.0011
Gm5879	-0.2663	0.3716	0.9995	-1.0915	0.0018	0.0255	-1.4149	0.0001	0.0027
Rara	-0.5426	0.1379	0.9995	-0.4173	0.2792	0.5061	-1.4155	0.0008	0.0102
Chm	-0.2302	0.3567	0.9995	-0.7618	0.0101	0.0693	-1.4172	0.0000	0.0015
Il1r1	-1.1654	0.0124	0.9995	-0.1859	0.7020	0.8397	-1.4181	0.0019	0.0176
Oxct1	-0.6618	0.0884	0.9995	-0.5878	0.1390	0.3344	-1.4197	0.0008	0.0107
Adck2	-0.0008	0.9987	0.9996	-2.5325	0.0018	0.0255	-1.4208	0.0146	0.0673
Gamt	-0.3173	0.3962	0.9995	-0.6539	0.1306	0.3213	-1.4233	0.0017	0.0168
Hmga2	0.1355	0.6197	0.9995	-1.0063	0.0019	0.0257	-1.4245	0.0000	0.0017
Cep131	0.2479	0.4408	0.9995	-1.5994	0.0006	0.0139	-1.4245	0.0008	0.0108
Cpt2	0.0041	0.9915	0.9995	-0.9402	0.0375	0.1515	-1.4253	0.0028	0.0226
Ctnna1	-0.2167	0.4386	0.9995	-1.0763	0.0016	0.0233	-1.4255	0.0001	0.0020
Nsmf	-0.2116	0.5339	0.9995	-1.3516	0.0036	0.0378	-1.4261	0.0010	0.0120
Ago1	-0.2171	0.4510	0.9995	-1.3850	0.0007	0.0152	-1.4270	0.0001	0.0037
Ghr	-0.1958	0.5161	0.9995	-1.3152	0.0014	0.0219	-1.4270	0.0003	0.0058
Psrc1	0.3538	0.5796	0.9995	-2.7869	0.0053	0.0472	-1.4301	0.0674	0.1812
Cux1	-0.2917	0.2837	0.9995	-0.9231	0.0048	0.0452	-1.4331	0.0000	0.0019
Pxylp1	-0.3972	0.2220	0.9995	-1.0898	0.0068	0.0544	-1.4393	0.0003	0.0058

Dcaf6	-0.2078	0.5744	0.9995	-0.8610	0.0442	0.1675	-1.4395	0.0012	0.0136
Fam3a	-0.1875	0.6653	0.9995	-0.9201	0.0925	0.2623	-1.4403	0.0077	0.0441
4930412O13Rik	-0.8492	0.0990	0.9995	-0.7044	0.2461	0.4708	-1.4483	0.0075	0.0434
Rhox4c	-0.2745	0.4995	0.9995	-1.0091	0.0502	0.1813	-1.4621	0.0033	0.0250
Tceanc	0.2981	0.4851	0.9995	-1.9831	0.0024	0.0295	-1.4663	0.0086	0.0475
Rhox4a2	0.0778	0.7674	0.9995	-1.4531	0.0001	0.0063	-1.4666	0.0000	0.0018
Plod2	-0.0749	0.8099	0.9995	-1.5385	0.0003	0.0109	-1.4668	0.0002	0.0044
Rhox4a	0.0723	0.7857	0.9995	-1.4242	0.0002	0.0072	-1.4672	0.0001	0.0020
Dock1	-0.7565	0.0796	0.9995	-0.8060	0.1043	0.2807	-1.4684	0.0020	0.0186
Glt8d1	-0.0398	0.9019	0.9995	-1.1443	0.0055	0.0486	-1.4708	0.0006	0.0083
Hint3	0.0685	0.8574	0.9995	-2.0408	0.0005	0.0130	-1.4730	0.0026	0.0216
Pmvk	-0.4336	0.3678	0.9995	-1.5580	0.0183	0.0980	-1.4731	0.0072	0.0422
Rhox4e	0.0007	0.9983	0.9995	-1.3352	0.0019	0.0256	-1.4747	0.0004	0.0066
Mettl27	-0.2817	0.3724	0.9995	-1.9428	0.0001	0.0064	-1.4753	0.0002	0.0048
Acaca	-0.0962	0.6922	0.9995	-1.0873	0.0007	0.0151	-1.4769	0.0000	0.0010
Stk39	-0.1059	0.6290	0.9995	-1.2274	0.0001	0.0047	-1.4796	0.0000	0.0004
Pkm	-0.0572	0.8258	0.9995	-1.3775	0.0001	0.0043	-1.4829	0.0000	0.0010
Il1b	0.7926	0.0598	0.9995	-2.4905	0.0002	0.0082	-1.4832	0.0092	0.0495
Syt8	-0.5609	0.1492	0.9995	-1.0144	0.0302	0.1330	-1.4865	0.0010	0.0116
Gm12816	-0.2734	0.3498	0.9995	-1.1763	0.0009	0.0172	-1.4888	0.0000	0.0018
Arhgap6	-0.0705	0.7707	0.9995	-1.4062	0.0001	0.0061	-1.4919	0.0000	0.0012
Sbk1	0.2226	0.6145	0.9995	-2.7144	0.0005	0.0134	-1.4925	0.0092	0.0495
Pex11g	-0.6357	0.1738	0.9995	-1.8183	0.0151	0.0879	-1.4937	0.0043	0.0299
Epcam	-0.3475	0.1532	0.9995	-1.3626	0.0000	0.0029	-1.4949	0.0000	0.0005
Cep112	-0.1652	0.6131	0.9995	-1.3536	0.0032	0.0349	-1.4960	0.0005	0.0079
Kdm5b	-0.0087	0.9755	0.9995	-1.4252	0.0003	0.0094	-1.4961	0.0001	0.0023
Rhox4b	0.0344	0.9163	0.9995	-1.3416	0.0020	0.0260	-1.4965	0.0004	0.0068
Fam171a2	-0.0097	0.9753	0.9995	-2.4113	0.0000	0.0025	-1.4971	0.0003	0.0056
Diaph2	-0.0269	0.9154	0.9995	-1.3041	0.0003	0.0103	-1.4974	0.0000	0.0014
Iqce	-0.7122	0.1489	0.9995	-0.6380	0.2675	0.4931	-1.4982	0.0059	0.0370
Lyl1	-0.2549	0.4165	0.9995	-1.1437	0.0057	0.0495	-1.4983	0.0002	0.0051
Snord83b	0.8266	0.0121	0.9995	-1.4498	0.0003	0.0106	-1.4986	0.0006	0.0085
Rabgap1l	0.0198	0.9332	0.9995	-1.5955	0.0000	0.0029	-1.4996	0.0000	0.0010
Rhox4g	0.0013	0.9972	0.9995	-1.2135	0.0132	0.0813	-1.5001	0.0020	0.0182
Efna4	-0.0917	0.7466	0.9995	-2.0067	0.0001	0.0055	-1.5057	0.0001	0.0036
Ndufaf3	0.0961	0.7606	0.9995	-1.3840	0.0018	0.0252	-1.5062	0.0005	0.0081
Higd1a	-0.4230	0.1672	0.9995	-1.1727	0.0013	0.0217	-1.5070	0.0001	0.0020
Eya2	0.0925	0.7377	0.9995	-1.3092	0.0005	0.0130	-1.5092	0.0001	0.0022
Sipa1l1	-0.1466	0.6013	0.9995	-1.2269	0.0006	0.0145	-1.5123	0.0000	0.0016
Pcdhb22	0.5701	0.4252	0.9995	-3.5186	0.0026	0.0313	-1.5172	0.0757	0.1958
Fam162a	0.1932	0.5622	0.9995	-1.8638	0.0000	0.0032	-1.5241	0.0001	0.0037
P4ha1	-0.1073	0.6867	0.9995	-1.5104	0.0001	0.0041	-1.5256	0.0000	0.0011
Gm10362	-0.3563	0.2319	0.9995	-1.2151	0.0008	0.0170	-1.5272	0.0000	0.0017
Maml3	0.1499	0.6537	0.9995	-1.3966	0.0029	0.0330	-1.5304	0.0008	0.0105
Fmnl1	-0.0867	0.7644	0.9995	-1.2265	0.0014	0.0217	-1.5369	0.0001	0.0025
Lgr6	0.4184	0.2902	0.9995	-1.8981	0.0009	0.0179	-1.5372	0.0038	0.0277
Fkbp1b	0.1225	0.7764	0.9995	-2.1664	0.0016	0.0236	-1.5390	0.0064	0.0393
Slc29a3	-0.4769	0.2850	0.9995	-0.7110	0.1587	0.3631	-1.5539	0.0027	0.0220
Ddit4	0.0307	0.9427	0.9995	-1.6670	0.0013	0.0213	-1.5552	0.0013	0.0137
Ypel1	-0.2874	0.5530	0.9995	-1.3466	0.0449	0.1692	-1.5558	0.0084	0.0468
Lnpep	-0.6899	0.0627	0.9995	-0.6713	0.1050	0.2818	-1.5566	0.0004	0.0063

Coasy	-0.0797	0.7781	0.9995	-1.9531	0.0000	0.0024	-1.5573	0.0000	0.0019
Ccdc136	-0.2984	0.3711	0.9995	-1.3170	0.0031	0.0342	-1.5579	0.0003	0.0058
Peak1	-0.4732	0.1909	0.9995	-0.9505	0.0215	0.1081	-1.5589	0.0003	0.0057
Trappc9	-0.2724	0.3798	0.9995	-1.2612	0.0026	0.0313	-1.5600	0.0001	0.0036
Ccdc50	-0.0801	0.7039	0.9995	-1.1905	0.0000	0.0034	-1.5611	0.0000	0.0002
P4ha2	0.1929	0.6066	0.9995	-1.2824	0.0055	0.0486	-1.5624	0.0013	0.0139
Megf8	-0.3602	0.2004	0.9995	-1.1808	0.0019	0.0257	-1.5671	0.0000	0.0018
Pgap1	-0.0436	0.8771	0.9995	-0.9328	0.0060	0.0506	-1.5719	0.0000	0.0018
Tpk1	0.0725	0.8531	0.9995	-1.6887	0.0043	0.0414	-1.5735	0.0028	0.0224
Pla2g7	-0.2834	0.6136	0.9995	-3.5004	0.0021	0.0271	-1.5747	0.0185	0.0775
Ccdc90b	0.2916	0.5208	0.9995	-1.7537	0.0076	0.0579	-1.5787	0.0079	0.0452
Ypel3	-0.3053	0.2280	0.9995	-1.3621	0.0002	0.0072	-1.5805	0.0000	0.0007
9-Mar	0.8964	0.1130	0.9995	-3.4067	0.0006	0.0135	-1.5828	0.0359	0.1187
Rhox4d	-0.2181	0.6142	0.9995	-1.2668	0.0235	0.1146	-1.5884	0.0029	0.0230
Fam114a1	-0.4392	0.2166	0.9995	-1.9334	0.0004	0.0121	-1.5909	0.0003	0.0059
Hebp2	-0.4786	0.1187	0.9995	-0.8838	0.0149	0.0873	-1.5954	0.0001	0.0020
Ubxn6	0.1028	0.7016	0.9995	-1.2883	0.0004	0.0118	-1.5968	0.0000	0.0014
Rps6ka6	0.3078	0.3158	0.9995	-1.6771	0.0002	0.0085	-1.5980	0.0002	0.0049
Vps13b	-0.1443	0.5911	0.9995	-1.5255	0.0001	0.0063	-1.5985	0.0000	0.0012
Dhrs3	-0.5030	0.2187	0.9995	-1.7655	0.0044	0.0421	-1.6014	0.0017	0.0166
Zfp704	0.0688	0.7826	0.9995	-1.6927	0.0000	0.0020	-1.6023	0.0000	0.0007
Scd1	-0.1719	0.7180	0.9995	-1.0634	0.0508	0.1823	-1.6046	0.0039	0.0278
Mt1	0.0344	0.9509	0.9995	-2.1767	0.0014	0.0223	-1.6064	0.0071	0.0417
Cradd	0.0121	0.9761	0.9995	-3.5393	0.0001	0.0051	-1.6076	0.0031	0.0238
Dennd4b	-0.7310	0.1421	0.9995	-0.3416	0.5393	0.7324	-1.6079	0.0053	0.0345
Kiz	-0.1297	0.6078	0.9995	-1.8137	0.0000	0.0020	-1.6085	0.0000	0.0008
Hist1h2ak	0.9716	0.1547	0.9995	-4.5893	0.0006	0.0145	-1.6101	0.0657	0.1777
Col8a1	0.5149	0.0272	0.9995	-1.7881	0.0000	0.0003	-1.6102	0.0000	0.0001
Atad2b	-0.1506	0.5699	0.9995	-1.6930	0.0000	0.0031	-1.6114	0.0000	0.0010
Rcor2	0.2786	0.5634	0.9995	-1.3289	0.0226	0.1118	-1.6129	0.0070	0.0414
Pcolce	-1.3128	0.0026	0.9995	-0.7302	0.0926	0.2623	-1.6145	0.0002	0.0051
Tmem106c	-0.7024	0.1124	0.9995	-1.0958	0.0563	0.1942	-1.6150	0.0021	0.0191
Ift88	0.0202	0.9545	0.9995	-1.5915	0.0017	0.0244	-1.6172	0.0007	0.0096
Pkp2	-0.1627	0.6460	0.9995	-1.7459	0.0007	0.0155	-1.6219	0.0004	0.0063
Map3k4	-0.1956	0.4335	0.9995	-1.4051	0.0001	0.0051	-1.6225	0.0000	0.0006
Mxi1	-0.0838	0.7714	0.9995	-1.6841	0.0001	0.0039	-1.6226	0.0000	0.0014
Ten1	-0.2016	0.6238	0.9995	-1.2681	0.0186	0.0993	-1.6249	0.0015	0.0158
Ccbe1	0.0347	0.8816	0.9995	-1.0642	0.0004	0.0122	-1.6251	0.0000	0.0004
Fah	0.1417	0.6783	0.9995	-1.4398	0.0016	0.0237	-1.6255	0.0004	0.0065
Stau2	-0.2735	0.2815	0.9995	-0.6476	0.0274	0.1256	-1.6267	0.0000	0.0009
2310002F09Rik	0.4456	0.4298	0.9995	-2.7572	0.0021	0.0268	-1.6268	0.0232	0.0905
Rsrc1	0.0101	0.9695	0.9995	-1.8125	0.0000	0.0017	-1.6282	0.0000	0.0008
Pgk1	0.2298	0.4097	0.9995	-1.7594	0.0000	0.0016	-1.6301	0.0000	0.0007
Clic1	-0.4515	0.3409	0.9995	-1.1070	0.0334	0.1416	-1.6302	0.0019	0.0176
Pacs1	-0.0313	0.8959	0.9995	-1.2219	0.0002	0.0092	-1.6335	0.0000	0.0006
Hes6	-0.4160	0.1628	0.9995	-1.4318	0.0007	0.0159	-1.6367	0.0000	0.0019
Copg2	-0.1580	0.7231	0.9995	-2.1175	0.0051	0.0462	-1.6411	0.0043	0.0298
Tbc1d16	-0.4769	0.1561	0.9995	-1.3647	0.0037	0.0383	-1.6413	0.0002	0.0043
Gm3414	-0.1676	0.6112	0.9995	-1.5081	0.0015	0.0229	-1.6471	0.0003	0.0057
Kif16b	-0.0948	0.7660	0.9995	-1.2493	0.0036	0.0375	-1.6548	0.0002	0.0040
Jmjd7	-0.0889	0.8147	0.9995	-1.7941	0.0027	0.0318	-1.6568	0.0013	0.0138

Bbs2	-0.5759	0.2366	0.9995	-2.2551	0.0047	0.0439	-1.6590	0.0052	0.0342
Ccni	0.0239	0.9087	0.9995	-1.7998	0.0000	0.0002	-1.6606	0.0000	0.0001
Sema3e	0.4100	0.1648	0.9995	-1.2360	0.0006	0.0140	-1.6638	0.0000	0.0012
Tbc1d22a	-0.0575	0.8354	0.9995	-1.7332	0.0001	0.0046	-1.6678	0.0000	0.0015
Gys1	-0.3906	0.3396	0.9995	-1.6489	0.0024	0.0301	-1.6684	0.0007	0.0091
Hist1h4b	0.6695	0.1888	0.9995	-2.9731	0.0012	0.0207	-1.6716	0.0166	0.0725
Patj	-0.3893	0.1261	0.9995	-1.2038	0.0006	0.0144	-1.6721	0.0000	0.0005
F730043M19Rik	-0.5150	0.3090	0.9995	-0.4212	0.4661	0.6771	-1.6832	0.0060	0.0376
Rasa4	0.1764	0.5513	0.9995	-1.4317	0.0004	0.0119	-1.6836	0.0001	0.0020
Cd109	0.1041	0.7981	0.9995	-1.8386	0.0025	0.0303	-1.6844	0.0030	0.0236
Pdcd4	-0.3948	0.2963	0.9995	-1.5874	0.0018	0.0249	-1.6867	0.0003	0.0061
Pdk1	0.2392	0.4576	0.9995	-2.4214	0.0000	0.0012	-1.6959	0.0001	0.0024
2700054A10Rik	-0.6531	0.1346	0.9995	-1.4145	0.0234	0.1146	-1.7040	0.0014	0.0146
Fgfbp1	0.0194	0.9514	0.9995	-1.0907	0.0057	0.0495	-1.7080	0.0001	0.0030
Tspan17	0.2005	0.6095	0.9995	-1.1618	0.0186	0.0991	-1.7105	0.0018	0.0175
Mindy4	-0.4682	0.3785	0.9995	-3.2942	0.0008	0.0169	-1.7151	0.0066	0.0399
Ralgapa2	-0.4732	0.1255	0.9995	-0.8750	0.0194	0.1015	-1.7206	0.0000	0.0016
Prkra	0.0183	0.9393	0.9995	-1.7188	0.0000	0.0018	-1.7225	0.0000	0.0004
Dubr	-0.9667	0.0829	0.9995	0.3454	0.5497	0.7406	-1.7242	0.0049	0.0326
Jarid2	0.0525	0.8858	0.9995	-1.6973	0.0019	0.0257	-1.7245	0.0005	0.0079
Cdkn2c	-0.2950	0.3864	0.9995	-1.5260	0.0010	0.0188	-1.7248	0.0001	0.0034
Rgs11	-0.0254	0.9588	0.9995	-3.4207	0.0008	0.0165	-1.7263	0.0084	0.0466
Cracr2b	-0.4665	0.3323	0.9995	-2.2418	0.0074	0.0569	-1.7287	0.0031	0.0241
Arhgef19	-0.4996	0.0932	0.9995	-0.6890	0.0439	0.1669	-1.7299	0.0000	0.0012
Enox2	-0.2643	0.3905	0.9995	-1.4893	0.0006	0.0138	-1.7347	0.0000	0.0018
Snd1	-0.1709	0.6467	0.9995	-0.9660	0.0277	0.1262	-1.7370	0.0003	0.0062
Smg6	-0.4969	0.0510	0.9995	-1.2895	0.0002	0.0081	-1.7411	0.0000	0.0003
Lmf1	0.0086	0.9856	0.9995	-1.5253	0.0209	0.1064	-1.7464	0.0058	0.0368
Immp2l	0.5982	0.1445	0.9995	-2.3200	0.0005	0.0130	-1.7530	0.0028	0.0226
Neat1	-0.2630	0.4702	0.9995	-1.5491	0.0037	0.0383	-1.7572	0.0003	0.0058
Gbe1	0.2695	0.4991	0.9995	-1.7655	0.0009	0.0175	-1.7608	0.0005	0.0082
Syne2	-0.5089	0.0658	0.9995	-1.2903	0.0002	0.0085	-1.7766	0.0000	0.0003
Papss1	-0.5277	0.2701	0.9995	-1.6638	0.0183	0.0983	-1.7805	0.0024	0.0204
Lpin1	-0.1817	0.6722	0.9995	-1.7023	0.0033	0.0355	-1.7855	0.0011	0.0129
Nbas	-0.7345	0.0825	0.9995	-0.3692	0.4222	0.6401	-1.7886	0.0007	0.0098
Per3	-0.5067	0.1921	0.9995	-2.3488	0.0007	0.0159	-1.7908	0.0004	0.0072
Slc2a8	-0.0868	0.8487	0.9995	-1.3871	0.0175	0.0958	-1.7917	0.0036	0.0265
Gtdc1	0.0826	0.8275	0.9995	-2.0837	0.0010	0.0185	-1.7918	0.0011	0.0129
Nmnat2	-0.0226	0.9495	0.9995	-2.1570	0.0003	0.0106	-1.8010	0.0003	0.0062
Aatk	0.0014	0.9964	0.9995	-2.7478	0.0000	0.0022	-1.8016	0.0001	0.0032
Selenon	-0.3309	0.3121	0.9995	-1.1055	0.0081	0.0605	-1.8038	0.0001	0.0032
Ephb2	0.2391	0.4778	0.9995	-2.2834	0.0001	0.0038	-1.8096	0.0003	0.0053
Hpcal1	-0.5377	0.0994	0.9995	-1.2892	0.0015	0.0227	-1.8109	0.0000	0.0011
Dbn1	0.0039	0.9904	0.9995	-1.7921	0.0002	0.0088	-1.8162	0.0001	0.0030
Acox2	-0.4094	0.3464	0.9995	-0.8327	0.1079	0.2859	-1.8173	0.0017	0.0166
Pcbp2	-0.4408	0.1487	0.9995	-1.0226	0.0041	0.0408	-1.8213	0.0000	0.0007
Acsl3	-0.3005	0.3155	0.9995	-1.4912	0.0002	0.0070	-1.8230	0.0000	0.0006
Pam	-0.1747	0.4697	0.9995	-1.2823	0.0001	0.0059	-1.8330	0.0000	0.0002
Vamp2	-0.7358	0.1776	0.9995	-0.2942	0.6192	0.7872	-1.8340	0.0044	0.0306
Fbxo24	-0.0200	0.9622	0.9995	-0.8204	0.0948	0.2656	-1.8351	0.0021	0.0187
Mtap	-0.1732	0.3583	0.9995	-1.5593	0.0000	0.0002	-1.8355	0.0000	0.0000

Sil1	0.1037	0.7493	0.9995	-2.5563	0.0000	0.0022	-1.8375	0.0001	0.0031
Thsd4	-0.0492	0.8905	0.9995	-2.1144	0.0005	0.0129	-1.8421	0.0005	0.0077
Gm16617	-0.4394	0.3031	0.9995	-1.1063	0.0413	0.1613	-1.8447	0.0009	0.0112
Epb41l1	-0.3535	0.2666	0.9995	-1.2610	0.0013	0.0213	-1.8456	0.0000	0.0010
Fam172a	-0.0587	0.8169	0.9995	-1.7618	0.0000	0.0032	-1.8521	0.0000	0.0006
Exoc7	-0.1626	0.5606	0.9995	-1.6722	0.0001	0.0051	-1.8558	0.0000	0.0007
Sfxn3	0.1428	0.5579	0.9995	-2.0616	0.0000	0.0006	-1.8669	0.0000	0.0002
Trpm4	0.2058	0.4629	0.9995	-1.2870	0.0009	0.0172	-1.8674	0.0000	0.0017
Fto	0.1272	0.6877	0.9995	-1.8216	0.0001	0.0068	-1.8722	0.0001	0.0020
Ephb6	-1.0721	0.0372	0.9995	-2.9501	0.0029	0.0331	-1.8726	0.0014	0.0146
Smarcd3	-0.4602	0.1845	0.9995	-0.8338	0.0404	0.1593	-1.8736	0.0001	0.0023
Pdzd4	0.1068	0.7784	0.9995	-2.3901	0.0004	0.0122	-1.8741	0.0009	0.0110
Eno2	-0.3369	0.4137	0.9995	-1.7464	0.0047	0.0441	-1.8812	0.0007	0.0098
Abcd4	0.3776	0.3952	0.9995	-2.1448	0.0019	0.0260	-1.8819	0.0031	0.0241
Hyi	0.3406	0.2724	0.9995	-2.3234	0.0000	0.0020	-1.8834	0.0000	0.0015
Dis3l2	0.2093	0.6838	0.9995	-2.7690	0.0014	0.0221	-1.9009	0.0078	0.0449
Deptor	-0.7520	0.0564	0.9995	-0.5440	0.2001	0.4154	-1.9113	0.0001	0.0032
Kdm7a	-0.4502	0.1291	0.9995	-1.8673	0.0001	0.0069	-1.9117	0.0000	0.0007
Dym	-0.2405	0.3905	0.9995	-0.9910	0.0062	0.0517	-1.9152	0.0000	0.0009
Jpx	-1.2864	0.0394	0.9995	0.4036	0.5206	0.7172	-1.9198	0.0033	0.0250
Bhlhe41	-0.1733	0.6016	0.9995	-2.3724	0.0000	0.0034	-1.9211	0.0000	0.0018
Gpi1	-0.2304	0.3916	0.9995	-1.6663	0.0000	0.0019	-1.9292	0.0000	0.0002
Mthfd2l	0.0227	0.9467	0.9995	-1.0943	0.0107	0.0716	-1.9377	0.0001	0.0036
Matn2	0.6656	0.1251	0.9995	-1.1484	0.0197	0.1026	-1.9460	0.0028	0.0224
Rhobtb1	-0.2468	0.5993	0.9995	-3.6998	0.0005	0.0123	-1.9467	0.0025	0.0210
Fggy	0.0013	0.9968	0.9995	-1.8657	0.0002	0.0086	-1.9571	0.0001	0.0020
Ebpl	0.0917	0.8340	0.9995	-1.4373	0.0115	0.0753	-1.9597	0.0019	0.0180
Bcl11a	-0.4630	0.4979	0.9995	-3.9559	0.0030	0.0336	-1.9615	0.0179	0.0761
Lfng	-0.4293	0.4009	0.9995	-1.9404	0.0136	0.0828	-1.9646	0.0035	0.0259
Bcl9	0.0279	0.9720	0.9995	-4.2836	0.0025	0.0304	-1.9695	0.0387	0.1250
Ero1l	0.1363	0.5843	0.9995	-1.7676	0.0000	0.0011	-1.9710	0.0000	0.0001
Asph	-0.3896	0.1437	0.9995	-1.5080	0.0001	0.0052	-1.9715	0.0000	0.0002
Lyst	-0.7769	0.0279	0.9995	-0.7228	0.0632	0.2061	-1.9716	0.0000	0.0011
Supt3	0.0606	0.8518	0.9995	-1.8838	0.0002	0.0078	-1.9772	0.0000	0.0018
Acacb	-0.6912	0.0750	0.9995	-0.7469	0.1125	0.2939	-1.9812	0.0002	0.0039
Mpst	-0.5997	0.2208	0.9995	-1.5641	0.0177	0.0961	-1.9812	0.0012	0.0131
Hgsnat	-0.6494	0.0971	0.9995	-0.8051	0.0910	0.2595	-1.9900	0.0002	0.0044
Mpnd	-0.0680	0.8327	0.9995	-2.0674	0.0001	0.0049	-2.0018	0.0000	0.0012
Pfkl	-0.1517	0.5717	0.9995	-1.8878	0.0000	0.0011	-2.0149	0.0000	0.0001
Gatd1	-0.1524	0.7367	0.9995	-1.4395	0.0214	0.1079	-2.0220	0.0019	0.0176
Sema4g	0.4687	0.5755	0.9995	-4.0505	0.0053	0.0473	-2.0221	0.0546	0.1578
Etv1	-0.0681	0.7584	0.9995	-1.4100	0.0000	0.0026	-2.0248	0.0000	0.0001
Car9	0.6658	0.3921	0.9995	-3.2501	0.0020	0.0265	-2.0343	0.0201	0.0822
Scd2	-0.6572	0.1569	0.9995	-0.8094	0.0891	0.2560	-2.0396	0.0002	0.0039
Snord37	1.4288	0.1320	0.9995	-3.7884	0.0051	0.0465	-2.0487	0.0991	0.2339
Zfp395	-0.3324	0.2403	0.9995	-1.9381	0.0001	0.0043	-2.0513	0.0000	0.0005
Dmpk	-0.2049	0.5042	0.9995	-1.5612	0.0006	0.0139	-2.0526	0.0000	0.0011
Guca1a	0.2655	0.4782	0.9995	-2.4057	0.0003	0.0094	-2.0534	0.0005	0.0079
Sptbn2	-0.5482	0.2772	0.9995	-3.1306	0.0032	0.0351	-2.0548	0.0023	0.0202
Pgf	-0.1427	0.7089	0.9995	-0.7625	0.0858	0.2506	-2.0601	0.0002	0.0046
Igf2bp2	-0.4334	0.0804	0.9995	-1.4085	0.0000	0.0026	-2.0643	0.0000	0.0000

Steap4	-1.9337	0.1529	0.9995	4.8392	0.0004	0.0121	-2.0784	0.0999	0.2354
Plod1	-0.4942	0.2125	0.9995	-1.6510	0.0038	0.0390	-2.0836	0.0001	0.0034
Ptprk	-0.0374	0.8777	0.9995	-1.8293	0.0000	0.0011	-2.0853	0.0000	0.0001
Nfia	-0.0703	0.7426	0.9995	-2.2465	0.0000	0.0001	-2.0864	0.0000	0.0000
Tpm2	-0.2694	0.5452	0.9995	-1.6491	0.0095	0.0663	-2.0914	0.0010	0.0119
Mfsd13a	0.2466	0.7209	0.9995	-5.1137	0.0014	0.0222	-2.0916	0.0242	0.0927
Parp16	-0.1863	0.6238	0.9995	-2.7304	0.0002	0.0073	-2.0923	0.0002	0.0051
Rnpepl1	-0.1931	0.5884	0.9995	-1.2193	0.0089	0.0641	-2.0925	0.0001	0.0026
Bend5	-0.5492	0.0435	0.9995	-1.7567	0.0000	0.0030	-2.0942	0.0000	0.0001
Fut8	-0.6476	0.0672	0.9995	-1.5094	0.0018	0.0255	-2.0999	0.0000	0.0011
Galk1	-0.2223	0.5056	0.9995	-1.6121	0.0003	0.0100	-2.1032	0.0000	0.0007
Aldoart1	-0.6704	0.1828	0.9995	-1.5920	0.0195	0.1019	-2.1039	0.0010	0.0120
Slc4a8	0.6049	0.1084	0.9995	-2.7398	0.0001	0.0048	-2.1106	0.0004	0.0064
Tlr5	1.4182	0.2370	0.9995	-4.7575	0.0056	0.0491	-2.1113	0.1502	0.3038
Krt19	0.2507	0.3635	0.9995	-2.2431	0.0000	0.0005	-2.1166	0.0000	0.0002
Unc13a	-0.8258	0.0867	0.9995	-1.1143	0.0708	0.2209	-2.1269	0.0008	0.0105
Mthfd1l	-0.1885	0.4878	0.9995	-1.6957	0.0000	0.0026	-2.1348	0.0000	0.0001
Ankrd37	-0.2034	0.6785	0.9995	-3.4818	0.0008	0.0165	-2.1445	0.0016	0.0166
Pgm1	-0.1352	0.6678	0.9995	-1.9708	0.0000	0.0030	-2.1569	0.0000	0.0004
Snord13	-0.4695	0.4841	0.9995	-0.6616	0.3324	0.5576	-2.1618	0.0031	0.0240
Large1	0.1497	0.7423	0.9995	-2.8239	0.0007	0.0153	-2.1637	0.0018	0.0174
Zfp775	-0.3201	0.5194	0.9995	-1.6348	0.0330	0.1405	-2.1799	0.0019	0.0176
Aldoa	-0.1333	0.5560	0.9995	-2.0094	0.0000	0.0002	-2.1888	0.0000	0.0000
Stag1	-0.2470	0.3018	0.9995	-1.7259	0.0000	0.0018	-2.2025	0.0000	0.0001
Egln3	0.0598	0.8706	0.9995	-1.6017	0.0014	0.0221	-2.2038	0.0001	0.0020
Adprh	-0.0237	0.9142	0.9995	-1.7699	0.0000	0.0011	-2.2055	0.0000	0.0001
Ptges	-0.3063	0.5281	0.9995	-0.7899	0.1724	0.3804	-2.2175	0.0020	0.0185
Usp11	0.1996	0.5806	0.9995	-2.8536	0.0000	0.0031	-2.2177	0.0001	0.0024
Trib2	-0.1877	0.5905	0.9995	-2.2942	0.0001	0.0067	-2.2263	0.0000	0.0018
Mirlet7b	0.3670	0.6276	0.9995	-4.7454	0.0010	0.0184	-2.2366	0.0231	0.0901
Gm11110	-0.4060	0.3084	0.9995	-2.8773	0.0004	0.0117	-2.2594	0.0002	0.0046
Six5	-0.3642	0.5019	0.9995	-1.6217	0.0339	0.1427	-2.2681	0.0031	0.0239
Hist1h1c	-0.1195	0.7619	0.9995	-3.7367	0.0000	0.0025	-2.2688	0.0001	0.0030
Sh2d3c	0.3686	0.4409	0.9995	-2.8771	0.0010	0.0191	-2.3121	0.0021	0.0191
Samd14	-0.6987	0.2076	0.9995	-1.2177	0.0956	0.2668	-2.3142	0.0020	0.0183
Scaper	-0.1285	0.6421	0.9995	-2.5474	0.0000	0.0007	-2.3413	0.0000	0.0001
Acsf3	-0.5866	0.3251	0.9995	-2.6202	0.0097	0.0670	-2.3421	0.0033	0.0252
Crocc	-0.5790	0.1865	0.9995	-1.0134	0.0682	0.2154	-2.3667	0.0002	0.0051
Trps1	0.0227	0.9197	0.9995	-2.1745	0.0000	0.0001	-2.3709	0.0000	0.0000
Cda	0.4931	0.1846	0.9995	-0.8865	0.0329	0.1402	-2.3785	0.0002	0.0050
Spa17	-0.2822	0.3388	0.9995	-2.0960	0.0000	0.0022	-2.3959	0.0000	0.0002
Acvr2b	-0.1352	0.7442	0.9995	-1.5642	0.0081	0.0605	-2.4052	0.0004	0.0068
Spred3	-0.0678	0.9132	0.9995	-2.0932	0.0192	0.1009	-2.4092	0.0052	0.0342
Fars2	-0.5473	0.3552	0.9995	-1.1820	0.1437	0.3409	-2.4344	0.0030	0.0236
Cdk14	-0.2289	0.3478	0.9995	-2.2583	0.0000	0.0005	-2.4351	0.0000	0.0000
Sbf2	-0.2684	0.2566	0.9995	-1.8295	0.0000	0.0012	-2.4368	0.0000	0.0000
Bmp7	0.1684	0.7301	0.9995	-2.1073	0.0041	0.0407	-2.4531	0.0012	0.0134
Kazald1	0.9007	0.3952	0.9995	-4.6625	0.0052	0.0468	-2.4725	0.0703	0.1861
Pde4d	-0.4139	0.3575	0.9995	-1.7024	0.0107	0.0716	-2.4923	0.0004	0.0072
Rorc	0.0855	0.8095	0.9995	-3.1386	0.0001	0.0046	-2.4963	0.0001	0.0026
Hist1h2ba	1.0238	0.2197	0.9995	-4.3141	0.0014	0.0223	-2.5080	0.0249	0.0947

Lrrc75b	-1.0592	0.0569	0.9995	-3.7132	0.0027	0.0321	-2.5118	0.0006	0.0086
Galt	-0.8672	0.1254	0.9995	0.0767	0.8976	0.9520	-2.5352	0.0008	0.0103
Macrod1	0.4301	0.2451	0.9995	-1.9326	0.0008	0.0160	-2.5466	0.0002	0.0040
Hist1h2br	0.4064	0.4581	0.9995	-4.9573	0.0002	0.0079	-2.5480	0.0032	0.0247
Hist1h2bq	0.4064	0.4558	0.9995	-4.9573	0.0002	0.0077	-2.5786	0.0030	0.0233
Gja1	-0.4820	0.3382	0.9995	-0.6115	0.2670	0.4926	-2.5913	0.0003	0.0054
Cxcr3	-1.8805	0.0236	0.9995	1.6482	0.0526	0.1865	-2.6058	0.0033	0.0249
Opn3	0.6528	0.2374	0.9995	-2.2352	0.0051	0.0461	-2.6251	0.0031	0.0241
Bnip3l-ps	-0.3462	0.5956	0.9995	-0.8180	0.2932	0.5209	-2.6360	0.0069	0.0408
Dync2h1	-0.2018	0.5171	0.9995	-2.0207	0.0001	0.0049	-2.6365	0.0000	0.0003
Paqr5	-1.3981	0.1128	0.9995	-0.0409	0.9668	0.9852	-2.6387	0.0090	0.0488
Cib2	-0.1373	0.7916	0.9995	-0.6240	0.2815	0.5081	-2.6391	0.0010	0.0118
Sergef	-0.4396	0.4628	0.9995	-0.9037	0.2232	0.4444	-2.6402	0.0021	0.0187
Bnip3	-0.2390	0.3589	0.9995	-2.7762	0.0000	0.0001	-2.6434	0.0000	0.0000
Hist2h2ac	-0.5919	0.3524	0.9995	-1.7476	0.0469	0.1739	-2.6505	0.0023	0.0197
Gpr146	-0.7001	0.0839	0.9995	-3.4190	0.0000	0.0038	-2.6602	0.0000	0.0012
Cldn9	-0.2111	0.5656	0.9995	-3.3005	0.0001	0.0054	-2.6760	0.0001	0.0020
Dsc2	-0.1838	0.7227	0.9995	-1.0108	0.1158	0.2989	-2.6828	0.0012	0.0132
Vwa1	0.0095	0.9869	0.9995	-2.1382	0.0213	0.1077	-2.6894	0.0039	0.0282
Adamtsl3	-1.0288	0.1424	0.9995	-2.5120	0.0206	0.1053	-2.7288	0.0028	0.0224
Dhx34	-0.0075	0.9851	0.9995	-1.7914	0.0028	0.0321	-2.7402	0.0002	0.0039
Hoxa3	-1.0683	0.2230	0.9995	-1.0416	0.3264	0.5523	-2.7457	0.0084	0.0467
2810408A11Rik	0.4666	0.4346	0.9995	-3.9212	0.0016	0.0237	-2.7767	0.0053	0.0345
Mnd1	0.4766	0.1417	0.9995	-2.7901	0.0000	0.0016	-2.7774	0.0000	0.0006
Ino80dos	-2.1756	0.0291	0.9995	1.3738	0.1812	0.3915	-2.8005	0.0057	0.0364
Fam196a	-2.2828	0.0378	0.9995	0.2845	0.8115	0.9042	-2.8279	0.0077	0.0444
Hist1h2al	-0.5563	0.2744	0.9995	-2.2258	0.0039	0.0395	-2.8329	0.0001	0.0036
Nthl1	-0.2301	0.7489	0.9995	-1.1781	0.2025	0.4182	-2.8346	0.0060	0.0376
Rnu2-10	0.2682	0.5752	0.9995	-1.1568	0.0428	0.1648	-2.8457	0.0009	0.0112
Gm4876	-3.9823	0.0053	0.9995	3.9697	0.0051	0.0463	-2.8570	0.0123	0.0603
Hist2h2aa1	-0.5838	0.2392	0.9995	-2.3771	0.0020	0.0260	-2.8597	0.0001	0.0026
Tle6	-0.7903	0.0853	0.9995	-2.9662	0.0009	0.0178	-2.8659	0.0001	0.0025
Pfkp	-0.4251	0.1915	0.9995	-2.6261	0.0000	0.0011	-2.8747	0.0000	0.0001
Gm9159	1.6280	0.0872	0.9995	-3.9422	0.0020	0.0265	-2.9366	0.0162	0.0718
Hist2h2aa2	-0.5631	0.2592	0.9995	-2.4391	0.0020	0.0261	-2.9376	0.0001	0.0026
Prss8	-0.2725	0.4429	0.9995	-2.3563	0.0005	0.0129	-2.9566	0.0000	0.0010
Nectin4	-1.0190	0.0341	0.9995	-1.6629	0.0189	0.0998	-2.9589	0.0001	0.0025
Bmf	0.3869	0.5264	0.9995	-2.1747	0.0163	0.0916	-2.9955	0.0057	0.0364
Gabbr1	-0.5204	0.2655	0.9995	-2.3236	0.0055	0.0482	-3.0122	0.0002	0.0042
Mnd1-ps	0.3795	0.2162	0.9995	-4.1324	0.0000	0.0008	-3.0195	0.0000	0.0006
Prelid2	0.2095	0.6098	0.9995	-2.9670	0.0001	0.0046	-3.0334	0.0000	0.0018
Hist1h1e	-0.1256	0.6438	0.9995	-3.0399	0.0000	0.0001	-3.0350	0.0000	0.0000
Gstm1	-0.0945	0.7487	0.9995	-1.5279	0.0003	0.0106	-3.0460	0.0000	0.0002
Naglu	-0.2646	0.5444	0.9995	-3.3987	0.0002	0.0092	-3.0842	0.0001	0.0023
Rab3d	-0.4059	0.4933	0.9995	-1.6925	0.0486	0.1776	-3.0854	0.0013	0.0140
Carf	-1.0890	0.1966	0.9995	-3.6945	0.0119	0.0769	-3.1015	0.0036	0.0264
9230105E05Rik	-1.0135	0.2227	0.9995	-3.1263	0.0153	0.0886	-3.1132	0.0034	0.0254
Hps1	-4.0201	0.0026	0.9995	3.8695	0.0036	0.0374	-3.1453	0.0045	0.0311
Hist1h2ac	-0.3272	0.4061	0.9995	-2.9575	0.0000	0.0033	-3.1519	0.0000	0.0004
Hist1h2ab	-0.4269	0.3656	0.9995	-2.7812	0.0004	0.0119	-3.1724	0.0000	0.0014
Adm	0.2881	0.5257	0.9995	-5.4566	0.0002	0.0088	-3.2332	0.0001	0.0037

Adssl1	-0.6089	0.0772	0.9995	-2.7457	0.0001	0.0052	-3.2362	0.0000	0.0004
1110046J04Rik	0.1401	0.8364	0.9995	-1.0079	0.1973	0.4123	-3.2508	0.0022	0.0193
A430090L17Rik	-3.8182	0.0024	0.9995	-0.0729	0.9553	0.9821	-3.2602	0.0033	0.0250
Hist1h2bj	1.3986	0.0831	0.9995	-5.4413	0.0006	0.0148	-3.2604	0.0115	0.0574
Hist1h1d	-0.6572	0.2162	0.9995	-2.6901	0.0065	0.0529	-3.2649	0.0004	0.0064
Hist1h2an	-0.2380	0.6552	0.9995	-2.8347	0.0051	0.0462	-3.2826	0.0009	0.0110
Hist1h2ad	-0.3438	0.4273	0.9995	-2.9423	0.0001	0.0063	-3.2877	0.0000	0.0007
Hist1h2ah	-0.2703	0.5168	0.9995	-3.0264	0.0001	0.0059	-3.3006	0.0000	0.0008
Nek3	-0.1734	0.7732	0.9995	-1.7997	0.0503	0.1815	-3.3084	0.0022	0.0197
2610203C22Rik	-0.6926	0.2907	0.9995	-1.1300	0.1732	0.3817	-3.3185	0.0007	0.0097
Iqcd	-1.1512	0.1994	0.9995	-3.2316	0.0230	0.1134	-3.3270	0.0040	0.0286
Hist1h1b	-0.3072	0.2344	0.9995	-1.9354	0.0000	0.0020	-3.3667	0.0000	0.0000
Hist1h2ag	-0.3099	0.4146	0.9995	-2.9506	0.0000	0.0026	-3.3746	0.0000	0.0002
Hist1h2bb	0.8233	0.3561	0.9995	-4.2620	0.0024	0.0300	-3.3797	0.0090	0.0489
Hist1h2ai	-0.4551	0.3213	0.9995	-2.8189	0.0004	0.0114	-3.4054	0.0000	0.0010
H1f0	-0.2131	0.4370	0.9995	-3.5892	0.0000	0.0000	-3.4097	0.0000	0.0000
Dbndd1	-0.4808	0.5378	0.9995	-0.9698	0.2846	0.5117	-3.4144	0.0019	0.0177
Man1c1	-0.1450	0.8379	0.9995	-5.3355	0.0008	0.0166	-3.4197	0.0024	0.0205
Ptch1	-1.9330	0.0783	0.9995	1.9034	0.0863	0.2511	-3.4409	0.0056	0.0358
Hist1h2ao	-0.5695	0.2512	0.9995	-2.6973	0.0001	0.0040	-3.4518	0.0000	0.0002
Hist1h2ap	-0.5695	0.2511	0.9995	-2.6972	0.0001	0.0040	-3.4555	0.0000	0.0002
Megf6	-0.5718	0.1923	0.9995	-2.8680	0.0013	0.0211	-3.4578	0.0000	0.0019
Hist1h2be	0.3089	0.6975	0.9995	-2.1441	0.0464	0.1728	-3.4737	0.0070	0.0414
Zfp820	-1.0637	0.2679	0.9995	-3.2306	0.0283	0.1278	-3.4753	0.0046	0.0314
Lrrc26	0.3549	0.6794	0.9995	-4.3751	0.0017	0.0245	-3.5172	0.0046	0.0315
Sspn	-0.2261	0.4063	0.9995	-3.1815	0.0000	0.0002	-3.5543	0.0000	0.0000
Add2	-1.0608	0.0905	0.9995	-1.4349	0.0999	0.2739	-3.6042	0.0005	0.0077
Hist1h2ae	-0.5291	0.3010	0.9995	-2.7677	0.0007	0.0155	-3.6570	0.0000	0.0011
Hist1h2bp	-0.1831	0.8293	0.9995	-3.5474	0.0150	0.0878	-3.6715	0.0052	0.0342
Ano8	-1.2227	0.2937	0.9995	-0.7023	0.6028	0.7760	-3.6903	0.0087	0.0477
Rnu11	0.0863	0.9130	0.9995	-1.6558	0.0888	0.2557	-3.6987	0.0022	0.0197
Espn	-1.0301	0.0257	0.9995	-3.2246	0.0001	0.0069	-3.6994	0.0000	0.0003
Hist2h3b	-0.8998	0.3492	0.9995	-1.8157	0.1637	0.3699	-3.7700	0.0036	0.0267
Gstt1	0.0615	0.9148	0.9995	-1.9637	0.0250	0.1190	-3.7848	0.0006	0.0082
Pou6f1	0.0765	0.9096	0.9995	-2.8022	0.0149	0.0873	-3.8013	0.0013	0.0141
Slc26a9	-0.5783	0.4367	0.9995	-1.3838	0.1439	0.3413	-3.8221	0.0009	0.0113
Hist2h2ab	-0.9900	0.1787	0.9995	-3.0284	0.0276	0.1260	-3.9123	0.0022	0.0197
Mgarp	-0.3591	0.5949	0.9995	-6.1243	0.0005	0.0123	-3.9832	0.0011	0.0122
Tmem53	-0.6695	0.3128	0.9995	-1.1650	0.1695	0.3764	-4.1247	0.0003	0.0058
Hist2h2bb	-0.2969	0.7216	0.9995	-2.1086	0.0934	0.2636	-4.1492	0.0053	0.0346
Aldoc	-0.0775	0.7997	0.9995	-4.2177	0.0000	0.0005	-4.1845	0.0000	0.0001
Sh2d7	-1.2662	0.0782	0.9995	-1.8163	0.0688	0.2166	-4.3251	0.0001	0.0033
Hist1h2bn	0.2262	0.7860	0.9995	-3.5323	0.0084	0.0616	-4.4178	0.0016	0.0159
Krt15	-0.1517	0.7661	0.9995	-6.3512	0.0002	0.0071	-4.5763	0.0002	0.0039
Hist1h2bk	0.8699	0.2342	0.9995	-3.3667	0.0040	0.0403	-4.6521	0.0017	0.0168
Tmem191c	-0.4976	0.6597	0.9995	-2.2857	0.1235	0.3117	-4.6557	0.0082	0.0459
Arhgap33	-1.7427	0.0945	0.9995	-3.2528	0.0367	0.1495	-5.0672	0.0005	0.0073
