# Supplementary material for: IL-17A Increases Doxorubicin Efficacy in Triple Negative Breast Cancer
Source: Front Oncol. 2022 Jul 18;12:928474. doi: 10.3389/fonc.2022.928474 (PMC9340269; doi:10.3389/fonc.2022.928474)
Supplement: Supplementary file 2 [file DataSheet_2.docx]

## Supplementary Figures


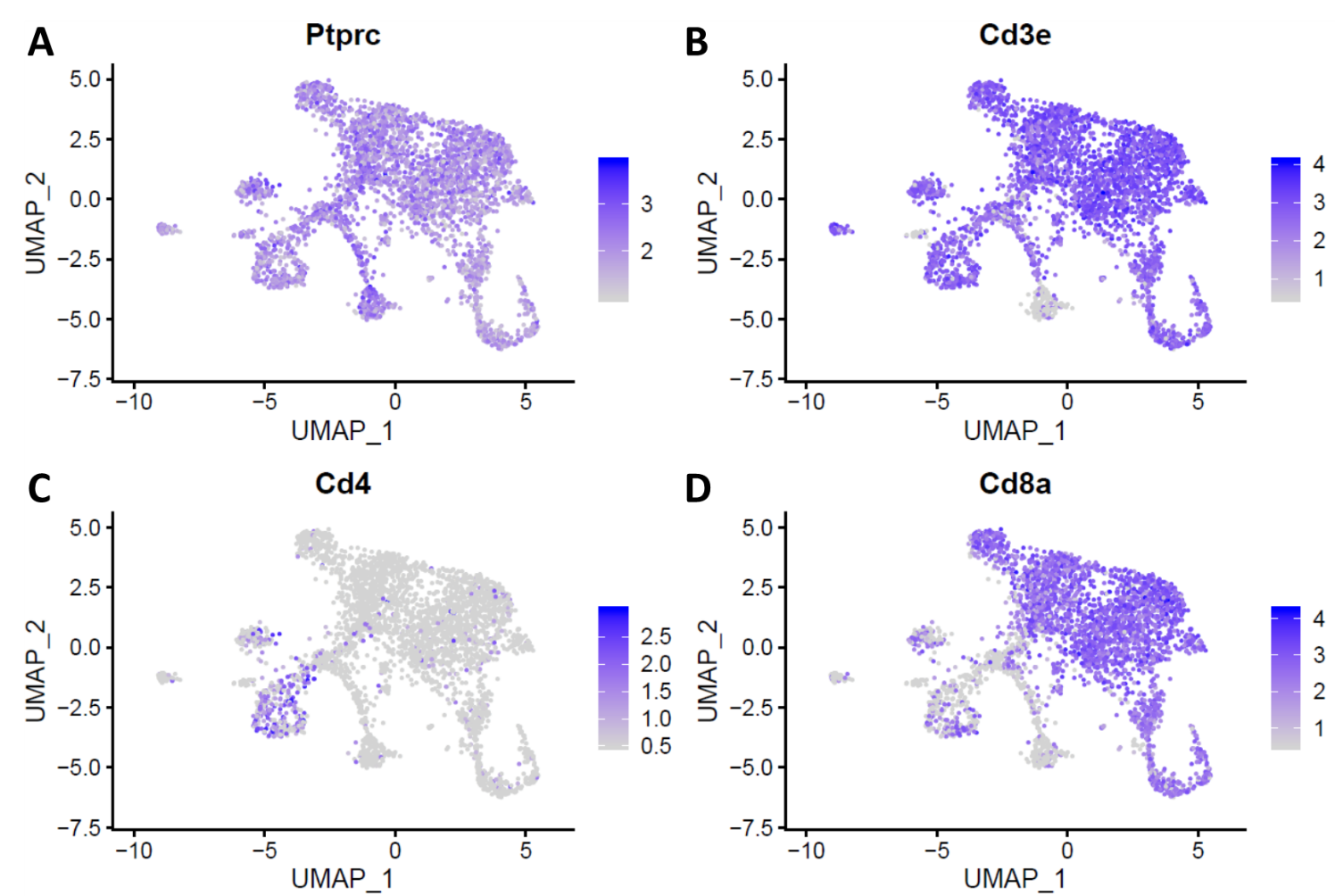


Supplemental Figure 1. Feature plots denoting T cell fold change of gene expression values. Plots denoting cellular expression of A) Ptprc (CD45 expression), B) CD3ε, C) CD4, and D) CD8a in tumor infiltrated T cell populations.


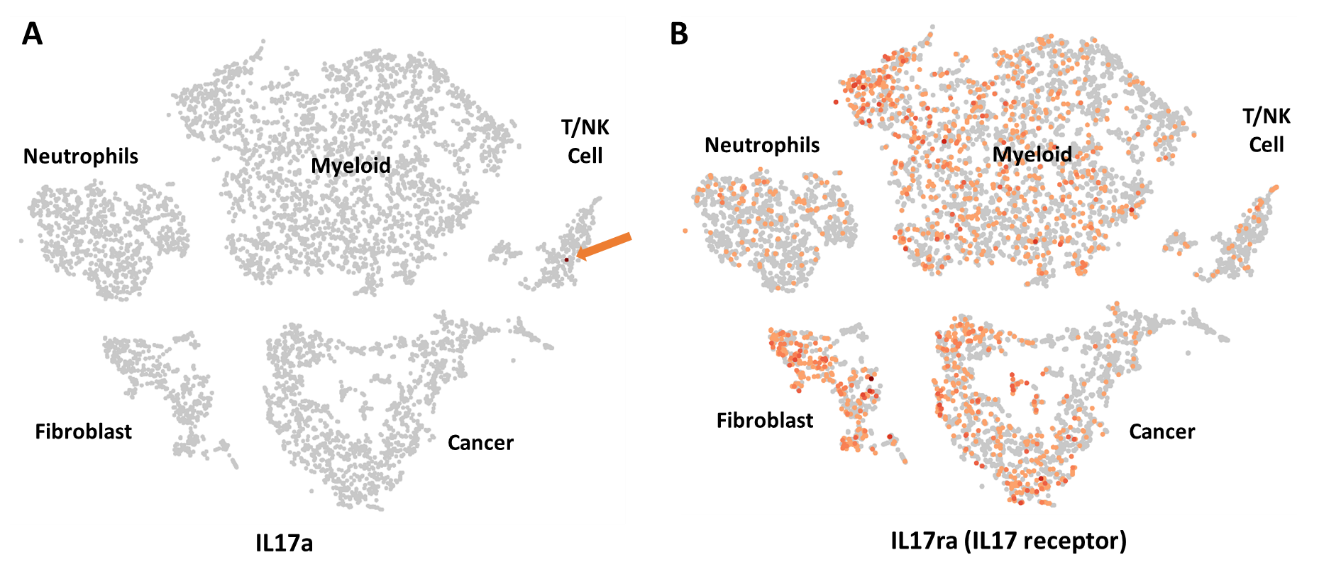


Supplemental Figure 2. UMAP projection of 4T1 syngeneic tumor cells. A) IL-17A expression restricted to IL-17+ T cells (arrowhead). B) IL-17 receptor (IL-17ra) is widely expressed across several cell types.
